# Evaluation of Multidentate Ligands Derived from Ethyl 1,2,4‐triazine‐3‐carboxylate Building Blocks as Potential An(III)‐Selective Extractants for Nuclear Reprocessing

**DOI:** 10.1002/open.202400306

**Published:** 2024-11-26

**Authors:** Andrey V. Zaytsev, Petr Distler, Jan John, Andreas Wilden, Giuseppe Modolo, Mark Sims, Frank W. Lewis

**Affiliations:** ^1^ Department of Applied Sciences Faculty of Health and Life Sciences Northumbria University Newcastle upon Tyne, Tyne and Wear NE1 8ST UK; ^2^ Department of Nuclear Chemistry Czech Technical University in Prague Břehová 7 11519 Prague 1 Czech Republic; ^3^ Forschungszentrum Jülich GmbH Institute of Fusion Energy and Nuclear Waste Management – Nuclear Waste Management (IFN-2) 52428 Jülich Germany

**Keywords:** Actinide, N,O ligands, Solvent extraction, Heterocycles, Nuclear reprocessing

## Abstract

Bis‐1,2,4‐triazine ligands are amongst the most promising soft *N*‐donor ligands for the partitioning of trivalent actinides from trivalent lanthanides; a key separation proposed in the future reprocessing of spent nuclear fuels. In an effort to improve the extraction properties of these benchmark ligands, we propose herein a general ligand design approach that is inspired by the field of drug discovery, and we apply it to a new class of ligands in which the bidentate 3‐(2‐pyridyl)‐1,2,4‐triazine unit of the benchmark ligands is replaced by a bidentate 1,2,4‐triazine‐3‐carboxamide unit. A series of nine novel ligands were synthesized by reactions of readily available ethyl 1,2,4‐triazine‐3‐carboxylate building blocks with different polyamine cores and evaluated for their ability to extract and separate Am(III) and Cm(III) from Eu(III). One of the reported ligands can co‐extract Am(III) and Eu(III) from nitric acid into cyclohexanone, albeit with no selectivity between the metal ions. NMR titration experiments suggested that ligand **23 b** formed a chiral 1 : 1 complex species with La(III) but not Lu(III) or Y(III), suggesting the coordination cavity of the ligand is sensitive to the size of the metal ion. The structures and thermodynamic parameters for the proposed complexes were further supported by DFT calculations.

## Graphical abstract

Several new N,O‐ligands containing the 1,2,4‐triazine‐3‐carboxamide chelating group were synthesized and evaluated as actinide selective extracting agents for reprocessing spent nuclear fuels. The likely complexes formed with lanthanides were investigated by ^1^H NMR titrations and DFT calculations. Our ligand design approach could aid in the design of future ligands to close the nuclear fuel cycle.

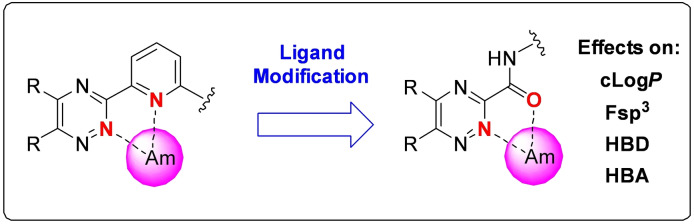

## Introduction

Electricity production from nuclear fission accounts for approx. 9 % of the world's energy needs today.^[1]^ However, spent fuel from nuclear reactors is radiotoxic and extremely hazardous, and is mainly composed of uranium and plutonium, and fission products. The PUREX process recovers and recycles uranium and plutonium which is re‐used as mixed‐oxide (MOX) fuel,^[2]^ but the remaining waste remains highly radiotoxic and long‐lived partly due to the presence of the minor actinides americium (Am), curium (Cm) and neptunium (Np). Removing the minor actinides would significantly reduce the long‐term heat load and radiotoxicity of the remaining waste for geological disposal from ca. 10^4^ years to a few hundred years. Advanced reprocessing of spent nuclear fuels therefore remains a crucial objective to support the current global resurgence in nuclear energy,^[3]^ and several solvent extraction flowsheets have been developed in recent years that remove all trans‐uranic actinides from spent nuclear fuels.^[4]^

**Figure 1:**
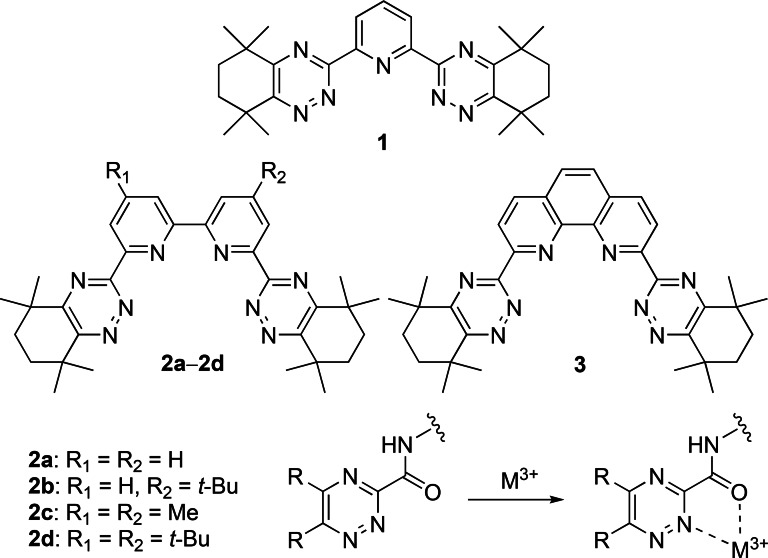
Structures of the benchmark bis‐1,2,4‐triazine ligands **1**–**3**, and the bidentate 1,2,4‐triazine‐3‐carboxamide chelating group.

The difficult separation of the minor actinides from the chemically similar lanthanide fission products generally requires soft *N*‐ or *S*‐donor ligands that can effectively discriminate between the two groups of elements, and numerous ligands have been evaluated for this separation by solvent extraction.^[5]^ The bis‐1,2,4‐triazine ligands **1**–**3** shown in Figure 1 are among the most promising and selective *N*‐donor ligands for this separation, and fulfil many of the challenging criteria for use in a solvent extraction process, including stability to hydrolysis in nitric acid, acceptable resistance to radiolysis, and high selectivity for extraction of trivalent actinides over trivalent lanthanides.^[6]^ However, the limited solubilities of these ligands in the diluents acceptable to the nuclear industry (e. g.: 1‐octanol, dodecane, kerosene), and their relatively slow extraction kinetics have hindered their further process development.

Efforts to simultaneously improve both ligand solubility and the extraction kinetics have been largely elusive. For example, alkylated derivatives **2 b**–**2 d** of the benchmark ligand **2 a** were synthesized containing one or more alkyl groups attached to the pyridine rings in an effort to increase ligand solubility.^[7]^ However, whilst this improved the ligand solubility only in the case of **2 b**, the extraction kinetics of this ligand were significantly slower than that of **2 a**, illustrating the limitations of this design approach. In another example, a less hydrophobic derivative of ligand **3** with 5‐membered aliphatic rings appended to the 1,2,4‐triazine rings showed slightly faster extraction kinetics, but marginally lower solubility in 1‐octanol than **3**.^[8]^ One notable exception is a camphor‐derived ligand similar to ligand **1** which showed both higher solubilities and more rapid extraction kinetics than ligands **1**–**3**.^[9]^ However, the ligand formed precipitates in contact with nitric acid which rendered further development infeasible, and the fundamental origins of its improved extraction properties over ligands **1**–**3** remain to be understood.

To overcome these limitations, we sought to develop a new and general ligand design approach inspired by the field of drug discovery that employs different physicochemical parameters to predict the likely success of drug candidates in clinical trials.^[10]^ These include the ‘Lipinski rule of 5’ parameters that are used to predict if a drug candidate will be orally active.^[11]^ In addition, a direct correlation has been observed between the degree of saturation of a drug and its solubility, suggesting that the fraction of saturated carbon atoms in a drug (Fsp^3^) can be used as a predictor of its solubility and hence its chances of clinical success.^[12]^

In the present context, this implies that ligands with too high a degree of unsaturation will have lower solubilities in diluents acceptable to the nuclear industry. Thus, reducing the number of aromatic rings present in bis‐1,2,4‐triazine ligands seemed a logical way to increase their solubilities. It was shown in a previous study that the faster rates of metal extraction exhibited by ligand **3** compared to ligand **2 a** were due to its higher surface concentrations at the interface, and its higher extraction rate constants compared to **2 a** in different diluents.^[6c]^ This was attributed to both the higher dipole moment of **3**, and the presence of water molecules in its coordination cavity which could take part in hydrogen‐bonding with water molecules at the phase interface. We therefore reasoned that the presence of hydrogen bond donor and acceptor (HBD and HBA) functional groups in a ligand will increase its polarity and could allow hydrogen bonding interactions with water molecules at the phase interface, potentially leading to higher ligand concentrations at the interface and hence faster rates of metal ion extraction. Thus, adding hydrogen bond donor and acceptor groups to a ligand could improve its extraction kinetics.

It was shown that replacing one of the lateral 1,2,4‐triazine rings in either ligand **1** or **2 a** with a pyridine ring leads to ligand systems which are completely unable to extract or separate the minor actinides Am(III) or Cm(III) from the lanthanides.^[13]^ Therefore, when considering which aromatic rings to remove or replace in ligand systems **1**–**3** (Figure 1) in order to increase ligand solubility, we decided to remove the pyridine/bipyridine/phenanthroline rings of ligands **1**–**3** and retain the crucial 1,2,4‐triazine rings.

Ethyl 1,2,4‐triazine‐3‐carboxylates seemed promising building blocks to employ as potential replacements for the pyridine/bipyridine/phenanthroline rings of ligands **1**–**3** as they are readily available in two steps from commercially available ethyl thiooxamate,^[14]^ and can be easily converted into 1,2,4‐triazine‐3‐carboxamides by reactions with different amines.^[15]^ This will naturally increase the Fsp^3^ values of the ligands, and permits further fine‐tuning of physicochemical properties through choice of the amine core. The 1,2,4‐triazine‐3‐carboxamide groups could serve as bidentate metal ion chelating groups in the multidentate ligands as illustrated in Figure 1, whilst introducing additional HBD and HBA groups to the ligands, thereby increasing ligand polarity and hence surface concentration at the phase interface. We decided in the present study to investigate novel multidentate 1,2,4‐triazine ligands in which the bidentate 3‐(2‐pyridyl)‐1,2,4‐triazine unit of ligands **1**–**3** is replaced with a 1,2,4‐triazine‐3‐carboxamide unit, and we report herein a range of novel 1,2,4‐triazine ligands derived from reactions of ethyl 1,2,4‐triazine‐3‐carboxylates with different polyamines that are commonly used as cores in multidentate ligand designs.^[16]^

## Results and Discussion

### Ligand Synthesis and Physicochemical Properties

**Scheme 1:**
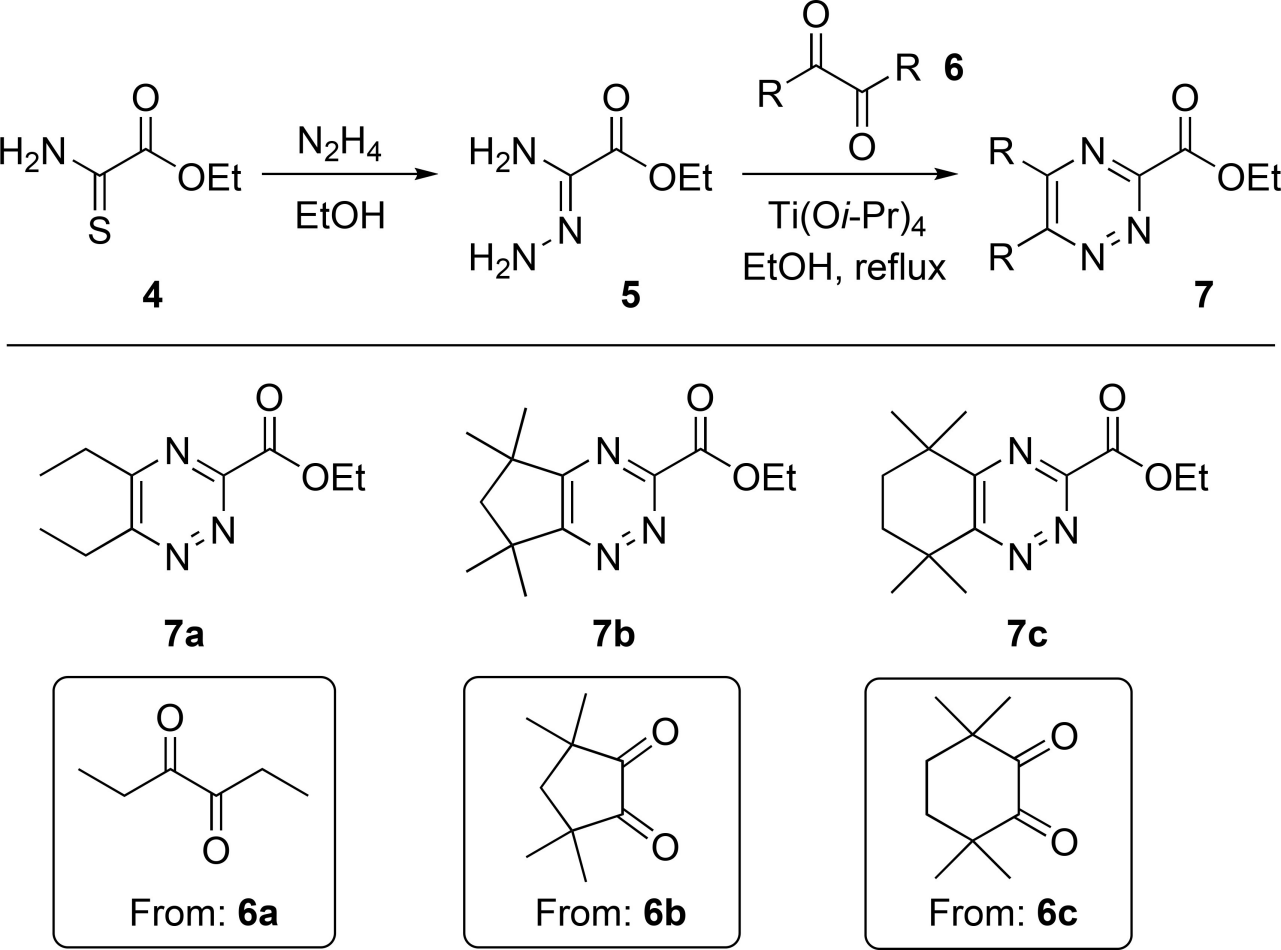
Synthesis of ethyl 1,2,4‐triazine‐3‐carboxylate building blocks **7 a**–**7 c**.

We commenced our studies with the synthesis of the requisite ethyl 1,2,4‐triazine‐3‐carboxylate building blocks. Ethyl thiooxamate **4** was first converted into the known amidrazone **5** using hydrazine hydrate following the literature procedure,^14^, ^17^ and used immediately in the next step to avoid potential self‐condensation of **5** during storage. Condensation reaction of **5** with 3,4‐hexanedione **6 a** in refluxing ethanol afforded **7 a** in 77 % yield (Scheme 1). Cyclic diketones **6 b** and **6 c** were also utilized as 1,2,4‐triazine ligands derived from these diketones are more resistant towards acid‐catalyzed hydrolysis and radiolysis in a process than those derived from acyclic diketones bearing hydrogen atoms in the alpha‐position.^[18]^ Diketones **6 b** and **6 c** proved somewhat less reactive than **6 a** in the condensation reactions with **5**. However, use of titanium(IV) isopropoxide as Lewis acid catalyst provided satisfactory results and **7 b** and **7 c** were obtained in reasonable yields of 61 % and 55 %, respectively (Scheme 1).

**Scheme 2:**
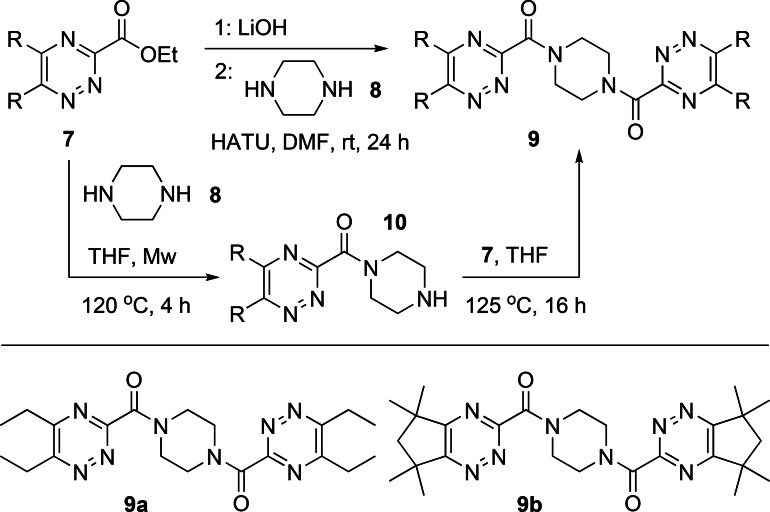
Synthesis of tetradentate ligands **9 a** and **9 b**.

With the building blocks **7 a**–**7 c** in hand, we next explored their reactions with different polyamine cores to synthesize multidentate triazine ligands,^[19]^ using **7 a** and piperazine to optimize the amidation step. Our initial approach was to convert esters **7** into the corresponding free acids by hydrolysis and explore amide coupling reactions with the polyamines. However, hydrolysis of **7 a** to the corresponding free acid resulted in partial decarboxylation, which precluded the use of amide coupling reagents (DCC, HATU, etc) to synthesize the ligands. We then explored direct amidation reactions of ester **7 a** with piperazine instead. Reaction of **7 a** with piperazine **8** in THF under microwave conditions generated the corresponding monoamide **10 a** in only 10 % yield, while attempted amidation reactions of **7 a** with **8** using lipase enzymes^14^, ^15^ resulted in no conversion to the product. However, we found that reaction of monoamide **10 a** with **7 a** in refluxing THF did generate the desired diamide **9 a**, albeit in low yield. To circumvent the low overall yield of this two‐step approach, we then returned to our original approach and converted **7 a** into the lithium salt of the acid, which was stable and did not undergo decarboxylation. Amide coupling (HATU) of the lithium salt of **7 a** with **8** generated the desired ligand **9 a** in 24 % yield (Scheme 2). In contrast to the low‐yielding reaction of **7 a** with piperazine **8**, the analogous reaction of **7 b** with **8** in THF under microwave conditions gave monoamide **10 b** in 83 % yield. Reaction of monoamide **10 b** with **7 b** in refluxing THF then gave ligand **9 b** in 37 % yield (Scheme 2).

**Scheme 3:**
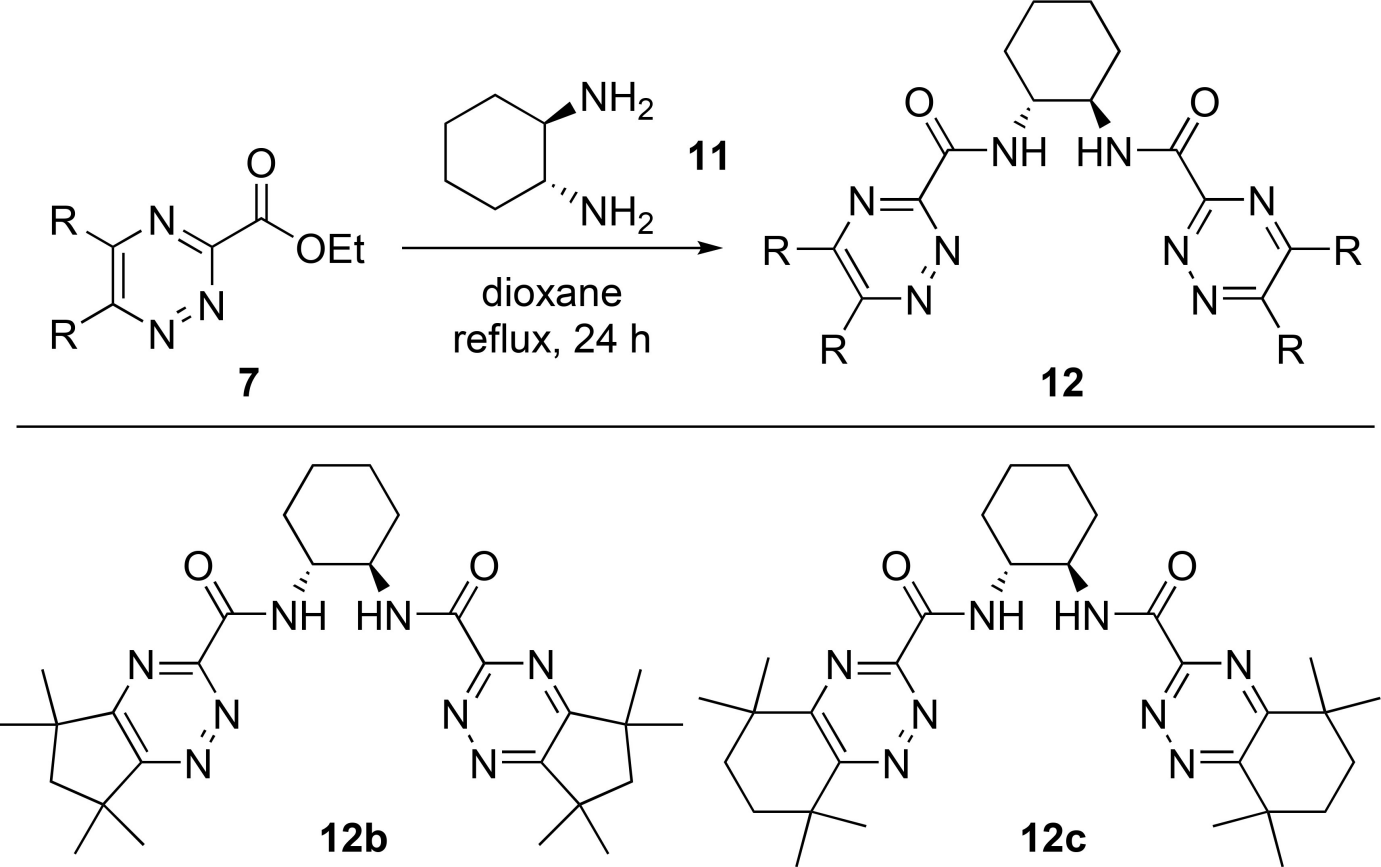
Synthesis of tetradentate ligands **12 b** and **12 c**.

We next explored potentially tetradentate triazine ligands **12** containing two bidentate 1,2,4‐triazine‐3‐carboxamide chelating groups derived from the pre‐organized diamine core (±)‐*trans*‐1,2‐diaminocyclohexane. Direct amidation reactions of esters **7 b** and **7 c** with (±)‐*trans*‐1,2‐diaminocyclohexane **11** in refluxing 1,4‐dioxane afforded the desired triazine ligands **12 b** and **12 c** in 54 % and 56 % yields, respectively (Scheme 3).

**Scheme 4:**
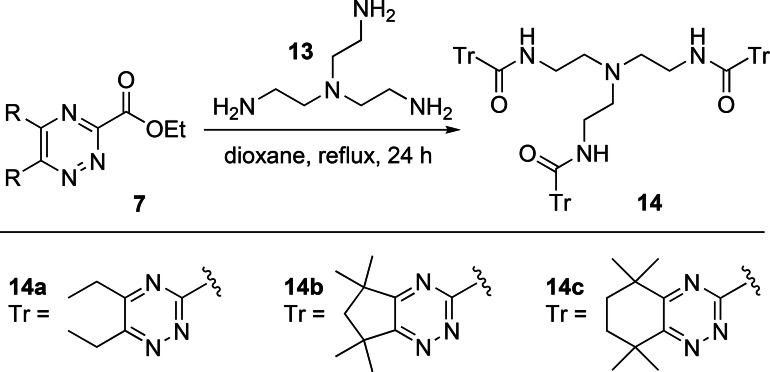
Synthesis of hexadentate ligands **14 a**–**14 c**.

Tris(2‐aminoethyl)amine (TREN) **13** is a widely used triamine core for the synthesis of hexadentate ligands containing bidentate catechol or hydroxypyridinone coordinating groups for use as sequestering agents for actinides^[16a]^ or as biostatic agents for chelation of Fe(III).^[20]^ We therefore explored the synthesis of analogous potentially hexadentate 1,2,4‐triazine ligands **14** containing three bidentate 1,2,4‐triazine‐3‐carboxamide chelating groups from reactions of **13** with ethyl 1,2,4‐triazine‐3‐carboxylate building blocks **7**. Amidation reactions of TREN **13** with each of the building blocks **7 a**–**7 c** in refluxing 1,4‐dioxane proceeded smoothly to form the target ligands **14 a**–**14 c**, albeit in low yields of ≤49 % (Scheme 4). A slightly lower yield of 41 % was obtained for ligand **14 a** under microwave conditions (THF, 2 h), whilst the use of lipase enzymes gave <20 % conversion of **14 a**, as judged by ^1^H NMR spectroscopy.^14^, ^15^

**Scheme 5:**
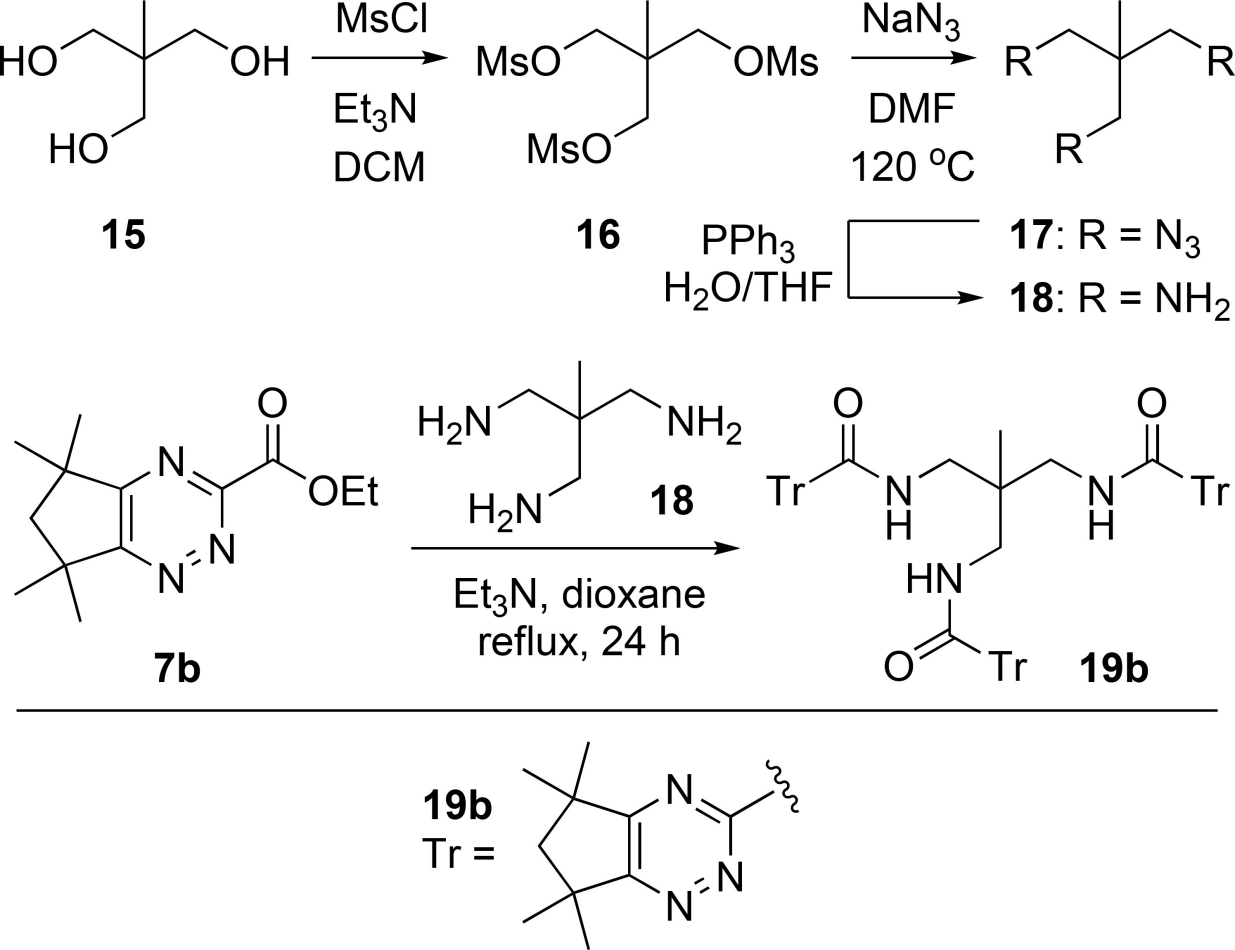
Synthesis of hexadentate ligand **19 b**.

The triamine tris(aminomethyl)ethane **18** is a frequently used core for the synthesis of tripodal ligands for coordination and sensing of different metal ions.^[21]^ Accordingly, we decided to employ **18** as a core for the synthesis of an analogous hexadentate 1,2,4‐triazine ligand **19 b** containing three bidentate 1,2,4‐triazine‐3‐carboxamide chelating groups. Triamine **18** was synthesized from commercially available triol **15** in three steps according to the literature procedures.^[22]^ Triol **15** was first converted into the trimesylate **16**, which was then treated with sodium azide in DMF to generate triazide **17**. Finally, Staudinger reduction^[23]^ of **17** using triphenylphosphine in the presence of water afforded triamine **18** in 31 % overall yield from **15**. Reaction of **18** with ethyl 1,2,4‐triazine‐3‐carboxylate building block **7 b** in refluxing 1,4‐dioxane gave the desired ligand **19 b** in 15 % yield (Scheme 5).

**Scheme 6:**
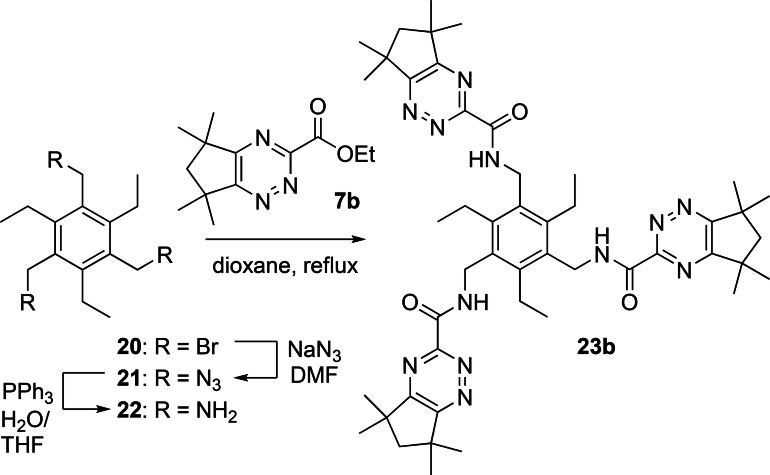
Synthesis of hexadentate ligand **23 b**.

Finally, we sought to synthesize a hexadentate 1,2,4‐triazine ligand with three bidentate 1,2,4‐triazine‐3‐carboxamide chelating groups containing the 1,3,5‐triethylbenzene core derived from triamine **22**, which has been utilized as a scaffold for many different ligands and receptors, including tripodal diglycolamide ligands for selective extraction of Am(III).^[24]^ Triamine **22** was synthesized from commercially available tribromide **20** in two steps and 52 % overall yield according to the literature procedures.^[25]^ Reaction of **20** with sodium azide afforded the crude triazide **21** which was reduced to triamine **22** using a Staudinger reduction. Reaction of **22** with the 1,2,4‐triazine building block **7 b** in refluxing 1,4‐dioxane gave the desired ligand **23 b** in 82 % yield (Scheme 6).

The physicochemical properties of the novel ligands **9 a**, **9 b**, **12 b**, **12 c**, **14 a**–**14 c**, **19 b** and **23 b** are presented in Table 1 and are compared to those of the benchmark 1,2,4‐triazine ligands **1**–**3** (Figure 1). As shown, the calculated partition coefficients (cLog*P* values) of the ligands span a wide range of values regardless of which method is used to calculate them. Whilst ligands **9 a**, **9 b**, **12 b**, **12 c**, **14 a** and **14 b** are all predicted to be less hydrophobic than ligands **1**–**3**, ligands **14 c**, **19 b** and **23 b** are predicted to be more hydrophobic than **1**–**3**. Ligand hydrophobicity has obvious implications for the solubility and extractability of metal complexes into the organic phase, and hence the distribution ratios of the metal ions that are obtained. However, as mentioned previously, making a ligand more hydrophobic (e. g.: by adding alkyl groups) is usually detrimental to the extraction kinetics. For the benchmark ligands **1**–**3**, the fraction of saturated carbons (Fsp^3^ value) ranges from 0.47 for ligand **3** to 0.59 for ligand **1**. For the novel ligands reported herein, all of them have higher Fsp^3^ values than ligands **1**–**3**, ranging from 0.6 for ligands **9 a** and **14 a** to 0.73 for ligand **12 c**. All the novel ligands except **9 a** and **9 b** contain hydrogen bond donor (HBD) amide NH groups which are absent in ligands **1**–**3**, whilst the number of hydrogen bond acceptor (HBA) groups in the novel ligands is comparable to (ligands **9 a**, **9 b**, **12 b**, **12 c**), or greater than (ligands **14 a**–**14 c**, **19 b**, **23 b**), those in ligands **1**–**3**.

**Table 1:** Physicochemical properties of the ligands.

Ligand	cLog*P* ^[a]^	cLog*P* ^[b]^	Fsp^3^	#HBD Groups	#HBA Groups
**1**	8.31±0.62	4.87	0.59	0	7
**2 a**	8.59±0.63	5.56	0.50	0	8
**3**	9.09±1.4	6.03	0.47	0	8
**9 a**	−0.25±0.71	1.6	0.60	0	8
**9 b**	4.31±0.84	2.86	0.69	0	8
**12 b**	6.49±0.75	3.61	0.71	2	8
**12 c**	7.62±0.75	4.1	0.73	2	8
**14 a**	1.43±0.77	2.15	0.60	3	13
**14 b**	8.27±0.95	3.85	0.69	3	13
**14 c**	9.96±0.95	4.66	0.71	3	13
**19 b**	8.71±0.93	4.35	0.68	3	12
**23 b**	11.8±0.92	6.49	0.63	3	12

^[a]^ Calculated from ChemSketch software. ^[b]^ Calculated from Swiss ADME, consensus cLog*P* values were used.

### Solvent Extraction Experiments

The novel ligands were then screened as potential selective extractants for the minor actinides Am(III) and Cm(III), as well as Eu(III) using solvent extraction experiments. Nitric acid solutions spiked with ^241^Am, ^244^Cm and ^152^Eu radionuclides were mixed with organic solutions of the ligands in the appropriate diluent (1‐octanol, cyclohexanone or nitrobenzene), and distribution ratios for each metal ion (*D*) were measured by α‐ or γ‐spectroscopy. Initial screening experiments were performed on ligands **12 b**, **12 c**, **14 b** and **14 c** using two nitric acid concentrations and 1‐octanol, cyclohexanone or nitrobenzene as diluent, and the results are shown in the Supporting Information (Table S1). With the exception of ligand **12 b** in cyclohexanone, all of the measured *D* values for Am(III) and Eu(III) were below 1 indicating that none of the novel ligands showed the ability to extract either metal ion from nitric acid solution. For ligand **12 b** in cyclohexanone, the *D* values for Am(III) and Eu(III) extraction from 1 M nitric acid were 1.03 and 1.07, respectively, indicating that **12 b** can co‐extract both elements from nitric acid, albeit with no selectivity between them (*SF*_Eu/Am_=1.04).

More detailed extraction experiments were then performed on ligands **9 a**, **14 a**, **19 b** and **23 b** across a range of nitric acid concentrations (see Supporting Information, Tables S2–S9). All of the *D* values for Am(III), Cm(III) and Eu(III) were below 1 and close to or below the detection limit, indicating that none of the novel ligands were able to extract Am(III), Cm(III) or Eu(III) from nitric acid solutions into 1‐octanol. The observed extraction results could be attributed to a number of reasons, as solvent extraction of metal ions into an organic phase is governed by several criteria, including the thermodynamic stability of the formed metal:ligand complex, the metal:ligand stoichiometry of the formed complex, its solubility/lipophilicity and the mechanism of extraction.^4^, ^26^

### NMR Titrations

In order to shed more light on the solvent extraction results, we next performed ^1^H NMR titrations of selected ligands with the diamagnetic lanthanides La(III) and Lu(III) as well as Y(III) in deuterated acetonitrile. In each case, 0.5 and 1 equivalents of metal nitrate solution were added to the free ligand to check for complex formation prior to performing the full titration. For ligands **9 b**, **12 b** and **12 c**, addition of 0.5 or 1 equivalents of La(III) or Y(III) gave NMR spectra that showed no new resonances for complex species (see Supporting Information, Figures S1–S7). In the case of **12 b** and **12 c**, a broadening of the free ligand resonances was observed on addition of either La(III) or Y(III), accompanied by a slight downfield shift in the amide N*H* protons from 8.36 ppm to ca. 9 ppm. This suggests complex species were not formed with these ligands.

Similar results were observed for ligand **14 b** which gave poorly resolved spectra in the titration with Y(III) (see Supporting Information, Figure S10). However, some minor new resonances did appear towards the end of the titration (singlets at 0.65, 1.17, 1.39 and 1.74 ppm). These could be due to a complex species but their relative amounts are very small, suggesting complex formation of **14 b** with Y(III) is minimal at best. Complex formation of the ligands with Y(III) could also be hampered by the relative kinetic inertness of Y(III) toward ligand substitution,^[27]^ which we observed previously in NMR studies of bis‐1,2,4‐triazine ligands.^[28]^ Some evidence for complex formation of **14 b** with La(III) was also observed. However, the new resonances were very broad and poorly resolved (see Supporting Information, Figure S9). New resonances appeared for the amide N*H* protons at 8.77 ppm and 9.44 ppm, and the region associated with the methylene protons adjacent to the nitrogen atoms between 2.5 ppm and 4 ppm was very complex. Although the results are inconclusive, this could be accounted for by the formation of a chiral complex in which the methylene protons of **14 b** are diastereotopic.

**Figure 2:**
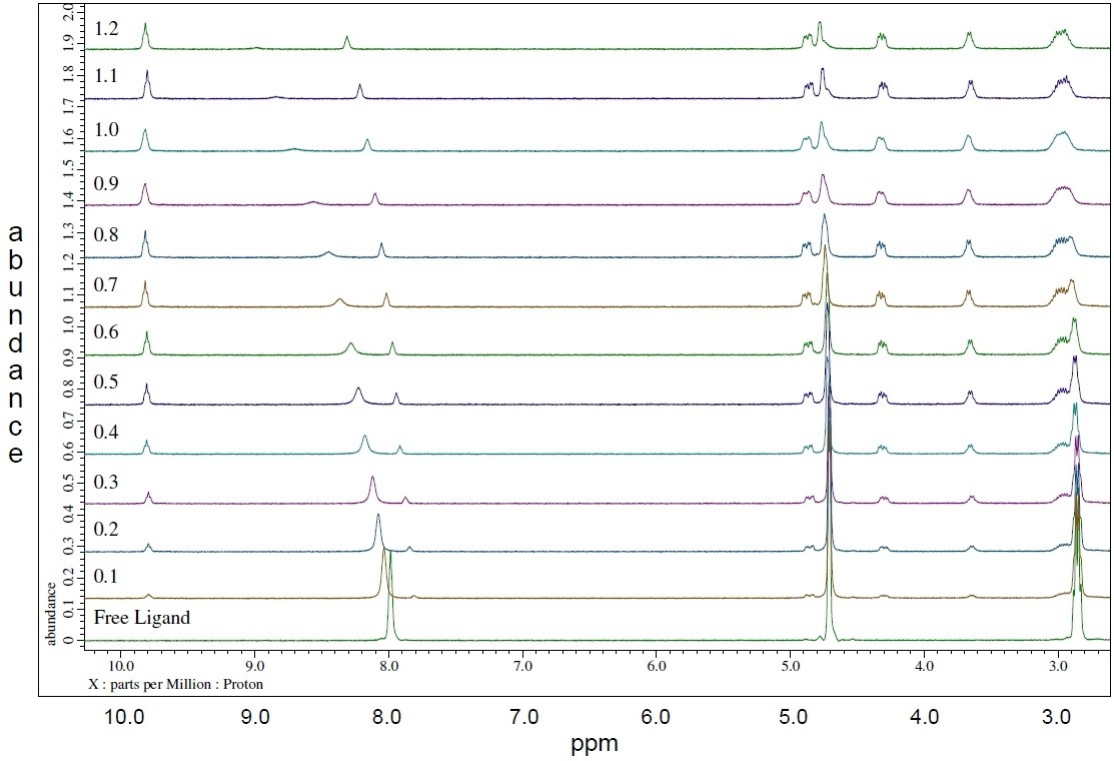
Stack plot for the ^1^H NMR titration of ligand **23 b** with La(NO_3_)_3_ in CD_3_CN. Region between 2.6 ppm and 10.2 ppm is shown for clarity. Bottom spectrum=free ligand. Each preceding spectrum corresponds to the addition of 0.1 equivalents of metal salt solution.

For ligand **23 b**, addition of 0.5 or 1 equivalents of La(III) to the free ligand gave NMR spectra that showed the disappearance of the free ligand resonances and the appearance of a new set of resonances associated with a complex species (see Supporting Information, Figures S12–S19). However, addition of either Lu(III) or Y(III) to solutions of **23 b** gave NMR spectra that showed no new resonances for complex species (see Supporting Information, Figures S21–S23). A full NMR titration of **23 b** with La(III) was therefore carried out. The free ligand resonances gradually disappeared and a new set of resonances appeared during the course of the NMR titration of **23 b** with La(III), suggesting a single complex species was formed with an overall metal:ligand stoichiometry of 1 : 1. Part of the stack plot for the titration of **23 b** with La(III) is presented in Figure 2, while the full stack plot and a species distribution curve are shown in the Supporting Information (Figures S24–S28).

During the NMR titration of **23 b** with La(III), we observed the appearance of two double‐doublets at 4.30 ppm and 4.85 ppm for the methylene protons adjacent to the amide N*H* protons, and a complex multiplet at 2.95 ppm for the methylene protons of the ethyl groups. This suggests these methylene groups are diastereotopic, indicating formation of a chiral complex between **23 b** and La(III). This could be accounted for by the formation of a chiral complex with a screw axis in which all three 1,2,4‐triazine‐3‐carboxamide groups of **23 b** coordinate to the metal in a bidentate fashion. However, other resonances are also present in addition to those expected for such a complex; an apparent doublet at 3.64 ppm, a broad singlet at 4.73 ppm and a broad singlet at 8.10 ppm (for amide N*H* protons). We tentatively propose that this is due to formation of a chiral complex in which two of the 1,2,4‐triazine‐3‐carboxamide groups of **23 b** coordinate to the metal in a bidentate fashion, while the third either does not coordinate, or coordinates in a different mode than the other two (e. g.: in a monodentate fashion). The lack of complex formation observed with **23 b** and the smaller Lu(III) ion suggests the size of the coordination cavity of **23 b** is better able to accommodate larger lanthanides such as La(III) but not smaller ones such as Lu(III).

### DFT Calculations

DFT calculations were then performed to gain an insight into the variation in complexation behaviour among the ligands. Specifically, calculation of the binding energies of ligands **9 b**, **12 b**, **14 b**, and **23 b** with La(III) in acetonitrile was carried out to provide a comparison with the NMR titration results, which showed variation in complexation behaviour between the novel ligands. The energetics of complexation of **1** were also determined as a benchmark, since BTP ligands are reported to bind La(III) in non‐aqueous environments.^[29]^

**Figure 3:**
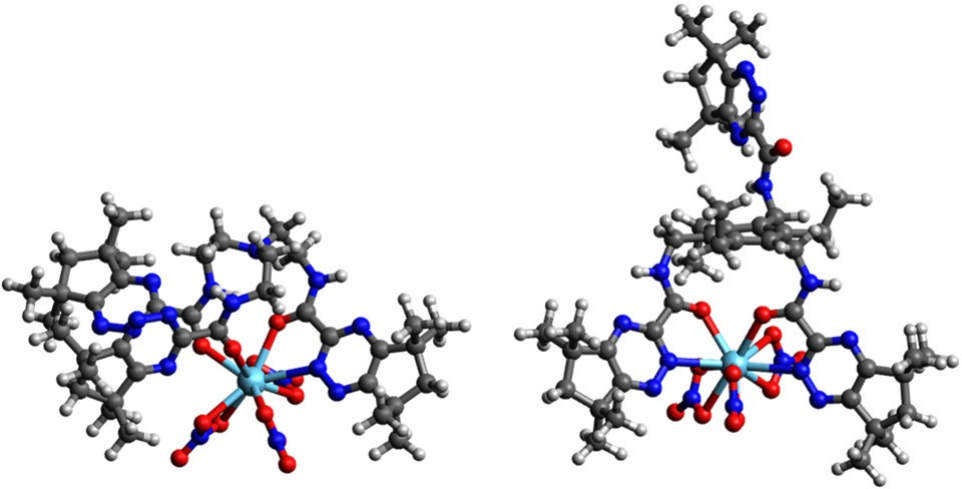
DFT optimized geometries of the La(III) complexes of ligands **14 b** (left) and **23 b** (right).

The most favourable complexes of **9 b**, **12 b**, **14 b**, and **23 b** were all calculated to be of the form La(κ^4^‐L)(NO_3_)_3_ in contrast with **1**, which was calculated to be La(κ^3^‐L)(NO_3_)_3_(H_2_O)_2_. Images of the complexes and full geometries are given in the Supporting Information (Figures S33–S35). Total Gibbs free energies of binding, Δ*G*, are given in Table 2, showing a negative binding energy was calculated for **1**, consistent with its ability to bind La(III) mentioned above. Ligands **14 b** and **23 b** show negative and low positive binding energies, respectively, consistent with the NMR titrations suggesting complexation with lanthanides does occur. Furthermore, the calculated complexes of **14 b** and **23 b** are of very low symmetry, which is consistent with the experimental indications of chirality. Interestingly, the most favourable complex of **14 b** shows only one of the three 1,2,4‐triazine‐3‐carboxamide units chelating to La(III) in a bidentate fashion, with the other two coordinating only *via* the *O*‐atom of the carboxamide group (Figure 3). The most favourable complex of **23 b** with La(III) shows that two of the three 1,2,4‐triazine‐3‐carboxamide units are chelating to the metal, with the third remaining unbound on the opposite side of the central benzene core (Figure 3). This structure is broadly consistent with the observed NMR titration results for **23 b**. Ligands **9 b** and **12 b** are calculated to have relatively large positive binding energies, again consistent with the NMR titrations, in which complexation was not observed.

**Table 2:** Calculated thermodynamic quantities of the complexes of ligands **1**, **9 b**, **12 b**, **14 b** and **23 b** with La(III).

Ligand	Δ*G* (kJ mol^−1^)^[a]^	Δ*H* (kJ mol^−1^)^[a]^	−*T*Δ*S* (kJ mol^−1^)^[a]^	*E_reorg_* (kJ mol^−1^)	*E_int_* (kJ mol^−1^)
**1**	−9.8	34.1	−43.9	7.8	−234.0
**9 b**	56.1	186.3	−130.3	68.4	−287.8
**12 b**	53.9	193.2	−139.3	83.9	−294.1
**14 b**	−11.3	116.9	−128.2	59.4	−362.6
**23 b**	4.5	140.2	−135.7	64.3	−336.6

^[a]^ Calculated at 298.15 K.

Encouraged by the consistency of the calculations with the experimental NMR results, further analysis was carried out to gain insights into the contributing factors to the binding energies. The enthalpic and entropic contributions to the binding are also listed in Table 2 and show significant variation between the ligands. All Δ*H* values are positive, indicative of stronger interactions between the water ligands and La(III) than between the synthesised ligands and La(III), but all have negative −*T*Δ*S* terms, consistent with the release of water molecules on complexation of the ligands. The values of Δ*H* and −*T*Δ*S* for **1** are significantly smaller than all the other ligands, consistent with its lower denticity and the smaller number of water molecules displaced.

A comparison of the novel ligands shows that **9 b** and **12 b** have very similar calculated contributions to their binding energies. The main difference between these ligands and ligands **14 b** and **23 b**, which showed some evidence of metal binding experimentally, is the enthalpy of binding rather than the entropic contribution. A further breakdown was calculated by determining the reorganisation energies, *E_reorg_*, of the ligands (the energy differences between the ligands in their free and bound conformations), and the interaction energies, *E_int_*, of the ligands (the energy difference between each ligand complex and the isolated ligand and remaining atoms of the complex, all in their complexed geometries), which are listed in Table 2. These results show that the trends in enthalpy of binding are largely consistent with both the reorganisation energies and interaction energies. Ligands **14 b** and **23 b** are calculated to have lower *E_reorg_* and *E_int_* values than **9 b** and **12 b**, indicating that their lack of binding is likely to arise from a combination of these factors. These results provide an indication that such calculations may be able to assist in the design of further novel ligands in future.

## Conclusions

We report the application of a new approach to the design of ligands for selective actinide extraction which is inspired by the field of drug discovery. A series of nine novel ligands were synthesized from readily available ethyl 1,2,4‐triazine‐3‐carboxylate building blocks and evaluated as potential selective extracting agents for the minor actinides Am(III) and Cm(III). One of the ligands is able to co‐extract Am(III) and Eu(III) from nitric acid into cyclohexanone, albeit with no selectivity between the two metals. Ligand **23 b** formed a 1 : 1 complex with the larger La(III) ion but not with the smaller Lu(III) or Y(III) ions, suggesting a size‐based sensitivity of the ligands’ coordination cavity. DFT calculations revealed that the two ligands that showed experimental evidence of complexation of La(III) by NMR had either low or negative Gibbs free energies of metal binding. We conclude that, whilst complex formation with Am(III) and Cm(III) may be possible with some of the ligands, the resulting complexes are not sufficiently hydrophobic to be extracted into the organic phase. Further exploration of this ligand design approach is underway with other multidentate *N*‐donor building blocks containing the crucial 1,2,4‐triazine ring for selective extraction of the minor actinides in nuclear fuel reprocessing.

## Experimental Section

### General Procedures

All solvents and reagents were purchased from Sigma‐Aldrich, Acros Organics, Fluorochem or Alfa‐Aesar and used without further purification unless otherwise specified. Reactions were monitored by TLC using silica gel with UV_254_ fluorescent indicator. NMR spectra were recorded on a JEOL ECS400FT Delta spectrometer (399.78 MHz for ^1^H NMR, 100.53 MHz for ^13^C NMR). Chemical shifts are reported in parts per million (ppm) relative to tetramethylsilane as internal standard. Coupling constants (*J*) are measured in hertz. Multiplets are reported as follows: b=broad, s=singlet, d=doublet, dd=double doublet, dt=double triplet, t=triplet, q=quartet, qu=quintet, m=multiplet, app d=apparent doublet, app t=apparent triplet. High resolution mass spectra were obtained on a Finnigan MAT900XLT high‐resolution double focussing MS spectrometer using nano‐electrospray ionisation (NESI) at the EPSRC UK National Mass Spectrometry Service (University of Swansea). Column chromatography was conducted using 0.060–0.20 mm silica gel (70–230 mesh), and automated flash column chromatography was performed using a Biotage Isolera One ISO‐1SV instrument. The calculated log*P* (cLog*P*) values of the ligands were calculated using ChemSketch software (available at https://www.acdlabs.com/) and SwissADME (available at http://www.swissadme.ch/). The fractions of saturated carbon atoms (Fsp^3^ values) of the ligands were calculated as the ratio of the number of saturated sp^3^ hybridized carbon atoms to the total number of carbon atoms for each ligand. Diketones **6 b** ^[8]^ and **6 c** ^[6c]^ were prepared as described previously.

### Amidrazone 5^[17]^

Ethyl thiooxamate **4** (2.03 g, 15.24367 mmol) was dissolved in ethanol (50 mL), and a solution of hydrazine hydrate (0.95 mL, 50 %, 1 equiv) in ethanol (10 mL) was added dropwise over 10 minutes. The reaction mixture was stirred at room temperature for 3 hours. The solvent was evaporated under reduced pressure and the solid was triturated with ethanol (100 mL). The insoluble solid was filtered and washed with ethanol (40 mL) and the filtrate was evaporated under reduced pressure to afford amidrazone **5** as an orange solid (1.67 g, 84 %). ^1^H NMR (399.8 MHz, CDCl_3_, Me_4_Si): δ=1.34 (t, *J*=7.2 Hz, 3H, C*H*_3_), 4.31 (q, *J*=7.2 Hz, 2H, C*H*_2_), 4.55 (br s, 2H, N*H*_2_), 4.81 (br s, 2H, N*H*_2_) ppm. ^13^C NMR (100.5 MHz, CDCl_3_, Me_4_Si): δ=14.2 (*C*H_3_), 62.4 (*C*H_2_), 140.3 (*C*=N), 162.0 (*C*=O) ppm.

### Synthesis of Esters 7. General Procedure A

Amidrazone **5** was dissolved in ethanol (50 mL per g of **2**) and the appropriate diketone **6** (1–1.1 equivalents) was added. The reaction mixture was stirred at room temperature for 1 hour and then heated under reflux for 24 hours. The flask was allowed to cool to room temperature and the solvent was evaporated. The residue was purified by flash column chromatography on silica gel, eluting with hexane/EtOAc (60 : 40) to afford the pure ester **7**.

### Synthesis of Esters 7. General Procedure B

Amidrazone **5** was dissolved in ethanol (50 mL per g of **2**) and the appropriate diketone **6** (1–1.1 equivalents) was added, followed by titanium(IV) isopropoxide (1.5 equivalents). The reaction mixture was stirred at room temperature for 2.5 hours and then heated under reflux for 24 hours. The flask was allowed to cool to room temperature and 0.5 M HCl (40 mL) was added followed by water (40 mL). The formed precipitate was filtered off and washed with ethyl acetate (20 mL). The filtrate was extracted with ethyl acetate (4×50 mL) and the combined organic extracts were washed with brine (2×30 mL), dried over MgSO_4_ and concentrated to afford the crude product as a brown oil. The crude product was purified by flash column chromatography on silica gel, eluting with petroleum ether/EtOAc (70 : 30) to afford the pure ester **7**.

### Ethyl 5,6‐diethyl‐1,2,4‐triazine‐3‐carboxylate (7 a)

Obtained from amidrazone **5** (1.98 g, 15.1 mmol) and 3,4‐hexanedione **6 a** (1.89 g, 16.6 mmol, 1.1 equivalents) as a yellow oil (2.42 g, 77 %) following general procedure A. ^1^H NMR (399.8 MHz, CDCl_3_, Me_4_Si): δ=1.35 (t, *J*=7.2 Hz, 3H, C*H*_3_), 1.40 (t, *J*=7.2 Hz, 3H, C*H*_3_), 1.45 (t, *J*=7.2 Hz, 3H, C*H*_3_), 2.91 (q, *J*=7.2 Hz, 2H, CH_2_), 3.07 (q, *J*=7.2 Hz, 2H, CH_2_), 4.53 (q, *J*=7.2 Hz, 2H, CH_2_) ppm. ^13^C NMR (100.5 MHz, CDCl_3_, Me_4_Si): δ=11.5 (*C*H_3_), 12.2 (*C*H_3_), 14.2 (*C*H_3_), 26.0 (*C*H_2_), 27.2 (*C*H_2_), 62.9 (*C*H_2_), 155.4 (quat), 162.8 (quat), 163.2 (quat), 163.3 (quat) ppm. HRMS (NESI): calcd. for C_10_H_15_N_3_O_2_ [M + H]^+^ 210.1243; found 210.1237.

### Ethyl 5,5,7,7‐tetramethyl‐6,7‐dihydro‐5*H*‐cyclopenta[*e*]‐1,2,4‐triazine‐3‐carboxylate (7 b)

Obtained from amidrazone **5** (1.06 g, 8.11 mmol) and 3,3,5,5‐tetramethylcyclopentane‐1,2‐dione **6 b** (1.25 g, 8.11 mmol, 1 equivalent) as a yellow solid (1.24 g, 61 %) following general procedure B. ^1^H NMR (399.8 MHz, CDCl_3_, Me_4_Si): δ=1.41 (s, 6H, 2×C*H*_3_), 1.45 (t, *J*=7.2 Hz, 3H, C*H*_3_), 1.48 (s, 6H, 2×C*H*_3_), 2.02 (s, 2H, C*H*_2_), 4.54 (q, *J*=7.2 Hz, 2H, C*H*_2_O) ppm. ^13^C NMR (100.5 MHz, CDCl_3_, Me_4_Si): δ=14.3 (*C*H_3_), 28.9 (2×*C*H_3_), 29.6 (2×*C*H_3_), 40.7 (quat), 41.4 (quat), 52.7 (*C*H_2_), 63.0 (*C*H_2_O), 156.7 (quat), 163.4 (quat), 170.0 (quat), 171.5 (quat) ppm. HRMS (NESI): calcd. for C_13_H_19_N_3_O_2_ [M+H]^+^ 250.1550; found 250.1552.

### Ethyl 5,5,8,8‐tetramethyl‐5,6,7,8‐tetrahydro‐1,2,4‐benzotriazine‐3‐carboxylate (7 c)

Obtained from amidrazone **5** (0.795 g, 6.07 mmol) and 3,3,6,6‐tetramethylcyclohexane‐1,2‐dione **6 c** (1.019 g, 6.07 mmol, 1 equivalent) as a yellow solid (0.877 g, 55 %) following general procedure B. ^1^H NMR (399.8 MHz, CDCl_3_, Me_4_Si): δ=1.37 (s, 6H, 2×C*H*_3_), 1.45 (t, *J*=7.2 Hz, 3H, C*H*_3_), 1.46 (s, 6H, 2×C*H*_3_), 1.82 (s, 4H, 2×C*H*_2_), 4.52 (q, *J*=7.2 Hz, 2H, C*H*_2_O) ppm. ^13^C NMR (100.5 MHz, CDCl_3_, Me_4_Si): δ=14.3 (CH3), 29.2 (2×*C*H_3_), 29.8 (2×*C*H_3_), 33.3 (*C*H_2_), 33.6 (*C*H_2_), 37.0 (quat), 37.5 (quat), 62.8 (*C*H_2_O), 155.3 (quat), 163.4 (quat), 165.5 (quat), 165.7 (quat) ppm. HRMS (NESI): calcd. for C_14_H_21_N_3_O_2_ [M+H]^+^ 264.1707; found 264.1710.

### Compound (9 a)

1 M LiOH (2.08 mL, 2.08 mmol) was added to a solution of ester **7 a** (0.396 g, 1.89 mmol) in THF (8 mL) and the reaction mixture was stirred at room temperature for 30 minutes. The volatiles were removed under reduced pressure and the residue was lyophilized. The solid was dissolved in dry DMF (15 mL) and HATU (0.791 g, 2.08 mmol) and piperazine **8** (0.081 g, 0.945 mmol) were sequentially added. The solution was stirred at room temperature for 24 hours. DMF was removed under reduced pressure, the residue was suspended in water (30 mL) and extracted with ethyl acetate (3×20 mL). The combined organic extracts were washed with water (3×30 mL), brine (30 mL) and dried over MgSO_4_. The solvent was removed under reduced pressure and the residue was purified by flash column chromatography on silica gel, eluting with DCM/acetone (1 : 1) to afford the desired compound **9 a** as an off‐white solid (0.094 g, 24 %). ^1^H NMR (399.8 MHz, CDCl_3_, Me_4_Si): δ=1.29–1.44 (m, 12H, 4×C*H*_3_CH_2_), 2.83–2.92 (m, 4H, 2×CH_3_C*H*_2_), 2.98–3.08 (m, 4H, 2×CH_3_C*H*_2_), 3.54 (s, 2H, C*H*_2_N), 3.59–3.62 (m, 2H, C*H*_2_N), 3.91–3.93 (m, 2H, C*H*_2_N), 4.01 (s, 2H, C*H*_2_N) ppm. ^13^C NMR (100.5 MHz, CDCl_3_, Me_4_Si): δ=10.9 (2×*C*H_3_CH_2_), 12.1 (2×*C*H_3_CH_2_), 25.9 (2×CH_3_ *C*H_2_), 26.0 (2×CH_3_ *C*H_2_), 27.0 (2×CH_3_ *C*H_2_), 27.1 (2×CH_3_ *C*H_2_), 41.9 (*C*H_2_N), 42.4 (*C*H_2_N), 46.8 (*C*H_2_N), 47.4 (*C*H_2_N), 159.0 (quat), 161.6 (quat), 161.7 (quat), 163.6 (quat), 164.0 (quat), 164.2 (quat) ppm. HRMS (NESI): calcd. for C_20_H_29_N_8_O_2_ [M+H]^+^ 413.2408; found 413.2405.

### Compound (10 b)

A solution of ester **7 b** (0.173 g, 0.694 mmol) and piperazine **8** (0.299 g, 3.47 mmol) in THF (1 mL) was subjected to microwave irradiation at 120 °C for 4 hours. The solvent was removed under reduced pressure and the residue was purified by flash column chromatography on silica gel, eluting with DCM/methanol (95 : 5) to afford the monoamide **10 b** as a light‐yellow solid (0.166 g, 83 %). ^1^H NMR (399.8 MHz, CDCl_3_, Me_4_Si): δ=1.39 (s, 6H, 2×CH3), 1.48 (s, 6H, 2×CH3), 2.01 (s, 2H, CH2), 2.90 (app t, *J*=3.9 Hz, 2H, C*H*_2_N), 3.01 (app t, *J*=3.9 Hz, 2H, C*H*_2_N), 3.37 (app t, *J*=3.9 Hz, 2H, C*H*_2_N), 3.84 (app t, *J*=3.9 Hz, 2H, C*H*_2_N) ppm. ^13^C NMR (100.5 MHz, CDCl_3_, Me_4_Si): δ=29.0 (*C*H_3_), 29.7 (*C*H_3_), 40.8 (quat), 41.5 (quat), 43.3 (*C*H_2_), 45.8 (*C*H_2_N), 46.3 (*C*H_2_N), 48.4 (*C*H_2_N), 52.6 (*C*H_2_N), 160.6 (quat), 164.2 (quat), 168.6 (quat), 171.5 (quat) ppm. HRMS (NESI): calcd. for C_15_H_24_N_5_O [M+H]^+^ 290.1975; found 290.1980.

### Compound (9 b)

A solution of monoamide **10 b** (0.138 g, 0.477 mmol) and ester **7 b** (0.178 g, 0.715 mmol) in THF (3 mL) was slowly concentrated at 125 °C and left at this temperature for 16 hours. The residue was suspended in a mixture of methanol/DCM (1 : 9) and the insoluble solid was filtered off. The filtrate was evaporated and the residue was purified by flash column chromatography on silica gel, eluting with ethyl acetate/methanol (100:0→80 : 20) to afford the desired compound **9 b** as a white solid (0.087 g, 37 %). ^1^H NMR (399.8 MHz, CDCl_3_, Me_4_Si): δ=1.39 (s, 6H, 2×C*H*_3_), 1.42 (s, 6H, 2×C*H*_3_), 1.47 (s, 6H, 2×C*H*_3_), 1.52 (s, 6H, 2×C*H*_3_), 2.01 (s, 2H, C*H*_2_), 2.05 (s, 2H, C*H*_2_), 3.54 (s, 2H, C*H*_2_N), 3.61–3.64 (m, 2H, C*H*_2_N), 3.95–3.98 (m, 2H, C*H*_2_N), 4.05 (s, 2H, C*H*_2_N) ppm. ^13^C NMR (100.5 MHz, CDCl_3_, Me_4_Si): δ=29.1 (4×*C*H_3_), 29.7 (2×*C*H_3_), 29.8 (2×*C*H_3_), 40.8 (2×quat), 40.9 (quat), 41.6 (quat), 42.0 (*C*H_2_), 42.5 (*C*H_2_), 46.9 (*C*H_2_N), 47.4 (*C*H_2_N), 52.6 (2×*C*H_2_N), 160.1 (quat), 164.4 (quat), 168.9 (quat), 169.1 (quat), 171.8 (2×quat) ppm. HRMS (NESI): calcd. for C_26_H_37_N_8_O_2_ [M+H]^+^ 493.3039; found 493.3037.

### Synthesis of Ligands 12, 14, 19 b and 23 b. General Procedure

The appropriate amine **11**, **13**, **18** or **22** was added to a solution of the appropriate ester **7** in 1,4‐dioxane (5 mL per g of **7**) and the reaction mixture was heated under reflux for 24 hours. The solvent was evaporated under reduced pressure and the crude product was purified by flash column chromatography to afford the pure ligand **14**, **19 b** or **23 b**. Ligands **12 b** and **12 c** were obtained as pure samples by triturating the crude products with diethyl ether and filtering the insoluble ligands, rather than by chromatography.

#### Compound (12 b)

Obtained from (±)‐*trans*‐1,2‐diaminocyclohexane **11** (0.114 g, 1.06 mmol) and ester **7 b** (0.584 g, 2.34 mmol, 2.2 equivalents) as a yellow solid (0.296 g, 54 %). ^1^H NMR (399.8 MHz, CDCl_3_, Me_4_Si): δ=1.35 (s, 6H, 2×C*H*_3_), 1.39 (s, 6H, 2×C*H*_3_), 1.44 (s, 6H, 2×C*H*_3_), 1.46 (s, 6H, 2×C*H*_3_), 1.84–1.85 (m, 2H, C*H*_2_), 1.99 (s, 4H, 2×C*H*_2_), 2.21–2.24 (m, 2H, C*H*_2_), 4.09–4.18 (m, 2H, 2×C*H*NH), 8.27 (d, *J*=7.3 Hz, 2H, 2×N*H*) ppm. ^13^C NMR (100.5 MHz, CDCl_3_, Me_4_Si): δ=24.8 (2×*C*H_2_), 29.0 (2×*C*H_3_), 29.1 (2×*C*H_3_), 29.7 (2×*C*H_3_), 29.8 (2×*C*H_3_), 32.6 (2×*C*H_2_), 40.7 (2×quat), 41.4 (2×quat), 52.9 (2×*C*H_2_), 53.8 (2×*C*H), 156.7 (2×quat), 161.8 (2×quat), 169.8 (2×quat), 171.9 (2×quat) ppm. HRMS (NESI): calcd. for C_28_H_40_N_8_O_2_ [M + H]^+^ 521.3347; found 521.3338.

#### Compound (12 c)

Obtained from (±)‐*trans*‐1,2‐diaminocyclohexane **11** (0.044 g, 0.409 mmol) and ester **7 c** (0.237 g, 0.90 mmol, 2.2 equivalents) as a yellow solid (0.126 g, 56 %). ^1^H NMR (399.8 MHz, CDCl_3_, Me_4_Si): δ=1.35 (s, 6H, 2×C*H*_3_), 1.37 (s, 6H, 2×C*H*_3_), 1.42 (s, 12H, 4×C*H*_3_), 1.42–1.48 (m, 4H, 2×C*H*_2_), 1.77 (s, 8H, 4×C*H*_2_), 1.77–1.83 (m, 2H, C*H*_2_), 2.24 (br s, 2H, C*H*_2_), 4.09 (br s, 2H, 2×C*H*NH), 8.28 (d, *J*=6.9 Hz, 2H, 2×N*H*) ppm. ^13^C NMR (100.5 MHz, CDCl_3_, Me_4_Si): δ=24.8 (2×*C*H_2_), 29.2 (2×*C*H_3_), 29.3 (2×*C*H_3_), 29.8 (2×*C*H_3_), 29.9 (2×*C*H_3_), 32.4 (2×*C*H_2_), 33.3 (2×*C*H_2_), 33.6 (2×*C*H_2_), 36.9 (2×quat), 37.5 (2×quat), 54.0 (2×*C*H), 154.9 (2×quat), 161.9 (2×quat), 165.3 (2×quat), 165.4 (2×quat) ppm. HRMS (NESI): calcd. for C_30_H_44_N_8_O_2_ [M+H]^+^ 549.3660; found 549.3646.

#### Compound (14 a)

Obtained from tris(2‐aminoethyl)amine **13** (0.058 g, 0.396 mmol) and ester **7 a** (0.265 g, 1.27 mmol, 3.2 equivalents) as a light yellow solid (0.054 g, 21 %). DCM/MeOH (95 : 5) was used as the chromatography eluent. ^1^H NMR (399.8 MHz, CDCl_3_, Me_4_Si): δ=1.29 (t, *J*=7.2 Hz, 9H, 3×C*H*_3_), 1.32 (t, *J*=7.2 Hz, 9H, 3×C*H*_3_), 2.88 (q, *J*=7.2 Hz, 6H, 3×C*H*_2_), 2.89 (t, *J*=6.0 Hz, 6H, 3×C*H*_2_N), 2.96 (q, *J*=7.2 Hz, 6H, 3×C*H*_2_), 3.61 (q, *J*=6.0 Hz, 6H, 3×C*H*_2_NH), 8.51 (t, *J*=6.0 Hz, 3H, 3×N*H*) ppm. ^13^C NMR (100.5 MHz, CDCl_3_, Me_4_Si): δ=11.5 (3×*C*H_3_), 12.1 (3×*C*H_3_), 25.8 (3×*C*H_2_), 27.2 (3×*C*H_2_), 38.1 (3×*C*H_2_N), 53.6 (3×*C*H_2_NH), 155.7 (3×quat), 161.9 (3×quat), 162.1 (3×quat), 163.4 (3×quat) ppm. HRMS (NESI): calcd. for C_30_H_45_N_13_O_3_ [M+H]^+^ 636.3841; found 636.3829.

#### Compound (14 b)

Obtained from tris(2‐aminoethyl)amine **13** (0.036 g, 0.248 mmol) and ester **7 b** (0.204 g, 0.818 mmol, 3.3 equivalents) as a yellow solid (0.077 g, 41 %). EtOAc/MeOH (95 : 5) was used as the chromatography eluent. ^1^H NMR (399.8 MHz, CDCl_3_, Me_4_Si): δ=1.40 (s, 18H, 6×C*H*_3_), 1.41 (s, 18H, 6×C*H*_3_), 1.99 (s, 6H, 3×C*H*_2_), 2.91 (t, *J*=6.4 Hz, 6H, 3×C*H*_2_N), 3.63 (t, *J*=6.4 Hz, 6H, 3×C*H*_2_NH), 8.54 (t, *J*=6.4 Hz, 3H, 3×N*H*) ppm. ^13^C NMR (100.5 MHz, CDCl_3_, Me_4_Si): δ=28.9 (6×*C*H_3_). 29.7 (6×*C*H_3_), 38.3 (3×*C*H_2_N), 40.5 (3×quat), 41.4 (3×quat), 52.8 (3×*C*H_2_), 53.8 (3×*C*H_2_NH), 157.0 (3×quat), 162.0 (3×quat), 169.6 (3×quat), 171.9 (3×quat) ppm. HRMS (NESI): calcd. for C_39_H_57_N_13_O_3_ [M + H]^+^ 756.4780; found 756.4774.

#### Compound (14 c)

Obtained from tris(2‐aminoethyl)amine **13** (0.039 g, 0.269 mmol) and ester **7 c** (0.234 g, 0.889 mmol, 3.3 equivalents) as a yellow solid (0.105 g, 49 %). EtOAc/MeOH (95 : 5) was used as the chromatography eluent. ^1^H NMR (399.8 MHz, CDCl_3_, Me_4_Si): δ=1.36 (s, 36H, 12×C*H*_3_), 1.79 (s, 12H, 6×C*H*_2_), 2.90 (t, *J*=6.4 Hz, 6H, 3×C*H*_2_N), 3.62 (q, *J*=6.4 Hz, 6H, 3×C*H*_2_NH), 8.50 (t, *J*=6.4 Hz, 3H, 3×N*H*) ppm. ^13^C NMR (100.5 MHz, CDCl_3_, Me_4_Si): δ=29.2 (6×*C*H_3_), 29.8 (6×*C*H_3_), 33.2 (3×*C*H_2_), 33.5 (3×*C*H_2_), 36.8 (3×quat), 37.5 (3×quat), 38.2 (3×*C*H_2_N), 53.8 (3×*C*H_2_NH), 155.4 (3×quat), 162.0 (3×quat), 165.2 (3×quat), 165.6 (3×quat) ppm. HRMS (NESI): calcd. for C_42_H_63_N_13_O_3_ [M+H]^+^ 798.5250; found 798.5241.

#### Compound (16)^[22]^

Methanesulfonyl chloride (1.71 g, 1.16 mL, 15.0 mmol) was added dropwise to a suspension of 1,1,1‐tris(hydroxymethyl)ethane **15** (0.545 g, 4.54 mmol) and triethylamine (1.52 g, 2.08 mL, 15.0 mmol) in DCM (20 mL) at 0 °C. The reaction mixture was allowed to stir at 0 °C for 3 hours. Cold water (20 mL) and DCM (15 mL) were sequentially added. The phases were separated and the organic phase was washed sequentially with 0.5 M HCl (20 mL), water (20 mL) and brine (20 mL). The organic phase was dried over MgSO_4,_ and the solvent was evaporated under reduced pressure to give the intermediate tris‐methanesulfonate ester **16** (1.44 g) as a beige solid which was used in the next step without further purification. ^1^H NMR (399.8 MHz, CDCl_3_, Me_4_Si): δ=1.15 (s, 3H, C*H*_3_), 3.06 (s, 9H, 3×CH_2_OSO_2_C*H*_3_), 4.14 (s, 6H, 3×C*H*_2_OSO_2_CH_3_) ppm. ^13^C NMR (100.5 MHz, CDCl_3_, Me_4_Si): δ=16.4 (*C*H_3_), 37.5 (3×CH_2_OSO_2_ *C*H_3_), 39.6 (quat), 69.3 (3×*C*H_2_OSO_2_CH_3_) ppm.

#### Compound (17)^[22]^

The intermediate tris‐methanesulfonate ester **16** (1.44 g) was dissolved in DMF (25 mL) and NaN_3_ (1.48 g, 22.7 mmol) was added. The solution was heated at 120 °C for 8 hours and the flask was allowed to cool to room temperature and stirring was continued at room temperature overnight. Water (100 mL) was added and the mixture was extracted with diethyl ether (3×50 mL). The combined organic extracts were washed with water (5×25 mL) and brine (25 mL), and then dried over MgSO_4_ and concentrated to approximately 25 % of the original volume to give a solution of the intermediate tris‐azide **17** which was used in the next step without further purification. ^1^H NMR (399.8 MHz, CDCl_3_, Me_4_Si): δ=0.97 (s, 3H, C*H*_3_), 3.25 (s, 6H, 3×C*H*_2_N_3_) ppm.

#### Compound (18)^[22]^

To the above solution of the tris‐azide **17** in diethyl ether was added THF (10 mL) and then water (2.7 mL, 2.7 g, 150 mmol). PPh_3_ (3.93 g, 15.0 mmol) was added in small portions over 15 minutes to the vigorously stirred reaction mixture at room temperature and then stirring was continued for a further 15 minutes. The reaction mixture was then heated to reflux for 2 hours. The reaction mixture was cooled to room temperature, water (20 mL) was added, the phases were separated and the aqueous phase was extracted with diethyl ether (2×15 mL). The aqueous phase was acidified with conc. HCl (1.1 mL) and then lyophilized to give a white solid which was recrystallized from MeOH to afford compound **18** as the tris‐hydrochloride salt as a white solid (0.315 g, 31 % over 3 steps). ^1^H NMR (399.8 MHz, D_2_O): δ=1.15 (s, 3H, C*H*_3_), 3.09 (s, 6H, 3×C*H*_2_NH_2_) ppm. ^13^C NMR (100.5 MHz, D_2_O): δ=16.8 (*C*H_3_), 35.2 (quat), 42.6 (3×*C*H_2_NH_2_) ppm.

#### Compound (19 b)

Obtained from amine **18** (0.086 g, 0.378 mmol) and ester **7 b** (0.311 g, 1.25 mmol, 3.3 equivalents) as a light‐yellow solid (0.041 g, 15 %). Triethylamine (1 mL) was used as a co‐solvent. EtOAc/MeOH (95 : 5→90 : 10) was used as the chromatography eluent. ^1^H NMR (399.8 MHz, CDCl_3_, Me_4_Si): δ=1.11 (s, 3H, C*H*_3_), 1.44 (s, 18H, 6×C*H*_3_), 1.49 (s, 18H, 6×C*H*_3_), 2.03 (s, 6H, 3×C*H*_2_), 3.48 (d, *J*=6.9 Hz, 6H, 3×C*H*_2_NH), 9.16 (t, *J*=6.9 Hz, 3H, 3×N*H*) ppm. ^13^C NMR (100.5 MHz, CDCl_3_, Me_4_Si): δ=19.3 (*C*H_3_), 29.1 (6×*C*H_3_), 29.8 (6×*C*H_3_), 40.7 (3×quat), 41.6 (3×quat), 42.8 (quat), 43.5 (3×*C*H_2_), 52.9 (3×*C*H_2_NH), 157.3 (3×quat), 163.5 (3×quat), 169.9 (3×quat), 172.2 (3×quat) ppm. HRMS (NESI): calcd. for C_38_H_54_N_12_O_3_ [M+H]^+^ 727.4520; found 727.4523.

#### Compound (21)^[25]^

1,3,5‐Tris(bromomethyl)‐2,4,6‐triethylbenzene **20** (0.843 g, 1.91 mmol) was dissolved in DMF (20 mL) and NaN_3_ (0.621 g, 9.56 mmol, 5 equivalents) was added. The solution was heated at 90 °C for 4 hours and then the flask was allowed to cool to room temperature and stirring was continued overnight. Water (100 mL) was added and the solution was extracted with diethyl ether (3×30 mL). The combined organic extracts were washed with water (4×30 mL), dried over MgSO_4_ and evaporated to afford the intermediate tris‐azide **21** (0.594 g, 95 %) as an off‐white solid which was used in the next step without further purification. ^1^H NMR (399.8 MHz, CDCl_3_, Me_4_Si): δ=1.22 (t, *J*=7.2 Hz, 9H, 3×C*H*_3_CH_2_), 2.83 (q, *J*=7.2 Hz, 6H, 3×CH_3_C*H*_2_), 4.47 (s, 6H, 3×C*H*_2_N_3_) ppm.

#### Compound (22)^[25]^

The intermediate tris‐azide **21** (0.592 g, 1.81 mmol) was dissolved in THF (15 mL) and water (0.35 mL) was added. PPh_3_ (3.18 g, 12.1 mmol, 6.7 equivalents) was added in small portions over 15 minutes to the vigorously stirred reaction mixture, and stirring was continued for 3 days. The solvents were evaporated and HCl (6 M, 30 mL) was added to the residue. The residue was sonicated and the insoluble solids were filtered and washed with water (10 mL). The filtrate was extracted with DCM (2×20 mL) and diethyl ether (20 mL). The aqueous filtrate was then neutralised with solid NaOH to pH 14 and then extracted with DCM (3×30 mL). The combined organic extracts were dried over MgSO_4_ and evaporated to afford the crude amine **22** (0.249 g, 55 %) as an off‐white solid which was used in the next step without further purification. ^1^H NMR (399.8 MHz, CDCl_3_, Me_4_Si): δ=1.22 (t, *J*=7.2 Hz, 9H, 3×C*H*_3_CH_2_), 2.81 (q, *J*=7.2 Hz, 6H, 3×CH_3_C*H*_2_), 3.86 (s, 6H, 3×C*H*_2_NH_2_) ppm.

#### Compound (23 b)

Obtained from amine **22** (0.05 g, 0.200 mmol) and ester **7 b** (0.20 g, 0.802 mmol, 4 equivalents) as a yellow solid (0.141 g, 82 %). Petroleum ether/EtOAc (50 : 50→0 : 100) was used as the chromatography eluent. ^1^H NMR (399.8 MHz, CDCl_3_, Me_4_Si): δ=1.28 (t, *J*=7.3 Hz, 9H, 3×C*H*_3_), 1.43 (s, 36H, 12×C*H*_3_), 2.01 (s, 6H, 3×C*H*_2_), 2.82 (q, *J*=7.3 Hz, 6H, 3×C*H*_2_), 4.78 (d, *J*=4.5 Hz, 6H, 3×C*H*_2_NH), 7.94 (t, *J*=4.5 Hz, 3H, 3×N*H*) ppm. ^13^C NMR (100.5 MHz, CDCl_3_, Me_4_Si): δ=16.6 (3×*C*H_3_), 23.4 (3×*C*H_2_), 29.1 (6×*C*H_3_), 29.7 (6×*C*H_3_), 38.5 (3×*C*H_2_NH), 40.6 (3×quat), 41.6 (3×quat), 52.8 (3×*C*H_2_NH), 131.9 (3×quat), 144.6 (3×quat), 156.8 (3×quat), 161.3 (3×quat), 169.8 (3×quat), 172.7 (3×quat) ppm. HRMS (NESI): calcd. for C_48_H_70_N_13_O_3_ [M+NH_4_]^+^ 876.5719; found 876.5717.

### Solvent Extraction Experiments

The aqueous solutions were prepared by spiking nitric acid solutions (0.001–4 M) with stock solutions of ^241^Am, ^152^Eu and ^244^Cm tracers (10 μL) in nitric acid. Ultrapure water (18.2 MΩcm) was used for all dilutions. The radiotracers ^241^Am, ^244^Cm and ^152^Eu were supplied by Isotopendienst M. Blaseg GmbH, Waldburg (Germany), Oak Ridge National Laboratory, Oak Ridge (USA), and Eckert & Ziegler Nuclitec GmbH, Braunschweig (Germany), respectively. Solutions of the ligands **9**, **12**, **14**, **19 b** and **23 b** (0.01 M) were prepared by dissolving **9**, **12**, **14**, **19 b** and **23 b** in 1‐octanol, cyclohexanone or nitrobenzene. Each organic phase (500 μL) was shaken separately with each of the aqueous phases (500 μL) for one hour at 22 °C using a thermostatted aluminum block installed on an IKA Vibrax Orbital Shaker Model VXR (2,200 rpm). After phase separation by centrifugation, 200 μL aliquots of each phase were withdrawn for activity measurement. Measurements of the γ‐ray emitters ^241^Am and ^152^Eu were performed with a HPGe γ‐ray spectrometer, EG & G Ortec, Munich (Germany). The γ‐lines at 59.5 keV, and 121.8 keV were examined for ^241^Am, and ^152^Eu, respectively. The nuclides ^241^Am and ^244^Cm were measured by means of alpha spectrometry with an Alpha Spectrometer OctêteTM PC obtained from EG & G Ortec, Munich (Germany). The distribution ratio (*D*) was calculated as the ratio between the radioactivity/concentration in the organic and the aqueous phase. The separation factor (SF) is calculated as the ratio between the distribution ratios of the corresponding metals. Distribution ratios between 0.01 and 100 exhibit a maximum error of ±5 %. The error may be up to ±20 % for smaller and larger values.

### NMR Titrations

Stock solutions (0.01 M) of each of the ligands **9 b**, **12 b**, **12 c**, **14 b** and **23 b**, and of the metal nitrate salts La(NO_3_)_3_.6H_2_O, Lu(NO_3_)_3_.H_2_O and Y(NO_3_)_3_.6H_2_O (Aldrich) were prepared in CD_3_CN (Fluorochem). A 0.5 mL aliquot of the appropriate ligand solution was placed in an NMR tube and the ^1^H NMR spectrum was recorded at 399.8 MHz on a JEOL ECS400FT Delta spectrometer. The appropriate lanthanide salt solution was added to the NMR tube in 50 μL aliquots (ie: 0.1 equivalents each time) using a calibrated Gilson 100 μL micropipette. The tube was inverted several times to ensure full mixing and the ^1^H NMR spectrum was recorded after each successive addition until the resonances of the free ligand had completely disappeared and/or until no further spectral changes were observed. Homogeneous solutions were obtained after each addition. In the titration of ligand **23 b** with La(NO_3_)_3_, the relative ratios of the different species present were calculated from the relative integrals of a suitable one‐proton resonance of the ligand **23 b**. These values were normalized such that, for a given one‐proton resonance, the total integration for all species present equalled unity. The species distributions at different metal:ligand ratios were calculated from these normalized relative ratios.

### DFT Calculations

All DFT calculations were carried out using Orca v5.0.4^[30]^ using the ZORA approximation.^[31]^ Calculations were performed using the BP86 functional^[32]^ with the ZORA‐def2‐SVP basis set^[33]^ for atoms other than La, for which the SARC‐ZORA‐TZVP basis set^[34]^ was used. The RI approximation was used, along with the SARC/J auxiliary basis set. This level of theory has been used previously for the calculation of stability constants of f‐block complexes.^[35]^ Solvation in acetonitrile was described using the CPCM model and was used for all calculations.^[36]^ For all La(III) ligand complexes, Architector^[37]^ was used to generate initial structures (between 1 and 6 structures were produced per ligand), which were subsequently optimised before frequency calculations were performed in order to obtain Gibbs free energies. For simplicity, calculations were limited to the formation of 1 : 1 complexes. Energies of multiple different bound geometries were determined, with various denticities and coordination numbers, as given in the Supporting Information (Table S10). Binding energies given in the main text correspond to the minimum values determined for the species given in Table S10. Due to the number of possible geometries that could be adopted, complexes involving H_2_O ligands not directly bound to La were not considered. La(NO_3_)_3_(H_2_O)_5_ was assumed to be the unbound La(III) species as it was calculated to have a lower Gibbs free energy of binding than La(NO_3_)_3_(H_2_O)_4_ and La(NO_3_)_3_(H_2_O)_6_, and is consistent with the reported stoichiometry of the crystal structure.^[38]^ To determine the energies of the La(NO_3_)_*x*_(H_2_O)_*y*_^3−*x*^ species, multiple geometries were optimised for each complex, again generated using Architector, before DFT calculations were performed using the methods described above. The specific equations used to determine the binding energies for each ligand are given in the Supporting Information (Scheme S1). The energy of H_2_O was calculated at the same level of theory.

## Conflict of Interests

The authors declare no conflict of interest.

## Supporting information

As a service to our authors and readers, this journal provides supporting information supplied by the authors. Such materials are peer reviewed and may be re‐organized for online delivery, but are not copy‐edited or typeset. Technical support issues arising from supporting information (other than missing files) should be addressed to the authors.

Supporting Information

## Data Availability

The data that support the findings of this study are available in the supplementary material of this article.
