# Supplementary material for: Evaluation of Multidentate Ligands Derived from Ethyl 1,2,4‐triazine‐3‐carboxylate Building Blocks as Potential An(III)‐Selective Extractants for Nuclear Reprocessing
Source: ChemistryOpen. 2024 Nov 26;14(6):e202400306. doi: 10.1002/open.202400306 (PMC13062950; doi:10.1002/open.202400306)
Supplement: Supplementary file 1 — Supporting Information [file OPEN-14-e202400306-s001.pdf]

ChemistryOpen

Supporting Information

Evaluation of Multidentate Ligands Derived from Ethyl 1,2,4-triazine-3-carboxylate Building Blocks as Potential An(III)-Selective Extractants for Nuclear Reprocessing

Andrey V. Zaytsev, Petr Distler, Jan John, Andreas Wilden, Giuseppe Modolo, Mark Sims, and Frank W. Lewis*

ELECTRONIC SUPPLEMENTARY INFORMATION

for the paper entitled

Evaluation of Multidentate Ligands Derived from Ethyl 1,2,4-triazine-3-carboxylate Building Blocks as Potential An(III)-Selective Extractants for Nuclear Reprocessing

Andrey V. Zaytsev,[a] Petr Distler,[b] Jan John,[b] Andreas Wilden,[c] Giuseppe Modolo,[c] Mark Sims[a] and Frank W. Lewis*[a]

[a] *Department of Applied Sciences, Faculty of Health and Life Sciences, Northumbria University, Newcastle upon Tyne, Tyne and Wear NE1 8ST, UK. E-mail: frank.lewis@northumbria.ac.uk*

[b] *Department of Nuclear Chemistry, Czech Technical University in Prague, Břehová 7, 11519 Prague 1, Czech Republic. E-mail: jan.john@fjfi.cvut.cz or petr.distler@fjfi.cvut.cz*

[c] *Forschungszentrum Jülich GmbH, Institute of Fusion Energy and Nuclear Waste Management - Nuclear Waste Management (IFN-2), 52428 Jülich, Germany. E-mail: g.modolo@fz-juelich.de or a.wilden@fz-juelich.de*

CONTENTS			PAGE

1.		**NMR Spectra**	S3
2.		**Mass Spectra**	S37
3.		**Solvent Extraction Studies**	S50
4.		**NMR Titrations with Lanthanide Salts**	S55
	4.1	NMR Titrations with Ligand **9b**	S55
	4.2	NMR Titrations with Ligand **12b**	S57
	4.3	NMR Titrations with Ligand **12c**	S60
	4.4	NMR Titrations with Ligand **14b**	S62
	4.5	NMR Titrations with Ligand **23b**	S65
5.		**DFT Calculations**	S83
	5.1	Coordinates of Optimized Geometry of La(III) Complex of Ligand **1**	S86
	5.2	Coordinates of Optimized Geometry of La(III) Complex of Ligand **9b**	S88
	5.3	Coordinates of Optimized Geometry of La(III) Complex of Ligand **12b**	S90
	5.4	Coordinates of Optimized Geometry of La(III) Complex of Ligand **14b**	S92
	5.5	Coordinates of Optimized Geometry of La(III) Complex of Ligand **23b**	S95

1: NMR Spectra

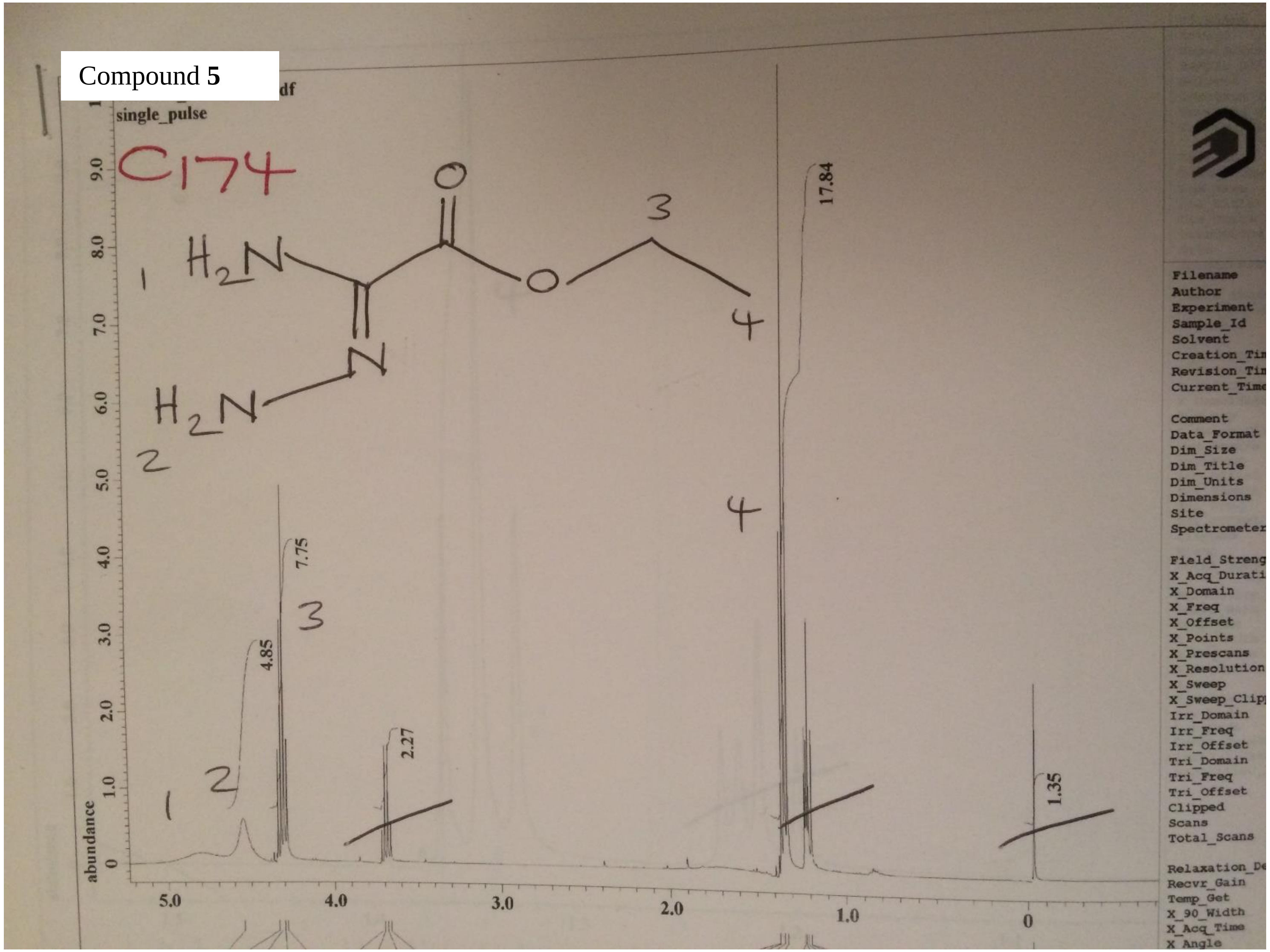

AVZ-I-19-1
single_pulse

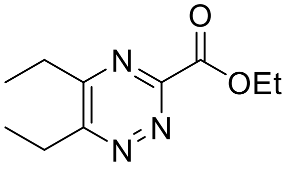

Compound **7a**

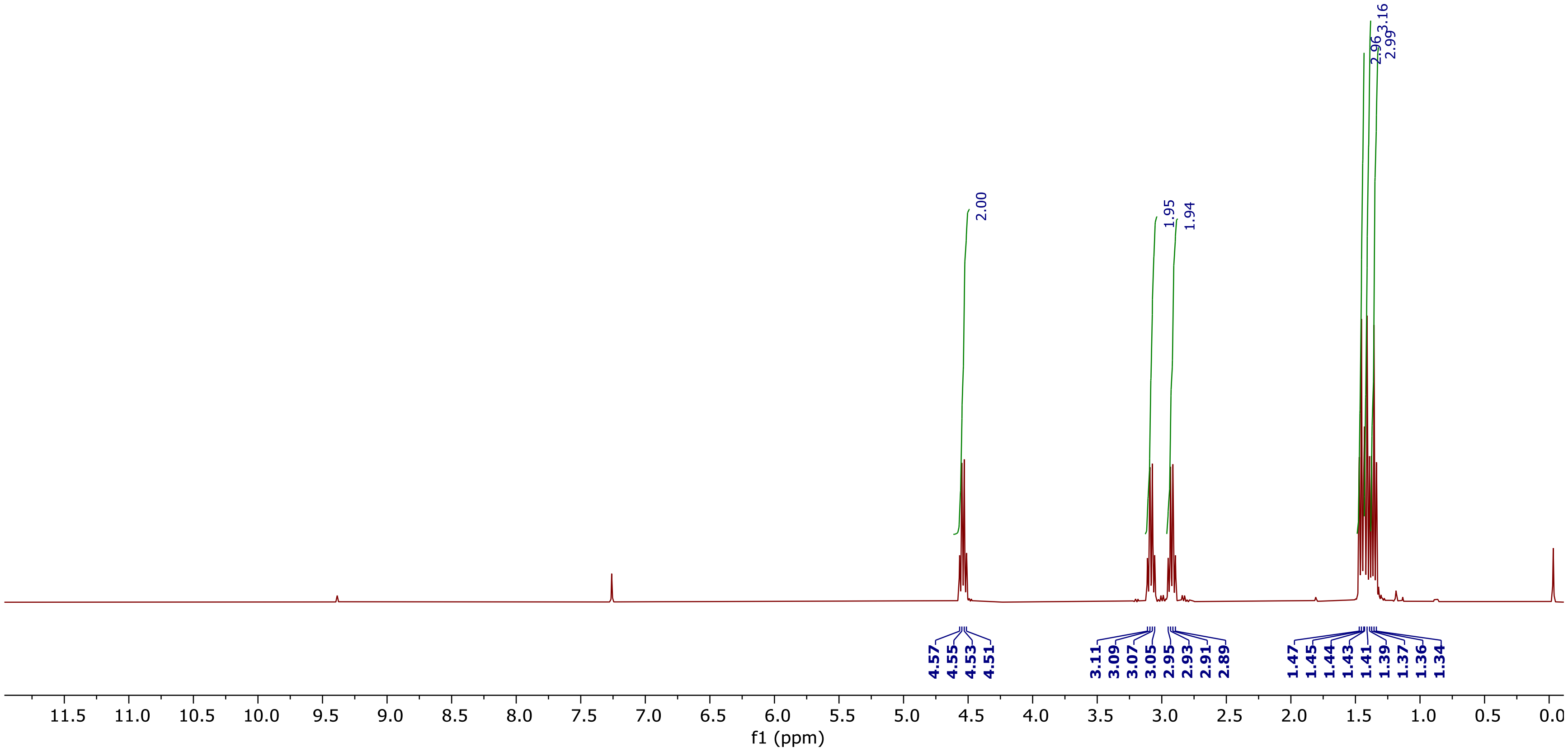

AVZ-I-19-1
single pulse decoupled gated NOE

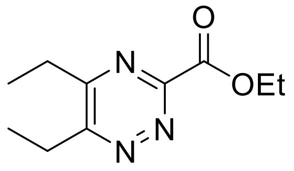

Compound **7a**

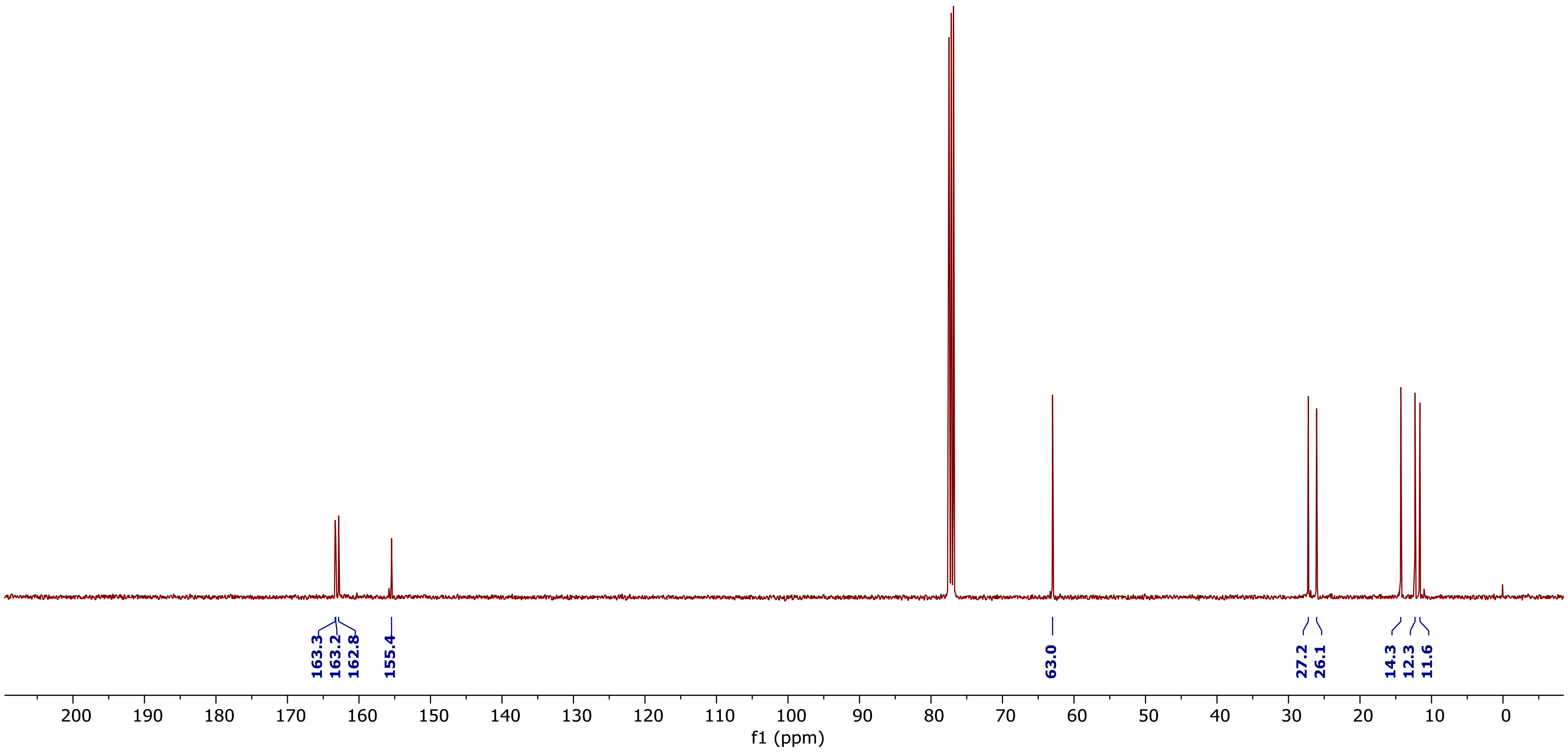

AVZ-I-73-2
single_pulse

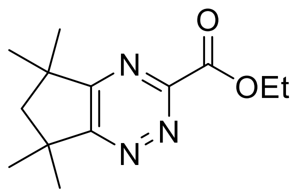

Compound **7b**

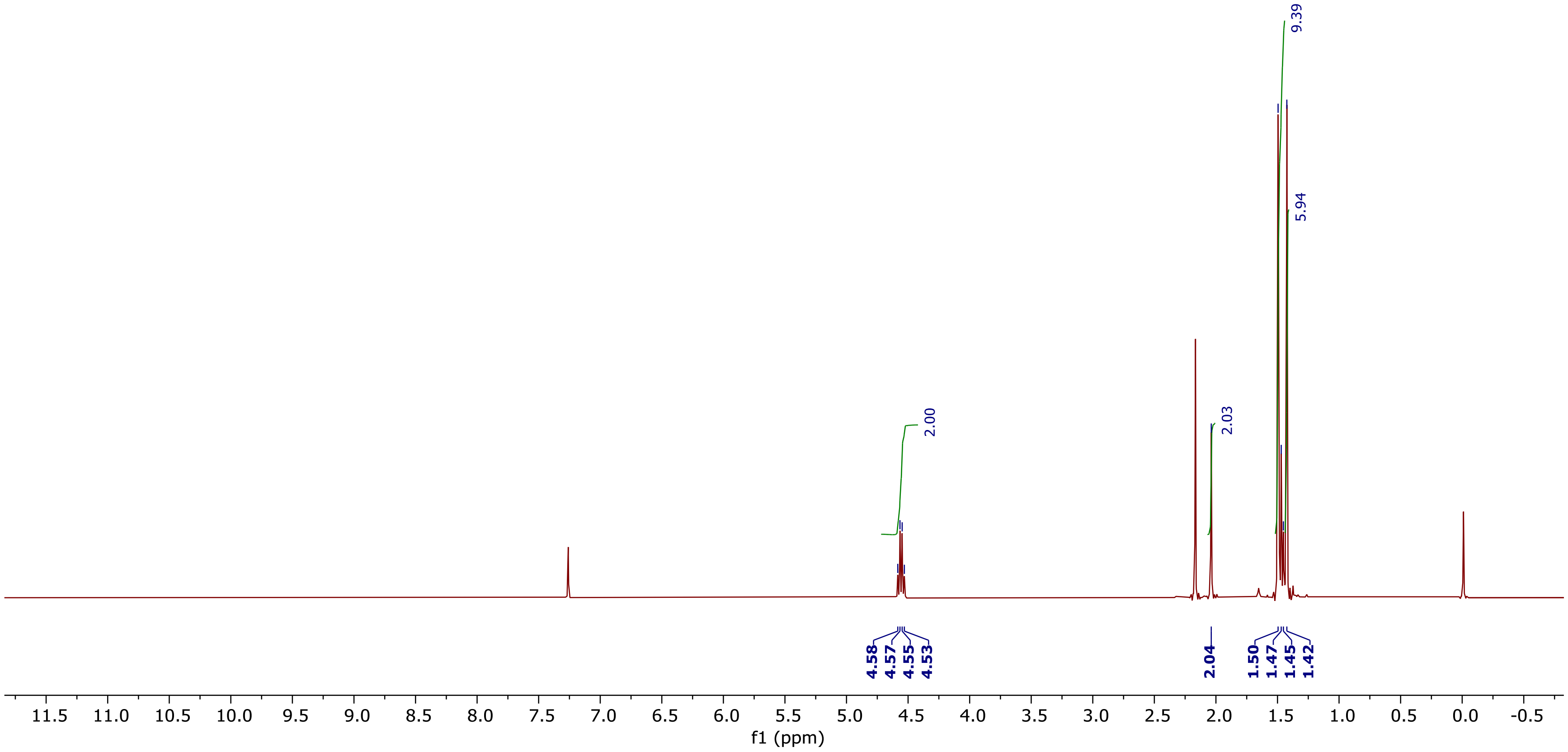

AVZ-I-73-2 1
single pulse decoupled gated NOE

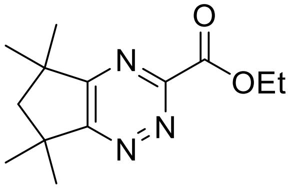

Compound **7b**

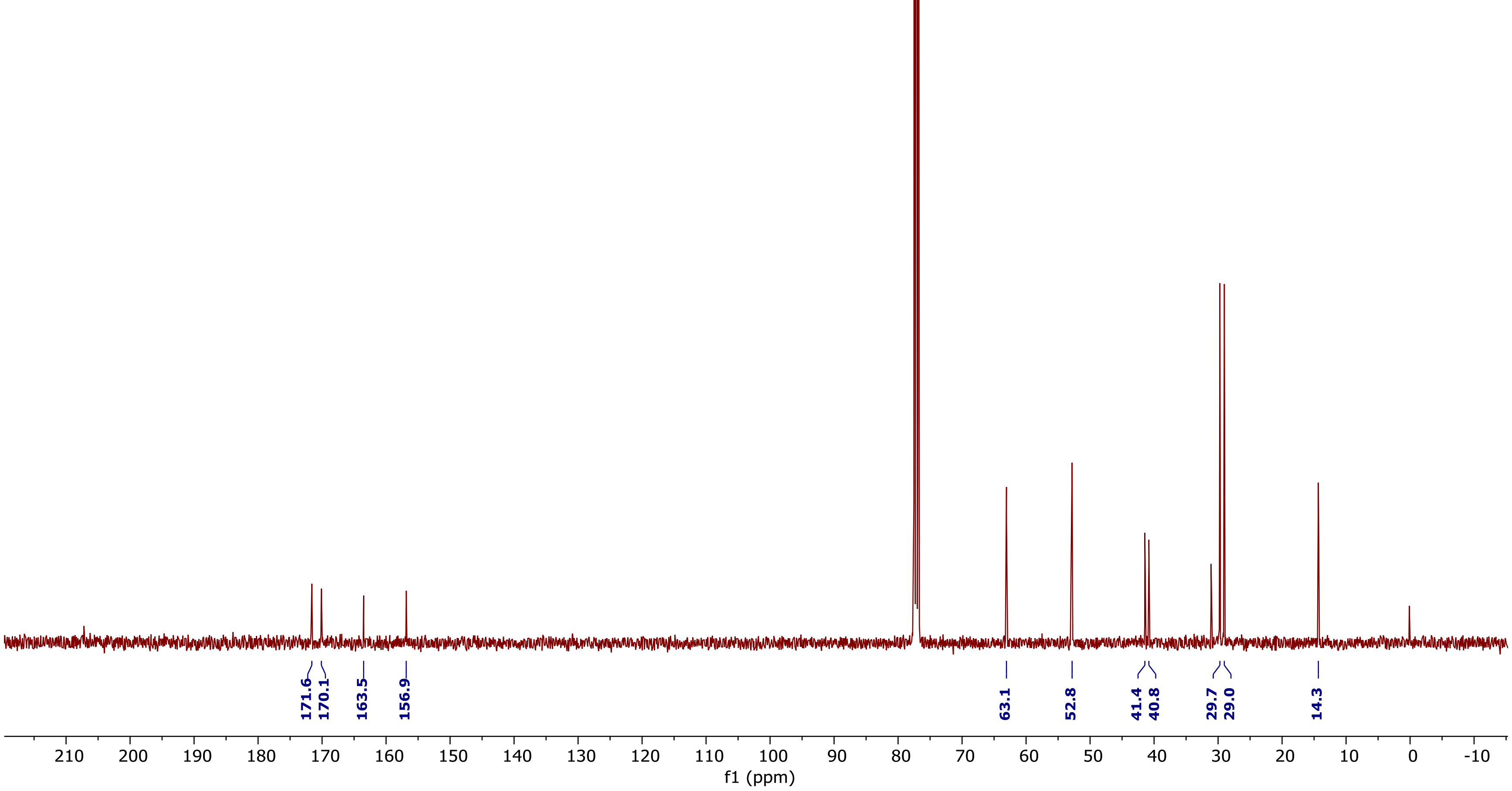

AVZ-I-57-2
single_pulse

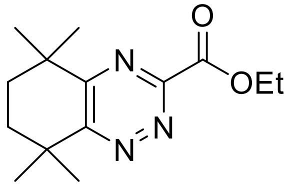

Compound **7c**

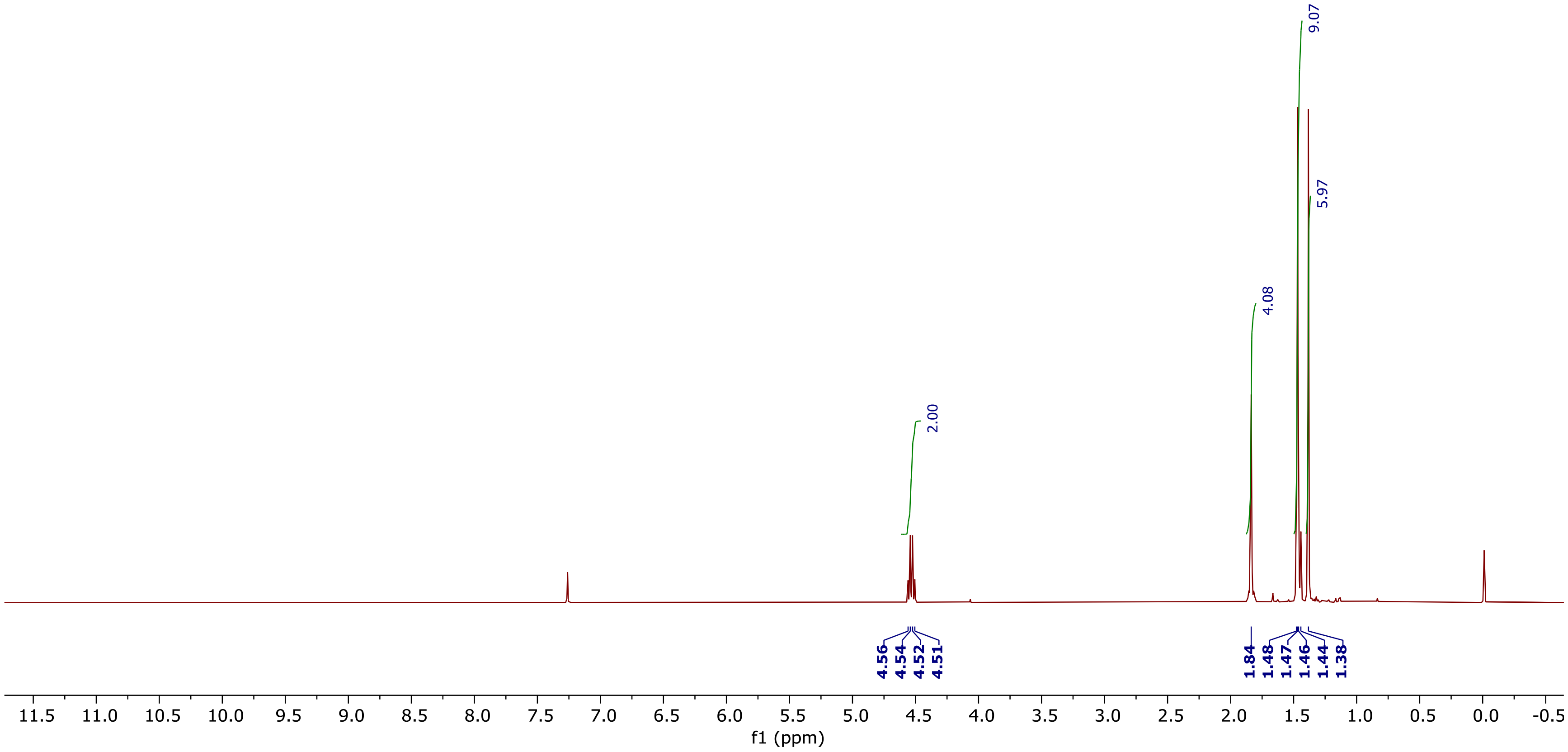

AVZ-I-57-2 1
single pulse decoupled gated NOE

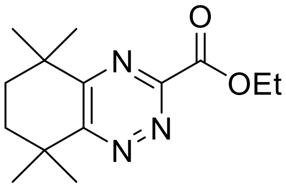

Compound **7c**

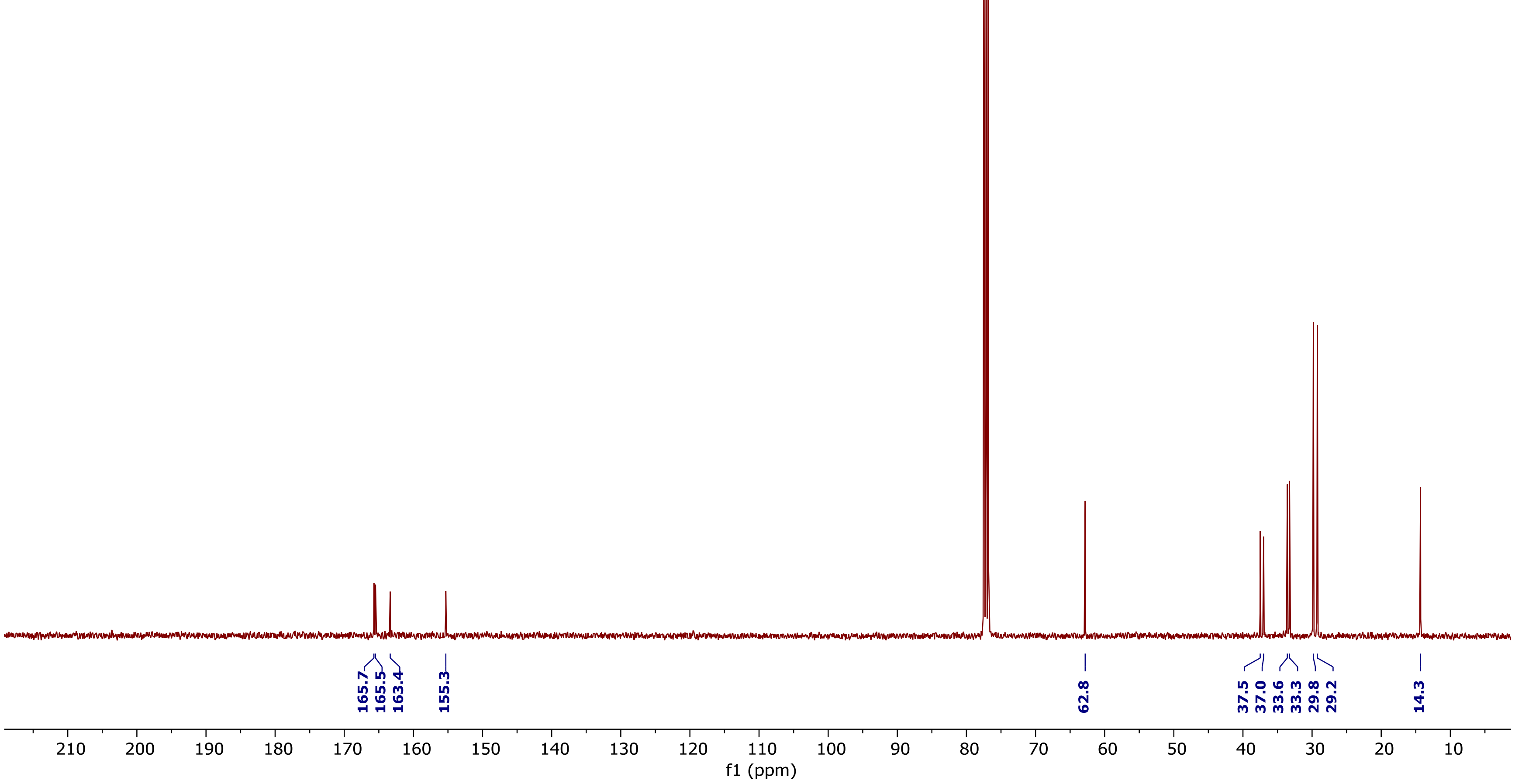

AVZ-I-20-1
single_pulse

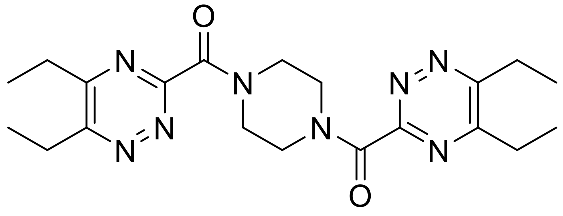

Compound **9a**

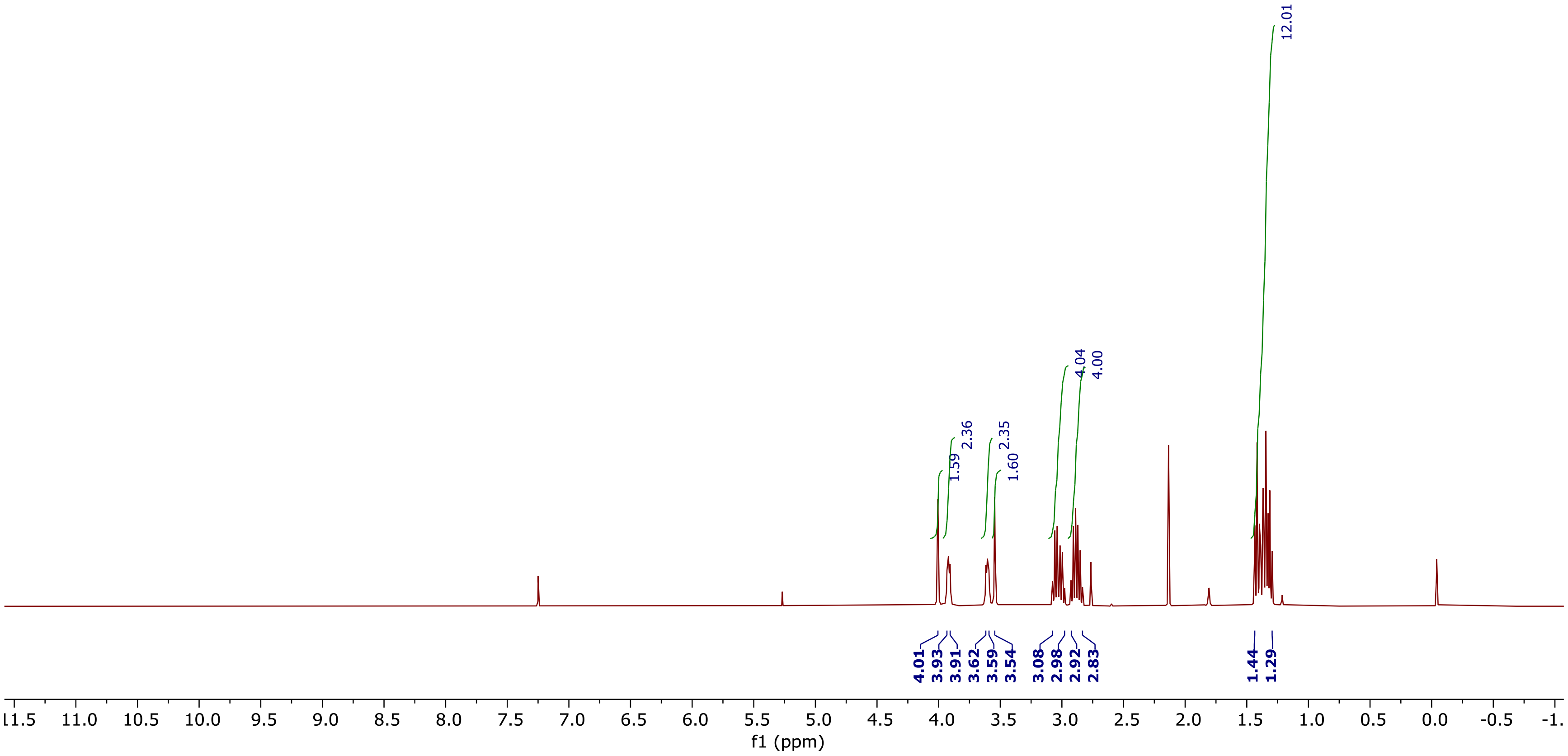

AVZ-I-20-1
single pulse decoupled gated NOE

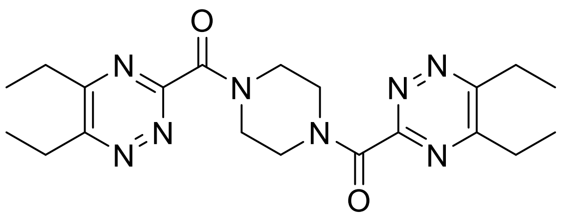

Compound **9a**

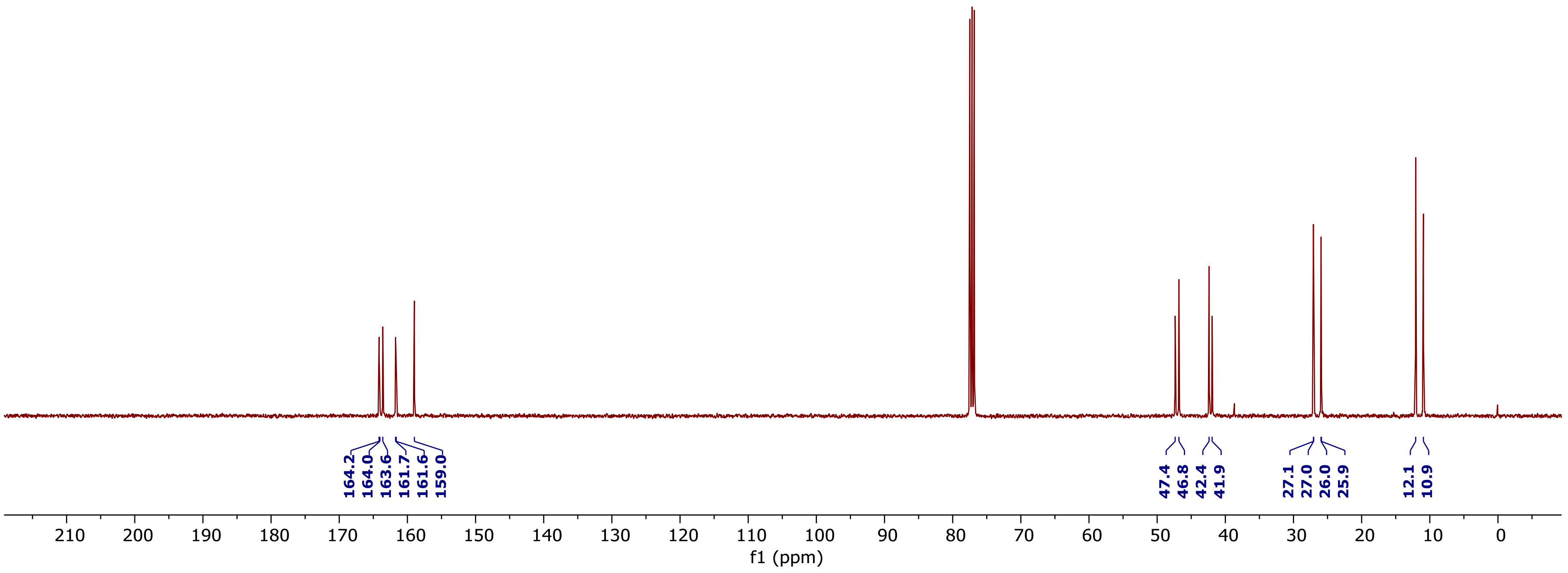

AVZ-I-76-1
single_pulse

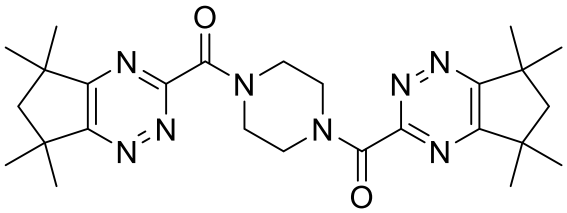

Compound **9b**

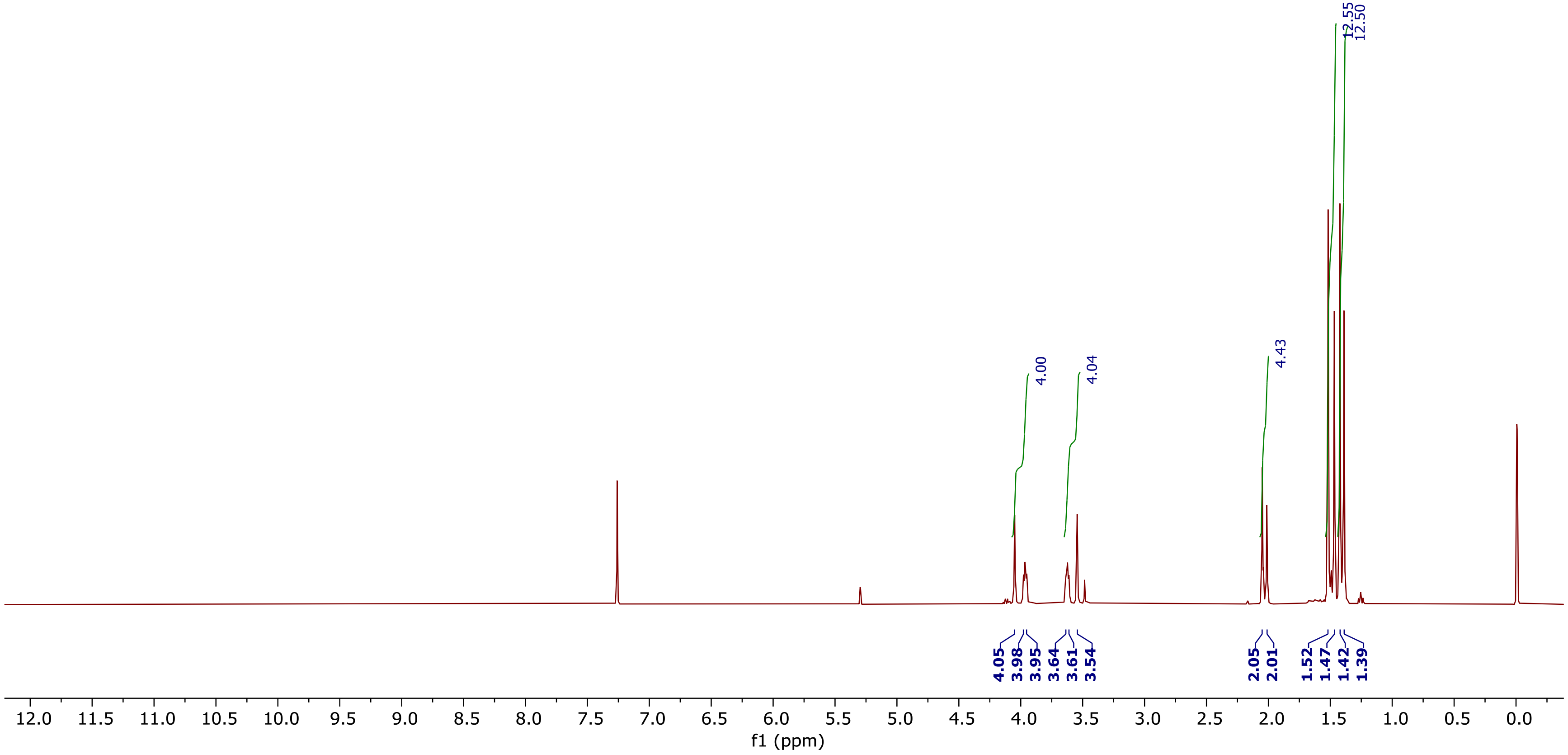

AVZ-I-76-1
single pulse decoupled gated NOE

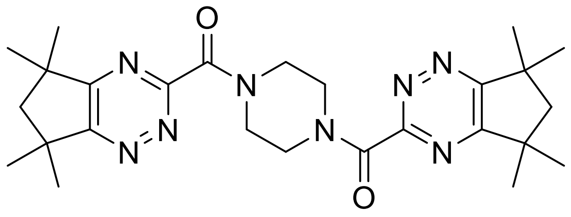

Compound **9b**

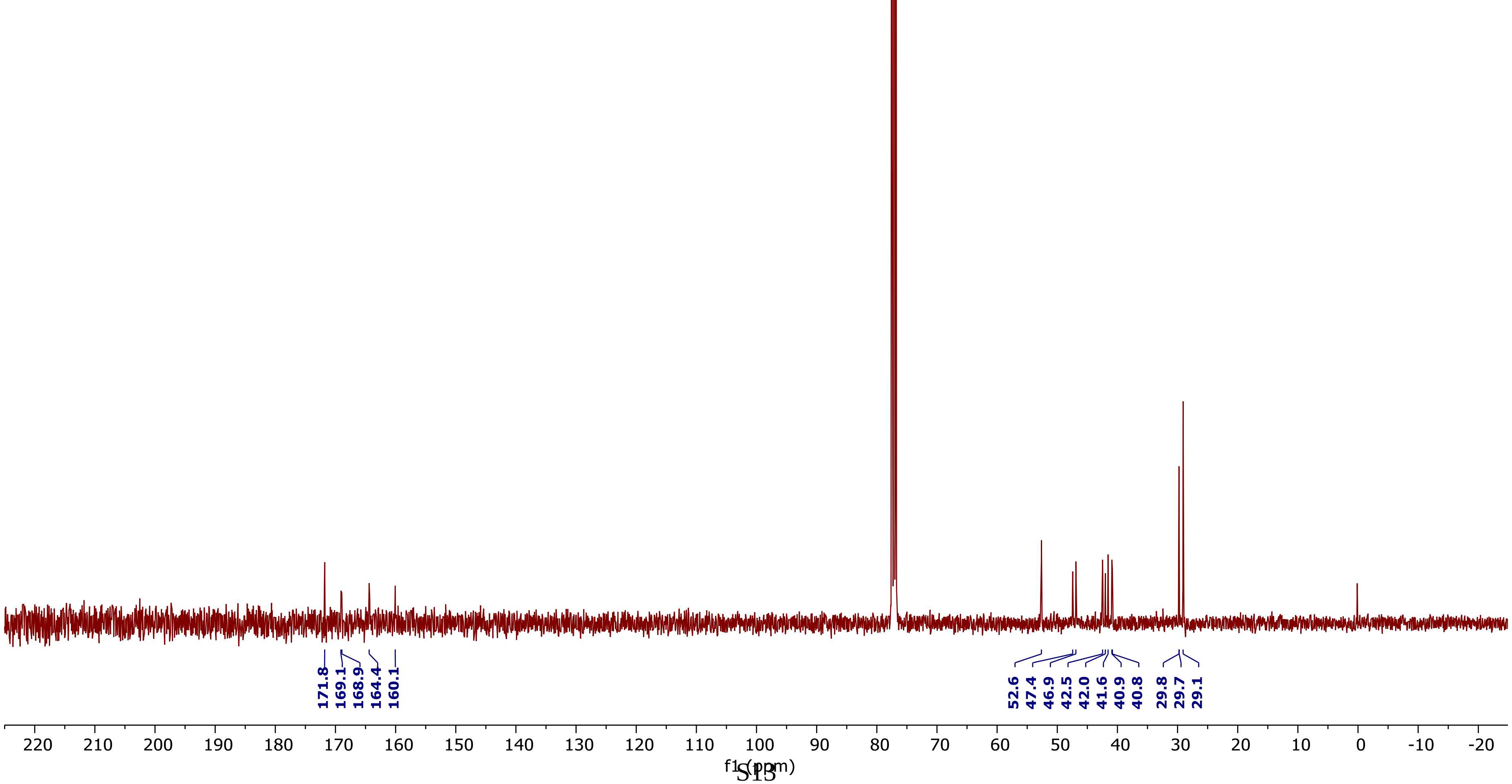

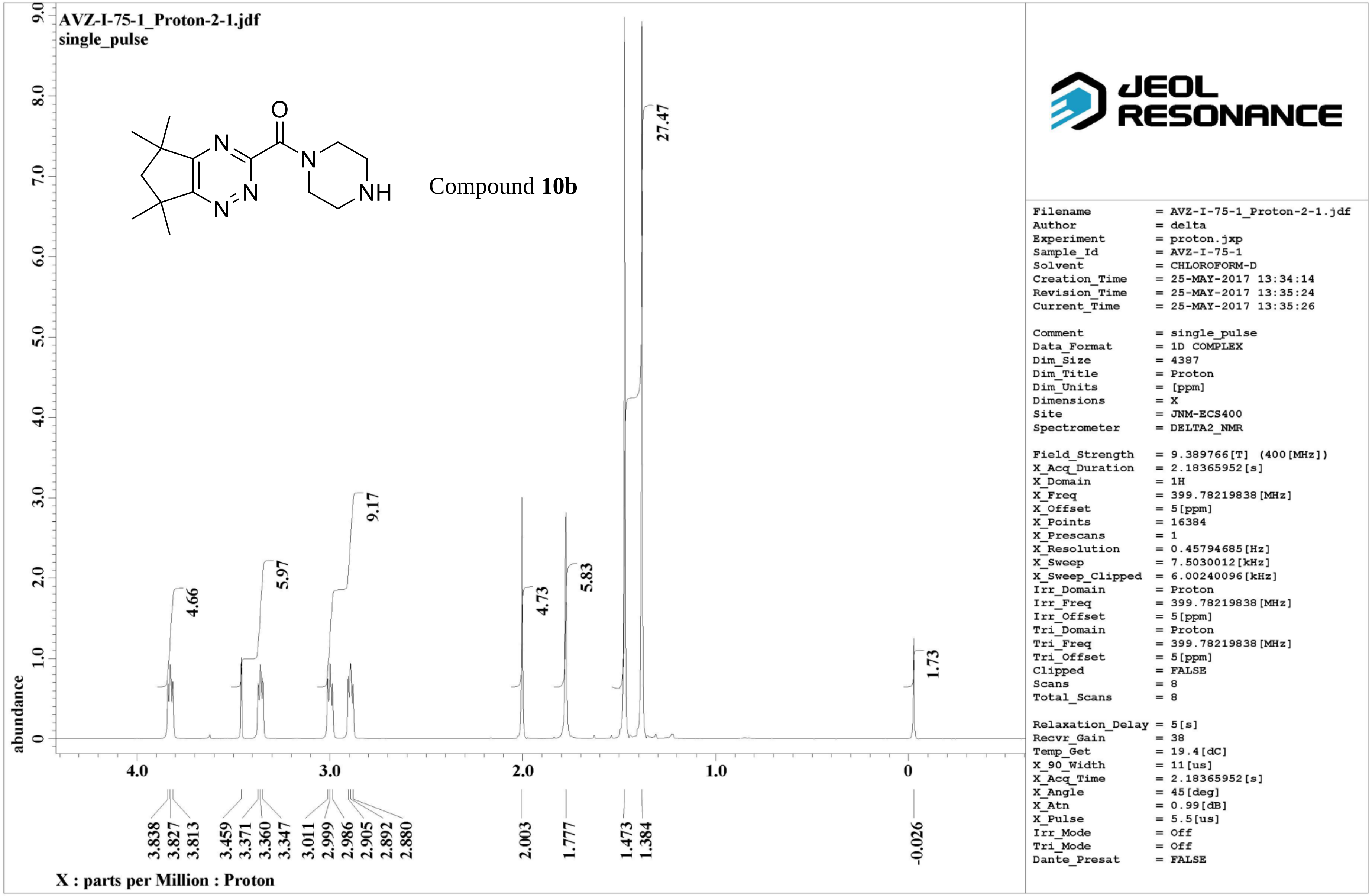
AVZ-I-75-1_Proton-2-1.jdf
single_pulse
Compound **10b**
O
N
N
N
N
NH
abundance
X : parts per Million : Proton
4.0
3.0
2.0
1.0
0
4.66
5.97
9.17
4.73
5.83
27.47
1.73
3.838
3.827
3.813
3.459
3.371
3.360
3.347
3.011
2.999
2.986
2.905
2.892
2.880
2.003
1.777
1.473
1.384
-0.026
JEOL RESONANCE
Filename = AVZ-I-75-1_Proton-2-1.jdf
Author = delta
Experiment = proton.jxp
Sample_Id = AVZ-I-75-1
Solvent = CHLOROFORM-D
Creation_Time = 25-MAY-2017 13:34:14
Revision_Time = 25-MAY-2017 13:35:24
Current_Time = 25-MAY-2017 13:35:26
Comment = single_pulse
Data_Format = 1D COMPLEX
Dim_Size = 4387
Dim_Title = Proton
Dim_Units = [ppm]
Dimensions = X
Site = JNM-ECS400
Spectrometer = DELTA2_NMR
Field_Strength = 9.389766[T] (400[MHz])
X_Acq_Duration = 2.18365952[s]
X_Domain = 1H
X_Freq = 399.78219838[MHz]
X_Offset = 5[ppm]
X_Points = 16384
X_Prescans = 1
X_Resolution = 0.45794685[Hz]
X_Sweep = 7.5030012[kHz]
X_Sweep_Clipped = 6.00240096[kHz]
Irr_Domain = Proton
Irr_Freq = 399.78219838[MHz]
Irr_Offset = 5[ppm]
Tri_Domain = Proton
Tri_Freq = 399.78219838[MHz]
Tri_Offset = 5[ppm]
Clipped = FALSE
Scans = 8
Total_Scans = 8
Relaxation_Delay = 5[s]
Recvr_Gain = 38
Temp_Get = 19.4[dC]
X_90_Width = 11[us]
X_Acq_Time = 2.18365952[s]
X_Angle = 45[deg]
X_Atn = 0.99[dB]
X_Pulse = 5.5[us]
Irr_Mode = Off
Tri_Mode = Off
Dante_Presat = FALSE

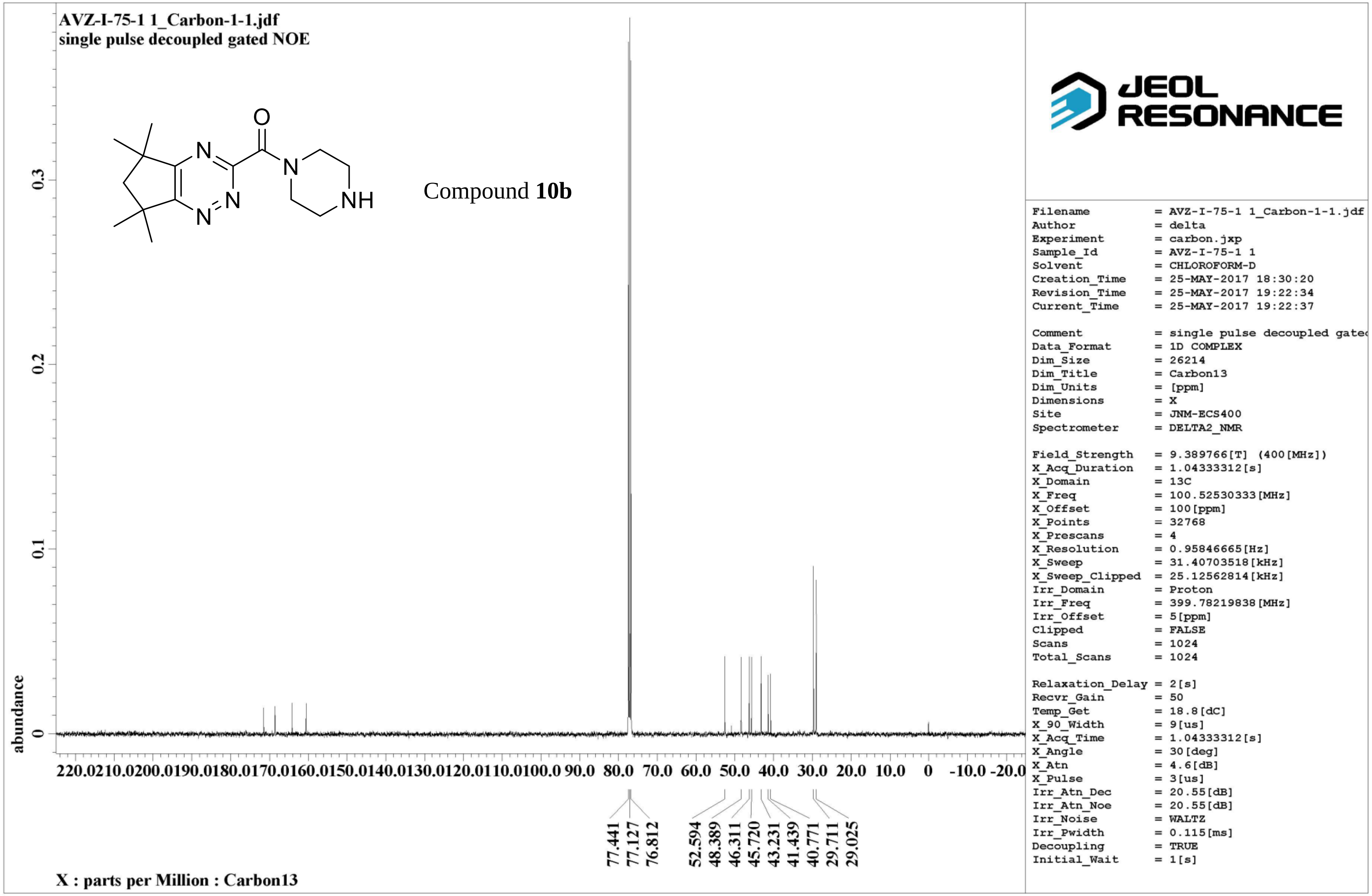
AVZ-I-75-1 1_Carbon-1-1.jdf
single pulse decoupled gated NOE
O
N
N
N
N
NH
Compound 10b
abundance
0
0.1
0.2
0.3
220.0 210.0 200.0 190.0 180.0 170.0 160.0 150.0 140.0 130.0 120.0 110.0 100.0 90.0 80.0 70.0 60.0 50.0 40.0 30.0 20.0 10.0 0 -10.0 -20.0
77.441
77.127
76.812
52.594
48.389
46.311
45.720
43.231
41.439
40.771
29.711
29.025
X : parts per Million : Carbon13
JEOL RESONANCE
Filename = AVZ-I-75-1 1_Carbon-1-1.jdf
Author = delta
Experiment = carbon.jxp
Sample_Id = AVZ-I-75-1 1
Solvent = CHLOROFORM-D
Creation_Time = 25-MAY-2017 18:30:20
Revision_Time = 25-MAY-2017 19:22:34
Current_Time = 25-MAY-2017 19:22:37
Comment = single pulse decoupled gated
Data_Format = 1D COMPLEX
Dim_Size = 26214
Dim_Title = Carbon13
Dim_Units = [ppm]
Dimensions = X
Site = JNM-ECS400
Spectrometer = DELTA2_NMR
Field_Strength = 9.389766[T] (400[MHz])
X_Acq_Duration = 1.04333312[s]
X_Domain = 13C
X_Freq = 100.52530333[MHz]
X_Offset = 100[ppm]
X_Points = 32768
X_Prescans = 4
X_Resolution = 0.95846665[Hz]
X_Sweep = 31.40703518[kHz]
X_Sweep_Clipped = 25.12562814[kHz]
Irr_Domain = Proton
Irr_Freq = 399.78219838[MHz]
Irr_Offset = 5[ppm]
Clipped = FALSE
Scans = 1024
Total_Scans = 1024
Relaxation_Delay = 2[s]
Recvr_Gain = 50
Temp_Get = 18.8[dC]
X_90_Width = 9[us]
X_Acq_Time = 1.04333312[s]
X_Angle = 30[deg]
X_Atn = 4.6[dB]
X_Pulse = 3[us]
Irr_Atn_Dec = 20.55[dB]
Irr_Atn_Noe = 20.55[dB]
Irr_Noise = WALTZ
Irr_Pwidth = 0.115[ms]
Decoupling = TRUE
Initial_Wait = 1[s]

AVZ-III-04-2
single_pulse

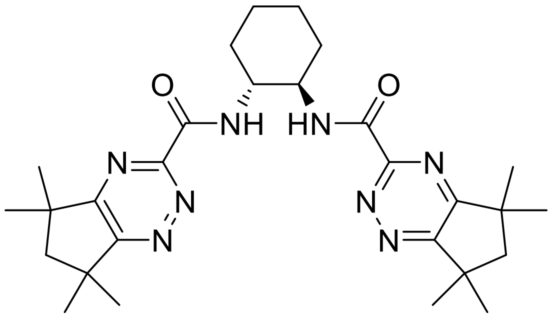

Compound **12b**

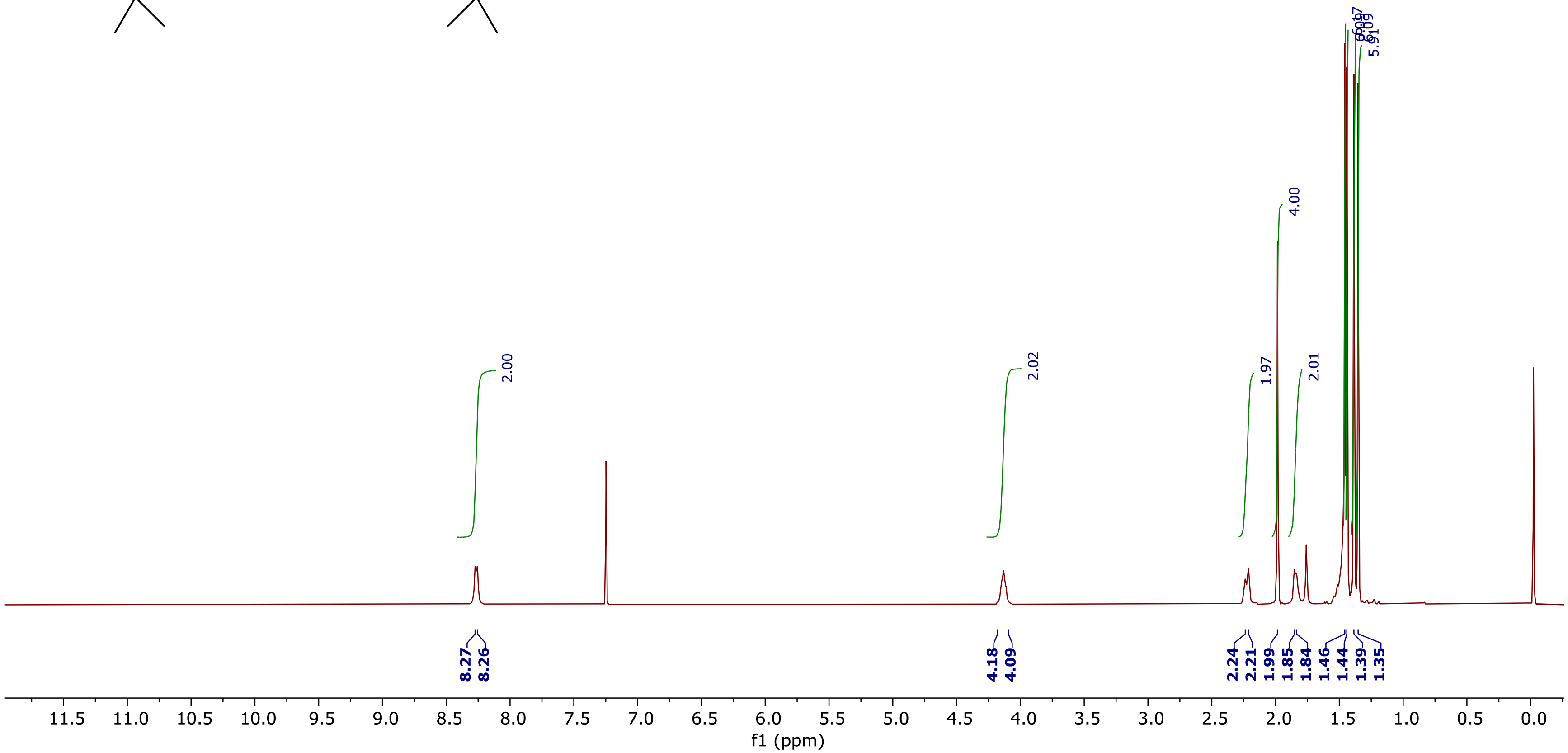

AVZ-III-04-2
single pulse decoupled gated NOE

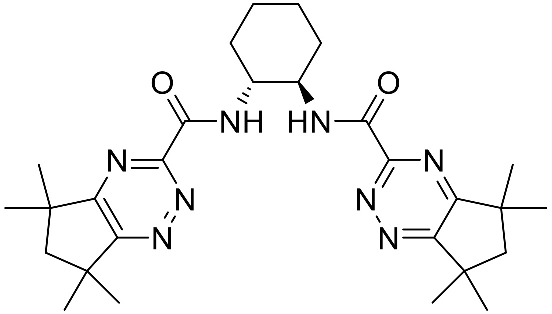

Compound **12b**

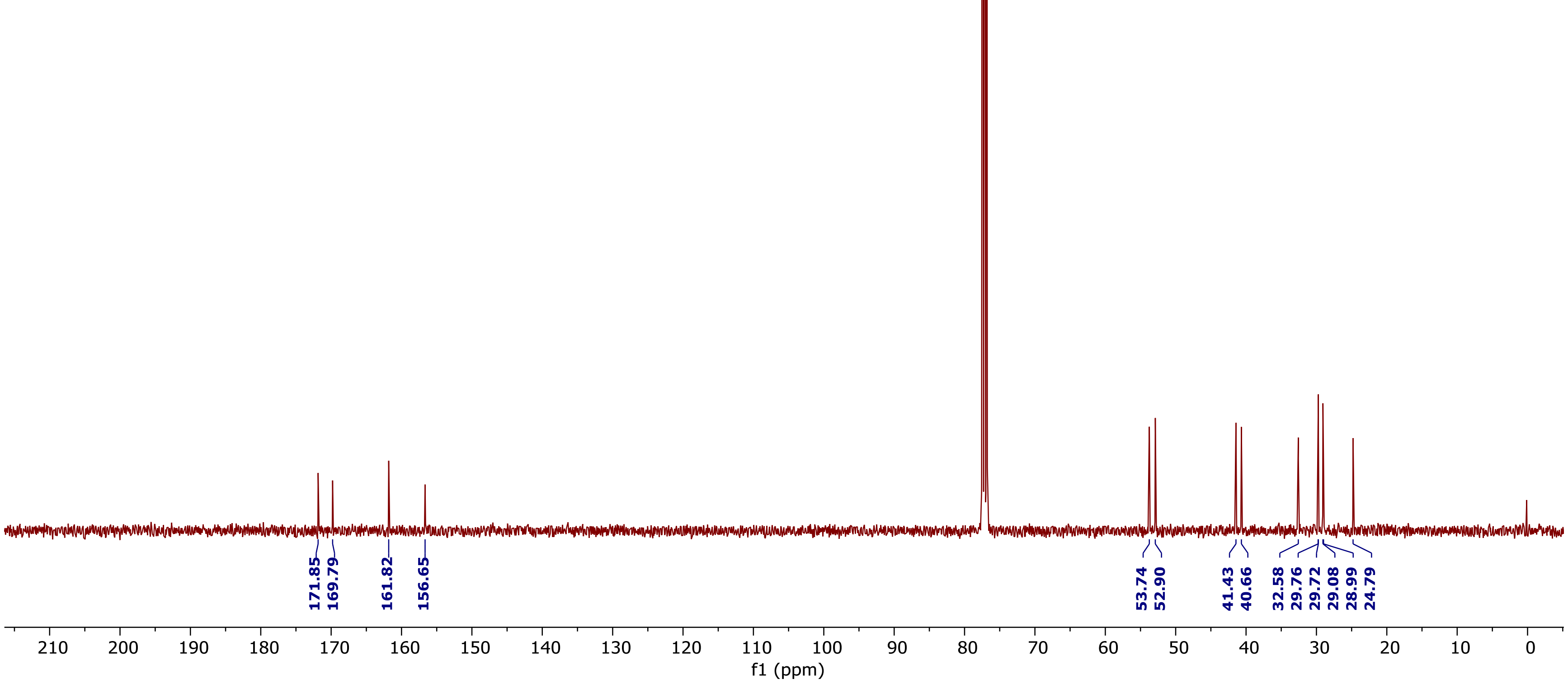

AVZ-III-24-1
single_pulse

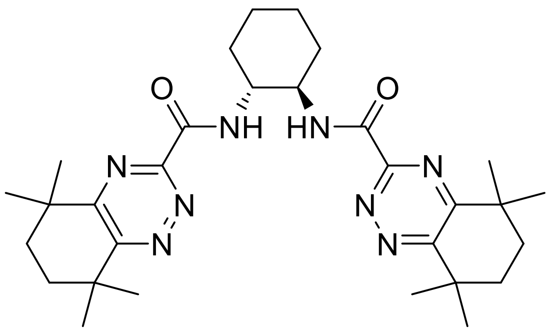

Compound **12c**

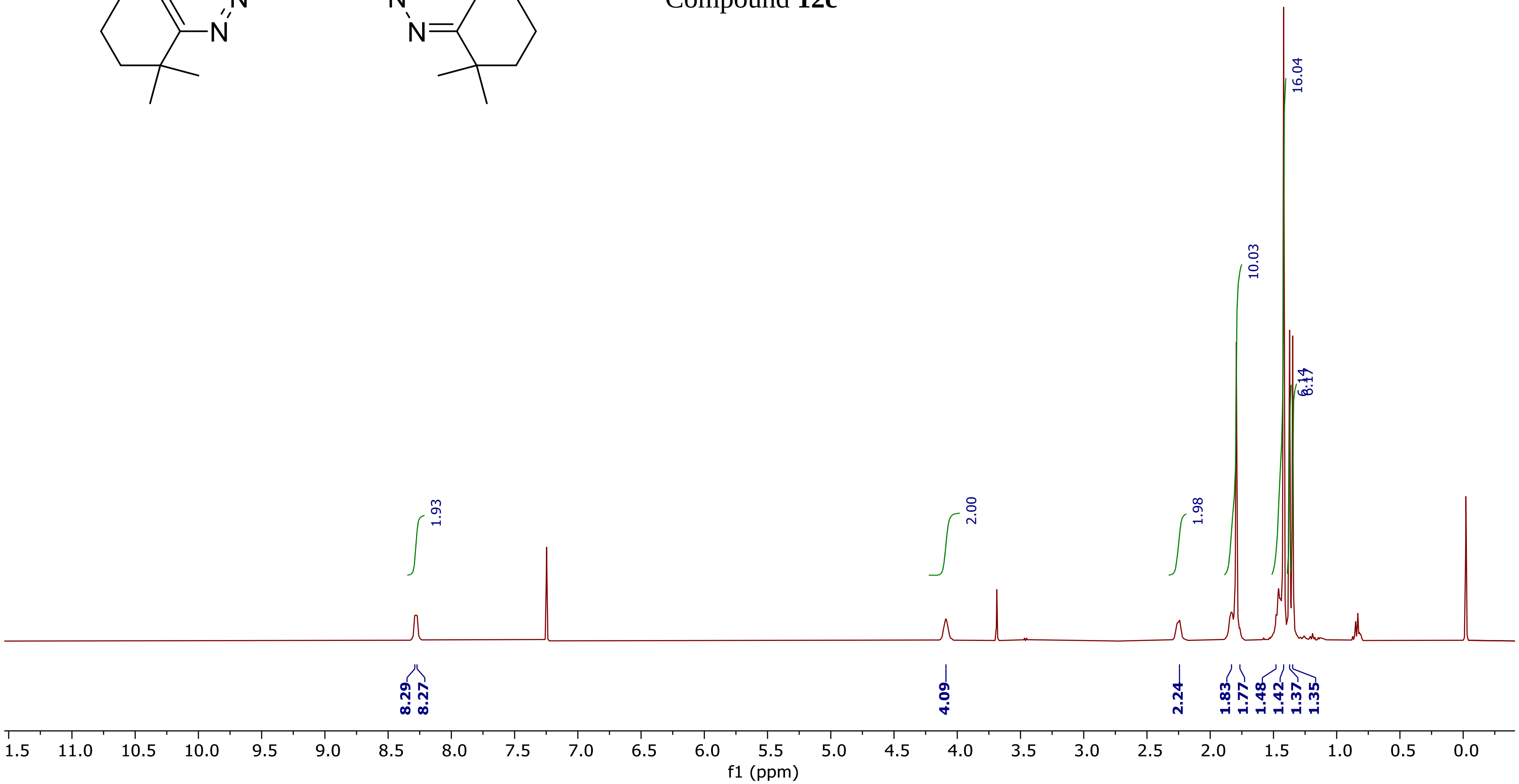

AVZ-III-24-1
single pulse decoupled gated NOE

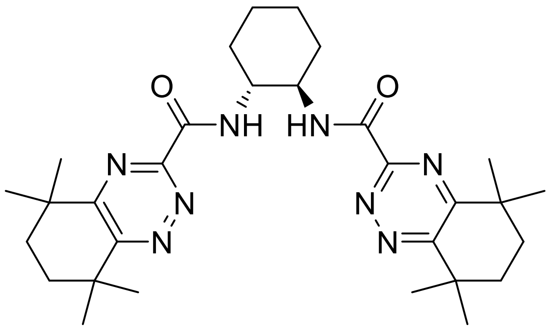

Compound **12c**

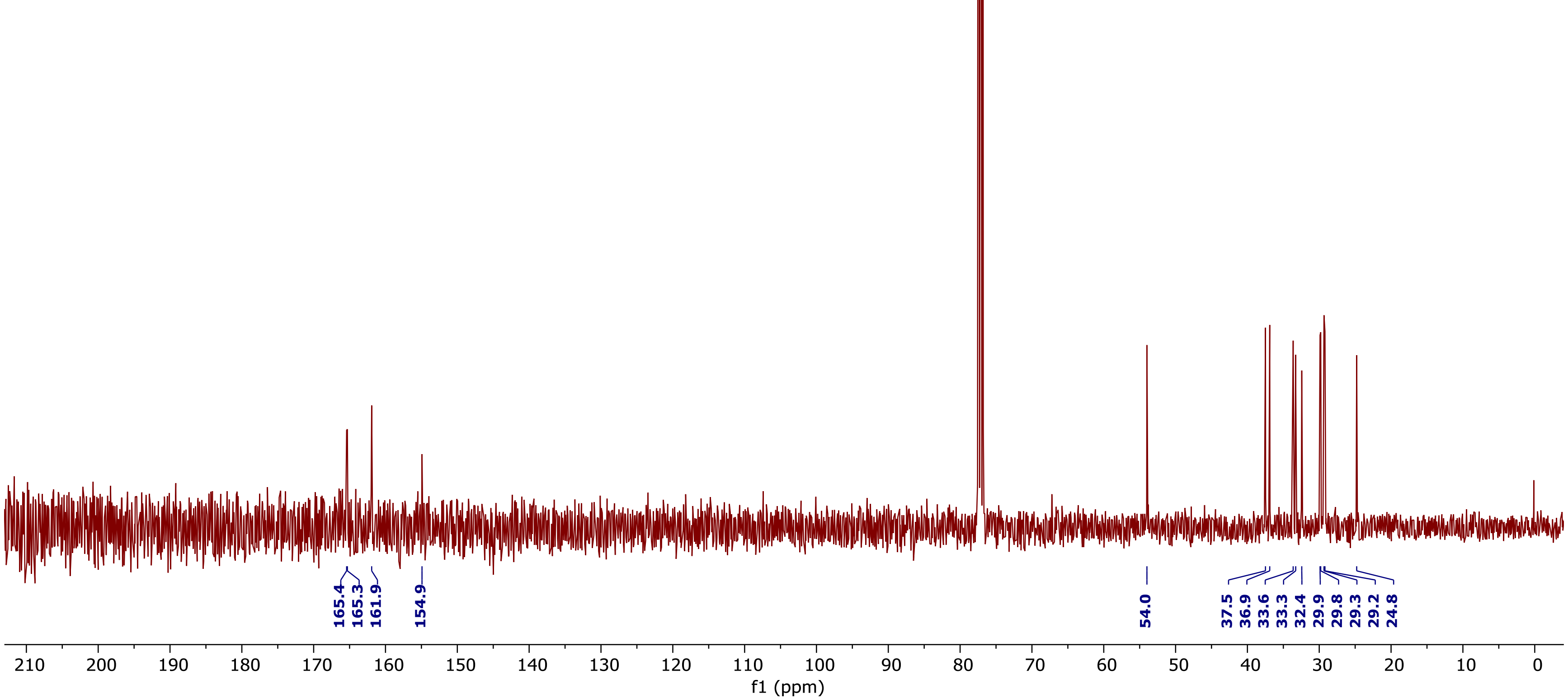

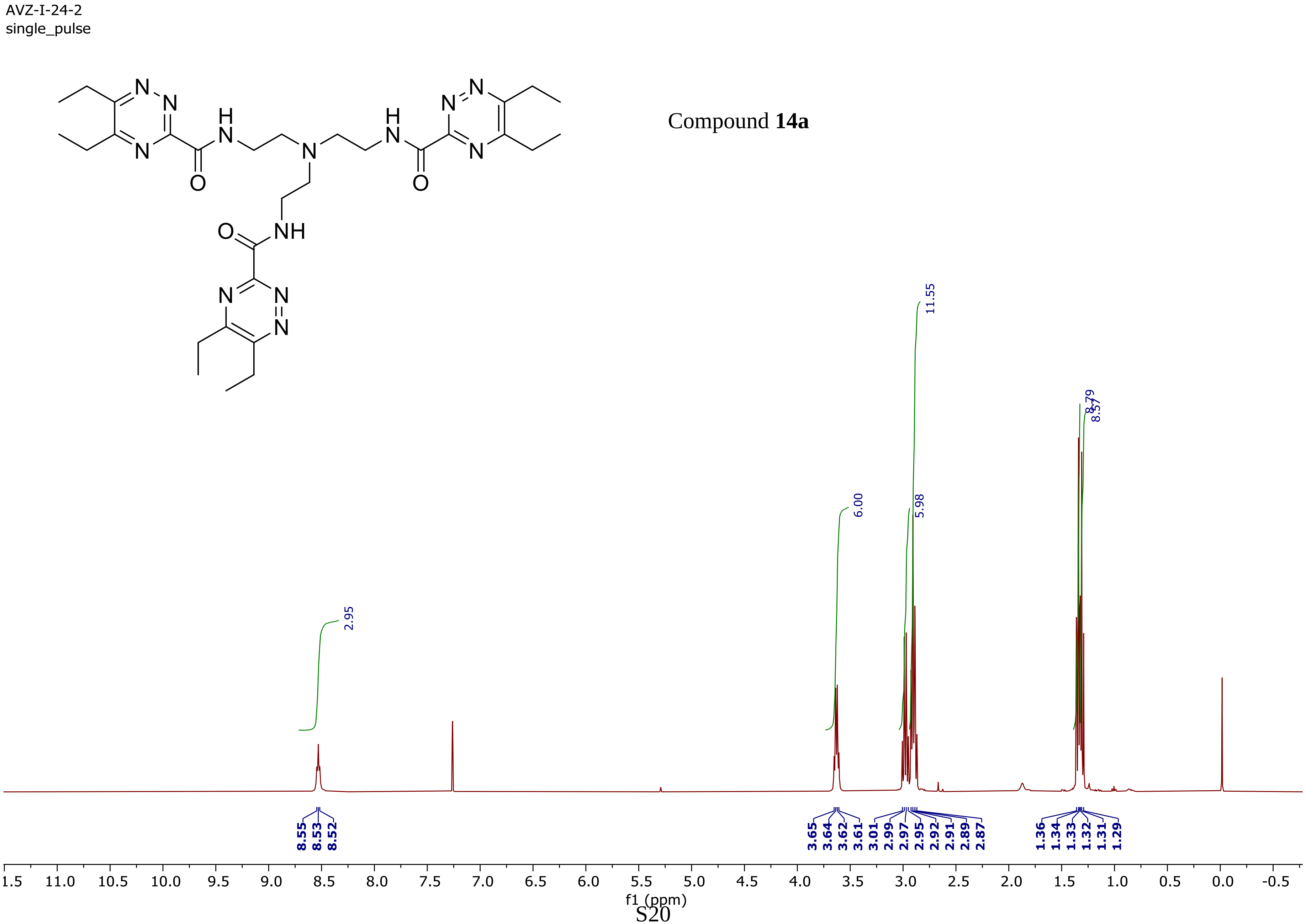

Compound **14a**

AVZ-I-24-2 1
single pulse decoupled gated NOE

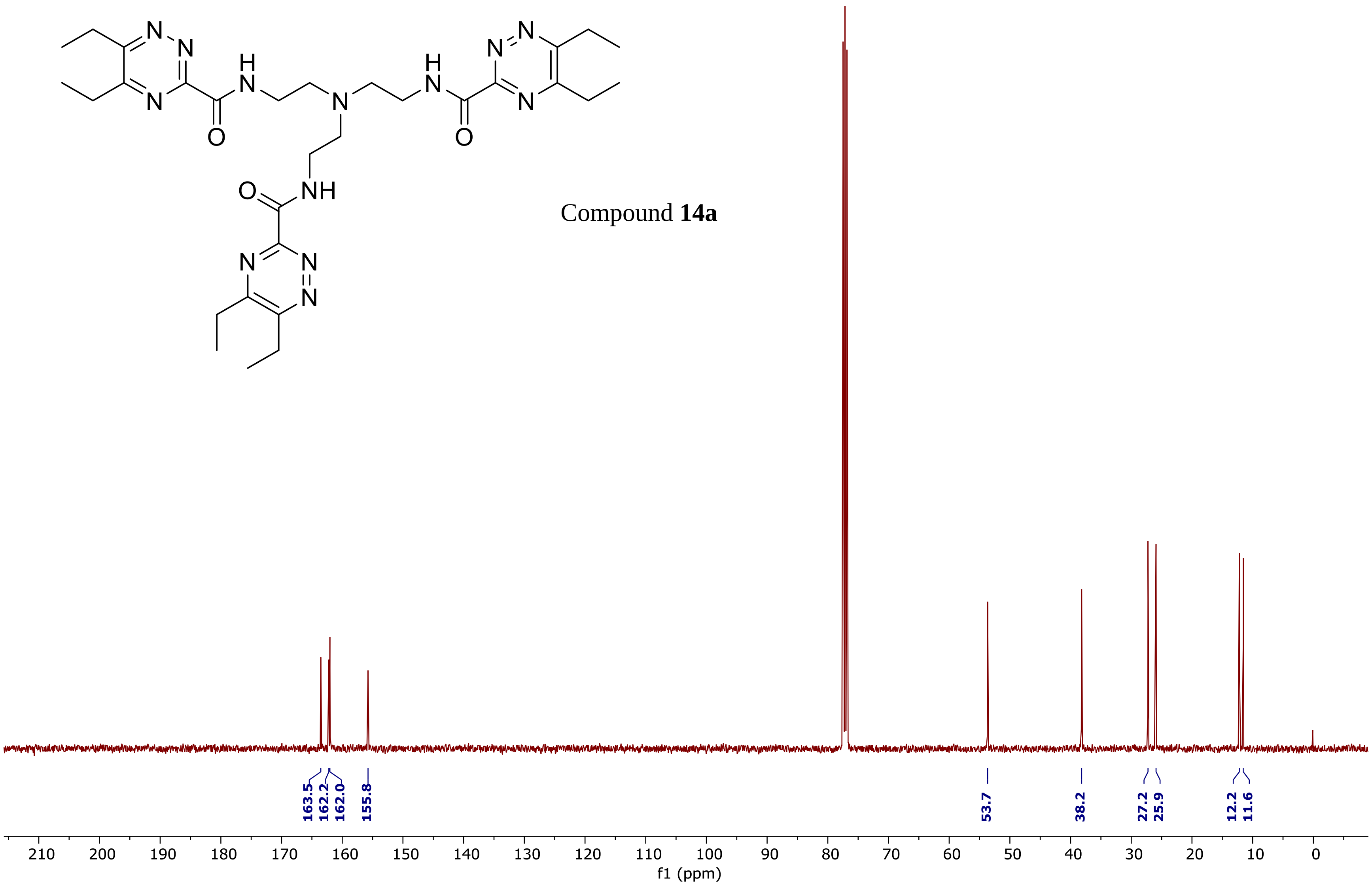

Compound **14a**

AVZ-I-82-1
single_pulse

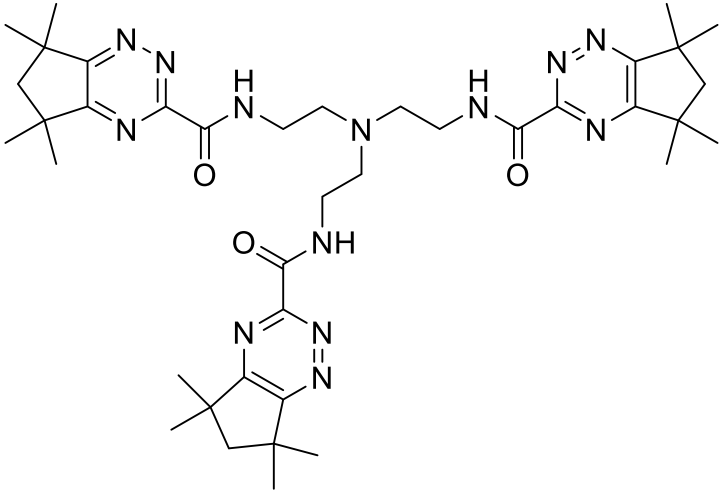

Compound **14b**

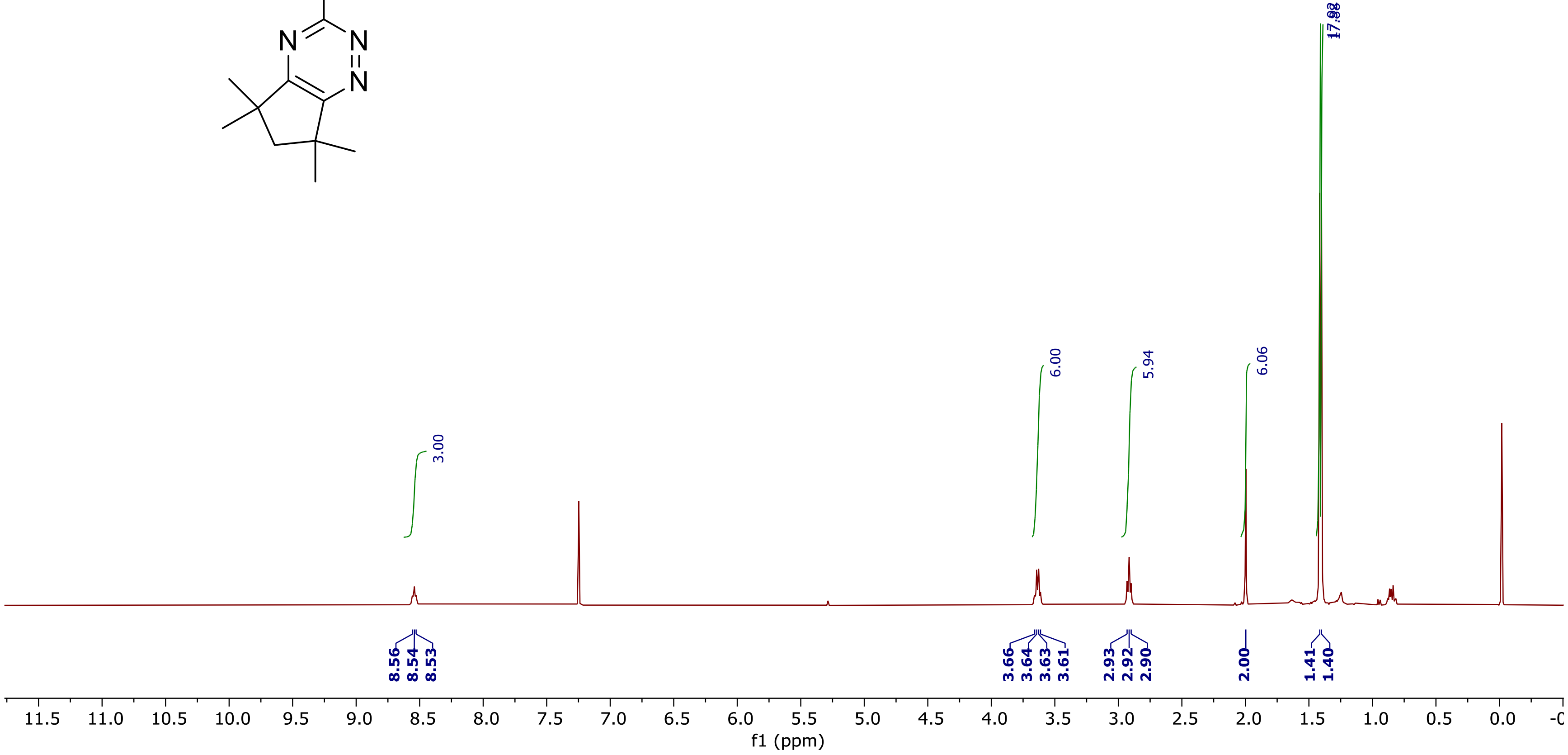

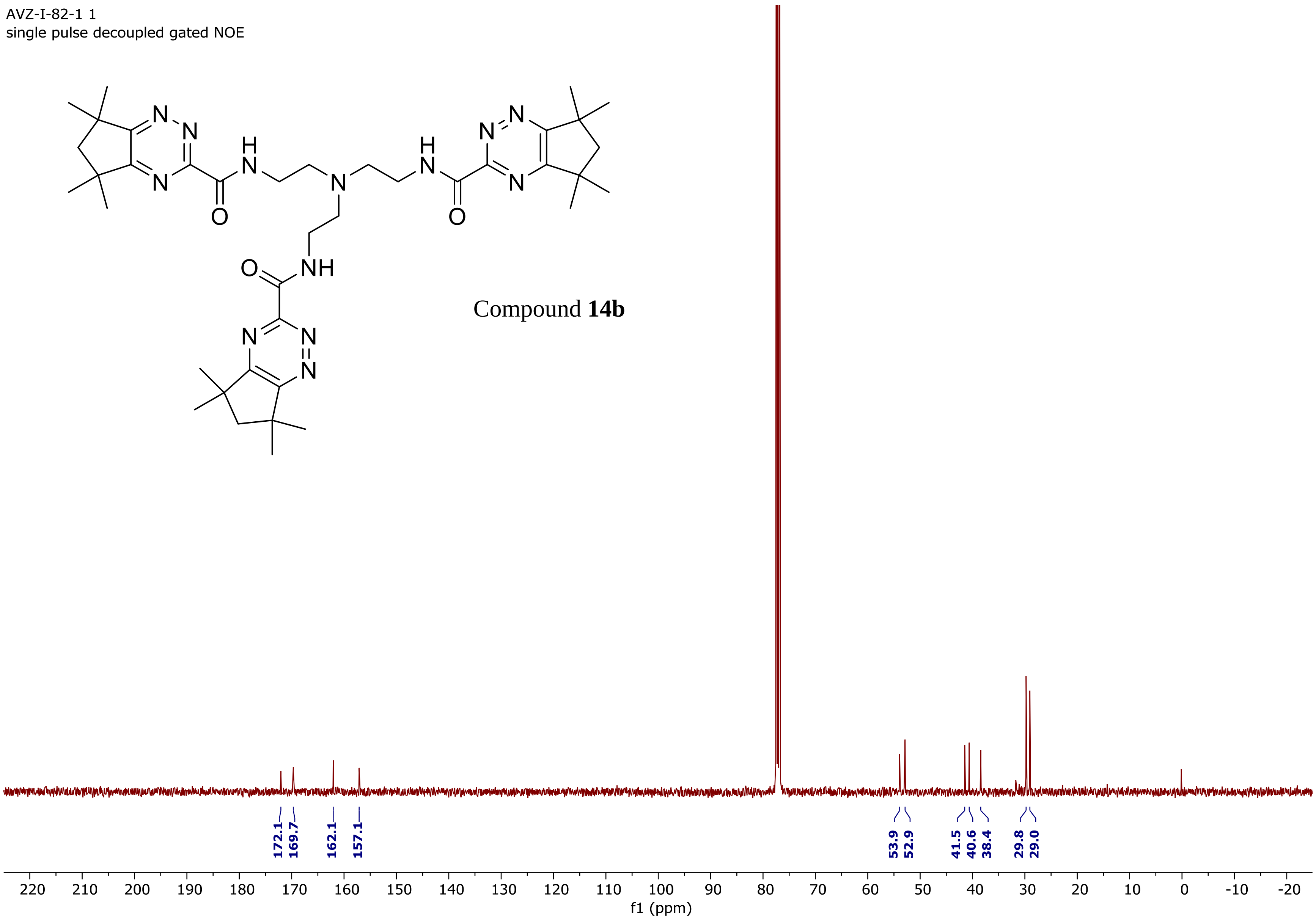
AVZ-I-82-1 1
single pulse decoupled gated NOE
Compound **14b**
172.1
169.7
162.1
157.1
53.9
52.9
41.5
40.6
38.4
29.8
29.0
220 210 200 190 180 170 160 150 140 130 120 110 100 90 80 70 60 50 40 30 20 10 0 -10 -20
f1 (ppm)

AVZ-I-90-1
single_pulse

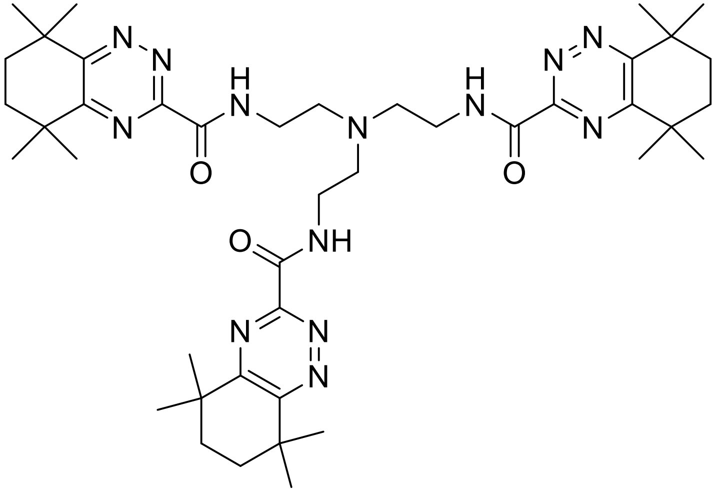

Compound **14c**

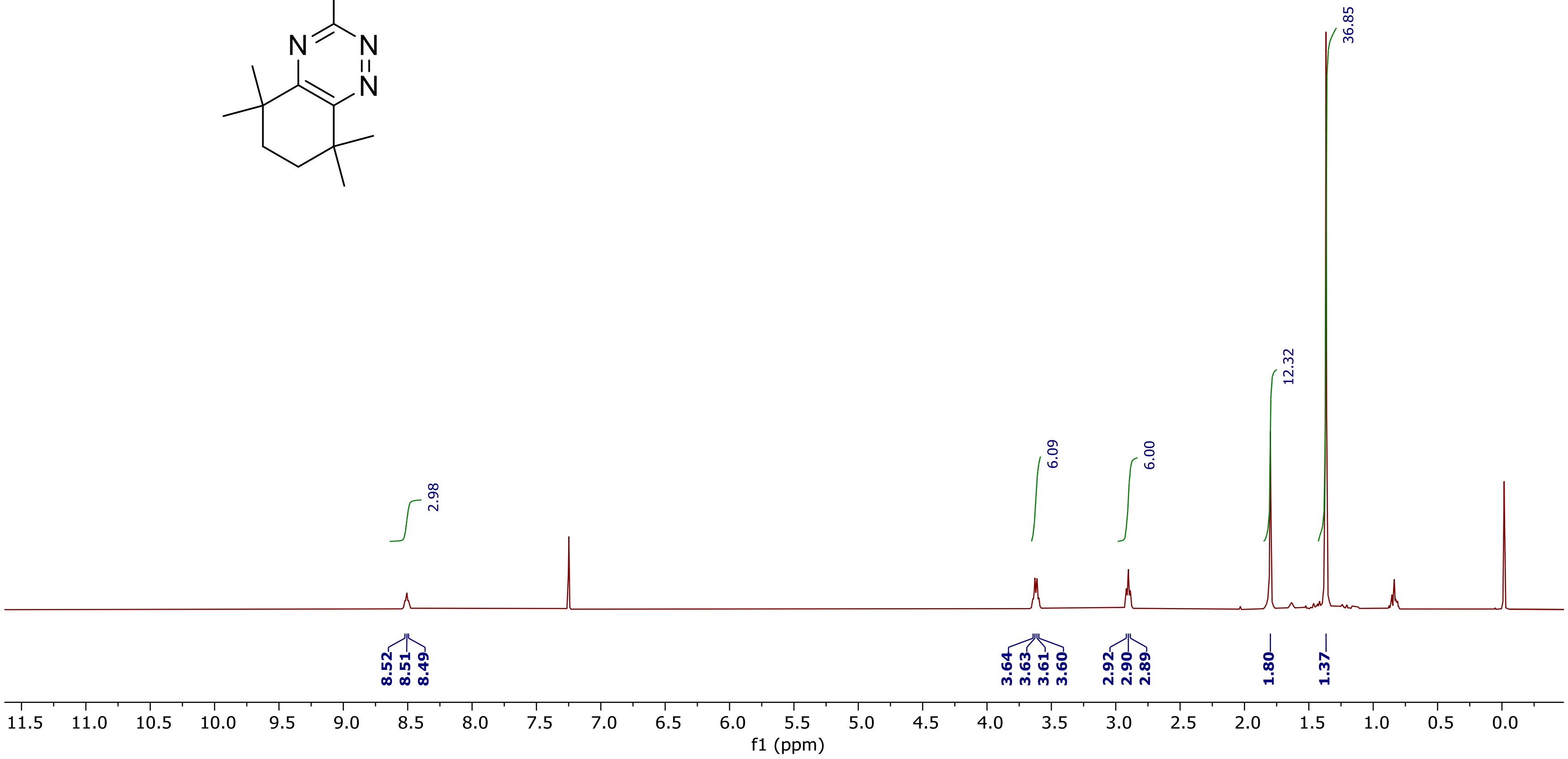

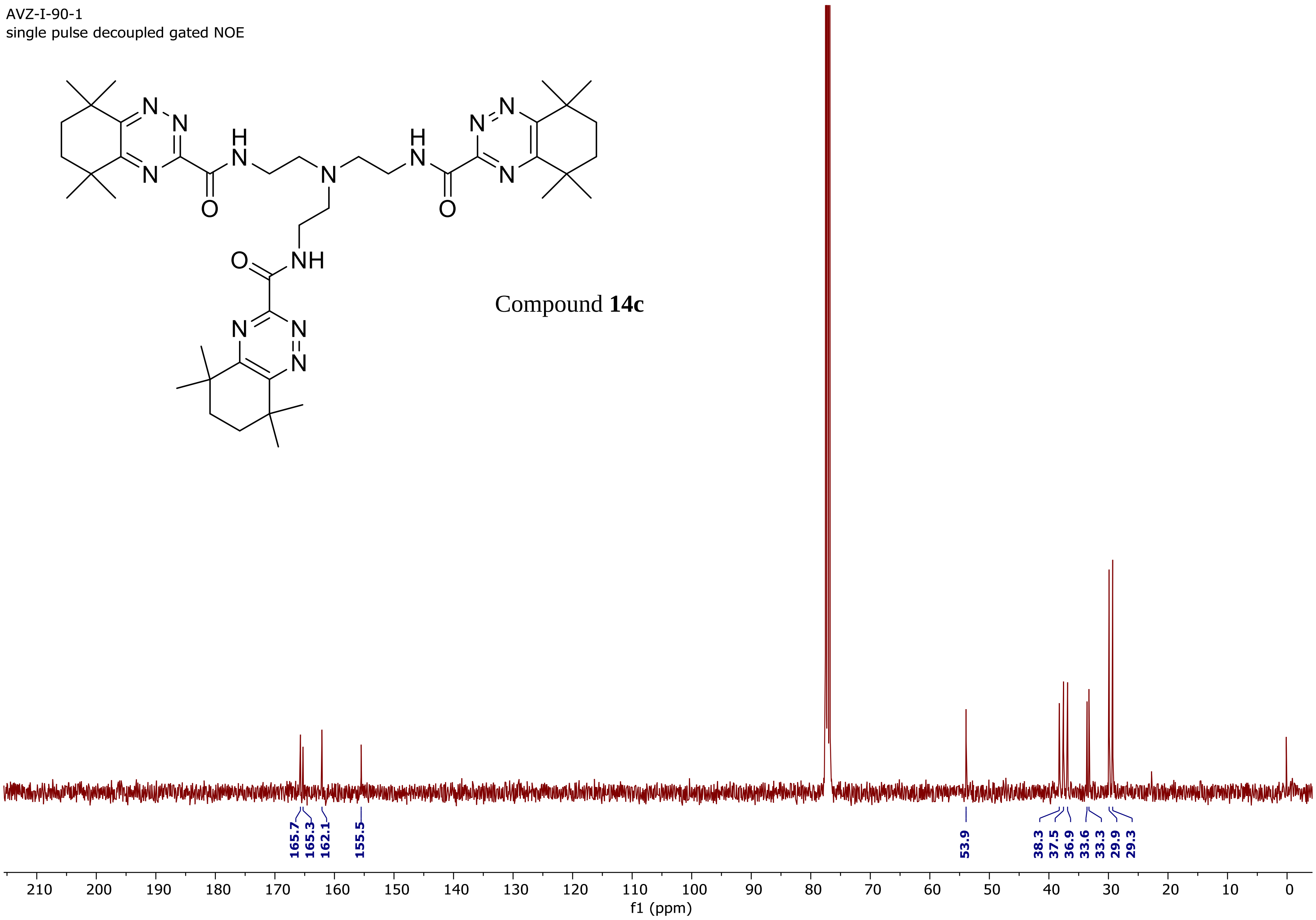

Compound **14c**

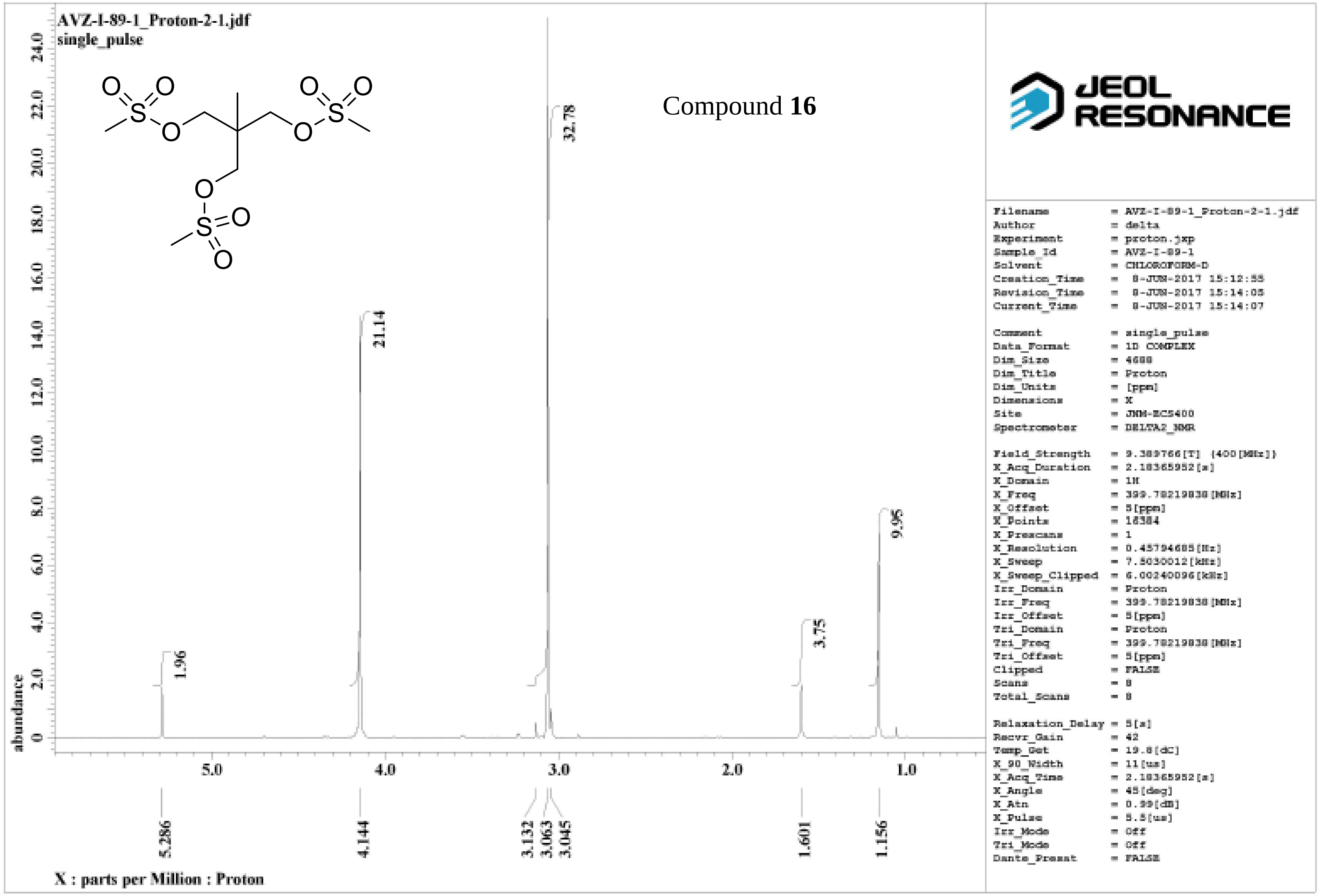
AVZ-I-89-1_Proton-2-1.jdf
single_pulse
Compound 16
abundance
X : parts per Million : Proton
1.96
21.14
32.78
3.75
9.95
5.286
4.144
3.132
3.063
3.045
1.601
1.156
Filename = AVZ-I-89-1_Proton-2-1.jdf
Author = delta
Experiment = proton.jxp
Sample_Id = AVZ-I-89-1
Solvent = CHLOROFORM-D
Creation_Time = 8-JUN-2017 15:12:55
Revision_Time = 8-JUN-2017 15:14:05
Current_Time = 8-JUN-2017 15:14:07
Comment = single_pulse
Data_Format = 1D COMPLEX
Dim_Size = 4688
Dim_Title = Proton
Dim_Units = [ppm]
Dimensions = X
Site = JNM-ECS400
Spectrometer = DELTA2_NMR
Field_Strength = 9.389766[T] (400[MHz])
X_Acq_Duration = 2.18365952[s]
X_Domain = 1H
X_Freq = 399.78219838[MHz]
X_Offset = 5[ppm]
X_Points = 16384
X_Prescans = 1
X_Resolution = 0.45794685[Hz]
X_Sweep = 7.5030012[kHz]
X_Sweep_Clipped = 6.00240096[kHz]
Irr_Domain = Proton
Irr_Freq = 399.78219838[MHz]
Irr_Offset = 5[ppm]
Tri_Domain = Proton
Tri_Freq = 399.78219838[MHz]
Tri_Offset = 5[ppm]
Clipped = FALSE
Scans = 8
Total_Scans = 8
Relaxation_Delay = 5[s]
Recvr_Gain = 42
Temp_Get = 19.8[dC]
X_90_Width = 11[us]
X_Acq_Time = 2.18365952[s]
X_Angle = 45[deg]
X_Atn = 0.99[dB]
X_Pulse = 5.5[us]
Irr_Mode = Off
Tri_Mode = Off
Dante_Presat = FALSE

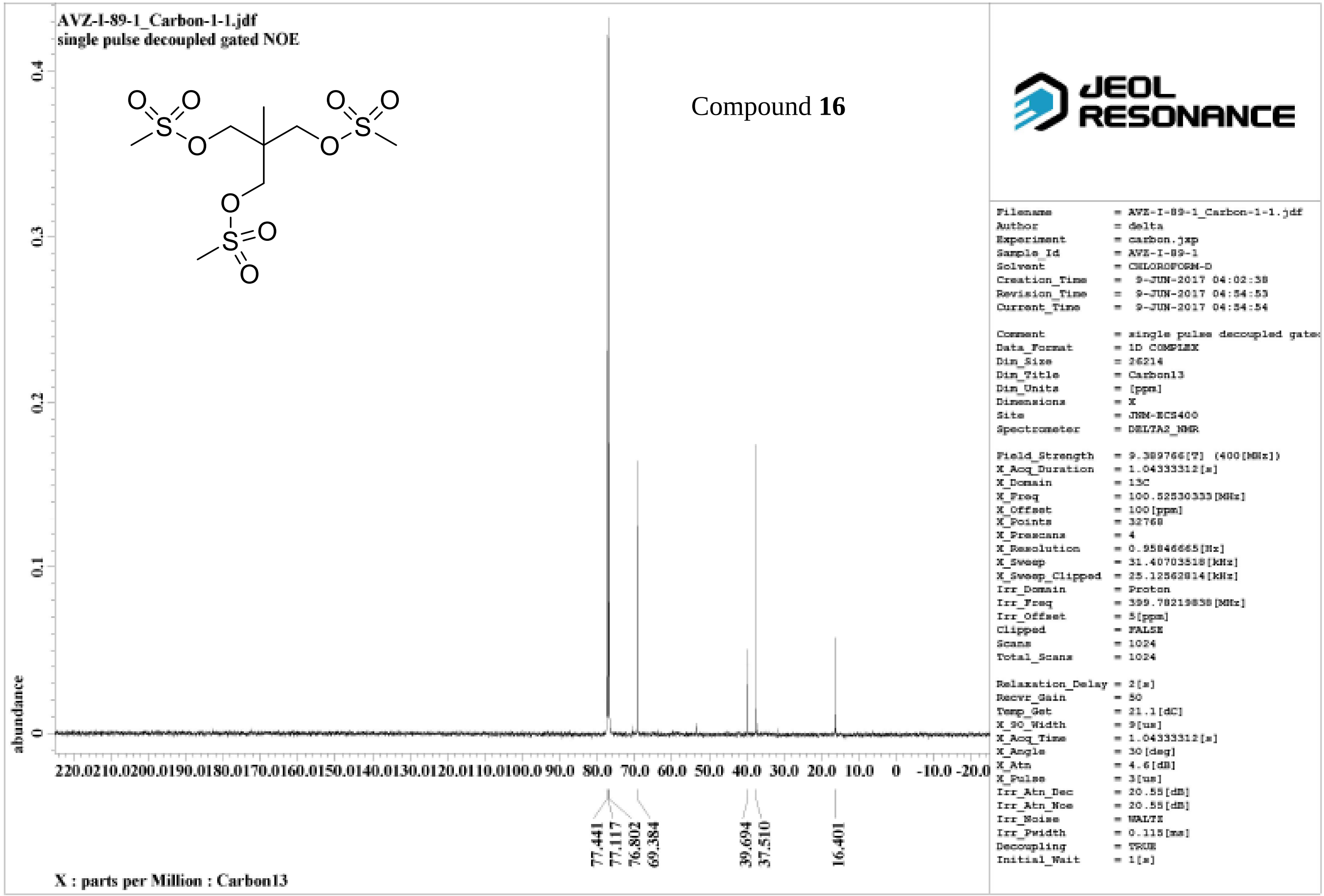
AVZ-I-89-1_Carbon-1-1.jdf
single pulse decoupled gated NOE
Compound 16
abundance
0
0.1
0.2
0.3
0.4
220.0 210.0 200.0 190.0 180.0 170.0 160.0 150.0 140.0 130.0 120.0 110.0 100.0 90.0 80.0 70.0 60.0 50.0 40.0 30.0 20.0 10.0 0 -10.0 -20.0
77.441
77.117
76.802
69.384
39.694
37.510
16.401
X : parts per Million : Carbon13
Filename = AVZ-I-89-1_Carbon-1-1.jdf
Author = delta
Experiment = carbon.jxp
Sample_Id = AVZ-I-89-1
Solvent = CHLOROFORM-D
Creation_Time = 9-JUN-2017 04:02:38
Revision_Time = 9-JUN-2017 04:54:53
Current_Time = 9-JUN-2017 04:54:54
Comment = single pulse decoupled gate
Data_Format = 1D COMPLEX
Dim_Size = 26214
Dim_Title = Carbon13
Dim_Units = [ppm]
Dimensions = X
Site = JNM-ECS400
Spectrometer = DELTA2_NMR
Field_Strength = 9.389766[T] (400[MHz])
X_Acq_Duration = 1.04333312[s]
X_Domain = 13C
X_Freq = 100.52530333[MHz]
X_Offset = 100[ppm]
X_Points = 32768
X_Prescans = 4
X_Resolution = 0.95846665[Hz]
X_Sweep = 31.40703518[kHz]
X_Sweep_Clipped = 25.12562814[kHz]
Irr_Domain = Proton
Irr_Freq = 399.78219838[MHz]
Irr_Offset = 5[ppm]
Clipped = FALSE
Scans = 1024
Total_Scans = 1024
Relaxation_Delay = 2[s]
Recvr_Gain = 50
Temp_Get = 21.1[dC]
X_90_Width = 9[us]
X_Acq_Time = 1.04333312[s]
X_Angle = 30[deg]
X_Atn = 4.6[dB]
X_Pulse = 3[us]
Irr_Atn_Dec = 20.55[dB]
Irr_Atn_Noe = 20.55[dB]
Irr_Noise = WALTZ
Irr_Pwidth = 0.115[ms]
Decoupling = TRUE
Initial_Wait = 1[s]

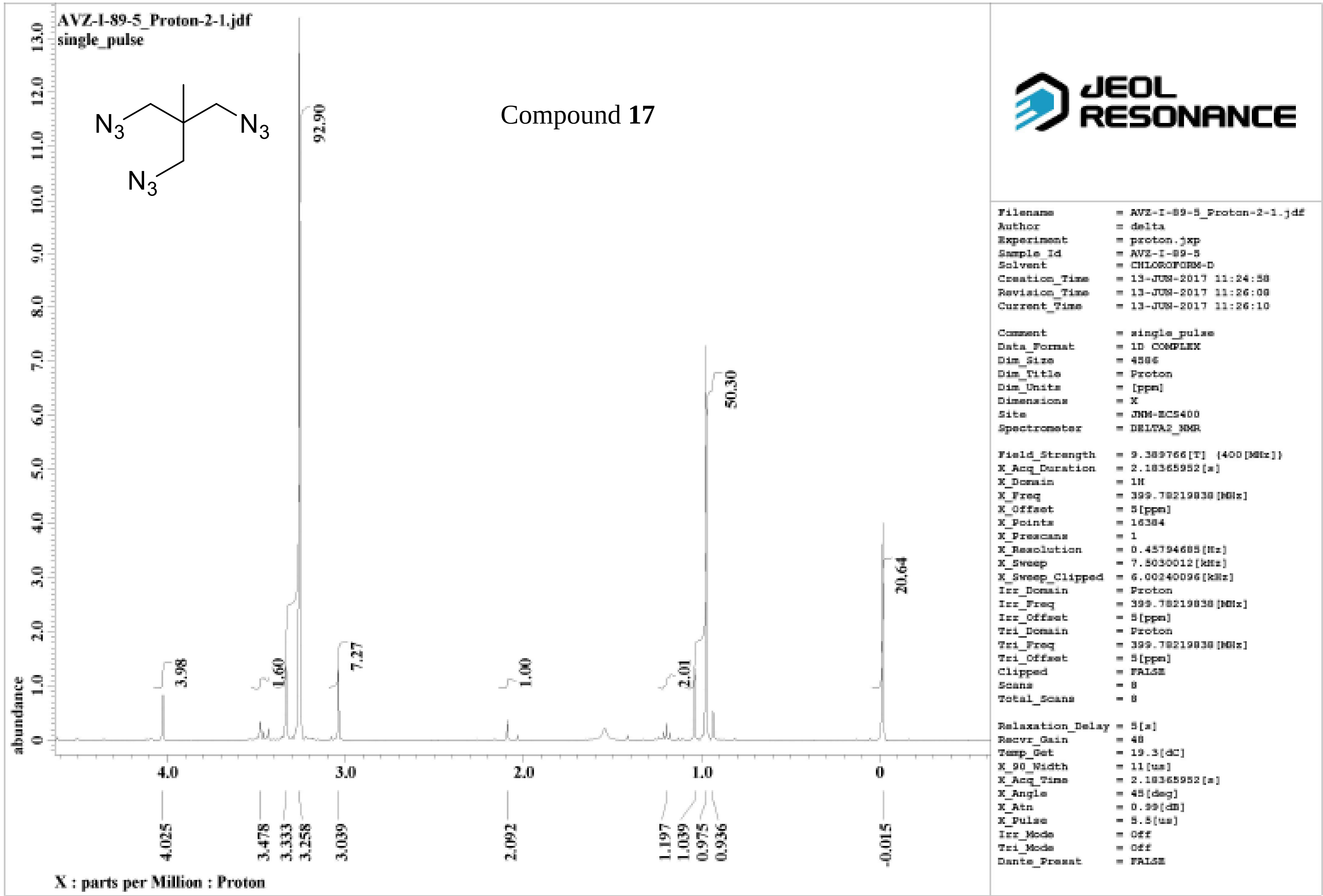

AVZ-I-89-5_Proton-2-1.jdf
single_pulse
Compound 17
N3
N3
N3
92.90
50.30
20.64
3.98
1.60
7.27
1.00
2.01
abundance
X : parts per Million : Proton
4.025
3.478
3.333
3.258
3.039
2.092
1.197
1.039
0.975
0.936
-0.015
JEOL RESONANCE
Filename = AVZ-I-89-5_Proton-2-1.jdf
Author = delta
Experiment = proton.jxp
Sample_Id = AVZ-I-89-5
Solvent = CHLOROFORM-D
Creation_Time = 13-JUN-2017 11:24:58
Revision_Time = 13-JUN-2017 11:26:08
Current_Time = 13-JUN-2017 11:26:10
Comment = single_pulse
Data_Format = 1D COMPLEX
Dim_Size = 4586
Dim_Title = Proton
Dim_Units = [ppm]
Dimensions = X
Site = JNM-ECS400
Spectrometer = DELTA2_NMR
Field_Strength = 9.389766[T] (400[MHz])
X_Acq_Duration = 2.18365952[s]
X_Domain = 1H
X_Freq = 399.78219838[MHz]
X_Offset = 5[ppm]
X_Points = 16384
X_Prescans = 1
X_Resolution = 0.45794685[Hz]
X_Sweep = 7.5030012[kHz]
X_Sweep_Clipped = 6.00240096[kHz]
Irr_Domain = Proton
Irr_Freq = 399.78219838[MHz]
Irr_Offset = 5[ppm]
Tri_Domain = Proton
Tri_Freq = 399.78219838[MHz]
Tri_Offset = 5[ppm]
Clipped = FALSE
Scans = 8
Total_Scans = 8
Relaxation_Delay = 5[s]
Recvr_Gain = 48
Temp_Get = 19.3[dC]
X_90_Width = 11[us]
X_Acq_Time = 2.18365952[s]
X_Angle = 45[deg]
X_Atn = 0.99[dB]
X_Pulse = 5.5[us]
Irr_Mode = Off
Tri_Mode = Off
Dante_Presat = FALSE

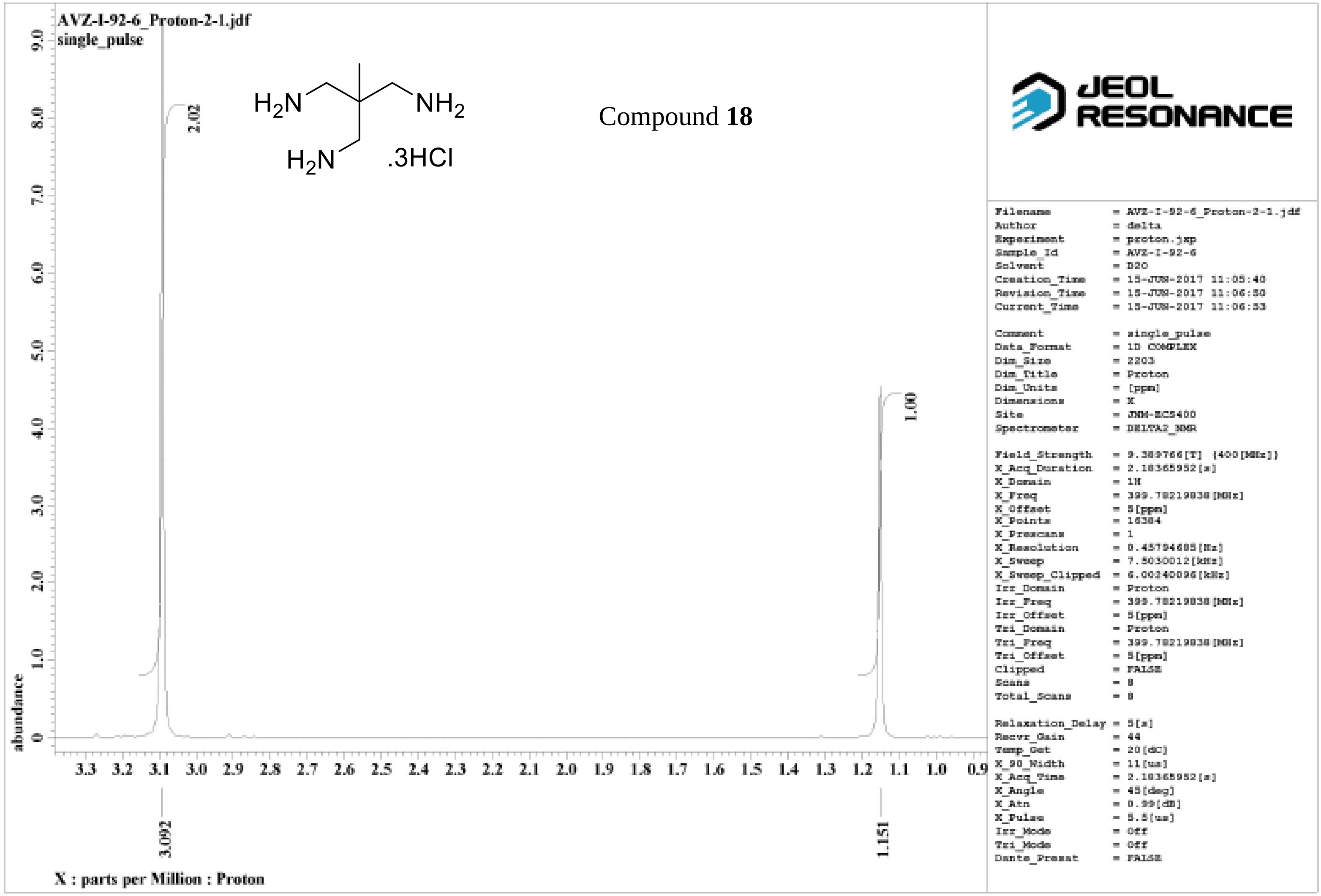

AVZ-I-92-6_Proton-2-1.jdf
single_pulse
H_2N
NH_2
H_2N
.3HCl
Compound **18**
2.02
1.00
abundance
9.0
8.0
7.0
6.0
5.0
4.0
3.0
2.0
1.0
0
3.3 3.2 3.1 3.0 2.9 2.8 2.7 2.6 2.5 2.4 2.3 2.2 2.1 2.0 1.9 1.8 1.7 1.6 1.5 1.4 1.3 1.2 1.1 1.0 0.9
3.092
1.151
X : parts per Million : Proton
JEOL RESONANCE
Filename = AVZ-I-92-6_Proton-2-1.jdf
Author = delta
Experiment = proton.jxp
Sample_Id = AVZ-I-92-6
Solvent = D2O
Creation_Time = 15-JUN-2017 11:05:40
Revision_Time = 15-JUN-2017 11:06:50
Current_Time = 15-JUN-2017 11:06:53
Comment = single_pulse
Data_Format = 1D COMPLEX
Dim_Size = 2203
Dim_Title = Proton
Dim_Units = [ppm]
Dimensions = X
Site = JNM-ECS400
Spectrometer = DELTA2_NMR
Field_Strength = 9.389766[T] (400[MHz])
X_Acq_Duration = 2.18365952[s]
X_Domain = 1H
X_Freq = 399.78219838[MHz]
X_Offset = 5[ppm]
X_Points = 16384
X_Prescans = 1
X_Resolution = 0.45794685[Hz]
X_Sweep = 7.5030012[kHz]
X_Sweep_Clipped = 6.00240096[kHz]
Irr_Domain = Proton
Irr_Freq = 399.78219838[MHz]
Irr_Offset = 5[ppm]
Tri_Domain = Proton
Tri_Freq = 399.78219838[MHz]
Tri_Offset = 5[ppm]
Clipped = FALSE
Scans = 8
Total_Scans = 8
Relaxation_Delay = 5[s]
Recvr_Gain = 44
Temp_Get = 20[dC]
X_90_Width = 11[us]
X_Acq_Time = 2.18365952[s]
X_Angle = 45[deg]
X_Atn = 0.99[dB]
X_Pulse = 5.5[us]
Irr_Mode = Off
Tri_Mode = Off
Dante_Presat = FALSE

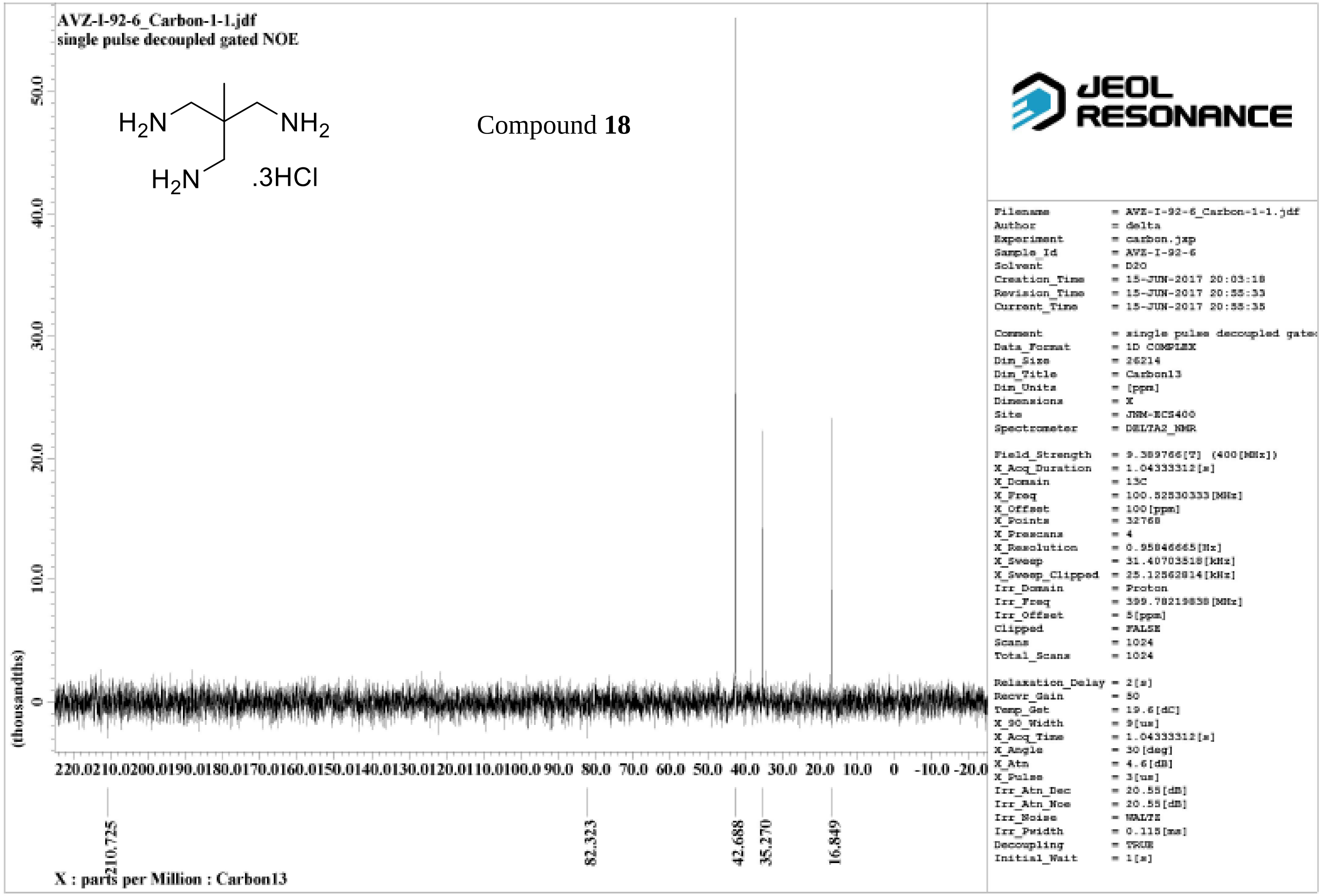
AVZ-I-92-6_Carbon-1-1.jdf
single pulse decoupled gated NOE
H2N
NH2
H2N
.3HCl
Compound 18
50.0
40.0
30.0
20.0
10.0
0
(thousandths)
220.0210.0200.0190.0180.0170.0160.0150.0140.0130.0120.0110.0100.0 90.0 80.0 70.0 60.0 50.0 40.0 30.0 20.0 10.0 0 -10.0 -20.0
210.725
82.323
42.688
35.270
16.849
X : parts per Million : Carbon13
JEOL RESONANCE
Filename = AVZ-I-92-6_Carbon-1-1.jdf
Author = delta
Experiment = carbon.jxp
Sample_Id = AVZ-I-92-6
Solvent = D2O
Creation_Time = 15-JUN-2017 20:03:18
Revision_Time = 15-JUN-2017 20:55:33
Current_Time = 15-JUN-2017 20:55:35
Comment = single pulse decoupled gate
Data_Format = 1D COMPLEX
Dim_Size = 26214
Dim_Title = Carbon13
Dim_Units = [ppm]
Dimensions = X
Site = JNM-ECS400
Spectrometer = DELTA2_NMR
Field_Strength = 9.389766[T] (400[MHz])
X_Acq_Duration = 1.04333312[s]
X_Domain = 13C
X_Freq = 100.52530333[MHz]
X_Offset = 100[ppm]
X_Points = 32768
X_Prescans = 4
X_Resolution = 0.95846665[Hz]
X_Sweep = 31.40703518[kHz]
X_Sweep_Clipped = 25.12562814[kHz]
Irr_Domain = Proton
Irr_Freq = 399.78219838[MHz]
Irr_Offset = 5[ppm]
Clipped = FALSE
Scans = 1024
Total_Scans = 1024
Relaxation_Delay = 2[s]
Recvr_Gain = 50
Temp_Get = 19.6[dC]
X_90_Width = 9[us]
X_Acq_Time = 1.04333312[s]
X_Angle = 30[deg]
X_Atn = 4.6[dB]
X_Pulse = 3[us]
Irr_Atn_Dec = 20.55[dB]
Irr_Atn_Noe = 20.55[dB]
Irr_Noise = WALTZ
Irr_Pwidth = 0.115[ms]
Decoupling = TRUE
Initial_Wait = 1[s]

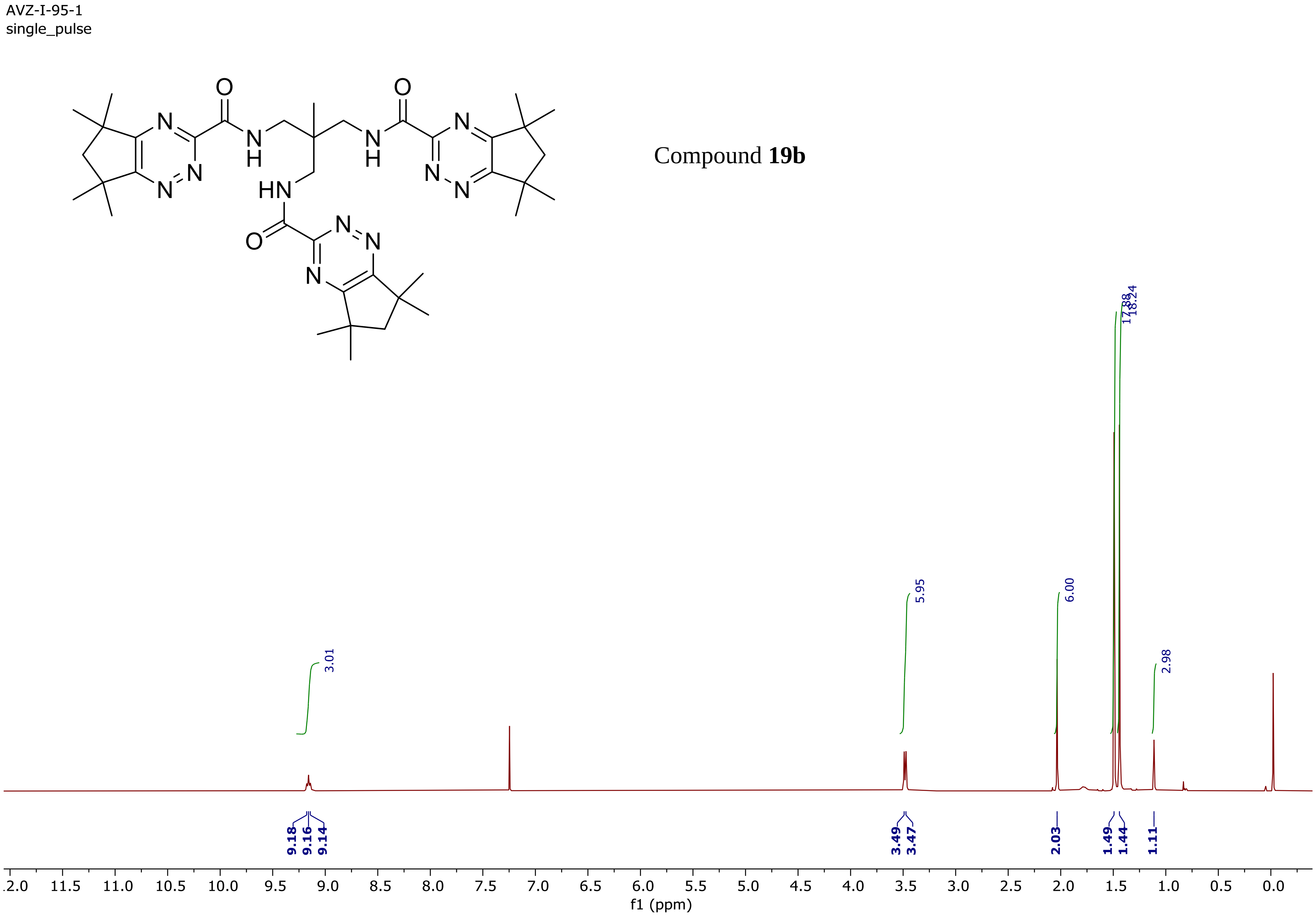

Compound **19b**

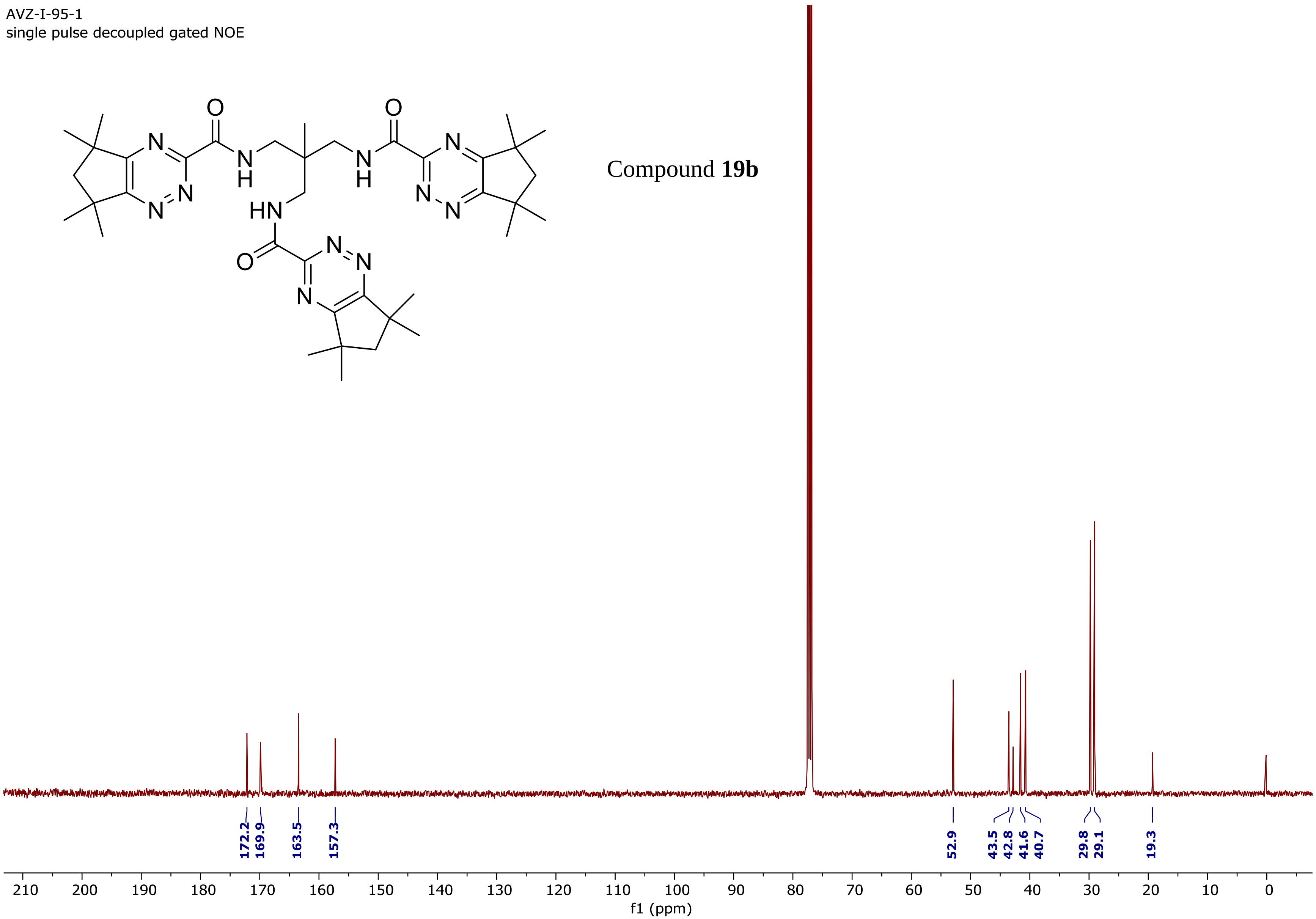

Compound **19b**

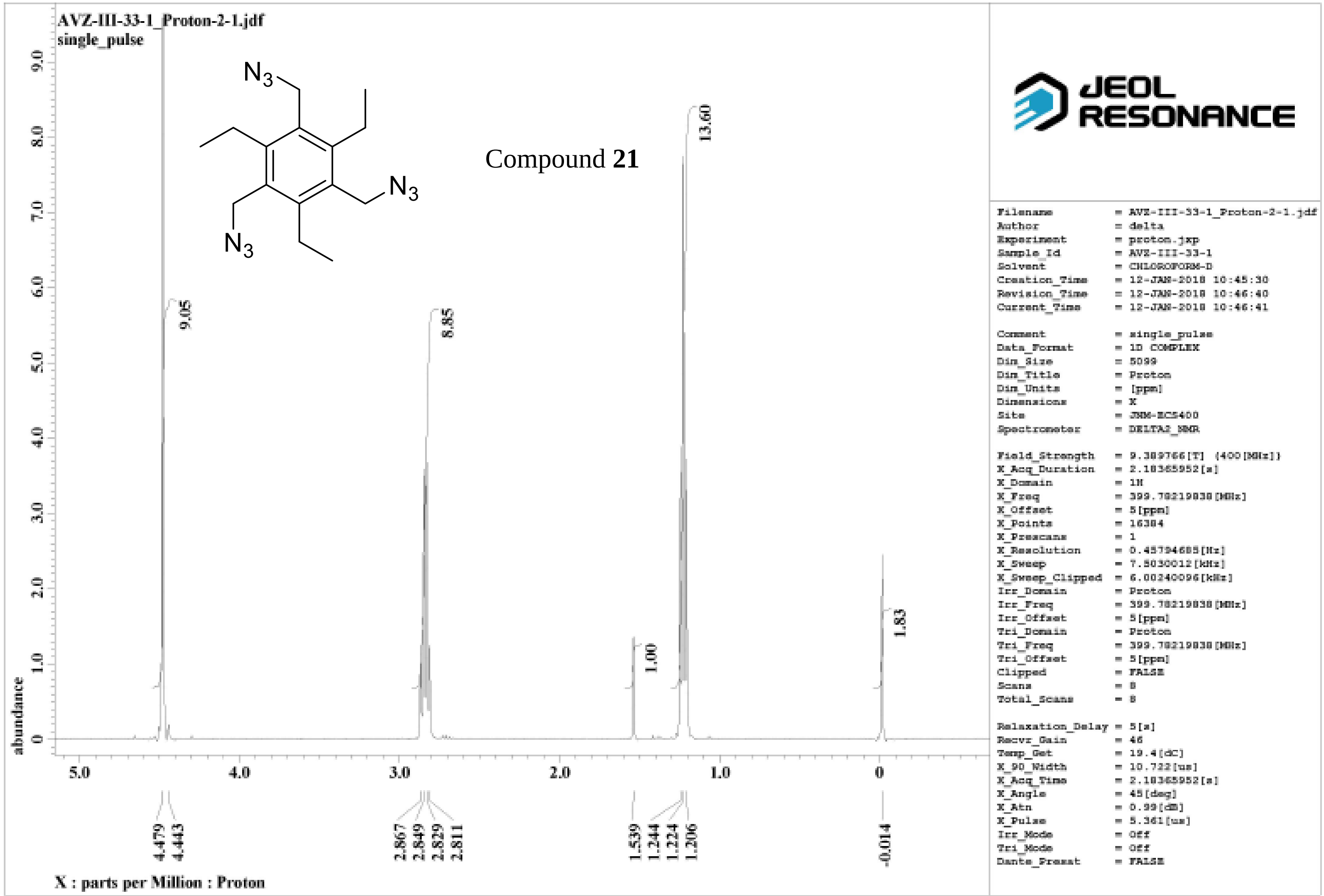

AVZ-III-33-1_Proton-2-1.jdf
single_pulse
N3
N3
N3
Compound 21
9.05
8.85
1.00
13.60
1.83
abundance
9.0
8.0
7.0
6.0
5.0
4.0
3.0
2.0
1.0
0
5.0
4.0
3.0
2.0
1.0
0
4.479
4.443
2.867
2.849
2.829
2.811
1.539
1.244
1.224
1.206
-0.014
X : parts per Million : Proton
JEOL RESONANCE
Filename = AVZ-III-33-1_Proton-2-1.jdf
Author = delta
Experiment = proton.jxp
Sample_Id = AVZ-III-33-1
Solvent = CHLOROFORM-D
Creation_Time = 12-JAN-2018 10:45:30
Revision_Time = 12-JAN-2018 10:46:40
Current_Time = 12-JAN-2018 10:46:41
Comment = single_pulse
Data_Format = 1D COMPLEX
Dim_Size = 5099
Dim_Title = Proton
Dim_Units = [ppm]
Dimensions = X
Site = JNM-ECS400
Spectrometer = DELTA2_NMR
Field_Strength = 9.389766[T] (400[MHz])
X_Acq_Duration = 2.18365952[s]
X_Domain = 1H
X_Freq = 399.78219838[MHz]
X_Offset = 5[ppm]
X_Points = 16384
X_Prescans = 1
X_Resolution = 0.45794685[Hz]
X_Sweep = 7.5030012[kHz]
X_Sweep_Clipped = 6.00240096[kHz]
Irr_Domain = Proton
Irr_Freq = 399.78219838[MHz]
Irr_Offset = 5[ppm]
Tri_Domain = Proton
Tri_Freq = 399.78219838[MHz]
Tri_Offset = 5[ppm]
Clipped = FALSE
Scans = 8
Total_Scans = 8
Relaxation_Delay = 5[s]
Recvr_Gain = 46
Temp_Get = 19.4[dC]
X_90_Width = 10.722[us]
X_Acq_Time = 2.18365952[s]
X_Angle = 45[deg]
X_Atn = 0.99[dB]
X_Pulse = 5.361[us]
Irr_Mode = Off
Tri_Mode = Off
Dante_Presat = FALSE

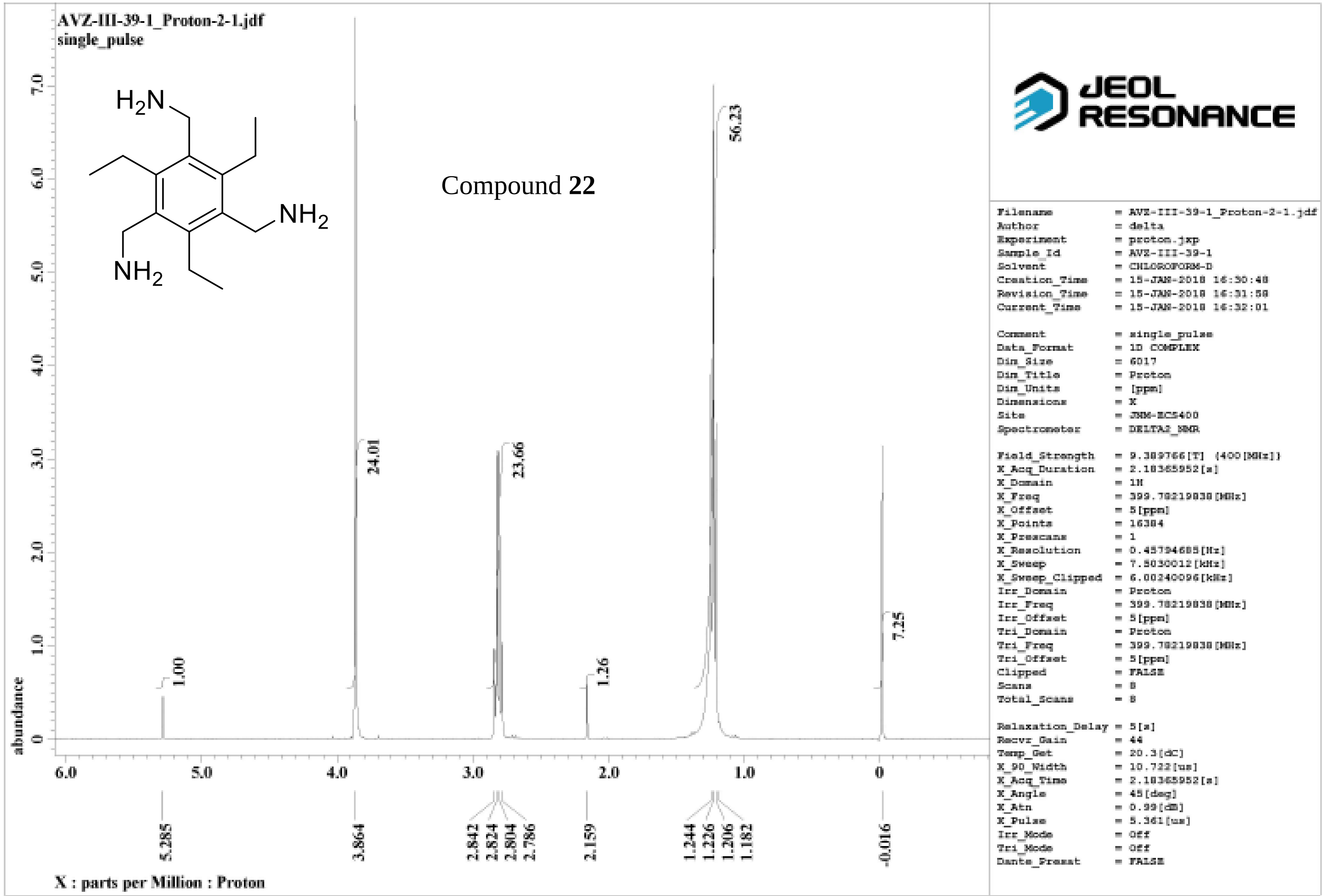

AVZ-III-39-1_Proton-2-1.jdf
single_pulse
H2N
NH2
NH2
Compound 22
24.01
23.66
56.23
1.00
1.26
7.25
abundance
7.0
6.0
5.0
4.0
3.0
2.0
1.0
0
6.0
5.0
4.0
3.0
2.0
1.0
0
5.285
3.864
2.842
2.824
2.804
2.786
2.159
1.244
1.226
1.206
1.182
-0.016
X : parts per Million : Proton
JEOL RESONANCE
Filename = AVZ-III-39-1_Proton-2-1.jdf
Author = delta
Experiment = proton.jxp
Sample_Id = AVZ-III-39-1
Solvent = CHLOROFORM-D
Creation_Time = 15-JAN-2018 16:30:48
Revision_Time = 15-JAN-2018 16:31:58
Current_Time = 15-JAN-2018 16:32:01
Comment = single_pulse
Data_Format = 1D COMPLEX
Dim_Size = 6017
Dim_Title = Proton
Dim_Units = [ppm]
Dimensions = X
Site = JNM-ECS400
Spectrometer = DELTA2_NMR
Field_Strength = 9.389766[T] (400[MHz])
X_Acq_Duration = 2.18365952[s]
X_Domain = 1H
X_Freq = 399.78219838[MHz]
X_Offset = 5[ppm]
X_Points = 16384
X_Prescans = 1
X_Resolution = 0.45794685[Hz]
X_Sweep = 7.5030012[kHz]
X_Sweep_Clipped = 6.00240096[kHz]
Irr_Domain = Proton
Irr_Freq = 399.78219838[MHz]
Irr_Offset = 5[ppm]
Tri_Domain = Proton
Tri_Freq = 399.78219838[MHz]
Tri_Offset = 5[ppm]
Clipped = FALSE
Scans = 8
Total_Scans = 8
Relaxation_Delay = 5[s]
Recvr_Gain = 44
Temp_Get = 20.3[dC]
X_90_Width = 10.722[us]
X_Acq_Time = 2.18365952[s]
X_Angle = 45[deg]
X_Atn = 0.99[dB]
X_Pulse = 5.361[us]
Irr_Mode = Off
Tri_Mode = Off
Dante_Presat = FALSE

AVZ-III-41-2
single_pulse

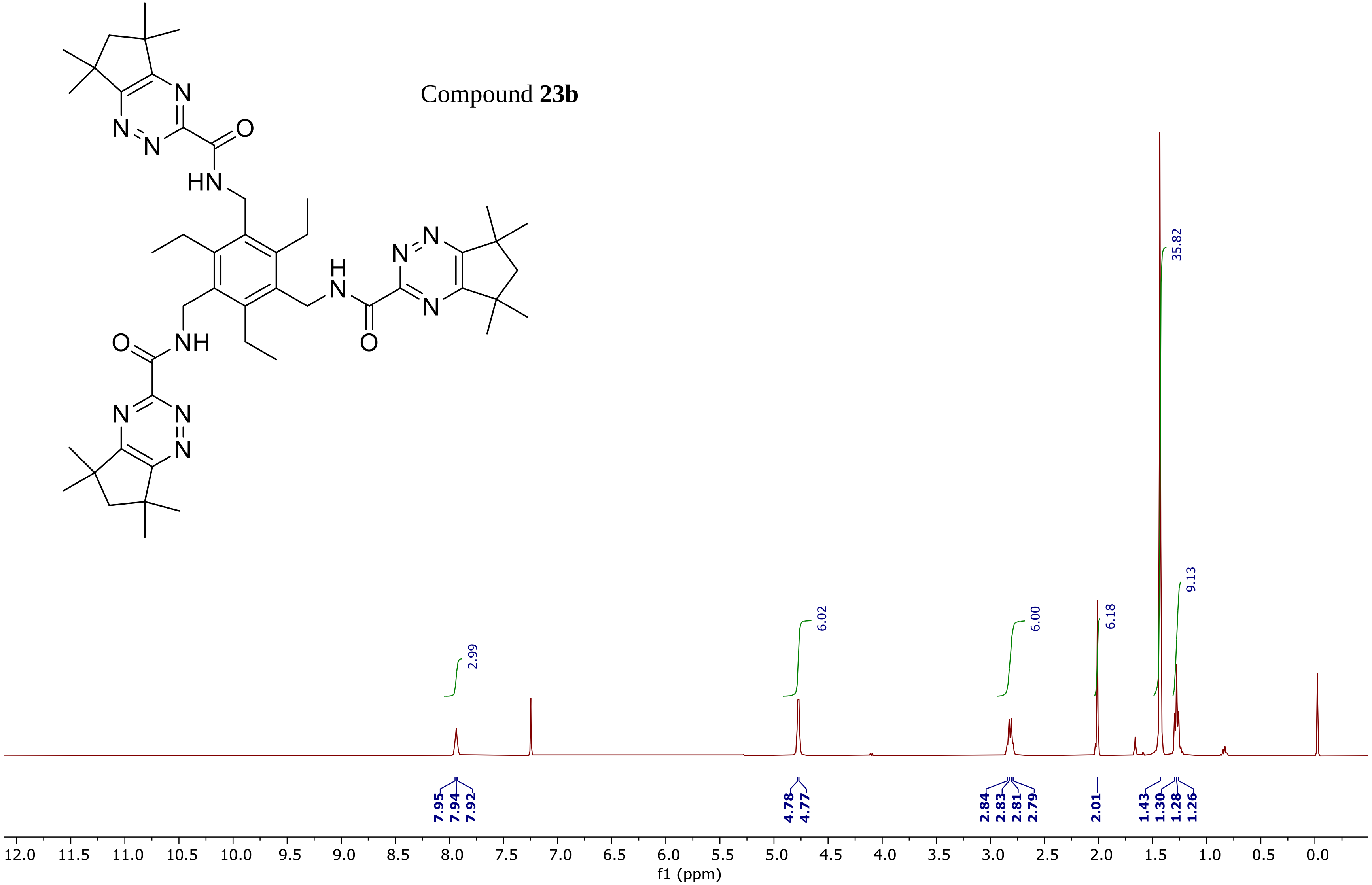

Compound **23b**

AVZ-III-41-2
single pulse decoupled gated NOE

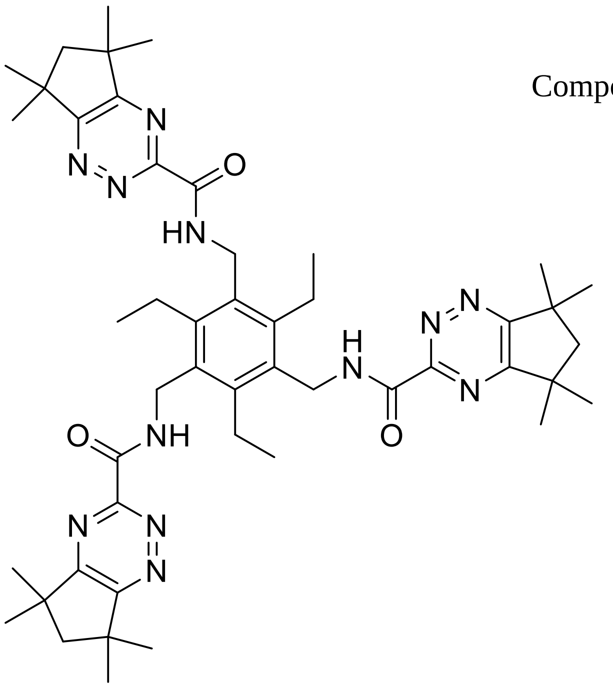

Compound **23b**

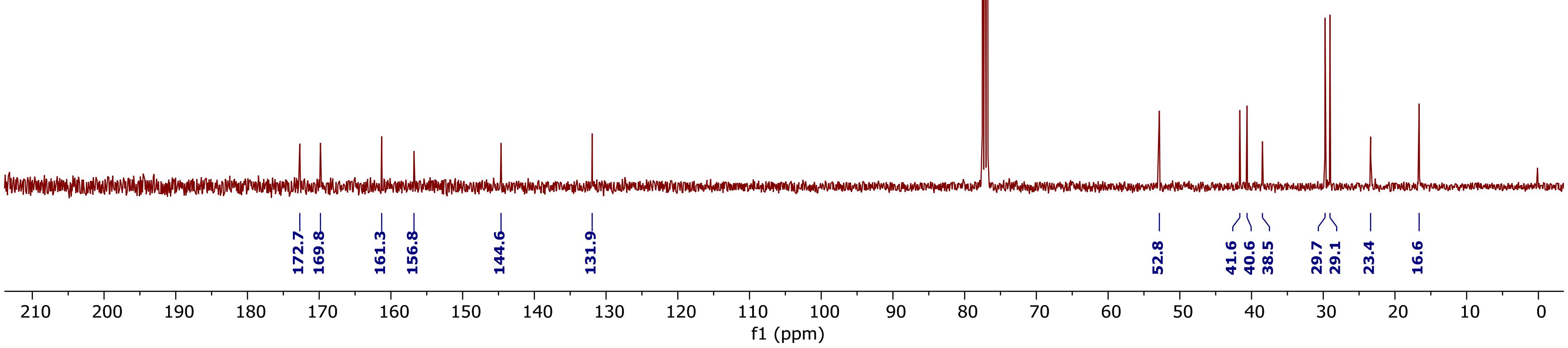

2: Mass Spectra

Compound **7a**

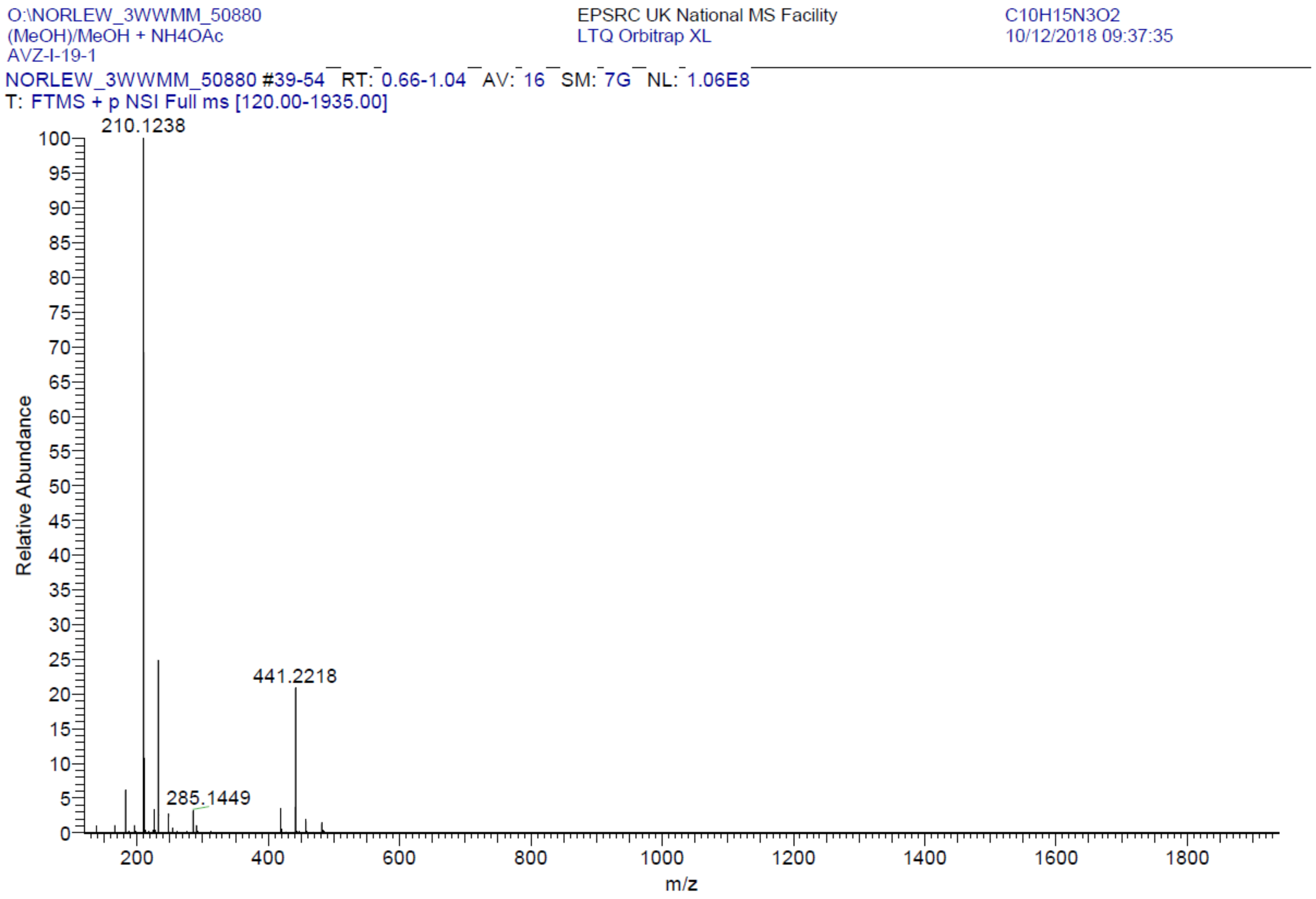

Compound **7b**

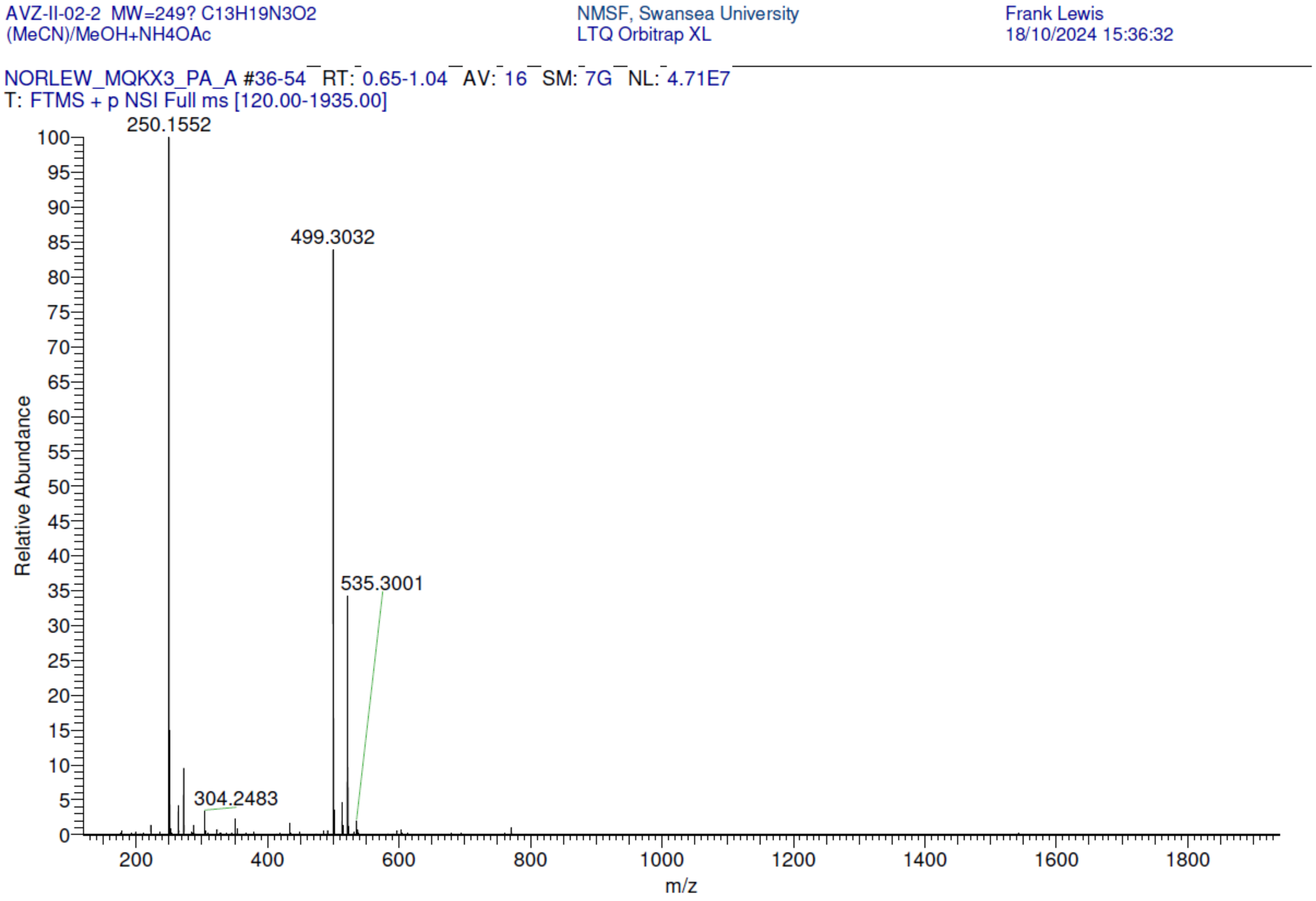
AVZ-II-02-2 MW=249? C13H19N3O2
(MeCN)/MeOH+NH4OAc
NMSF, Swansea University
LTQ Orbitrap XL
Frank Lewis
18/10/2024 15:36:32
NORLEW_MQKX3_PA_A #36-54 RT: 0.65-1.04 AV: 16 SM: 7G NL: 4.71E7
T: FTMS + p NSI Full ms [120.00-1935.00]
250.1552
499.3032
535.3001
304.2483
Relative Abundance
m/z

Compound **7c**

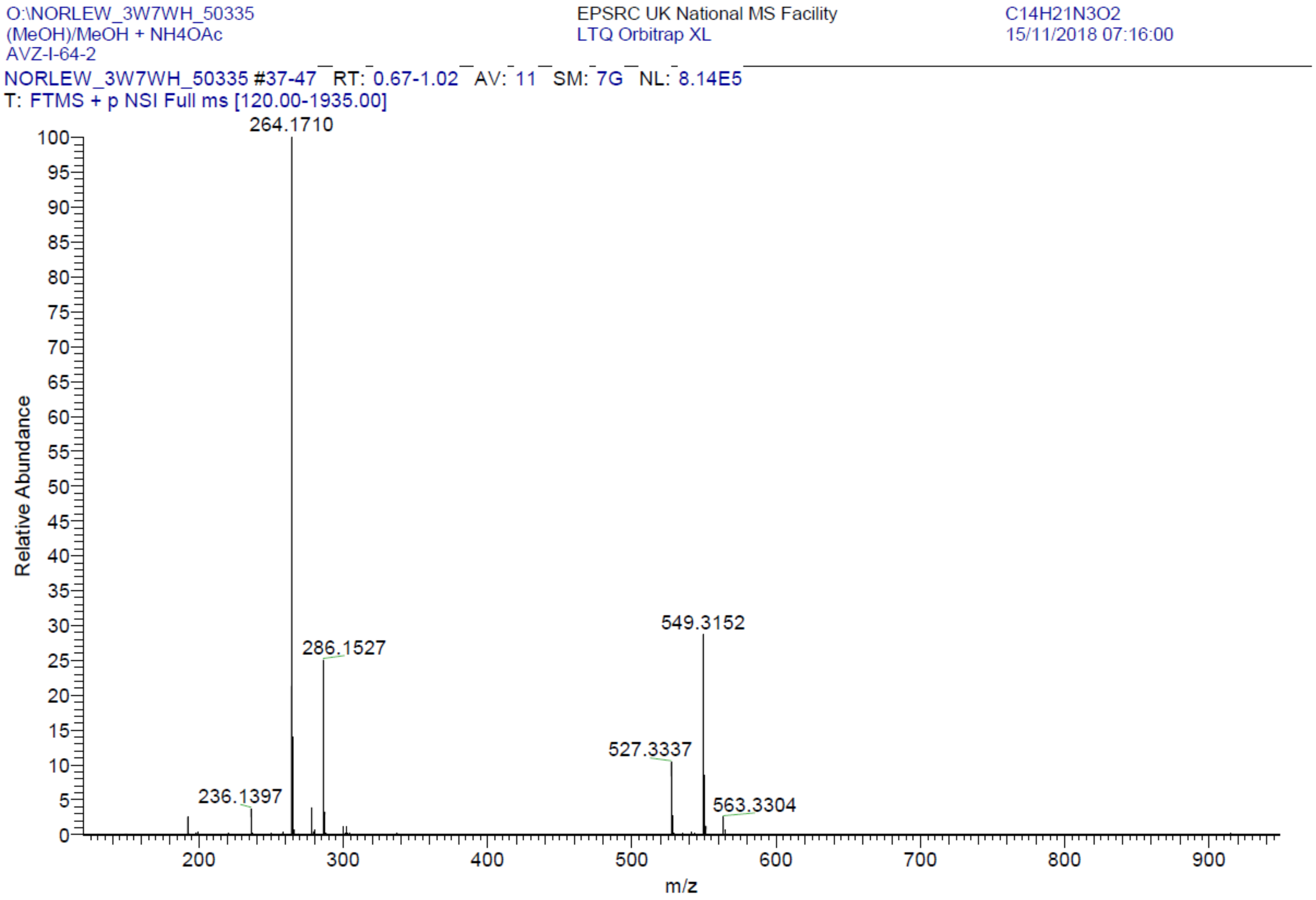
O:\NORLEW_3W7WH_50335
(MeOH)/MeOH + NH4OAc
AVZ-I-64-2
EPSRC UK National MS Facility
LTQ Orbitrap XL
C14H21N3O2
15/11/2018 07:16:00
NORLEW_3W7WH_50335 #37-47 RT: 0.67-1.02 AV: 11 SM: 7G NL: 8.14E5
T: FTMS + p NSI Full ms [120.00-1935.00]
264.1710
286.1527
236.1397
549.3152
527.3337
563.3304
Relative Abundance
m/z

Compound **10b**

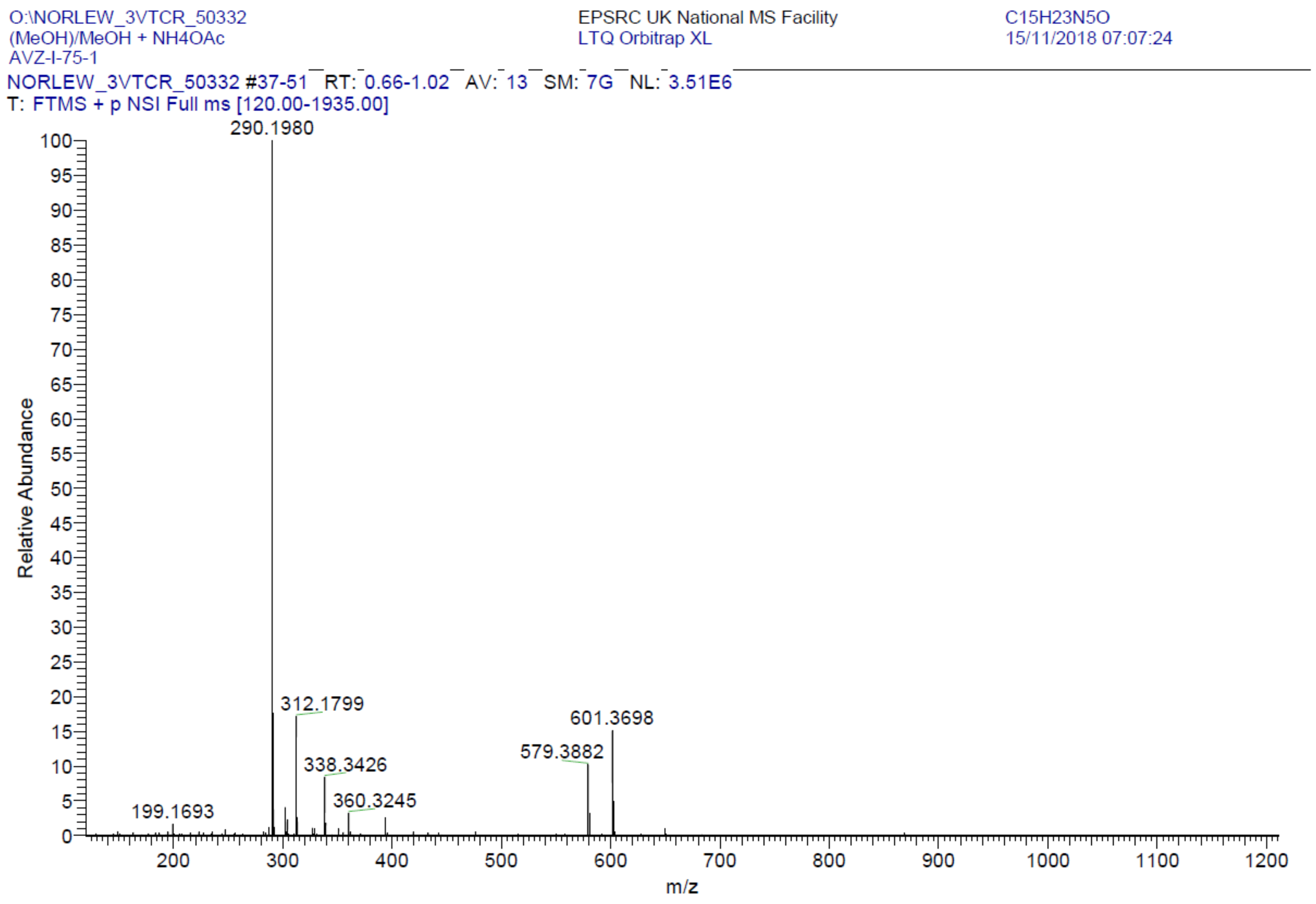

Compound **9a**

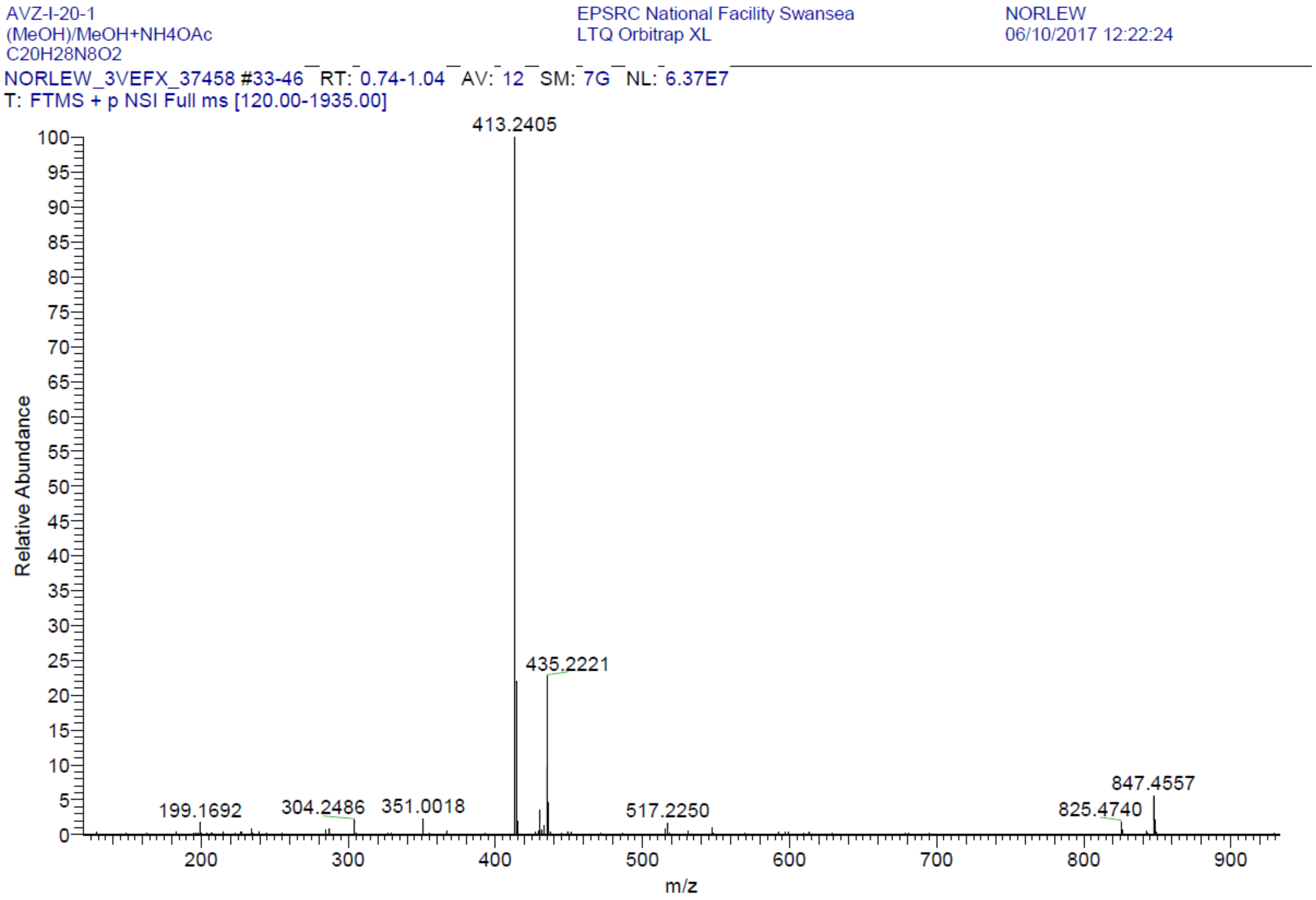

AVZ-I-20-1
(MeOH)/MeOH+NH4OAc
C20H28N8O2
EPSRC National Facility Swansea
LTQ Orbitrap XL
NORLEW
06/10/2017 12:22:24
NORLEW_3VEFX_37458 #33-46 RT: 0.74-1.04 AV: 12 SM: 7G NL: 6.37E7
T: FTMS + p NSI Full ms [120.00-1935.00]
413.2405
435.2221
199.1692
304.2486
351.0018
517.2250
825.4740
847.4557
Relative Abundance
m/z

Compound **9b**

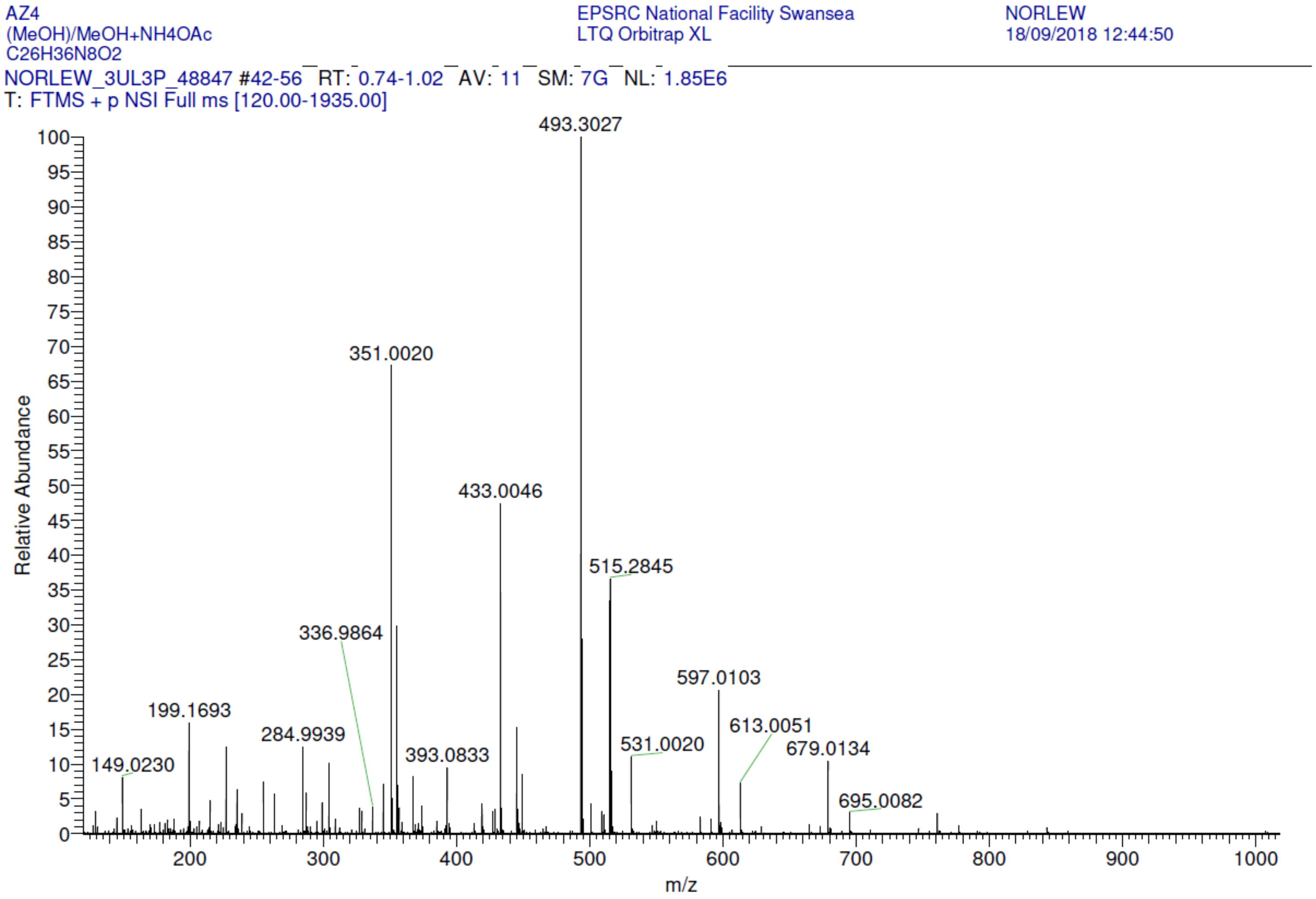
AZ4
(MeOH)/MeOH+NH4OAc
C26H36N8O2
EPSRC National Facility Swansea
LTQ Orbitrap XL
NORLEW
18/09/2018 12:44:50
NORLEW_3UL3P_48847 #42-56 RT: 0.74-1.02 AV: 11 SM: 7G NL: 1.85E6
T: FTMS + p NSI Full ms [120.00-1935.00]
493.3027
351.0020
433.0046
515.2845
336.9864
597.0103
199.1693
613.0051
284.9939
531.0020
679.0134
393.0833
149.0230
695.0082
Relative Abundance
m/z

Compound **12b**

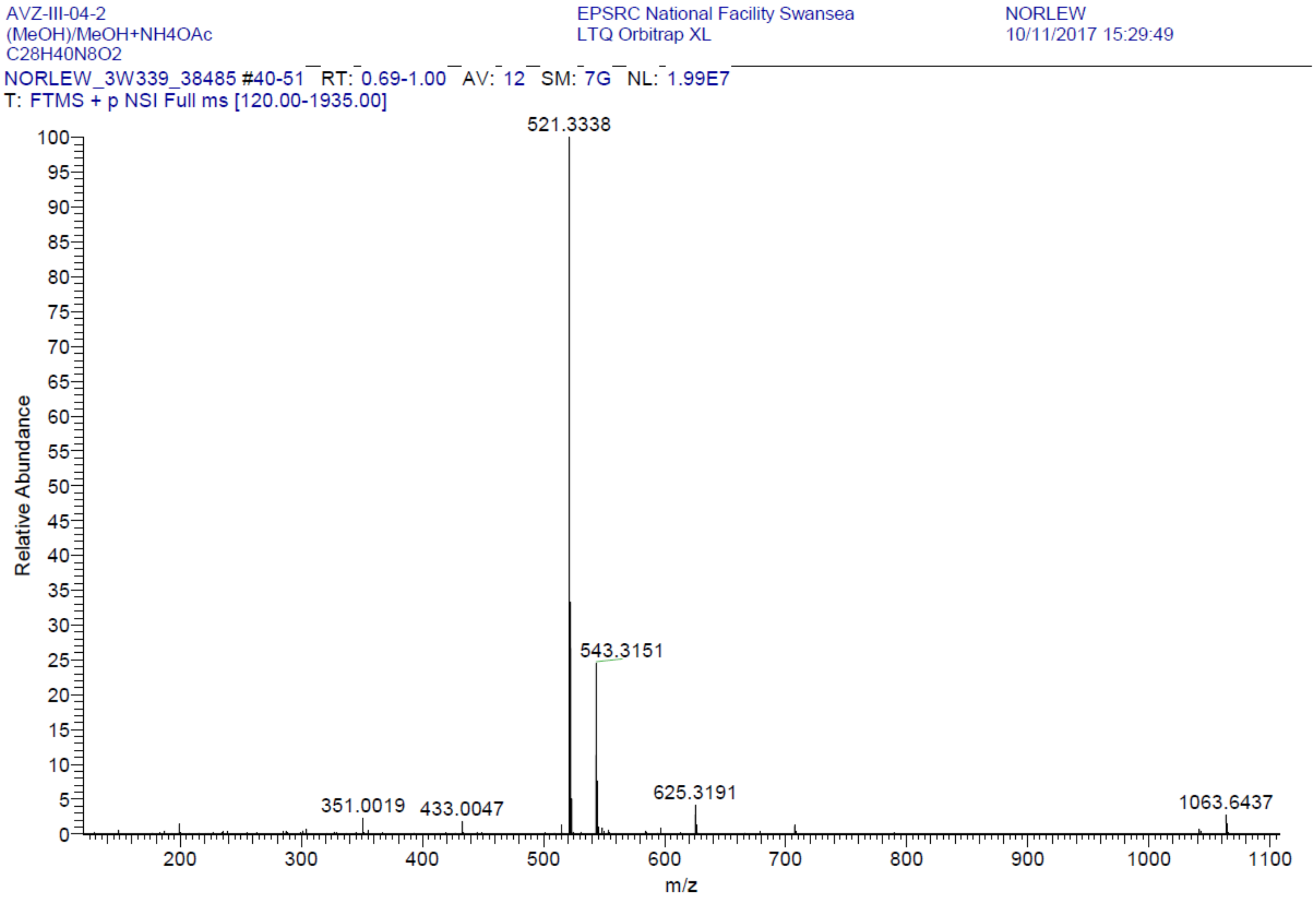

Compound **12c**

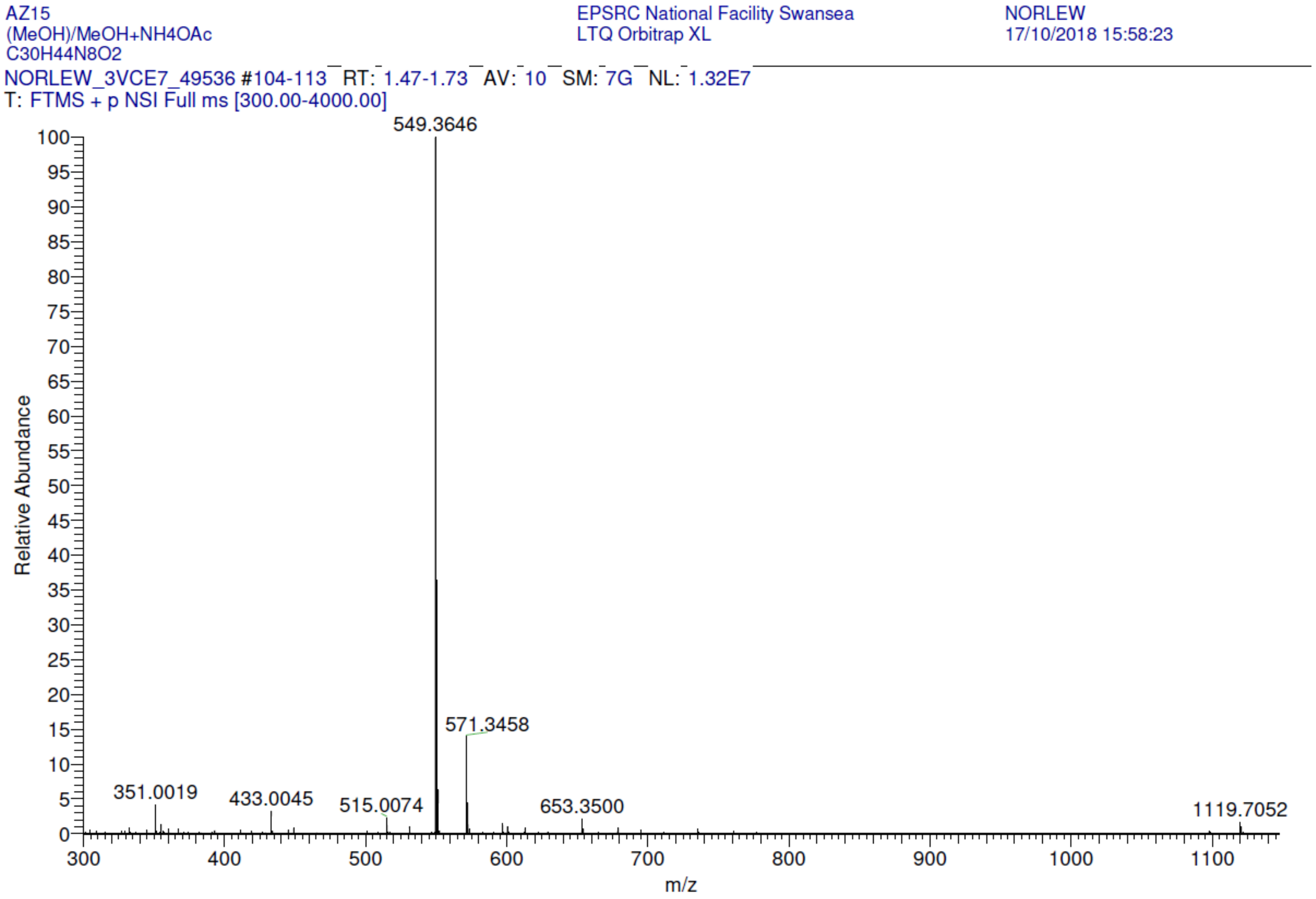

Compound **14a**

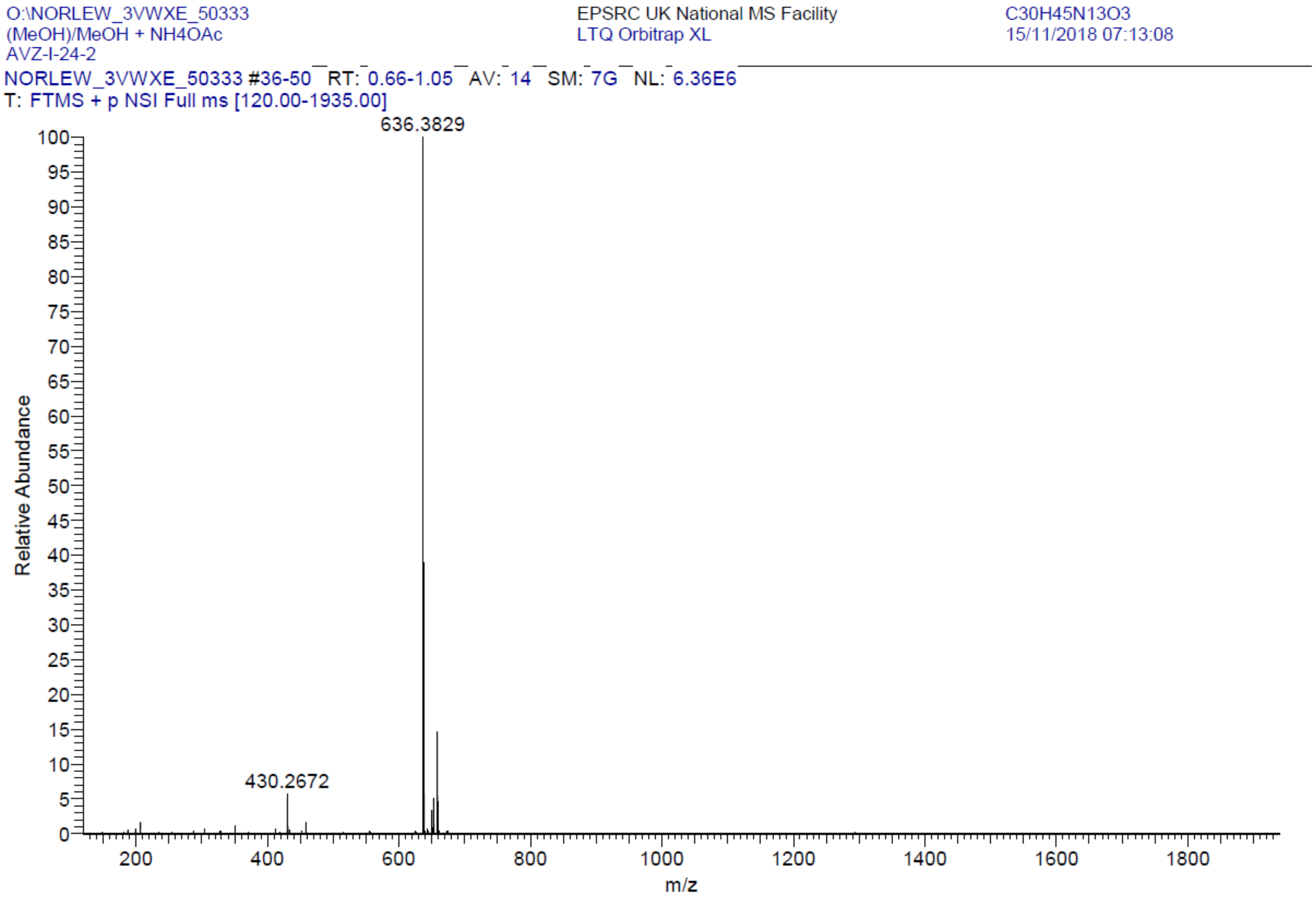

Compound **14b**

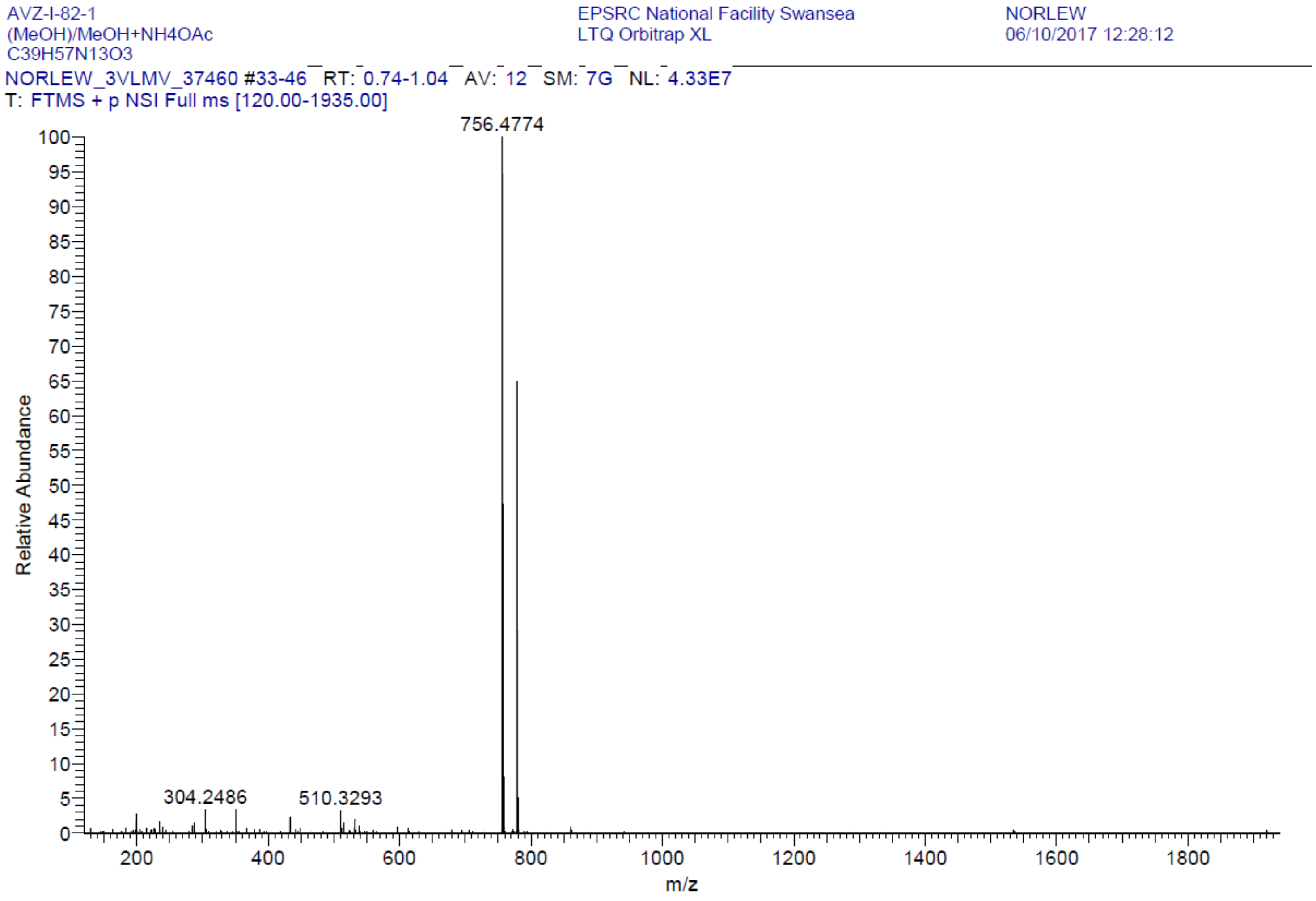

Compound **14c**

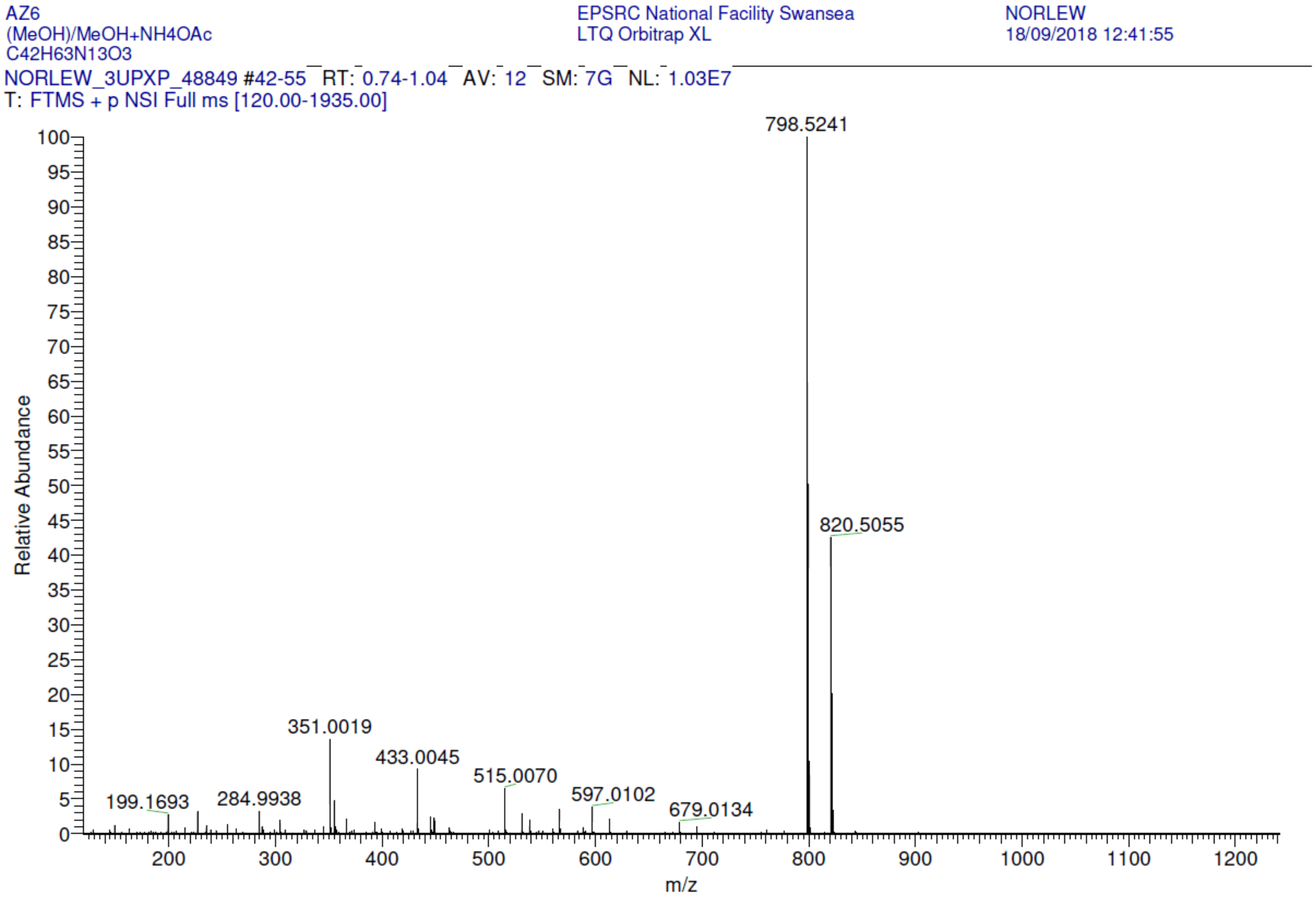

Compound **19b**

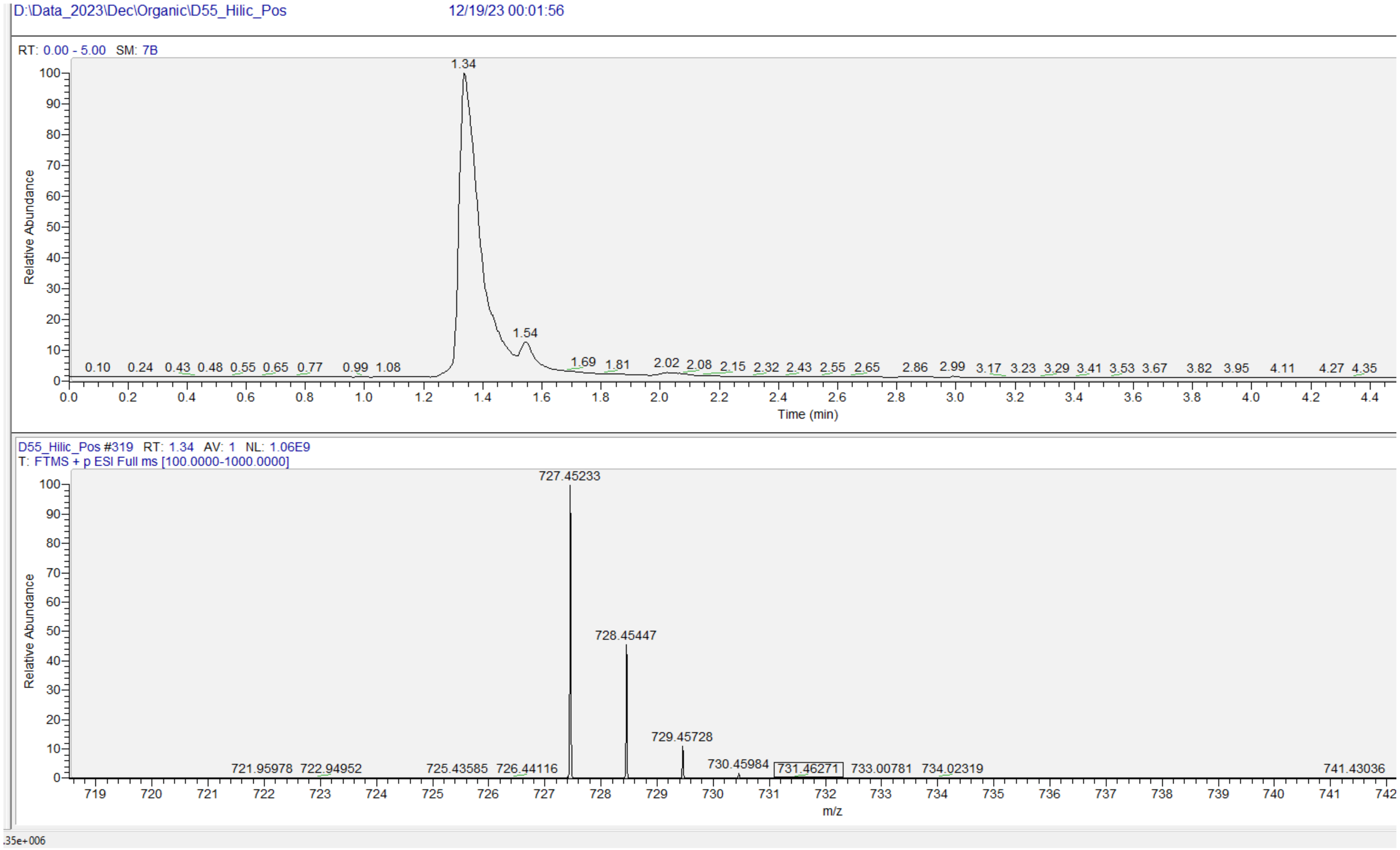

Compound **23b**

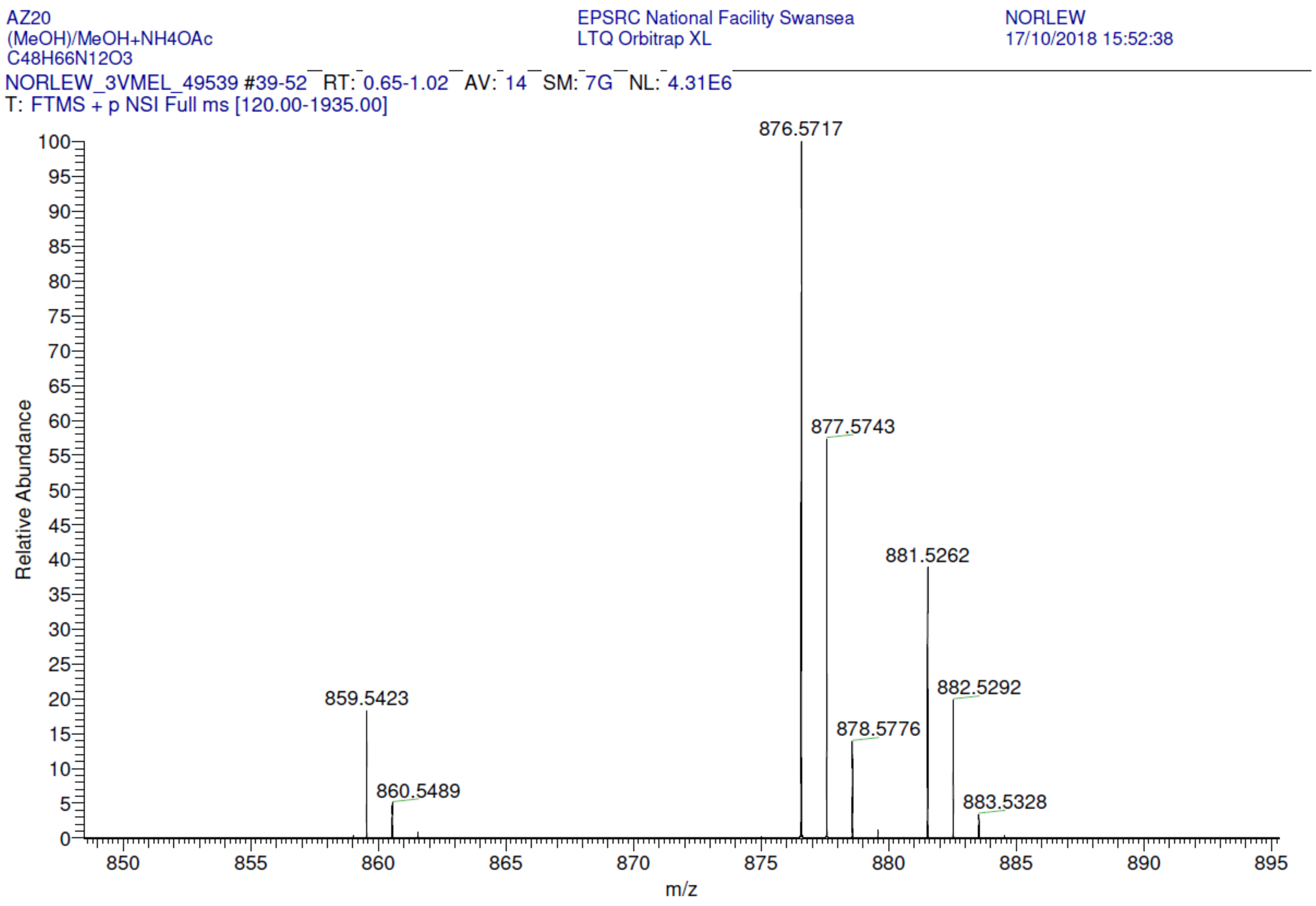

3: Solvent Extraction Studies

Table S1. Extraction of Am(III) and Eu(III) by solutions of ligands **12b**, **12c**, **14b** and **14c** (0.01 M each in different diluents) from nitric acid. Results are from gamma spectrometry (*D* = distribution ratio, *SF* = separation factor, nd = not determined due to very low *D* values, temperature: 22 ^{o}C ± 1 ^{o}C).

**Ligand**	**Diluent**	**[HNO_3] initial (mol/L)**	**Contact time (min)**	D_{Am}	D_{Eu}	$SF_{Eu/Am}$
**12b**	1-octanol	1	120	<0.003	<0.003	nd
**12b**	1-octanol	0.001	60	<0.003	0.003	nd
**12b**	cyclohexanone	1	120	1.03	1.07	1.04
**12b**	nitrobenzene	1	60	<0.003	<0.003	nd
**12c**	1-octanol	1	120	<0.003	<0.003	nd
**12c**	1-octanol	0.001	60	<0.003	<0.003	nd
**12c**	cyclohexanone	1	120	<0.003	<0.003	nd
**12c**	nitrobenzene	1	60	<0.003	<0.003	nd
**14b**	1-octanol	1	120	0.007	0.003	nd
**14b**	1-octanol	0.001	60	0.003	0.004	nd
**14b**	cyclohexanone	1	120	<0.003	<0.003	nd
**14c**	1-octanol	1	60	0.009	0.003	nd
**14c**	1-octanol	1	120	0.006	<0.003	nd
**14c**	1-octanol	0.001	60	0.003	<0.003	nd

Table S2. Extraction of Am(III) and Eu(III) by ligand **9a** (0.01 M) into 1-octanol as a function of the nitric acid concentration. Results are from gamma spectrometry (*D* = distribution ratio, *SF* = separation factor, contact time: 60 min, temperature: 22 °C ± 1 °C).

**$[HNO_3]$ final (mol/L)**	D_{Am}	D_{Eu}	$SF_{Am/Eu}$
0.001	0.001	0.003	nd
0.013	0.001	0.002	nd
0.111	0.001	0.003	nd
1.031	0.001	0.003	nd
1.998	0.001	0.003	nd
4.123	0.002	0.004	nd

Table S3. Extraction of Am(III) and Cm(III) by ligand **9a** (0.01 M) into 1-octanol as a function of the nitric acid concentration. Results are from alpha spectrometry (*D* = distribution ratio, *SF* = separation factor, contact time: 60 min, temperature: 22 °C ± 1 °C).

**$[HNO_3]$ final (mol/L)**	D_{Am}	D_{Cm}	$SF_{Am/Cm}$
0.001	0.003	0.002	nd
0.013	0.004	0.001	nd
0.111	0.001	<0.001	nd
1.031	0.002	0.001	nd
1.998	0.002	0.001	nd
4.123	0.003	0.002	nd

Table S4. Extraction of Am(III) and Eu(III) by ligand **14a** (0.01 M) into 1-octanol as a function of the nitric acid concentration. Results are from gamma spectrometry (*D* = distribution ratio, *SF* = separation factor, contact time: 60 min, temperature: 22 ^{o}C ± 1 ^{o}C).

**[HNO$_3$] final (mol/L)**	D_{Am}	D_{Eu}	$SF_{Am/Eu}$
0.001	0.001	0.003	nd
0.013	0.001	0.003	nd
0.111	0.001	0.003	nd
1.031	0.001	0.003	nd
1.998	0.002	0.003	nd
4.123	0.002	0.004	nd

Table S5. Extraction of Am(III) and Cm(III) by ligand **14a** (0.01 M) into 1-octanol as a function of the nitric acid concentration. Results are from alpha spectrometry (*D* = distribution ratio, *SF* = separation factor, contact time: 60 min, temperature: 22 ^{o}C ± 1 ^{o}C).

**[HNO$_3$] final (mol/L)**	D_{Am}	D_{Cm}	$SF_{Am/Cm}$
0.001	0.004	0.001	nd
0.013	0.003	0.001	nd
0.111	0.001	0.001	nd
1.031	0.002	0.001	nd
1.998	0.002	0.001	nd
4.123	0.003	0.002	nd

Table S6. Extraction of Am(III) and Eu(III) by ligand **19b** (0.01 M) into 1-octanol as a function of the nitric acid concentration. Results are from gamma spectrometry (*D* = distribution ratio, *SF* = separation factor, contact time: 60 min, temperature: 22 ^{o}C ± 1 ^{o}C).

**$[HNO_3]$ final (mol/L)**	**D_{Am}**	**D_{Eu}**	**$SF_{Am/Eu}$**
0.001	0.002	0.003	nd
0.013	0.001	0.003	nd
0.111	0.001	0.003	nd
1.031	0.001	0.003	nd
1.998	0.002	0.003	nd
4.123	0.003	0.003	nd

Table S7. Extraction of Am(III) and Cm(III) by ligand **19b** (0.01 M) into 1-octanol as a function of the nitric acid concentration. Results are from alpha spectrometry (*D* = distribution ratio, *SF* = separation factor, contact time: 60 min, temperature: 22 ^{o}C ± 1 ^{o}C).

**$[HNO_3]$ final (mol/L)**	**D_{Am}**	**D_{Cm}**	**$SF_{Am/Cm}$**
0.001	0.008	0.003	nd
0.013	0.003	0.001	nd
0.111	0.001	0.001	nd
1.031	0.001	0.001	nd
1.998	0.002	0.001	nd
4.123	0.004	0.003	nd

Table S8. Extraction of Am(III) and Eu(III) by ligand **23b** (0.01 M) into 1-octanol as a function of the nitric acid concentration. Results are from gamma spectrometry (*D* = distribution ratio, *SF* = separation factor, contact time: 60 min, temperature: 22 ^{o}C ± 1 ^{o}C).

**[HNO_3] final (mol/L)**	D_{Am}	D_{Eu}	$SF_{Am/Eu}$
0.001	0.001	0.003	nd
0.013	0.001	0.002	nd
0.111	0.001	0.002	nd
1.031	0.002	0.002	nd
1.998	0.002	0.004	nd
4.123	0.003	0.004	nd

Table S9. Extraction of Am(III) and Cm(III) by ligand **23b** (0.01 M) into 1-octanol as a function of the nitric acid concentration. Results are from alpha spectrometry (*D* = distribution ratio, *SF* = separation factor, contact time: 60 min, temperature: 22 ^{o}C ± 1 ^{o}C).

**[HNO_3] final (mol/L)**	D_{Am}	D_{Cm}	$SF_{Am/Cm}$
0.001	0.004	0.001	nd
0.013	0.003	0.001	nd
0.111	0.001	0.001	nd
1.031	0.002	0.001	nd
1.998	0.002	0.001	nd
4.123	0.004	0.003	nd

4: NMR Titrations with Lanthanide Salts

4.1: NMR Titrations with Ligand 9b

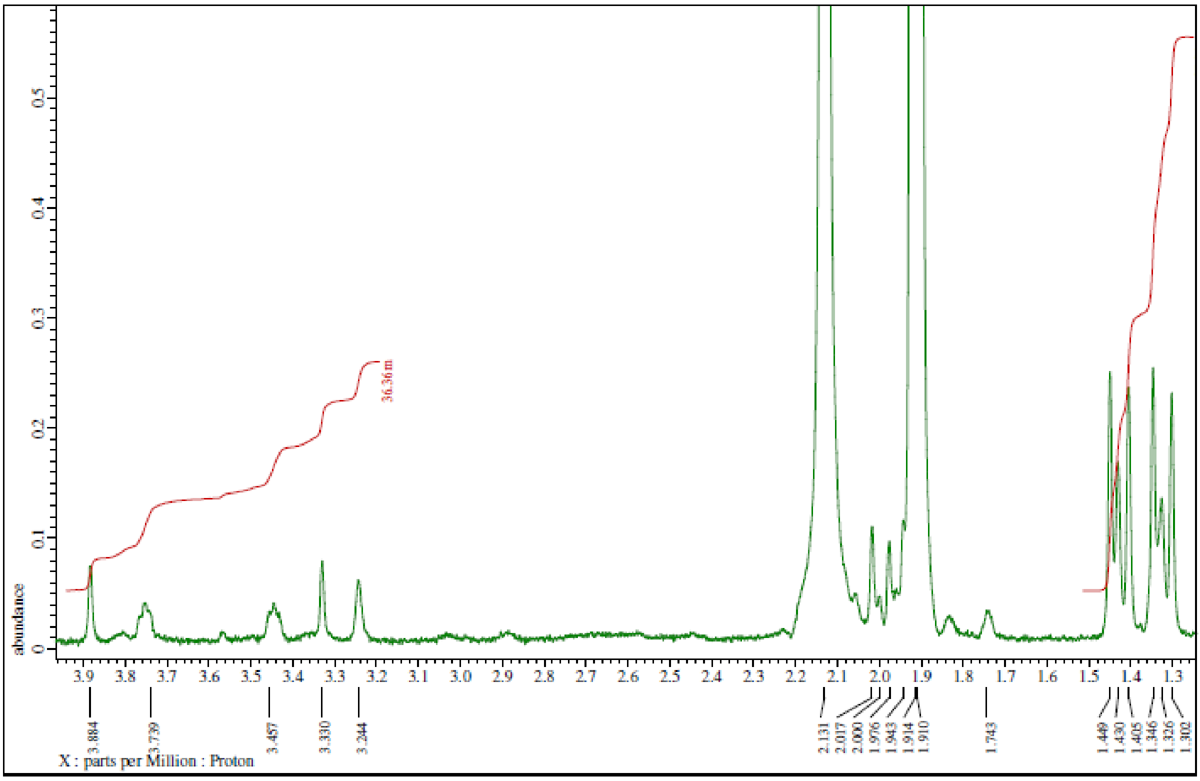

Figure S1. ^{1}H NMR spectrum of ligand **9b** in CD_3CN. Peaks at 1.91 ppm and 2.13 ppm are due to solvents.

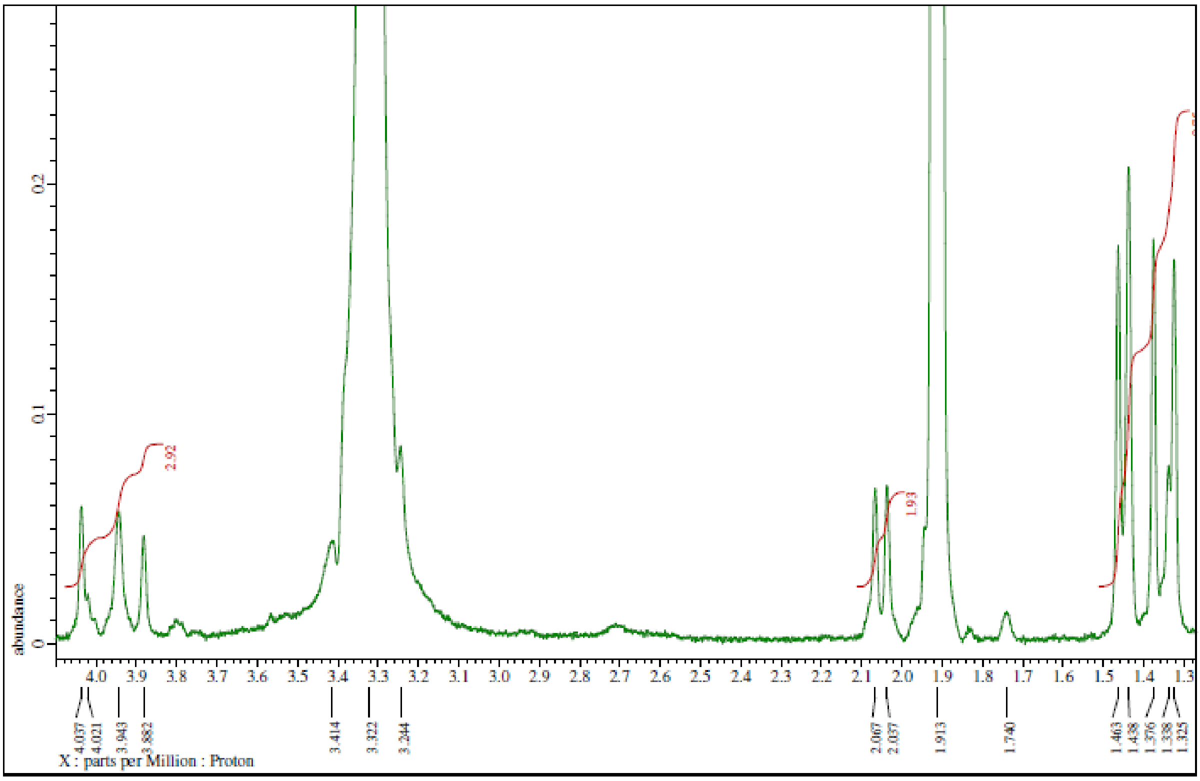

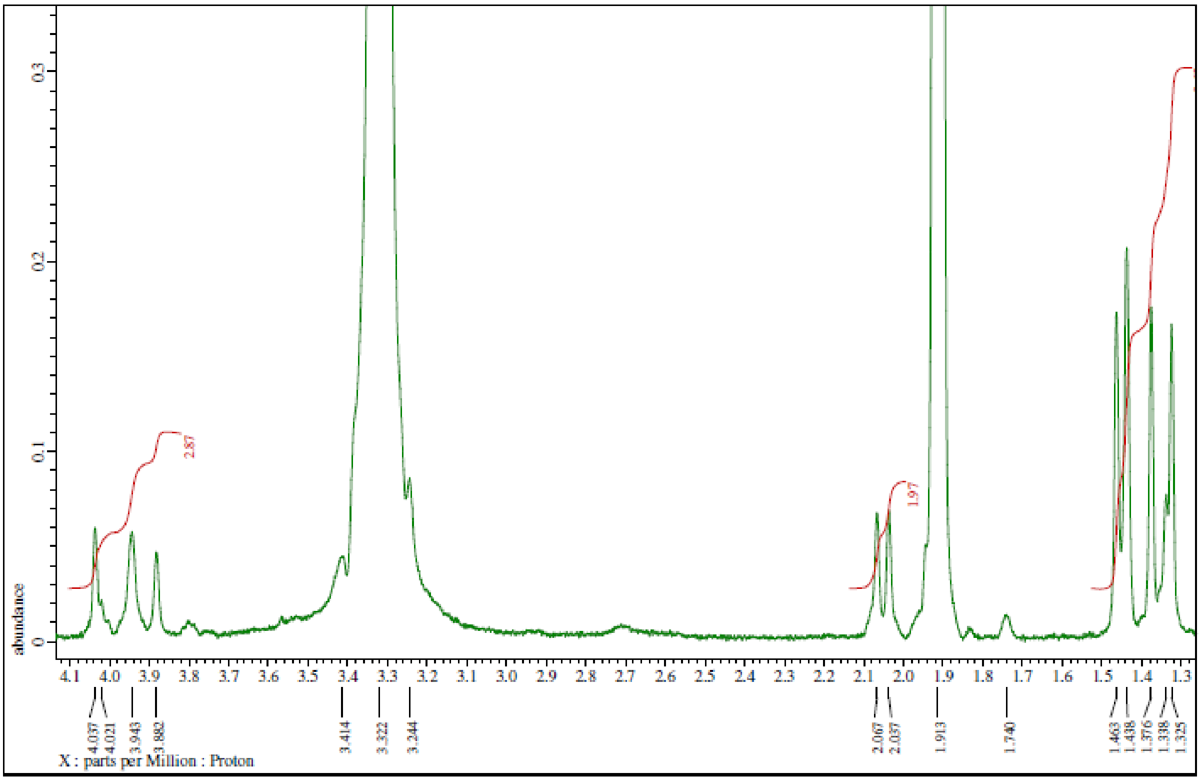

Figure S2. Top: ^1H NMR spectrum of ligand **9b** + $Y(NO_3)_3$ (0.5 equivalents) in CD_3CN. Bottom: ^1H NMR spectrum of ligand **9b** + $Y(NO_3)_3$ (1 equivalent) in CD_3CN. Peaks at 1.91 ppm and 3.32 ppm are due to solvents.

4.2: NMR Titrations with Ligand 12b

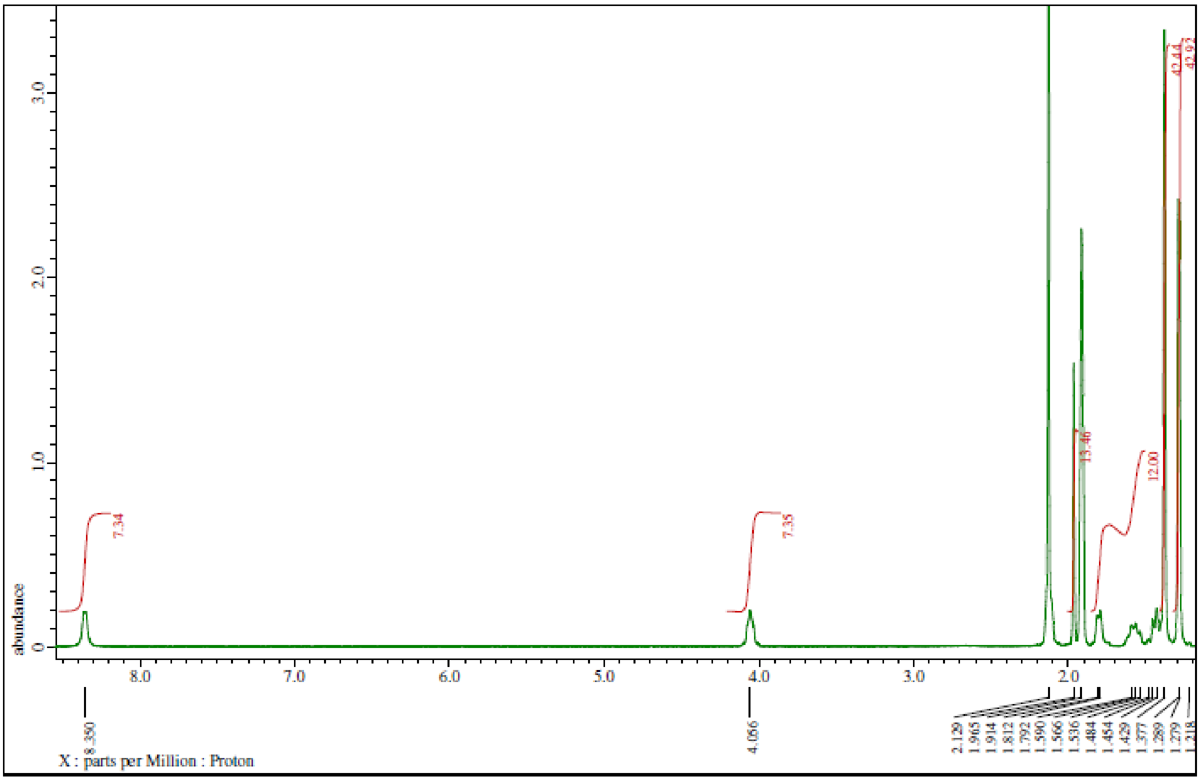

Figure S3. ^{1}H NMR spectrum of ligand **12b** in CD_3CN. Peaks at 1.91 ppm and 2.12 ppm are due to solvents.

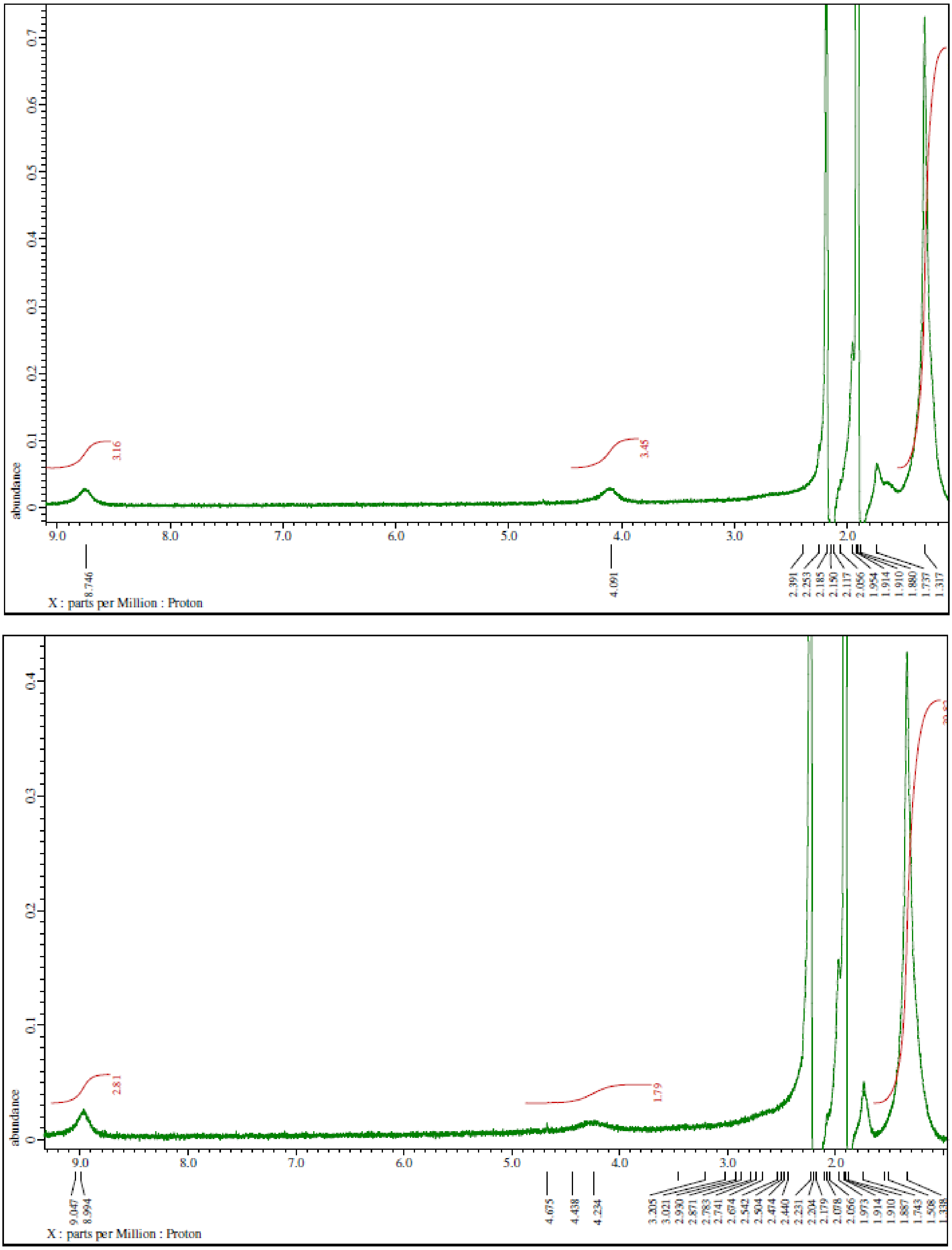

Figure S4. Top: ^{1}H NMR spectrum of ligand **12b** + $La(NO_3)_3$ (0.5 equivalents) in CD_3CN. Bottom: ^{1}H NMR spectrum of ligand **12b** + $La(NO_3)_3$ (1 equivalent) in CD_3CN. Peaks at 1.91 ppm and 2.18 ppm are due to solvents.

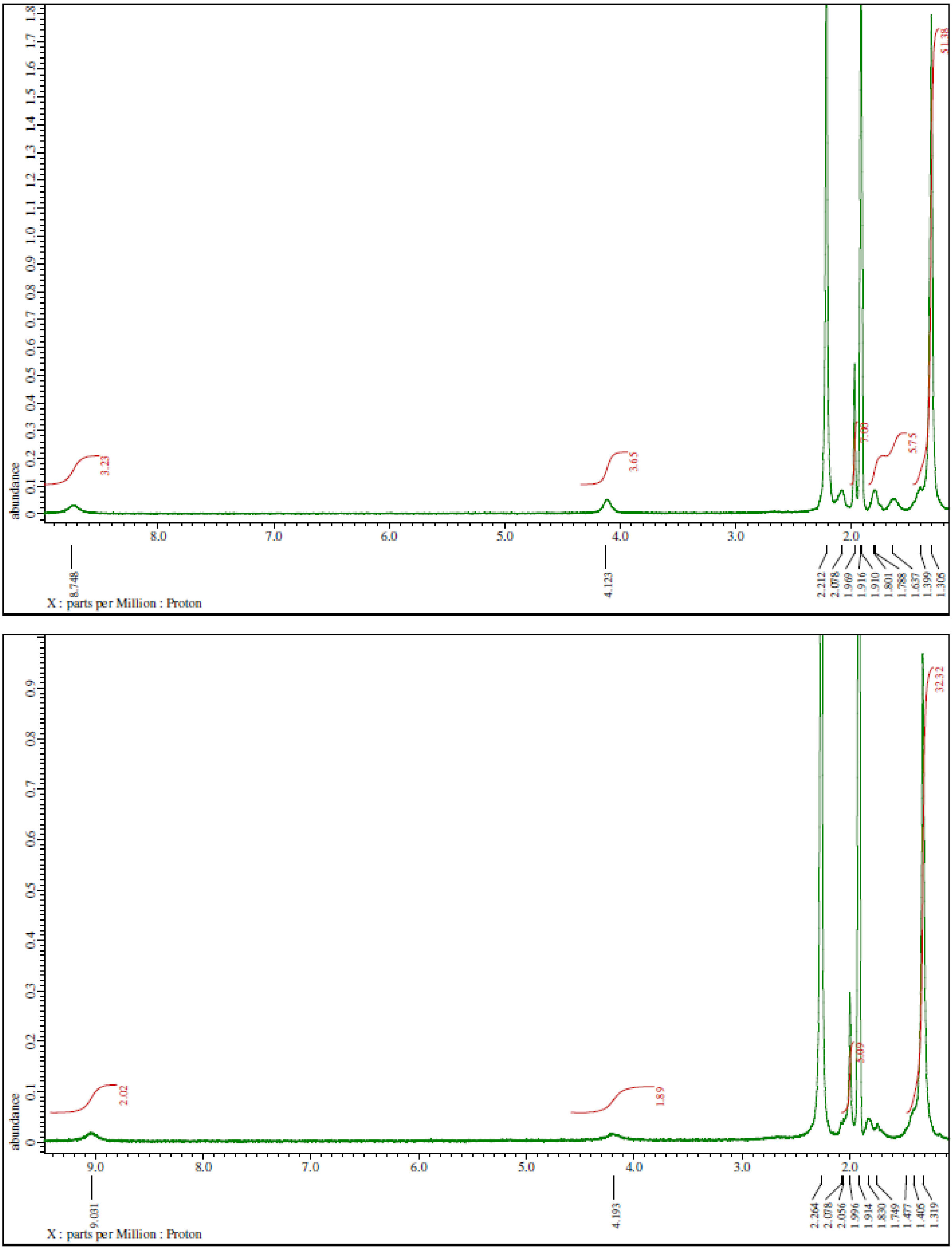

Figure S5. Top: ^{1}H NMR spectrum of ligand **12b** + $Y(NO_3)_3$ (0.5 equivalents) in CD_3CN. Bottom: ^{1}H NMR spectrum of ligand **12b** + $Y(NO_3)_3$ (1 equivalent) in CD_3CN. Peaks at 1.91 ppm and 2.26 ppm are due to solvents.

4.3: NMR Titrations with Ligand 12c

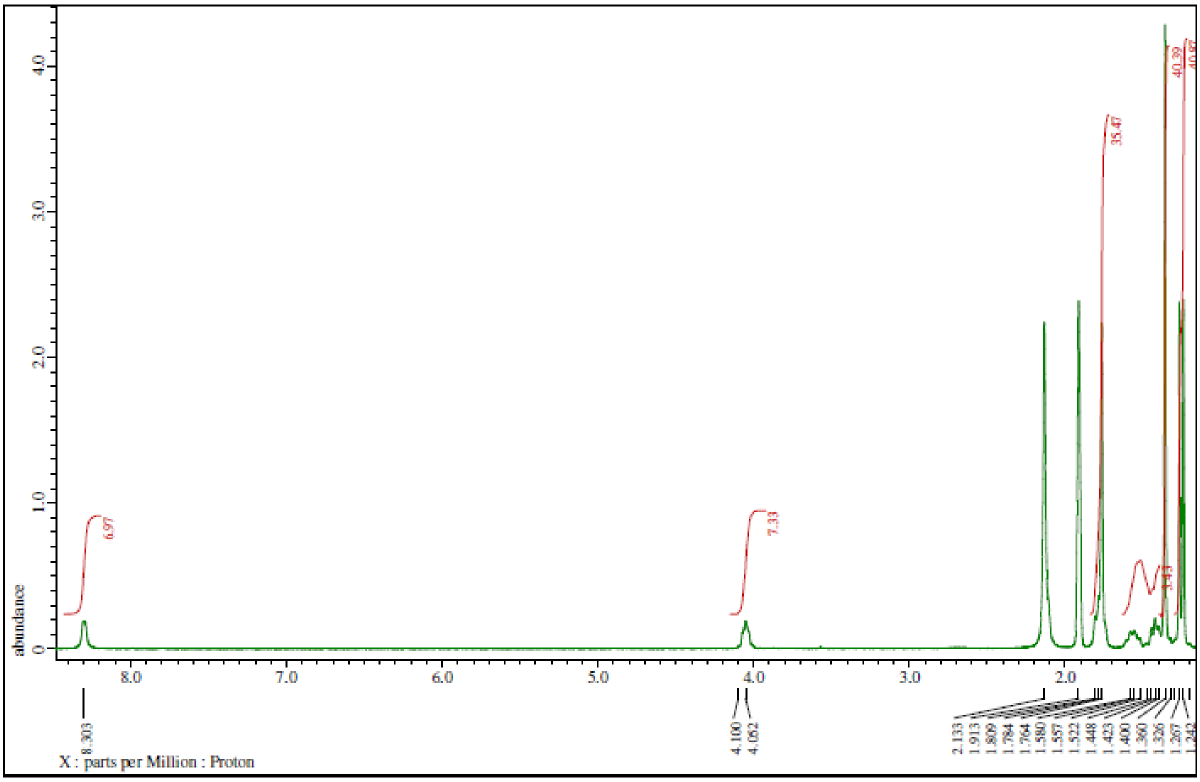

Figure S6. ^{1}H NMR spectrum of ligand **12c** in CD_3CN. Peaks at 1.91 ppm and 2.13 ppm are due to solvents.

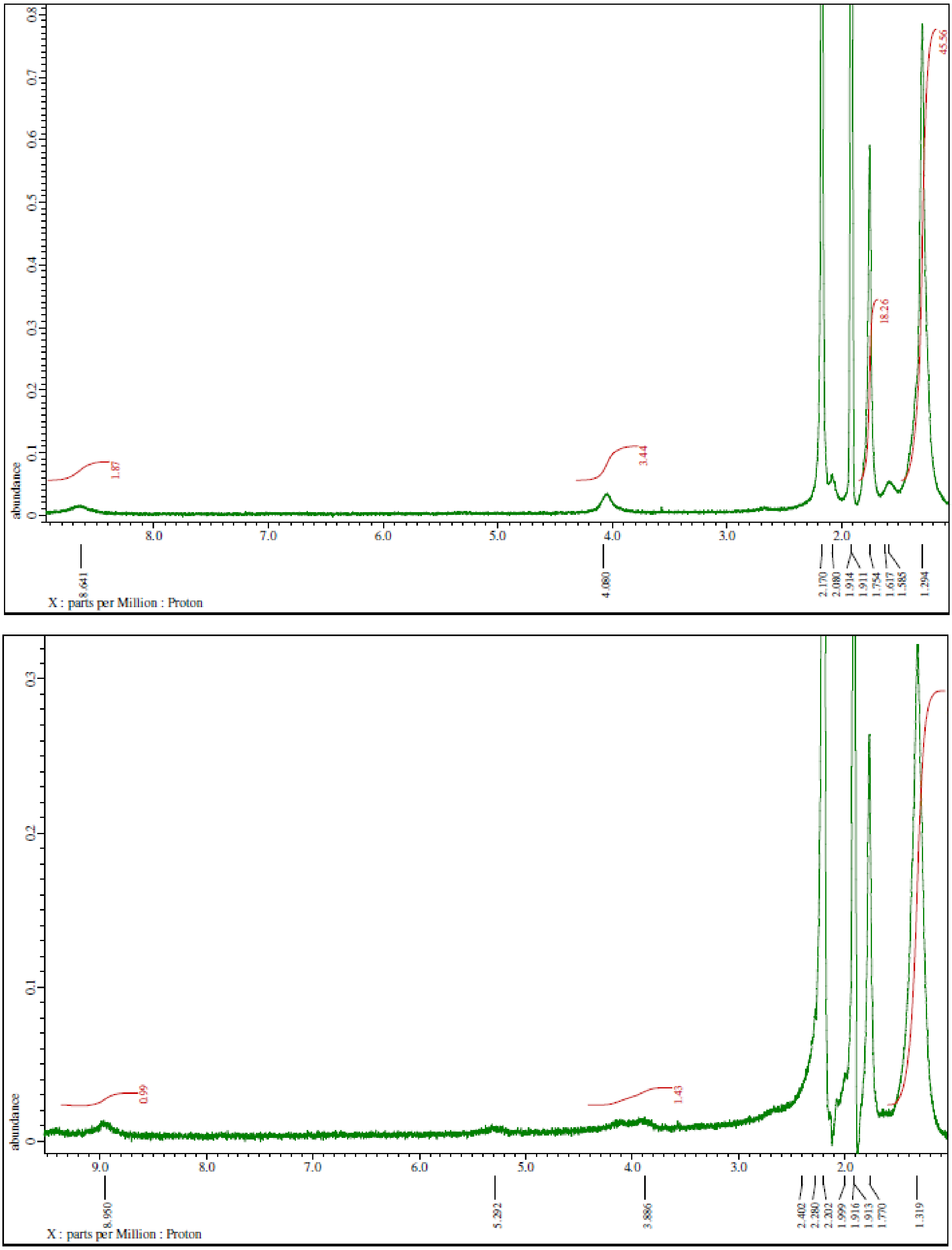

Figure S7. Top: ^{1}H NMR spectrum of ligand **12c** + $Y(NO_3)_3$ (0.5 equivalents) in CD_3CN. Bottom: ^{1}H NMR spectrum of ligand **12c** + $Y(NO_3)_3$ (1 equivalent) in CD_3CN. Peaks at 1.91 ppm and 2.17 ppm are due to solvents.

4.4: NMR Titrations with Ligand 14b

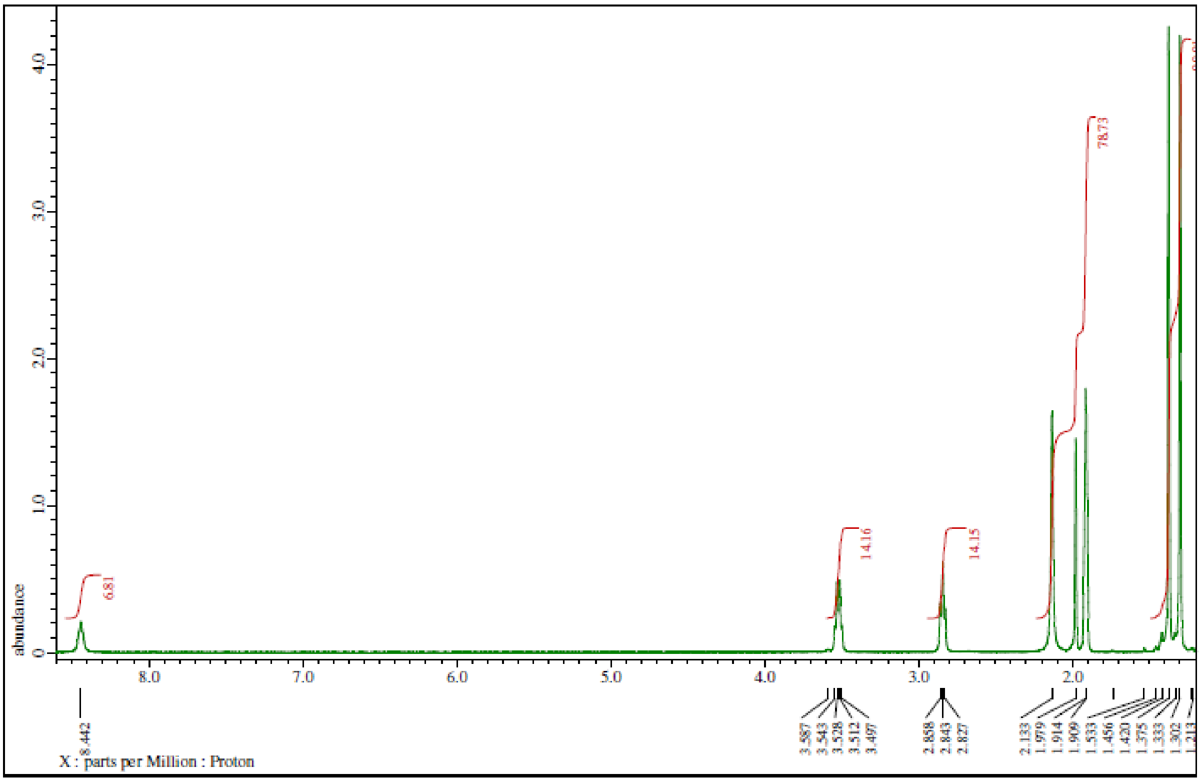

Figure S8. ^{1}H NMR spectrum of ligand **14b** in CD_3CN. Peaks at 1.91 ppm and 2.13 ppm are due to solvents.

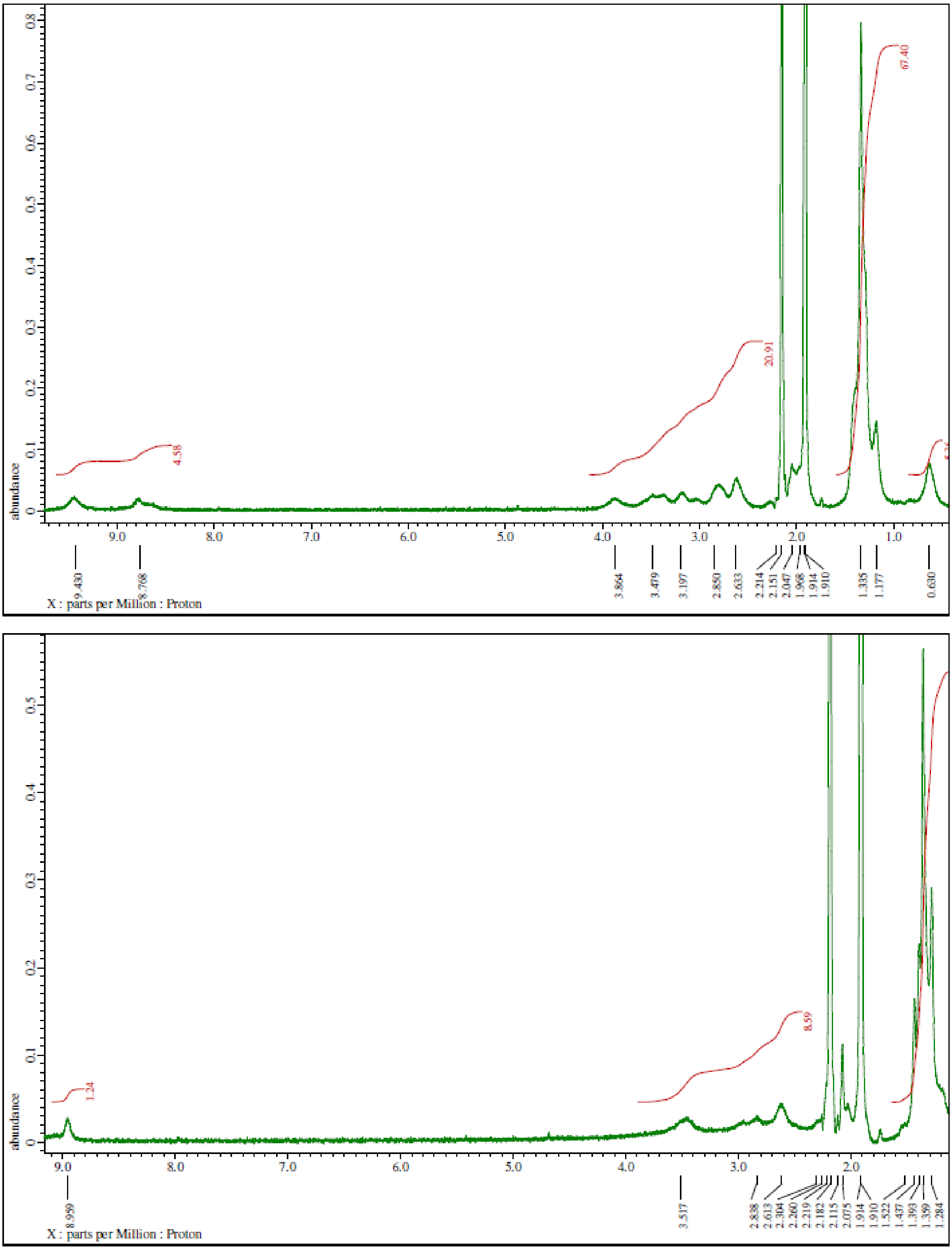

Figure S9. Top: ^{1}H NMR spectrum of ligand **14b** + $La(NO_3)_3$ (0.5 equivalents) in CD_3CN. Bottom: ^{1}H NMR spectrum of ligand **14b** + $La(NO_3)_3$ (1 equivalent) in CD_3CN. Peaks at 1.91 ppm and 2.15 ppm are due to solvents.

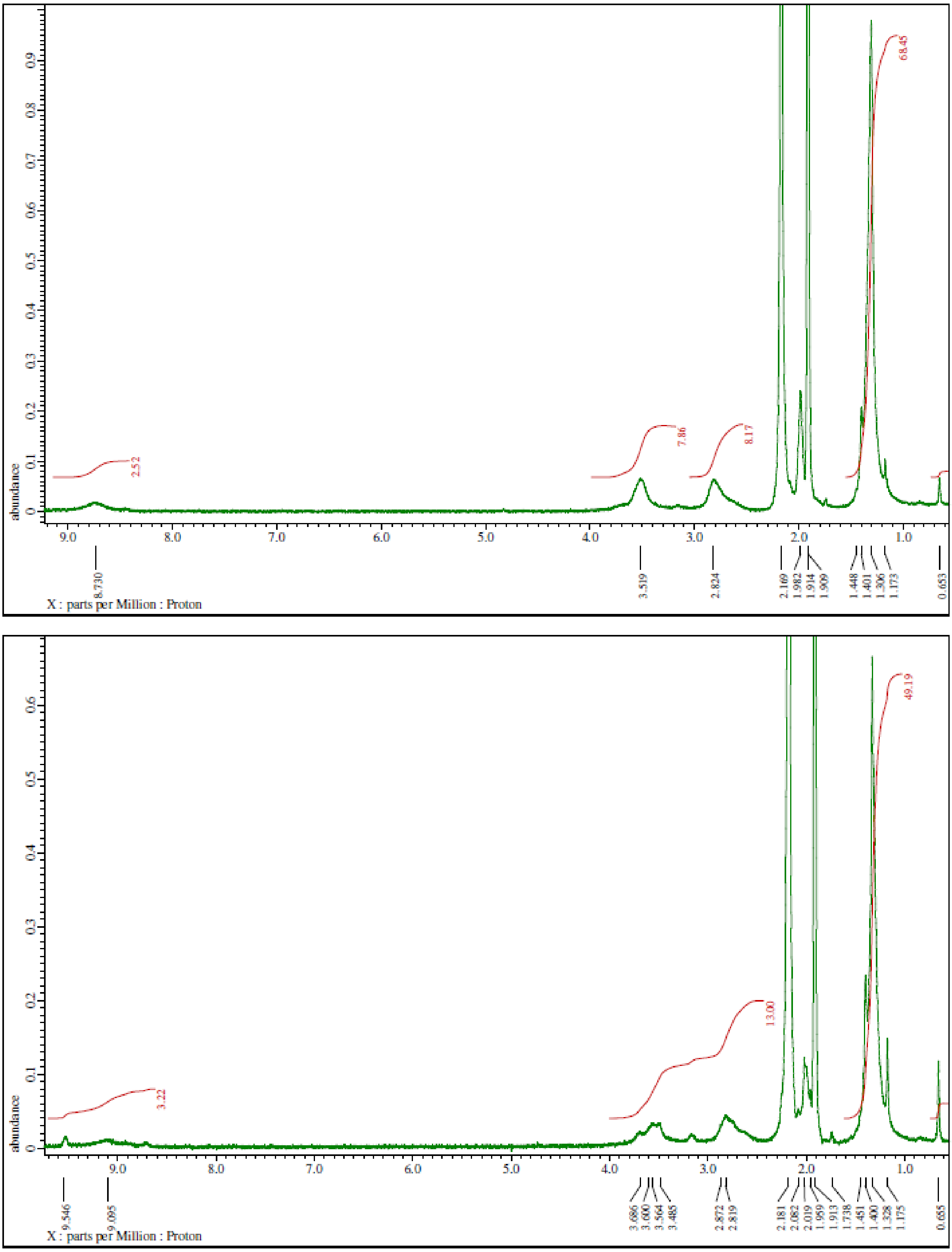

Figure S10. Top: ^{1}H NMR spectrum of ligand **14b** + $Y(NO_3)_3$ (0.5 equivalents) in CD_3CN. Bottom: ^{1}H NMR spectrum of ligand **14b** + $Y(NO_3)_3$ (1 equivalent) in CD_3CN. Peaks at 1.91 ppm and 2.18 ppm are due to solvents.

4.5: NMR Titrations with Ligand 23b

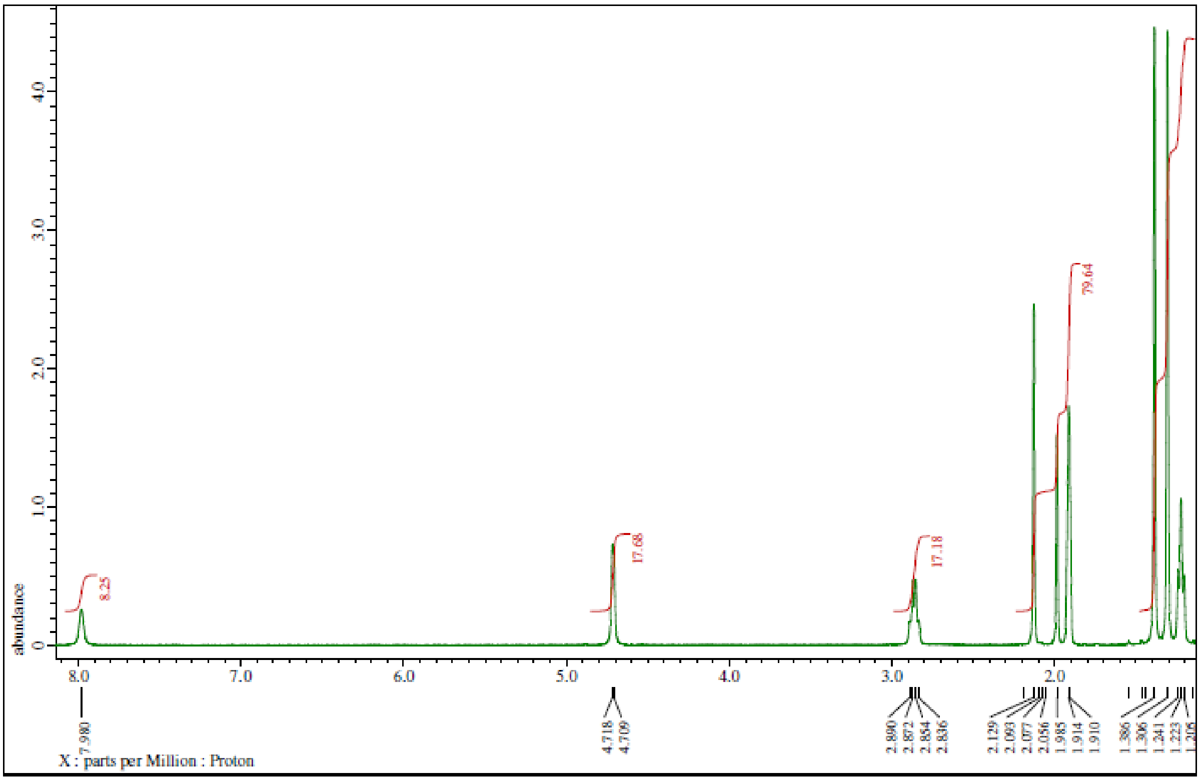

Figure S11. ^{1}H NMR spectrum of ligand **23b** in CD_3CN. Peaks at 1.91 ppm and 2.12 ppm are due to solvents.

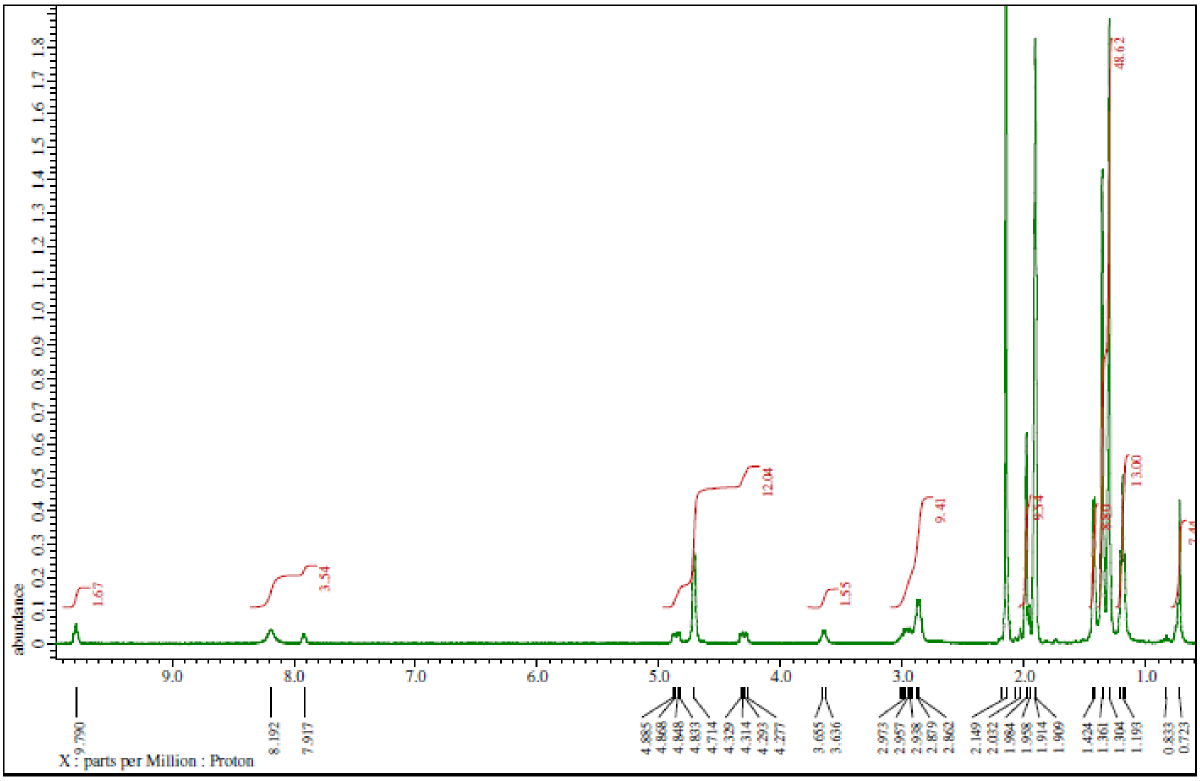

Figure S12. ¹H NMR spectrum of ligand **23b** + $La(NO_3)_3$ (0.5 equivalents) in CD_3CN. Peaks at 1.91 ppm and 2.14 ppm are due to solvents.

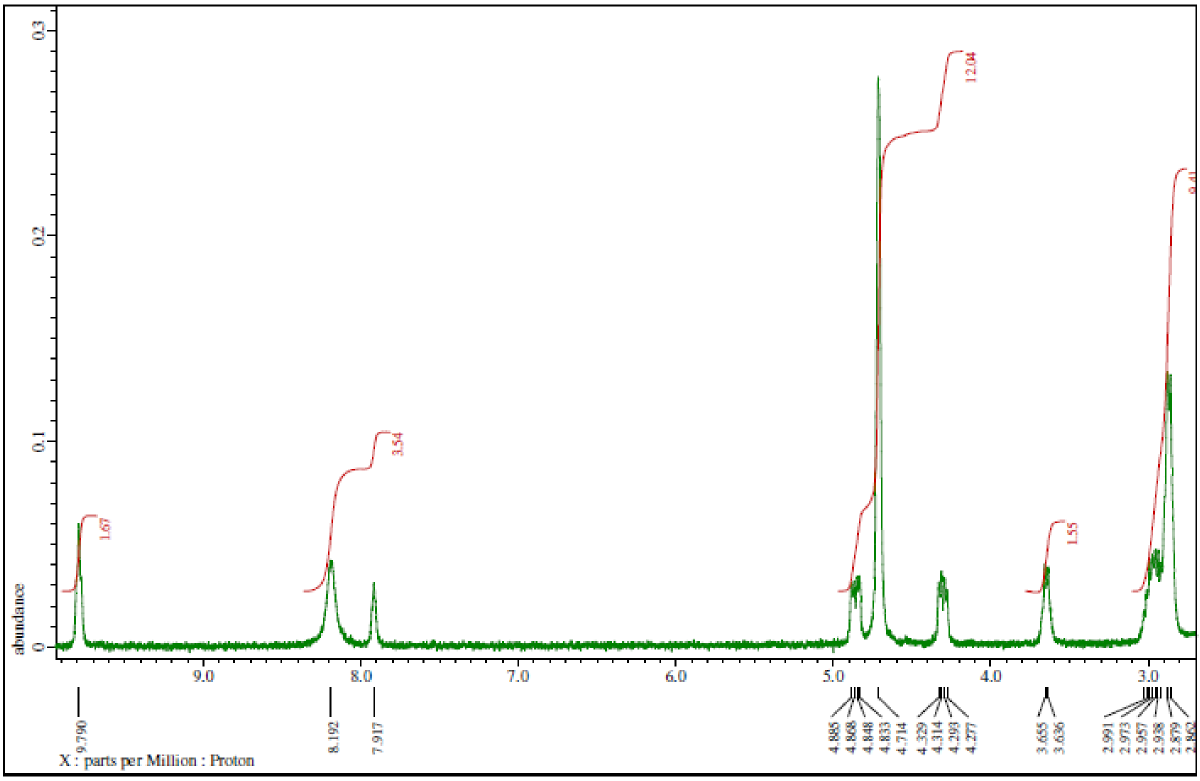

Figure S13. ¹H NMR spectrum of ligand **23b** + $La(NO_3)_3$ (0.5 equivalents) in CD_3CN. Region between 2.7 ppm and 9.8 ppm is shown for clarity.

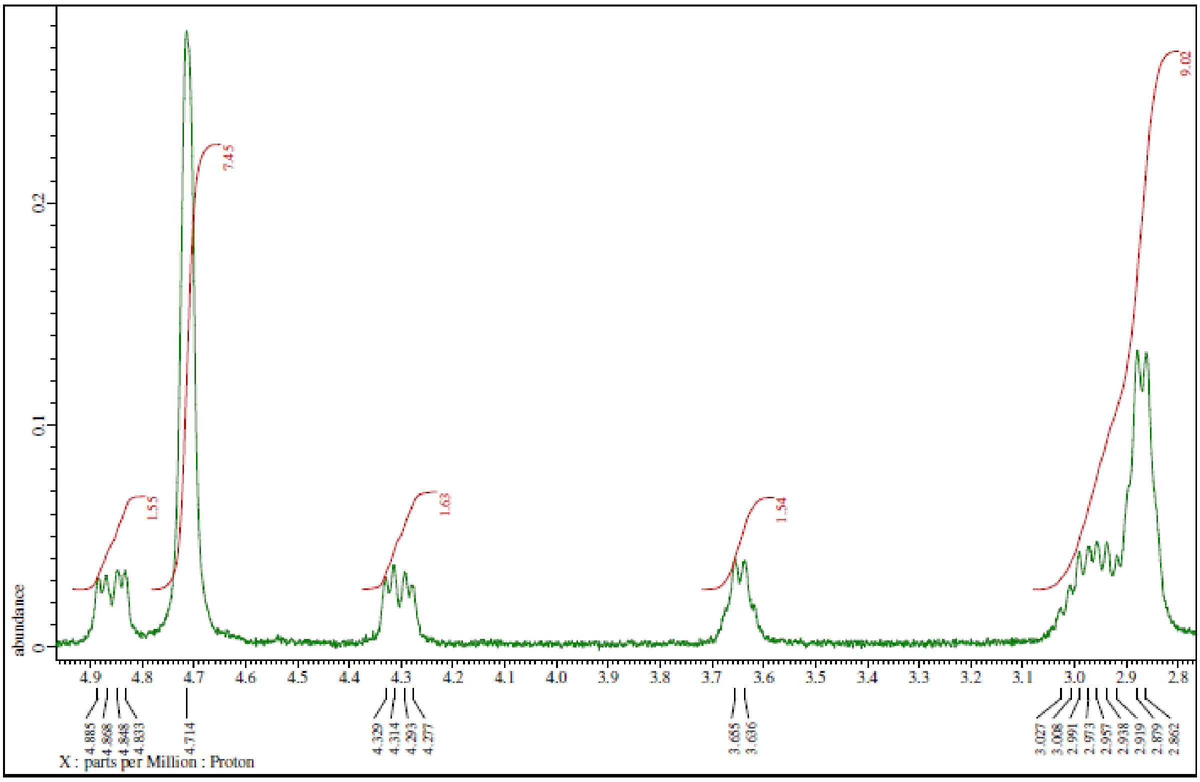

Figure S14. ^{1}H NMR spectrum of ligand **23b** + $La(NO_3)_3$ (0.5 equivalents) in CD_3CN. Region between 2.8 ppm and 4.9 ppm is shown for clarity.

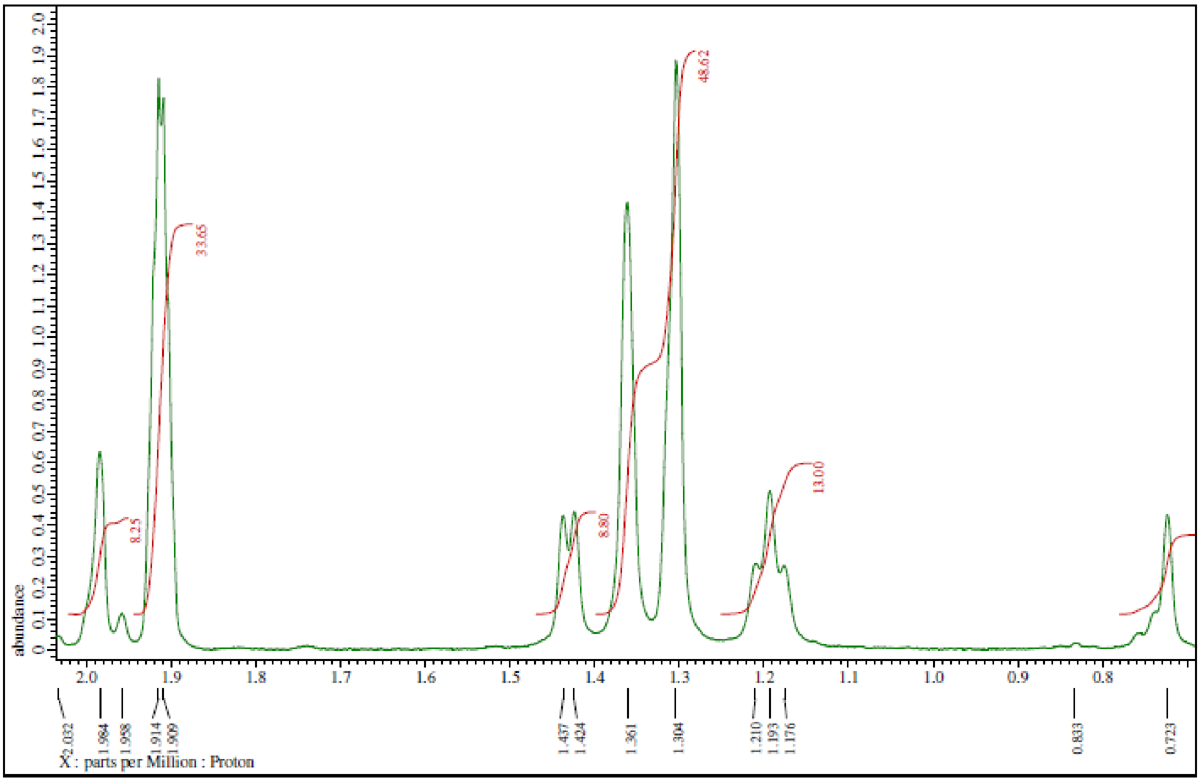

Figure S15. ^{1}H NMR spectrum of ligand **23b** + $La(NO_3)_3$ (0.5 equivalents) in CD_3CN. Region between 0.7 ppm and 2.0 ppm is shown for clarity.

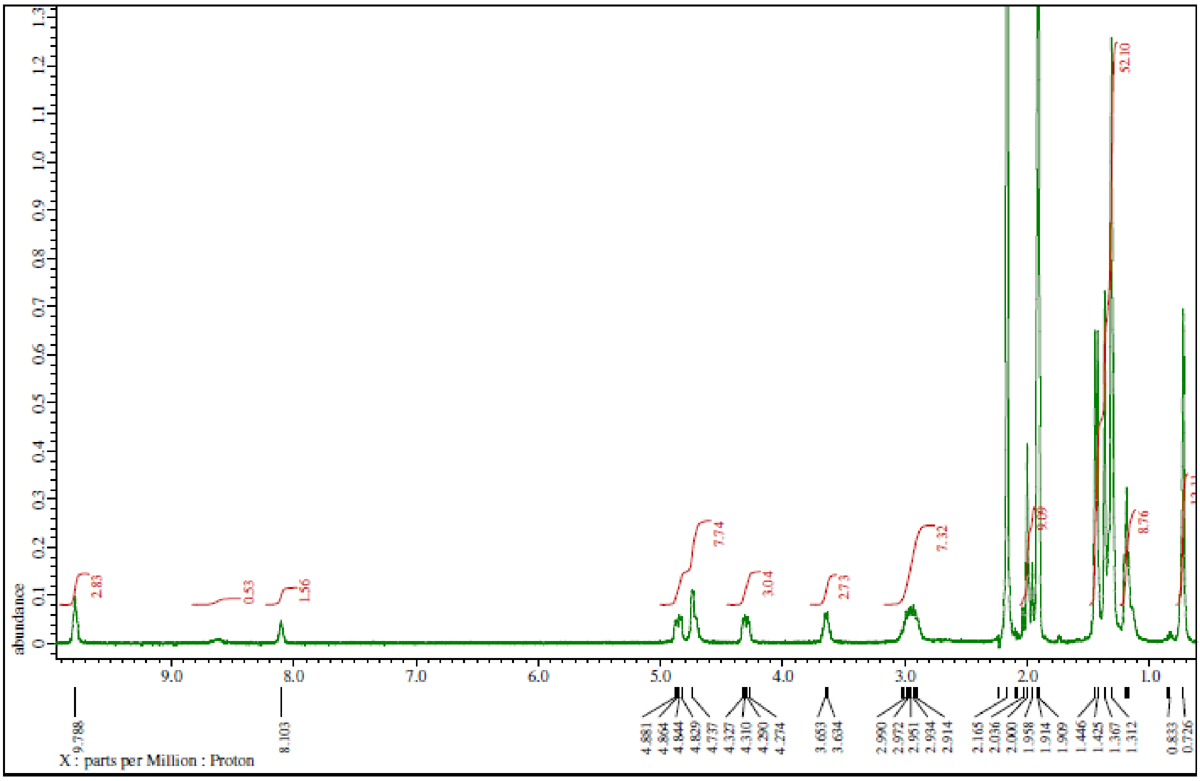

Figure S16. ^{1}H NMR spectrum of ligand **23b** + $La(NO_3)_3$ (1 equivalent) in CD_3CN. Peaks at 1.91 ppm and 2.16 ppm are due to solvents.

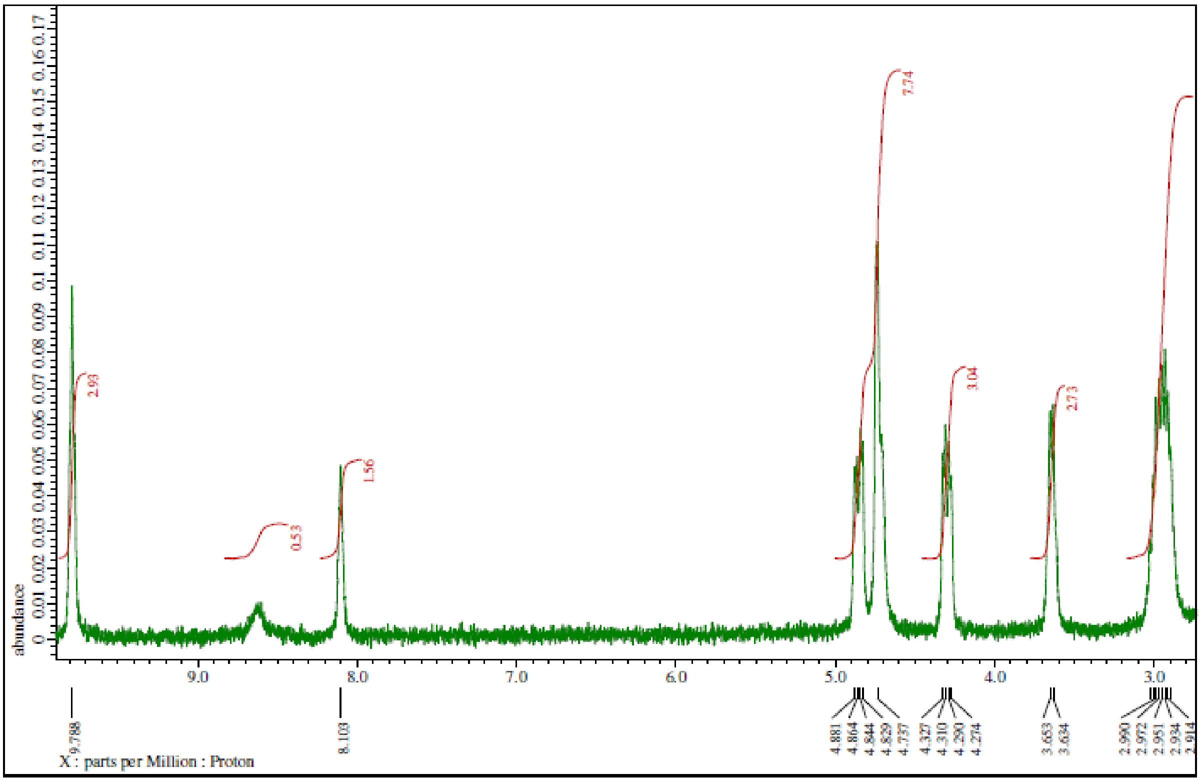

Figure S17. ^{1}H NMR spectrum of ligand **23b** + $La(NO_3)_3$ (1 equivalent) in CD_3CN. Region between 2.8 ppm and 9.8 ppm is shown for clarity.

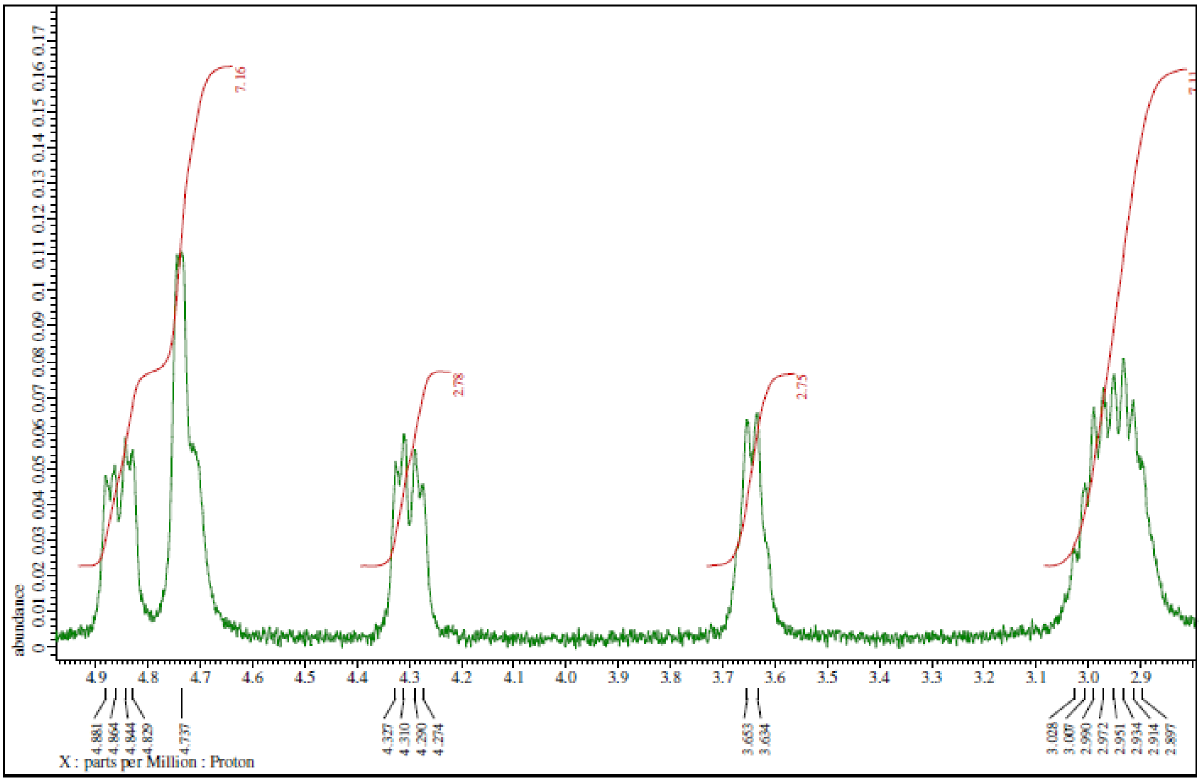

Figure S18. ^{1}H NMR spectrum of ligand **23b** + $La(NO_3)_3$ (1 equivalent) in CD_3CN. Region between 2.8 ppm and 4.9 ppm is shown for clarity.

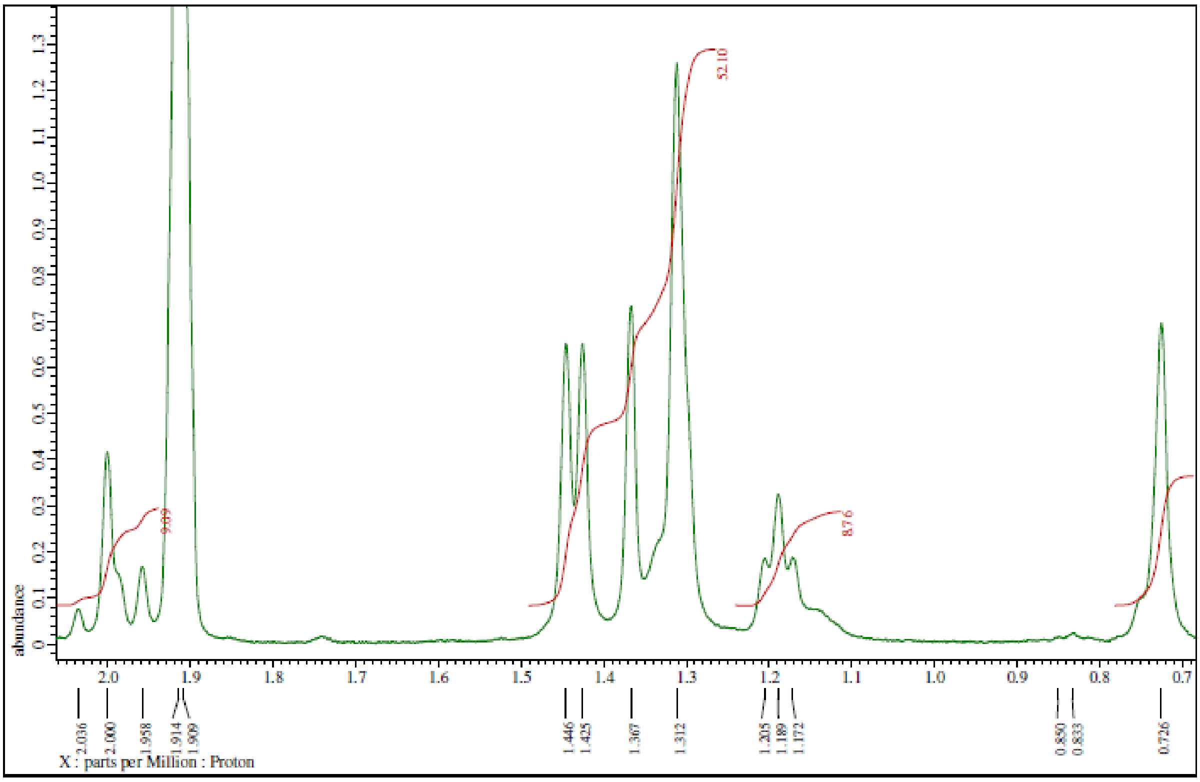

Figure S19. ^{1}H NMR spectrum of ligand **23b** + $La(NO_3)_3$ (1 equivalent) in CD_3CN. Region between 0.7 ppm and 2.0 ppm is shown for clarity.

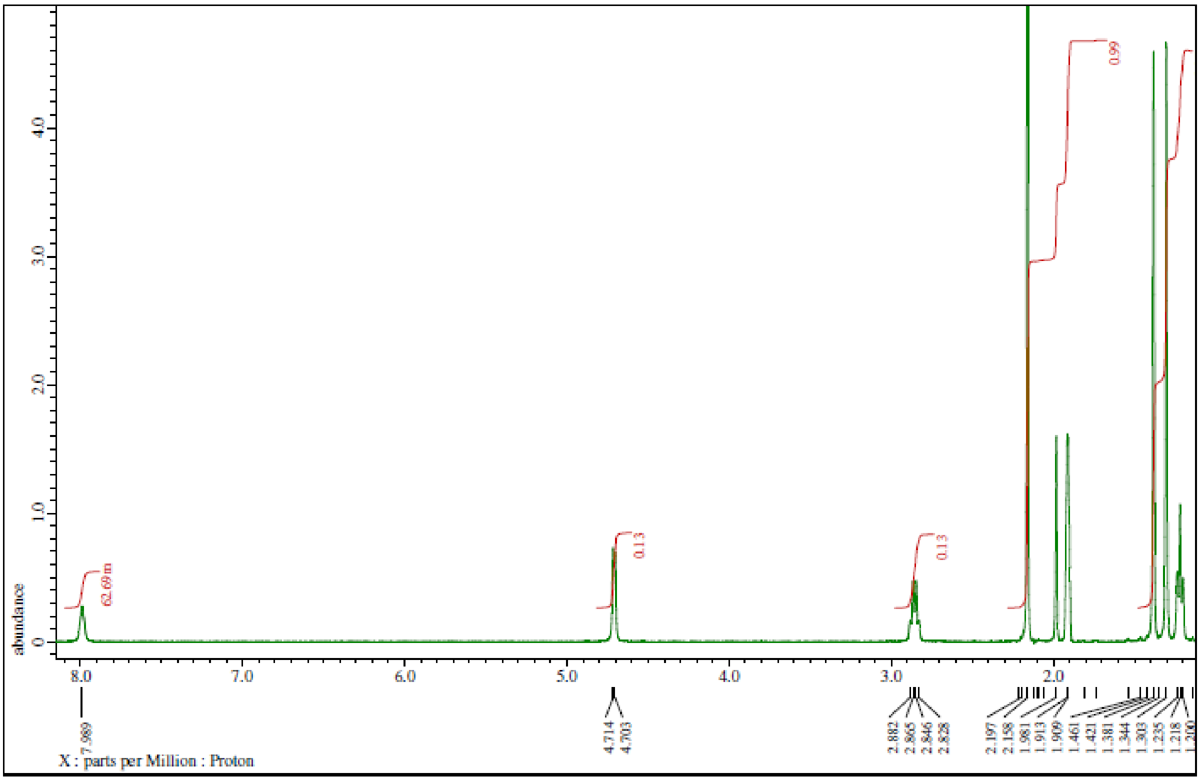

Figure S20. ^{1}H NMR spectrum of ligand **23b** in CD_3CN. Peaks at 1.90 ppm and 2.15 ppm are due to solvents.

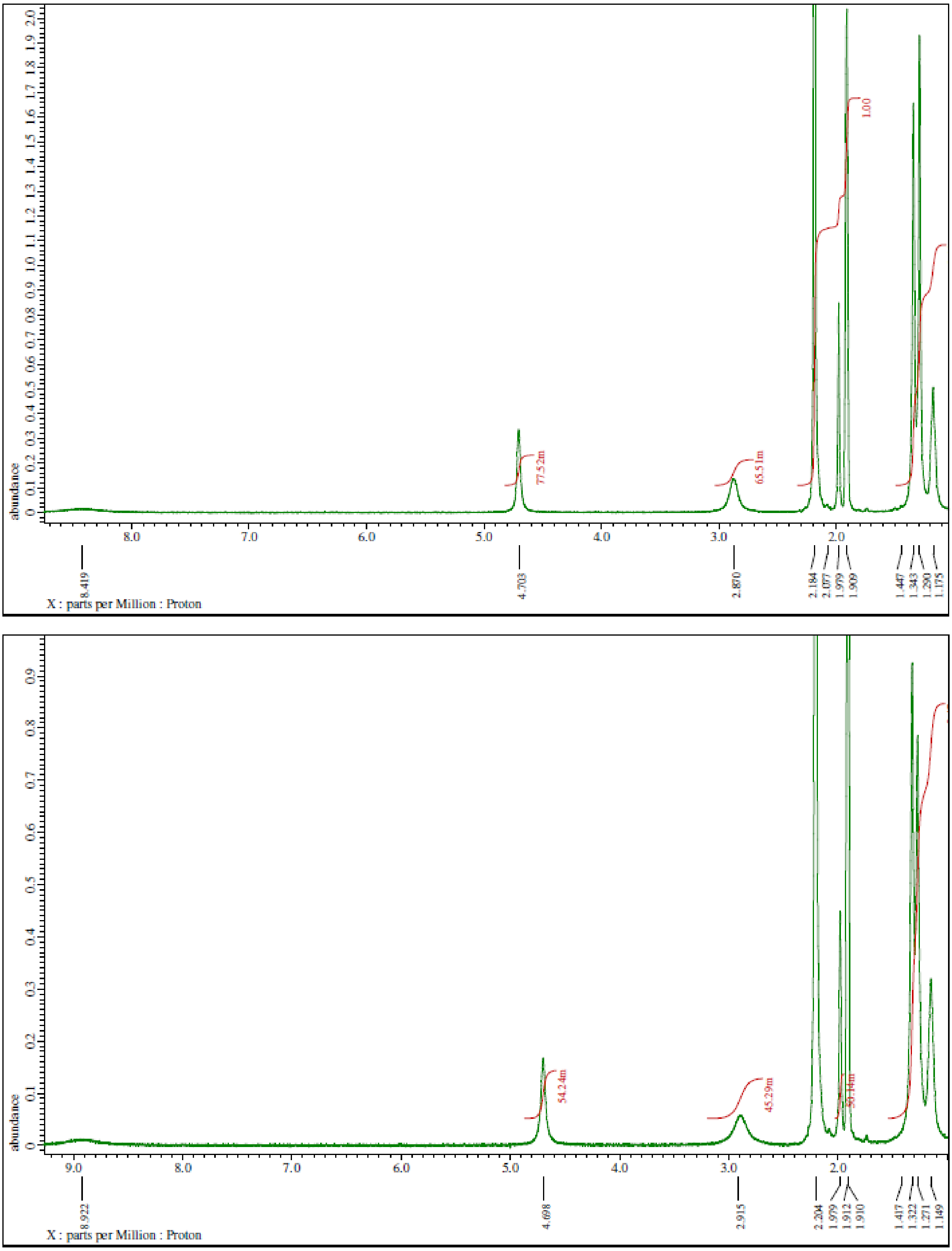

Figure S21. Top: ^{1}H NMR spectrum of ligand **23b** + $Lu(NO_3)_3$ (0.5 equivalents) in CD_3CN. Bottom: ^{1}H NMR spectrum of ligand **23b** + $Lu(NO_3)_3$ (1 equivalent) in CD_3CN. Peaks at 1.91 ppm and 2.20 ppm are due to solvents.

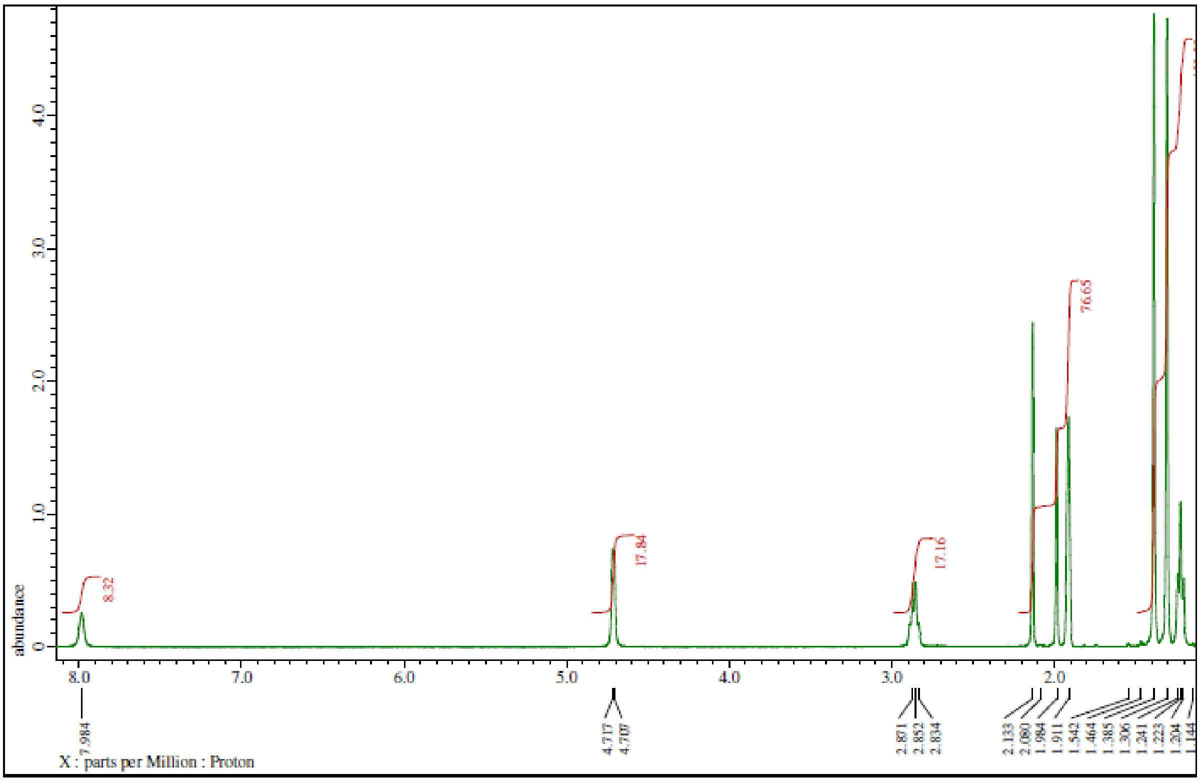

Figure S22. ^{1}H NMR spectrum of ligand **23b** in CD_3CN. Peaks at 1.91 ppm and 2.13 ppm are due to solvents.

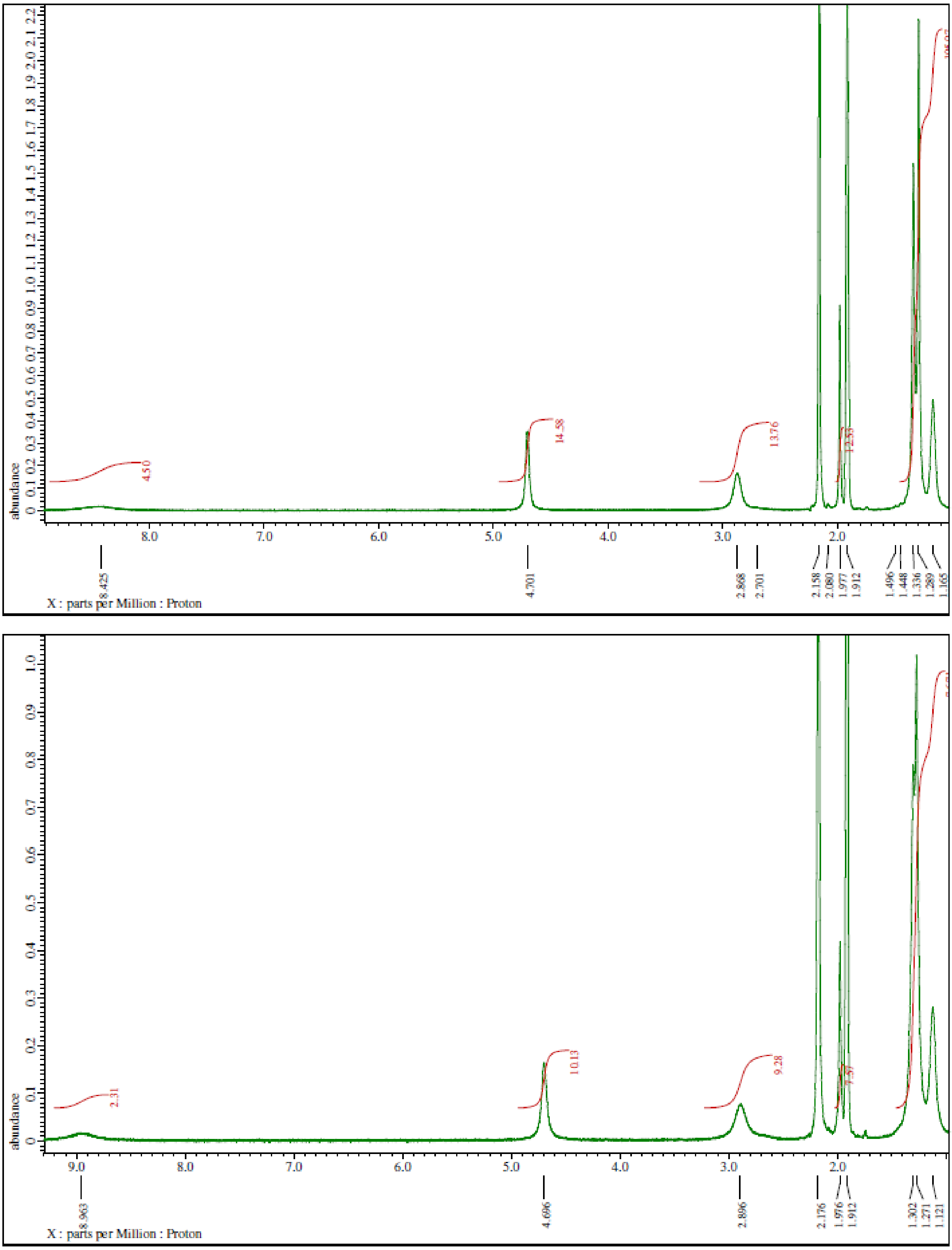

Figure S23. Top: ^{1}H NMR spectrum of ligand **23b** + $Y(NO_3)_3$ (0.5 equivalents) in CD_3CN. Bottom: ^{1}H NMR spectrum of ligand **23b** + $Y(NO_3)_3$ (1 equivalent) in CD_3CN. Peaks at 1.91 ppm and 2.17 ppm are due to solvents.

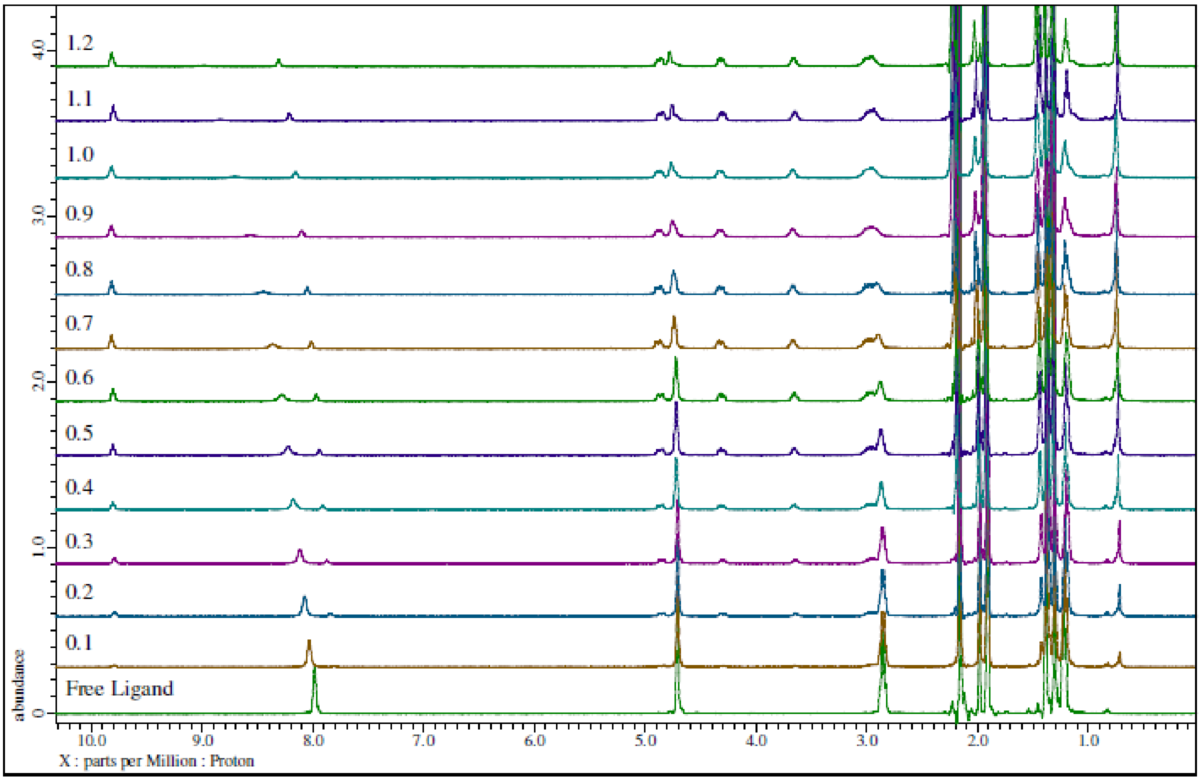

Figure S24. Stack plot for the ^{1}H NMR titration of ligand **23b** with $La(NO_3)_3$ in CD_3CN. Bottom spectrum = free ligand. Each preceding spectrum corresponds to the addition of 0.1 equivalents of metal salt solution.

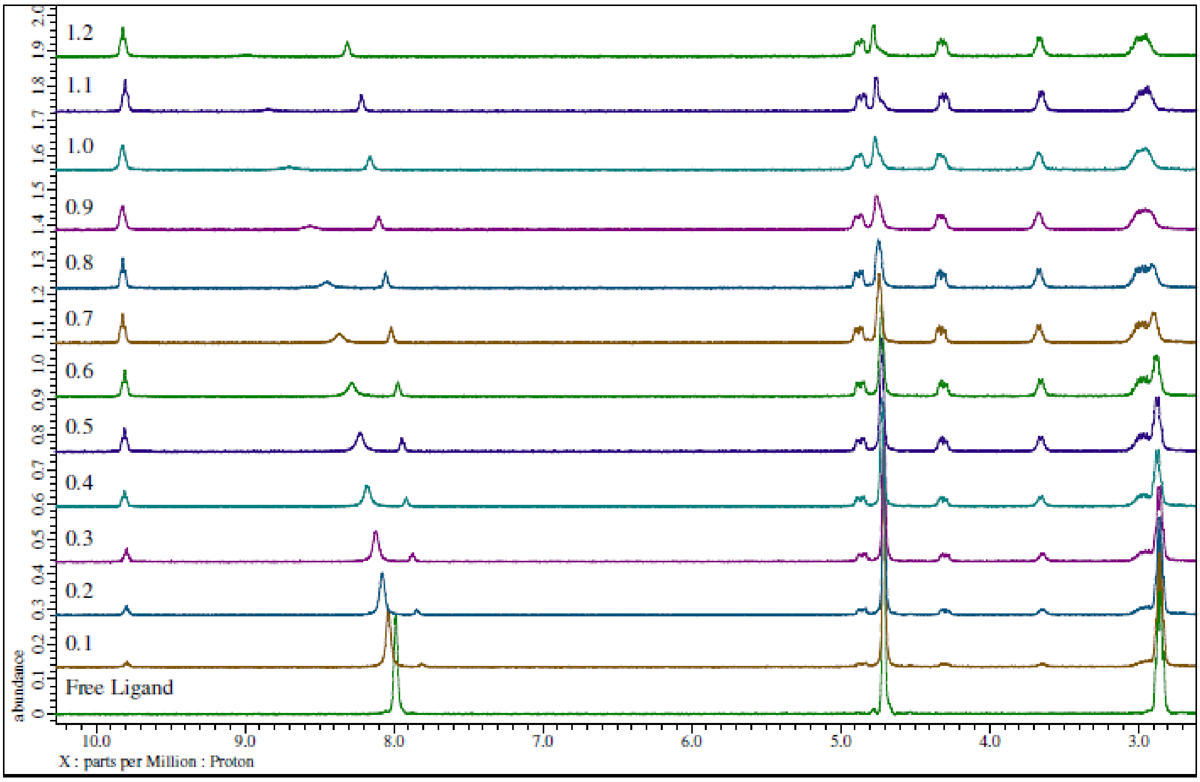

Figure S25. Stack plot for the ^{1}H NMR titration of ligand **23b** with $La(NO_3)_3$ in CD_3CN. Region between 2.6 ppm and 10.2 ppm is shown for clarity. Bottom spectrum = free ligand. Each preceding spectrum corresponds to the addition of 0.1 equivalents of metal salt solution.

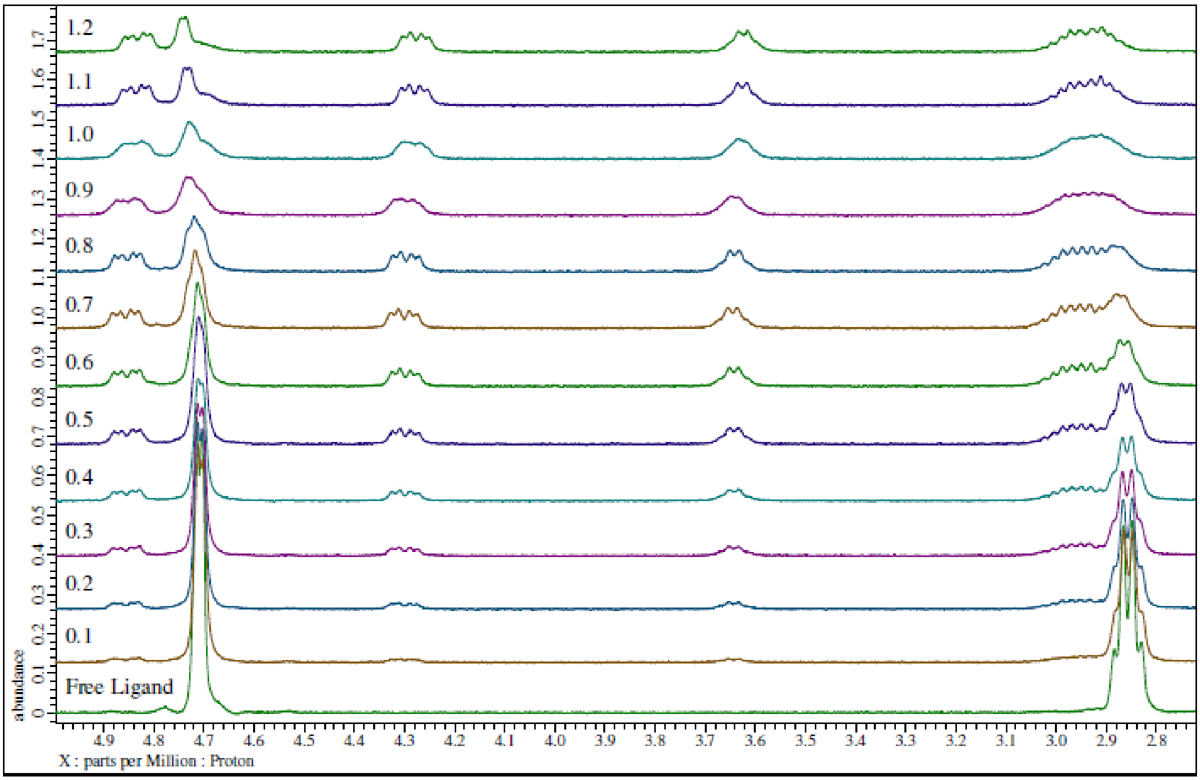

Figure S26. Stack plot for the ¹H NMR titration of ligand **23b** with $La(NO_3)_3$ in CD_3CN. Region between 2.7 ppm and 5.0 ppm is shown for clarity. Bottom spectrum = free ligand. Each preceding spectrum corresponds to the addition of 0.1 equivalents of metal salt solution.

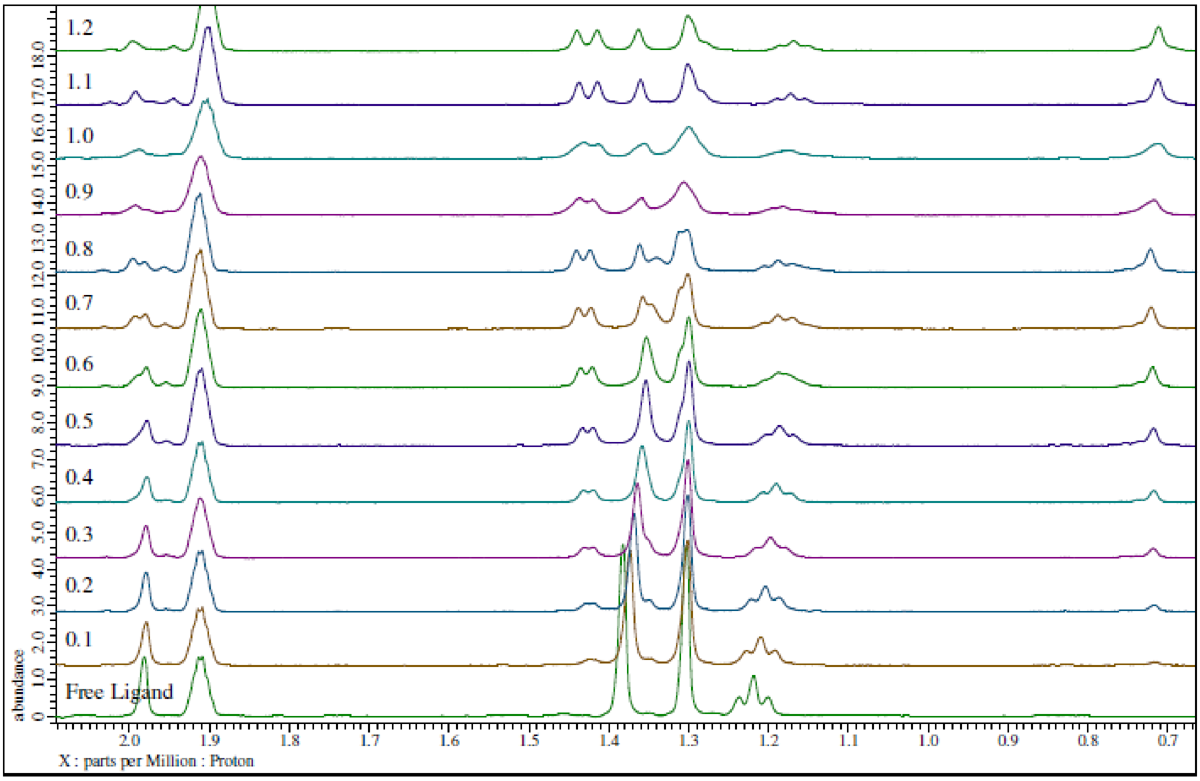

Figure S27. Stack plot for the ^{1}H NMR titration of ligand **23b** with $La(NO_3)_3$ in CD_3CN. Region between 0.67 ppm and 2.09 ppm is shown for clarity. Bottom spectrum = free ligand. Each preceding spectrum corresponds to the addition of 0.1 equivalents of metal salt solution.

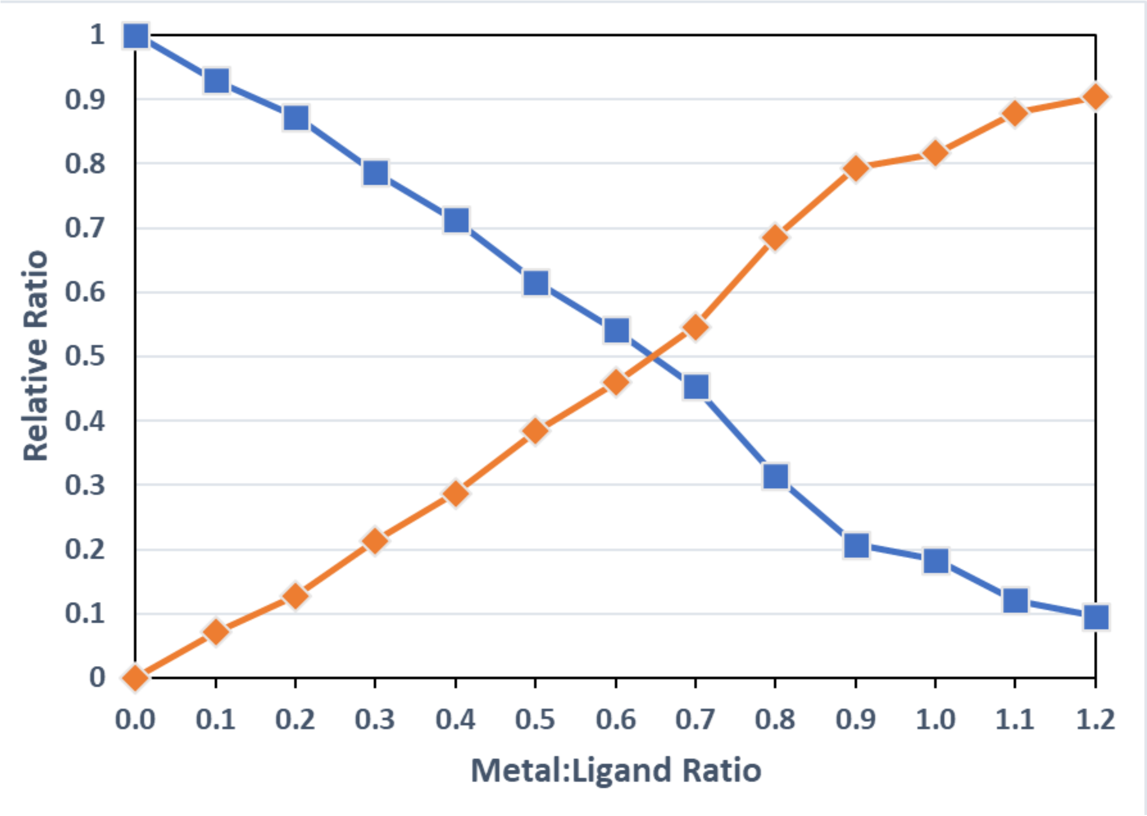

Figure S28. Species distribution for the ^{1}H NMR titration of ligand **23b** with $La(NO_3)_3$ in CD_3CN (■ = free ligand, ♦ = presumed 1:1 complex species).

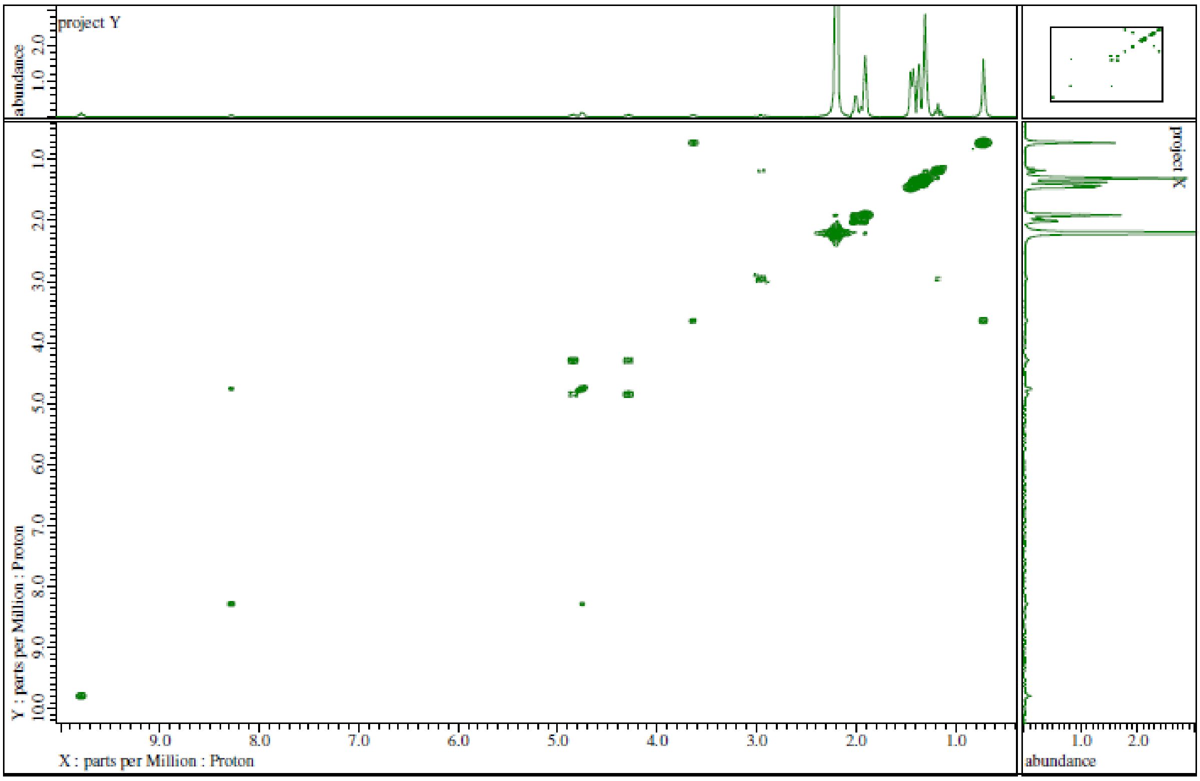

Figure S29. H-H COSY spectrum for the solution obtained at the end of the ^{1}H NMR titration of ligand **23b** with $La(NO_3)_3$ in CD_3CN.

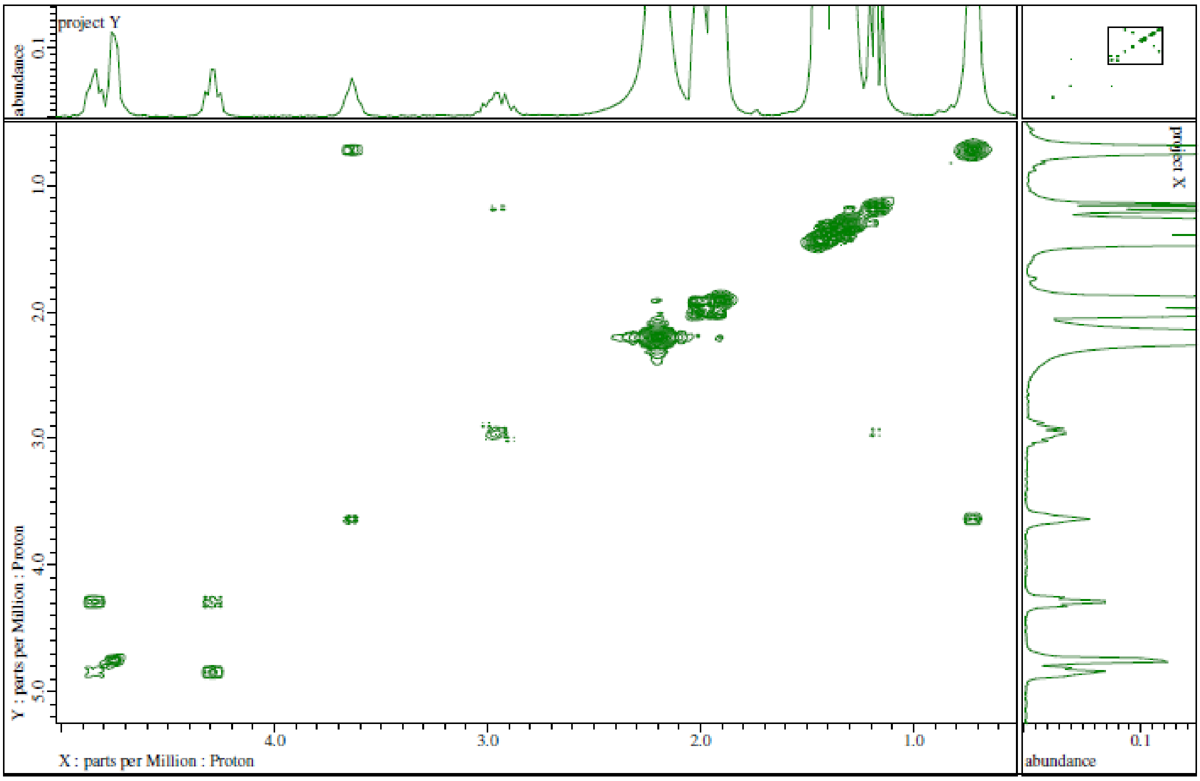

Figure S30. H-H COSY spectrum for the solution obtained at the end of the ^{1}H NMR titration of ligand **23b** with $La(NO_3)_3$ in CD_3CN. Region between 0.5 ppm and 5.0 ppm is shown for clarity.

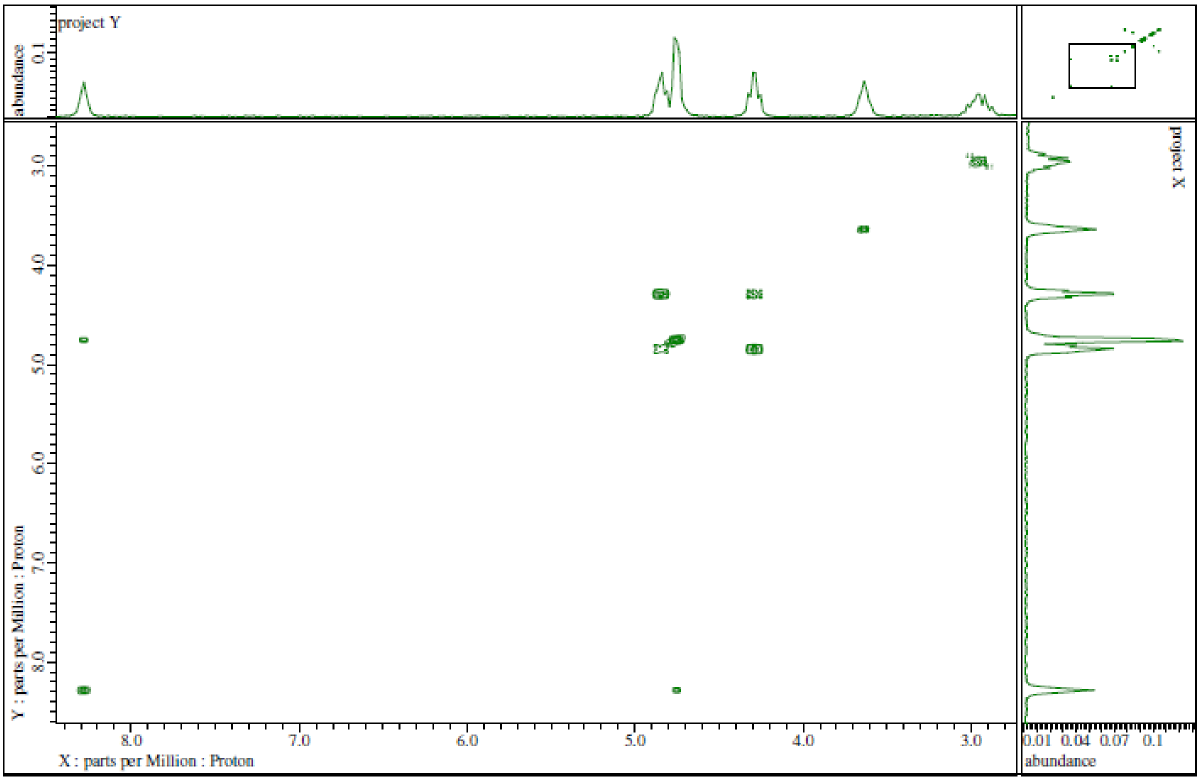

Figure S31. H-H COSY spectrum for the solution obtained at the end of the ^{1}H NMR titration of ligand **23b** with $La(NO_3)_3$ in CD_3CN. Region between 2.8 ppm and 8.4 ppm is shown for clarity.

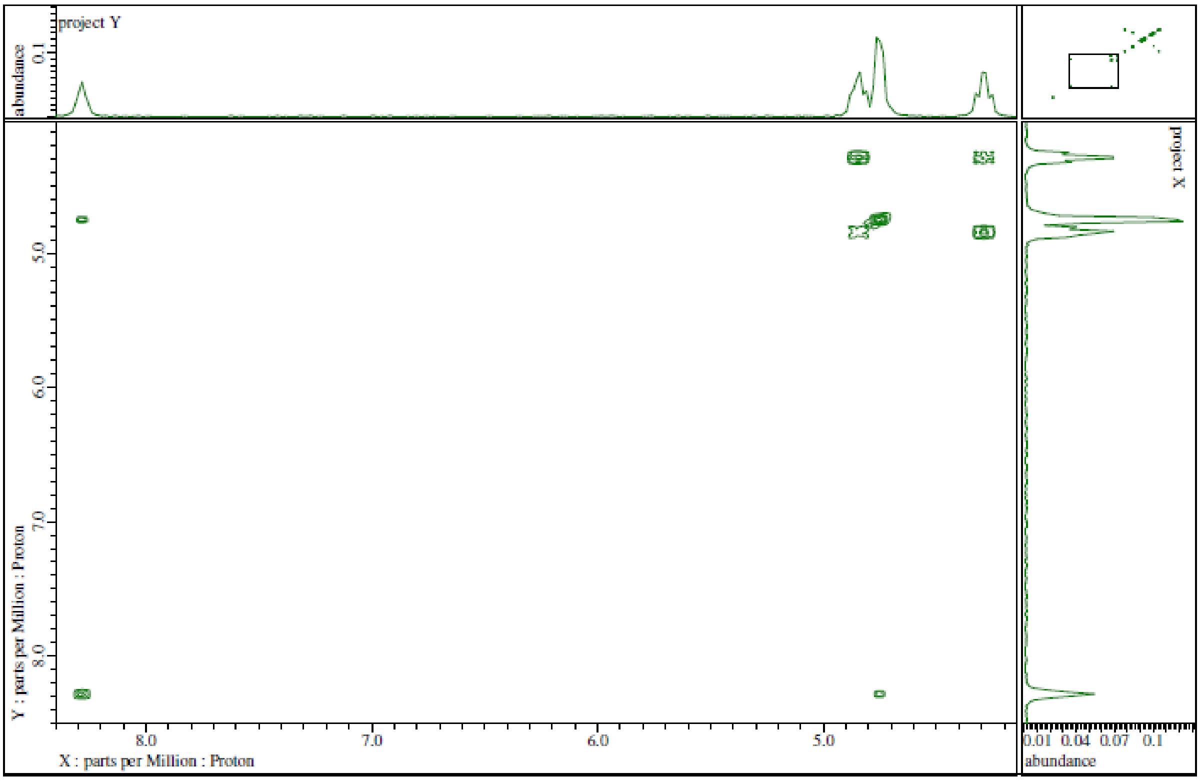

Figure S32. H-H COSY spectrum for the solution obtained at the end of the ^{1}H NMR titration of ligand **23b** with $La(NO_3)_3$ in CD_3CN. Region between 4.2 ppm and 8.4 ppm is shown for clarity.

5: DFT Calculations

Table S10. La(III) ligand complexes for which energies were determined. Complexes with the lowest calculated binding energies are shown in bold.

Ligand	Complexes	Nominal CN
**1**	La(κ^3-L)$(NO_3)_3(H_2O)$	10
	**La(κ^3-L)$(NO_3)_3(H_2O)_2$**	**11**
	La(κ^3-L)$(NO_3)_3(H_2O)_3$	12
**9b**	**La(κ^4-L)$(NO_3)_3$**	**10**
	La(κ^4-L)$(NO_3)_3(H_2O)$	11
	La(κ^4-L)$(NO_3)_3(H_2O)_2$	12
**12b**	**La(κ^4-L)$(NO_3)_3$**	**10**
	La(κ^4-L)$(NO_3)_3(H_2O)$	11
	La(κ^4-L)$(NO_3)_3(H_2O)_2$	12
**14b**	La(κ^6-L)$(NO_3)_2^+$	10
	La(κ^3-L)$(NO_3)_3(H_2O)$	10
	**La(κ^4-L)$(NO_3)_3$**	**10**
	La(κ^3-L)$(NO_3)_3(H_2O)_2$	11
	La(κ^6-L)$(NO_3)_3$	12
**23b**	La(κ^6-L)$(NO_3)_2^+$	10
	**La(κ^4-L)$(NO_3)_3$**	**10**
	La(κ^4-L)$(NO_3)_3(H_2O)$	11
	La(κ^6-L)$(NO_3)_3$	12

(CN = coordination number)

1: $L + La(NO_3)_3(H_2O)_5 \longrightarrow LaL(NO_3)_3(H_2O)_2 + 3H_2O$

9b: $L + La(NO_3)_3(H_2O)_5 \longrightarrow LaL(NO_3)_3 + 5H_2O$

12b: $L + La(NO_3)_3(H_2O)_5 \longrightarrow LaL(NO_3)_3 + 5H_2O$

14b: $L + La(NO_3)_3(H_2O)_5 \longrightarrow LaL(NO_3)_3 + 5H_2O$

23b: $L + La(NO_3)_3(H_2O)_5 \longrightarrow LaL(NO_3)_3 + 5H_2O$

Scheme S1. Reactions used to determine the binding energies of the ligands (L) with La(III).

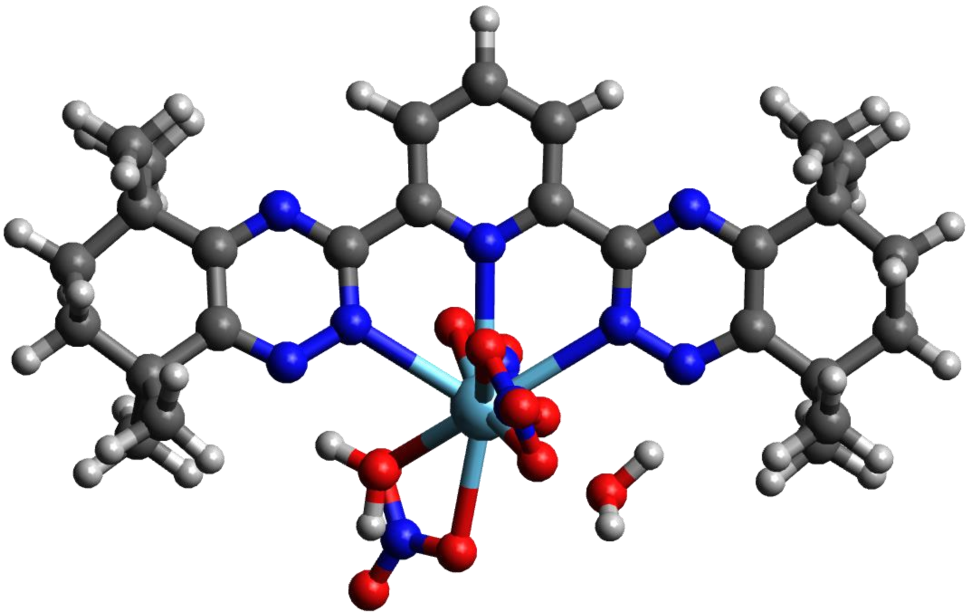

Figure S33. DFT optimized geometry of the La(III) complex of ligand **1**.

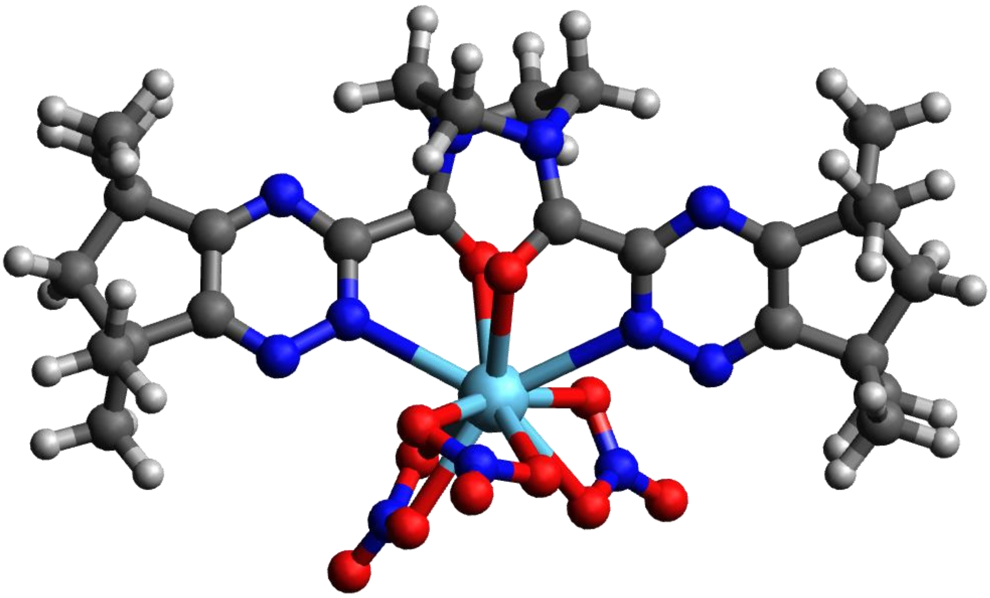

Figure S34. DFT optimized geometry of the La(III) complex of ligand **9b**.

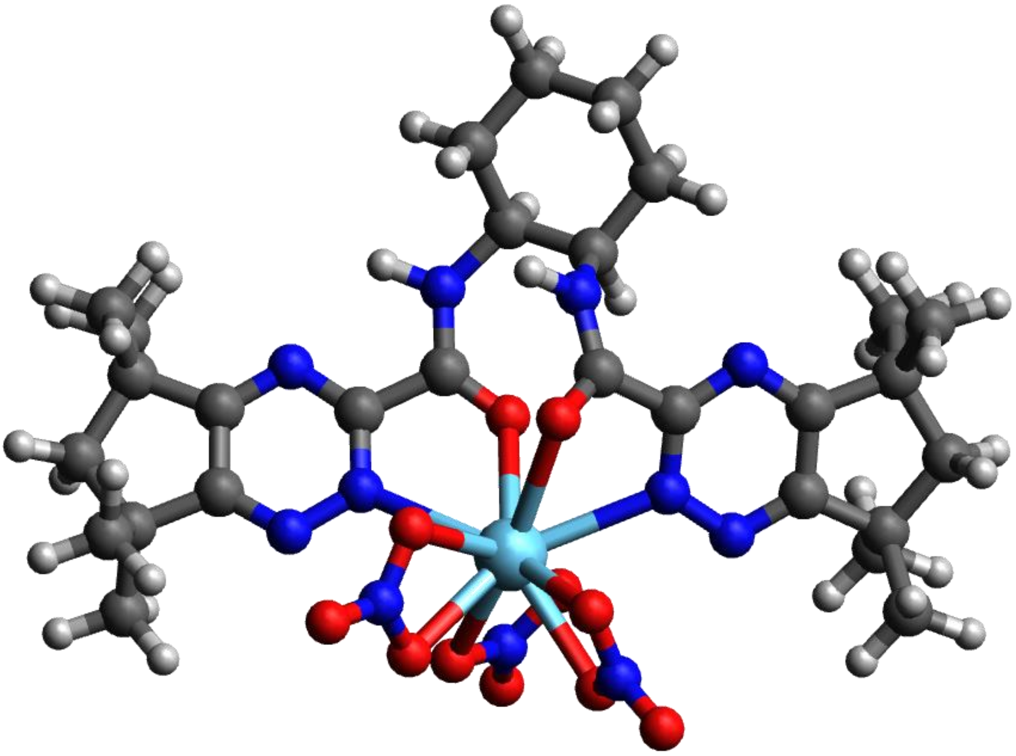

Figure S35. DFT optimized geometry of the La(III) complex of ligand **12b**.

5.1: Coordinates of Optimized Geometry of La(III) Complex of Ligand 1

La	0.64781518258811	0.14018084060310	0.04446501897803
C	3.97439118103322	-2.62738239842292	-4.20782382211724
C	2.64426768894235	-3.41443966665309	-4.29880102863533
C	1.67623648853180	-2.67545869195695	-5.26284457087023
C	2.93307286446611	-4.85235300520656	-4.80435467575113
C	1.76251628851118	-5.81264693386223	-4.57409421416422
C	1.45909303724541	-6.03852352120275	-3.07100401214518
C	0.06366049608381	-6.68849181443474	-2.90812859820833
C	2.52859774571885	-6.96115362025296	-2.42173903913764
C	1.48914326354194	-4.70450027322904	-2.33420070677855
N	1.05312017009286	-4.67263077681865	-1.07231647183086
C	0.99992758062027	-3.48938079968300	-0.43927445367585
C	0.61933360211570	-3.46144153303283	1.00010459740730
C	0.40615947719072	-4.67736117798731	1.68387364996069
C	0.10925228661220	-4.63835159644315	3.05152452239273
C	0.02638369060160	-3.39321040524031	3.68596199242809
C	0.23070752996118	-2.22543196142034	2.91957933660279
C	0.10322926787481	-0.89200690437216	3.57114247556927
N	0.15986557574833	0.20247252355642	2.78694726660154
N	-0.01598102770702	1.39220230072497	3.35364580792108
C	-0.22264691550883	1.52656813762327	4.67027877680175
C	-0.21938390096037	0.34512078288062	5.48893045528054
N	-0.07568358933882	-0.84520517851198	4.90173191170716
C	-0.40849527518051	0.37697834594199	7.00087811275105
C	-1.84228899133765	-0.14914242992369	7.29746613718127
C	0.62242605614648	-0.55492837621578	7.68042499412693
C	-0.23437125874333	1.82547414184323	7.52300566678685
C	-0.95802952848815	2.86243298398974	6.65923379298069
C	-0.40349960800928	2.94189788501320	5.21305306790727
C	0.98609619219100	3.63791899633393	5.19232348179827
C	-1.38925386916481	3.74372395258854	4.33050994044541
N	0.52603537611579	-2.25660065264164	1.60321473990740
N	1.30039729644635	-2.31718350575412	-1.03101221355658
N	1.82343468546059	-2.34385508820524	-2.25204153036145
C	1.98267870214122	-3.49152205507806	-2.92530835123911
H	4.46597931726592	-2.63524200642434	-5.19732558643579
H	3.81185730591200	-1.57564726439520	-3.91698890564001
H	4.66363570129075	-3.08990090862356	-3.47842119559542
H	2.14687182237246	-2.58937216522088	-6.25885668736731
H	0.71727829185780	-3.21107484722318	-5.37696324883070
H	1.45718355813942	-1.65961951836993	-4.89015300730785
H	3.18402216593413	-4.79767227273703	-5.87982482362946
H	3.83813407047618	-5.23674233397009	-4.29471388522857
H	0.85293559632060	-5.42120032108983	-5.07038021674296
H	1.97040333511243	-6.79358428034351	-5.04025374761901
H	0.04522760522201	-7.64662645555535	-3.45790342351737
H	-0.16515515040879	-6.89046986696977	-1.84813073097557
H	-0.72970807775348	-6.03935639801722	-3.32105533464271
H	2.49512167160888	-7.95252118787680	-2.90857431717921
H	3.54948163819691	-6.55484645855640	-2.53124069150429
H	2.32502458181891	-7.09171511394099	-1.34461595551966
H	0.48470909482563	-5.62134418367575	1.13579391840902

H	-0.05426756719765	-5.56450837485243	3.61476438651277
H	-0.19882388484727	-3.30281826141833	4.75292388015597
H	-1.97419372941921	-1.16842766820524	6.89384629996422
H	-1.99500263651661	-0.18277067224643	8.39135033918215
H	-2.62253555849271	0.49683602647924	6.85784989361318
H	0.49687275170894	-1.60062323002884	7.35343492791023
H	1.65666816132259	-0.23900792894245	7.45284714624533
H	0.48253661546812	-0.51159941778250	8.77547638603423
H	-0.60196242819979	1.86855204044949	8.56509053515975
H	0.84660694146418	2.06255999845139	7.56741000095543
H	-2.03903029075721	2.62589529348463	6.61378174815783
H	-0.87983207300751	3.86605517441718	7.11692120825488
H	1.72634856967295	3.10498644358111	5.81475332004397
H	1.37974341794138	3.69038510282189	4.16169128443895
H	0.88550727715750	4.66831632764772	5.57902377083228
H	-2.36540412875301	3.23158210542019	4.25495006272241
H	-1.55694172525232	4.73573834650628	4.78721442685201
H	-0.99709706134325	3.90099239826586	3.31177241201306
N	-2.38327172329599	-0.44266010816035	0.01252495727810
O	-3.59333988685343	-0.62673115619963	-0.09199149207787
O	-1.53937572724264	-1.39530062153902	-0.10684213674841
O	-1.89629941770251	0.72386531364359	0.24748188764222
N	3.50551591256990	0.53305565077771	1.14022625281507
O	4.68600365039555	0.66104063813361	1.45499749252369
O	2.78205521872339	1.53454855785921	0.80306281363045
O	2.92963461717478	-0.61312639292495	1.13035585636429
N	-0.28721937686501	1.25912513111583	-2.67332522957271
O	-0.69608932568483	1.69423747334572	-3.74711042783476
O	-0.45050114736606	0.03059999467800	-2.33651638304929
O	0.32610402155947	2.00119138638471	-1.82884947142941
O	2.54925383880825	0.16162094201741	-1.75178860926306
H	2.52593633110888	0.89408063585204	-2.39381764630464
H	2.39765681273133	-0.68377676445238	-2.26994215223231
O	0.19065181930507	2.56647875302508	0.97387973310311
H	0.99251109882595	3.11376379297888	0.88365560716007
H	0.10287034312353	2.40071012785691	1.96245270120879

5.2: Coordinates of Optimized Geometry of La(III) Complex of Ligand 9b

La	-0.21422388189693	0.08340135118706	-0.27848934753523
C	0.61279191505226	-6.45303243687904	-0.53995980738267
C	1.29724733976012	-5.73018920994593	-1.73077672747841
C	0.35528859534946	-5.73305089462672	-2.95288740062555
C	2.70984754263372	-6.33438037952360	-2.06591776568852
C	3.82752176535476	-5.44520215566599	-1.41209382907261
C	5.01354951439129	-5.19282573570494	-2.36761356356443
C	4.35303107685940	-6.03417921376283	-0.07450306783593
C	3.07032378862393	-4.16944393728852	-1.11943580338873
N	3.58596505069029	-3.00576899662476	-0.72863672918993
C	2.67314727083096	-2.03764676837145	-0.51631612264271
C	3.07391563706845	-0.69176880063771	0.05794910367214
O	2.22752327948037	-0.11674074199500	0.78711063561209
N	4.29589124432988	-0.17338535542105	-0.22877895499797
C	4.69038723501898	1.11684348713099	0.36953988868732
C	4.64651754209533	2.22948264657035	-0.72656478367501
N	3.68721949880329	1.77646093856410	-1.75202637807035
C	2.35000259587735	1.86800100664726	-1.54756968155265
O	1.51974613949658	1.03667084079272	-1.99711905649771
C	1.78789930086434	3.00925660918318	-0.71952559485691
N	0.67747323788448	2.66236586499903	-0.04231142333566
N	0.01750871830063	3.57674818937109	0.68020336453148
C	0.49059767685136	4.82240139181353	0.65901750413681
C	1.66337200495336	5.16152705523851	-0.07287411536748
N	2.33334500430296	4.24029374066768	-0.76326868671292
C	2.01775551456701	6.62469680290926	0.07000115673486
C	2.29293850005092	7.27721464089347	-1.30148525414223
C	3.27773513430176	6.74911873016122	0.96945847958986
C	0.72848688542009	7.19627289422573	0.76199428214241
C	-0.09579635680453	6.01258159603461	1.38930655827316
C	-1.61081889542108	6.15422743772905	1.13008309462197
C	0.15882713071860	5.85537618376041	2.91201617817432
C	4.22296470025688	0.64207124055564	-2.52967103581353
C	4.91219138118749	-0.36927885161183	-1.55464251367548
N	1.34411920400766	-2.15389466668868	-0.69298758311087
N	0.82164073082567	-3.31334316128061	-1.11354519895021
C	1.66755033042246	-4.32498456480980	-1.30562700895755
H	0.39280794948656	-7.49785438312539	-0.82384264838134
H	-0.33769924288075	-5.95329503122637	-0.28011625378775
H	1.25306768509334	-6.46743727534406	0.35932544542323
H	0.15698477037389	-6.77334284246415	-3.26798052237969
H	0.80402343718545	-5.19262809318772	-3.80546839778413
H	-0.61026991543831	-5.25617778904061	-2.70840761053727
H	2.84710025451989	-6.32422390875350	-3.16252406666222
H	2.78682177701684	-7.38567506251413	-1.73846788297760
H	5.50633246439029	-6.15187464028541	-2.60853800436530
H	5.76208410966666	-4.52853634419712	-1.90014253075138
H	4.67592501021121	-4.73072301072571	-3.31246623729815
H	4.88194081808994	-6.98237806195075	-0.27890451552879
H	3.53584519688523	-6.24183695640737	0.63811064802082
H	5.06125178823807	-5.33459560036921	0.40451693047922
H	5.71180018754400	1.01737817744146	0.77881017478907

H	3.99275566913894	1.34539130287913	1.18921778844467
H	5.62488369782334	2.36205018442842	-1.21763703081443
H	4.35056768544792	3.19860407997691	-0.30236044327938
H	3.16900894046152	6.81347770779603	-1.78879138613842
H	2.50185996098751	8.35353315913171	-1.16554548494523
H	1.42352712272864	7.17631166896689	-1.97547596069995
H	4.14591623989233	6.25988549349405	0.49265088242115
H	3.12449843906119	6.29193037249740	1.96244569677946
H	3.51496514263125	7.81826724177430	1.11476600750259
H	0.10648517901764	7.69460667008237	-0.00357859781956
H	0.98276990817704	7.95598543162389	1.52117278266040
H	-1.82937179742873	6.22134912094420	0.04934466098151
H	-1.98683880849683	7.07212353620795	1.61673878010718
H	-2.16319689058416	5.29289776843859	1.54588192937069
H	1.23299624223033	5.75352990518044	3.14536323332057
H	-0.36564065848663	4.96443645783413	3.30168918626583
H	-0.22601699006102	6.74592020811845	3.44062878795164
H	4.95187595216462	1.02997871453532	-3.26365912345971
H	3.38898438405023	0.16565582258652	-3.06621288264554
H	5.99274986276211	-0.17242921685418	-1.45678595726262
H	4.78916451175613	-1.40368481437119	-1.90289665889011
N	-2.77040323879707	1.66124262106358	-0.67161910639024
O	-3.81156231686146	2.28003342980962	-0.84473288375912
O	-2.50842951948000	1.06205219124568	0.43379527028075
O	-1.87141931961520	1.57334488365774	-1.58837965942120
N	-1.61430233707021	-1.62986347604581	-2.35346201577674
O	-2.19709769649375	-2.33422770220480	-3.16880025400363
O	-0.59040112901580	-0.92733074516231	-2.66538557424311
O	-2.00114159186833	-1.55408111499501	-1.12635677655377
N	-0.59450650610110	-0.52546046053428	2.66373712896806
O	-0.73548398078071	-0.81030517208292	3.84587962001671
O	-0.22469590084437	0.63823411210822	2.27967794881530
O	-0.81249989923762	-1.38955533757749	1.72943275190241

5.3: Coordinates of Optimized Geometry of La(III) Complex of Ligand 12b

La	-0.21555780749696	-0.05346637696964	0.01137988309241
C	3.33125242621883	-5.14387138913688	1.90029325454156
C	3.76482398569068	-4.74785253539905	0.47257965065696
C	3.03550035048349	-5.64486682166875	-0.56255143173032
C	5.32728589416677	-4.77638993396885	0.29501639988947
C	5.78538909647607	-3.50582485429610	-0.50724106994574
C	7.05603324967940	-2.85906509225829	0.08481276015219
C	6.00420327523815	-3.80054677112296	-2.01664844339992
C	4.58156031776500	-2.60347508141471	-0.36095806107980
N	4.49146308295574	-1.32082428294609	-0.70093646477414
C	3.28685865871950	-0.76426633883404	-0.46517534960434
C	3.05716478659037	0.69478430989544	-0.81170608978831
O	2.01654712606949	1.25601187003568	-0.38499863091681
N	3.99365698249291	1.22300585579777	-1.60701127835416
C	4.09151814865094	2.52192115714475	-2.30562573691690
C	4.82176908888673	2.26581020120170	-3.64460513385774
C	5.00694377322252	3.55361959468873	-4.46128714433080
C	3.66059362198393	4.25699468770144	-4.69211448848698
C	2.96743022844186	4.54423757880700	-3.35311898761175
C	2.74472498233563	3.28170905452688	-2.49380526990434
N	1.71236428255911	2.37443969036646	-3.03873136489019
C	0.57188742701092	2.06741050187966	-2.39070466873723
O	-0.00269583401782	0.95535322066597	-2.48336211822737
C	-0.08754363444241	3.11417886602778	-1.50794393653346
N	-0.70243697761570	2.59305798900648	-0.43066922183483
N	-1.42202657497126	3.37859975872833	0.37946766924327
C	-1.51667910816667	4.66569236114125	0.04609137492209
C	-0.84660151974418	5.19201856396263	-1.09276939866290
N	-0.10977049557417	4.40873243530059	-1.87994093368391
C	-1.07592689057327	6.68003940943317	-1.24747376780057
C	-1.49172723894988	7.04183531733161	-2.68930581429136
C	0.22942063225510	7.43367269274939	-0.87402822987598
C	-2.22828672252071	6.92958887143744	-0.20962505471332
C	-2.28804295974013	5.72775639865489	0.80241051960881
C	-1.55630769723155	6.04089297870753	2.13382703553362
C	-3.73676218278008	5.28413862645188	1.09554665429342
N	2.20857719659346	-1.38985186832619	0.03147751749867
N	2.28394308189298	-2.69088199977888	0.35454245886578
C	3.45742818060500	-3.29406035918457	0.17917283583886
H	3.63477671343337	-6.18618308331420	2.10573894075028
H	2.23442698844878	-5.07552741686672	2.01083751977322
H	3.80069490867295	-4.49072820012260	2.65753996108228
H	3.29260273323372	-6.70283189993262	-0.37513285160962
H	3.32285142480314	-5.39878493781320	-1.59978582607111
H	1.94049823218313	-5.52925575914116	-0.47278427776292
H	5.65924146889558	-5.70624254347764	-0.19853037144210
H	5.79879459280978	-4.75222414188885	1.29439000447176
H	7.90264823388211	-3.56428072763854	0.00374697328937
H	6.91770792660721	-2.60561550087603	1.15106839637708
H	7.32024939333289	-1.93703023370975	-0.46297855751982
H	6.85458845248318	-4.49626172121699	-2.13187923971902
H	6.23724879304811	-2.86907959377022	-2.56298486924739

H	5.11552537099171	-4.26098393679310	-2.48202294719203
H	4.78073805754011	0.58251233937217	-1.77872943138369
H	4.72416812270574	3.19270257678525	-1.68853105945270
H	4.25018954682030	1.52243625344088	-4.23720250420470
H	5.80203296031918	1.80113928482958	-3.42526664070463
H	5.49560729871293	3.30921882875050	-5.42236183221347
H	5.69247827835138	4.23703806405355	-3.92024245110956
H	3.00955197351173	3.61846543021706	-5.32348976761219
H	3.80590203901637	5.20217872036563	-5.24705511384766
H	1.98501714666471	5.02603259535352	-3.49550719131342
H	3.58608862447711	5.24558177297261	-2.75906859439105
H	2.39462129485187	3.59405361450100	-1.49676761329983
H	2.01833462755392	1.64523111513752	-3.69159251101369
H	-0.68295873799727	6.80585022962534	-3.40346915984493
H	-1.70585022845326	8.12374869425342	-2.75468359360098
H	-2.39923906825217	6.49038168798570	-2.99343735177651
H	1.04264208242831	7.17546588483114	-1.57552415945214
H	0.56693588536647	7.19488655483509	0.14967619894824
H	0.04966081521238	8.52218429874056	-0.93292309787671
H	-3.18900728536060	6.98383196004520	-0.75371149228960
H	-2.09829285940875	7.89186880516300	0.31540074417529
H	-0.51446404162164	6.36554797147142	1.96633994368255
H	-1.53863878847815	5.14913349478092	2.78587251093864
H	-2.08851010444357	6.85096795308846	2.66436384867562
H	-4.26833150098725	5.01289437602750	0.16583681824473
H	-4.28861809840076	6.10914677925855	1.58109930724856
H	-3.75316975947733	4.41228932051589	1.77366430928317
N	-3.22547956117344	0.14395239146662	0.19796239089165
O	-4.44394456675584	0.21708288324223	0.27687435924735
O	-2.62376603509789	-0.01115963272299	-0.92772291911197
O	-2.46986252732190	0.21961630572858	1.23585557697914
N	0.35992484788788	0.21647984818063	2.97787831250855
O	0.58718429990452	0.31310864003809	4.17694774279166
O	0.34557444752886	1.23978390267272	2.20162085262719
O	0.12118299338653	-0.91751669532647	2.41988327564226
N	-0.78532284138796	-2.84980026898647	-1.01389750543310
O	-1.00560310654470	-3.98461406238591	-1.41833541708569
O	-0.24627777779964	-1.95373415366782	-1.76087947487519
O	-1.07932791926460	-2.48511635441924	0.18321991064544

5.4: Coordinates of Optimized Geometry of La(III) Complex of Ligand 14b

La	-0.01066534076943	-1.15618296998722	-0.21384553029533
C	-2.96726625909780	-6.13145390036918	2.67578438065453
C	-1.52594493489011	-6.11479684100917	3.22898649915573
C	-1.51052230011766	-5.42899915421433	4.62070349718201
C	-0.88411824270853	-7.55022044009440	3.25692923913457
C	0.59702406569917	-7.47027659925175	2.73984603073272
C	0.95894543198415	-8.63494659681397	1.79471312442552
C	1.62714374729563	-7.39948659711450	3.90081910536255
C	0.59281669660095	-6.14239570276529	2.01641447034270
N	1.53553105779586	-5.65982771447121	1.21261216257439
C	1.27432779712251	-4.43078040707933	0.72138830364035
C	2.27378806926390	-3.78777108350922	-0.20883771435411
O	2.01631879143942	-2.65756143936468	-0.70243582608081
N	3.38304757724966	-4.48849333416081	-0.45315092304652
C	4.47568550545995	-4.02291197317870	-1.30078628020556
C	5.63231461891789	-3.37776409279826	-0.50051340036650
N	5.22985843503646	-2.22874469359894	0.31754280078864
C	5.22709691992520	-2.45003786560362	1.76754053983987
C	3.95008565484158	-2.00289726980139	2.50747333391796
N	3.75522705217189	-0.56132446958787	2.55843477061883
C	2.70319721588453	0.11023974347297	2.06648250712004
O	1.74212532291584	-0.41034772670166	1.45431443985841
C	2.74929901797079	1.60854203096356	2.33663408438998
N	3.87031379994639	2.06115613544426	2.92121521496750
N	3.98666508217158	3.36378840770798	3.21509529266416
C	2.95366410068944	4.15046519569436	2.90782678862567
C	1.78224783971514	3.63922377741844	2.28566434591677
N	1.67315694159085	2.34633150926581	1.98859376198141
C	0.74282888013323	4.72091066222048	2.06304414269200
C	-0.41813160554647	4.51335299654574	3.07209097049000
C	0.18598119628480	4.70449302452325	0.62436297973070
C	1.57309911664611	6.02007726811717	2.36478766259970
C	2.85981388273365	5.63798331368643	3.18136206009944
C	4.11202995574303	6.39249436961049	2.68825101447530
C	2.68239900441163	5.86301987023832	4.70603677972776
C	5.79011292320887	-0.93884367743585	-0.10126907470753
C	5.17797339552048	-0.40254108603470	-1.40739688668401
N	3.72034500487139	-0.38577931628466	-1.30133599228199
C	2.81991621139281	0.54016454771605	-1.65087519832447
O	1.59064366992187	0.41333589063854	-1.39798670361128
C	3.26500217264797	1.77945282187496	-2.40459804168439
N	2.68801759370832	2.92355971740815	-2.00297529300449
N	2.97501644105954	4.06180354000549	-2.65785278227937
C	3.80479152234811	3.98366313036908	-3.69948460151628
C	4.39882651792318	2.75460216389899	-4.08945786781387
N	4.13787274590048	1.62884986060554	-3.42853587947027
C	5.30686963597002	2.91634544943403	-5.29243149629226
C	4.62581482626178	2.27457383333929	-6.53052159505493
C	6.68315854443191	2.25893859193941	-5.05872315448055
C	5.40353571138809	4.48098595373532	-5.40300858478505
C	4.22537326191733	5.13473827272920	-4.59282542685764
C	4.68607880208405	6.35712749011531	-3.77219251040942

C	3.03500114427933	5.53390229180793	-5.50369743535128
N	0.18970630850944	-3.68365768773401	0.98366869079724
N	-0.76709278370802	-4.16795719261716	1.78977673802570
C	-0.57975873250252	-5.38394314811287	2.29864253455789
H	-3.61440692808157	-6.73216862057646	3.34005655329175
H	-3.00319863846207	-6.57488338223082	1.66469696584916
H	-3.37982606734223	-5.10823042897789	2.62418510954646
H	-2.16455504649035	-5.98916522152640	5.31291629578686
H	-1.88752136128274	-4.39328632665547	4.54491645250071
H	-0.49625833626462	-5.39663492511917	5.05552978283492
H	-1.45892274459094	-8.20695406896710	2.57874483541460
H	-0.93601101204870	-7.99835698102040	4.26440478637166
H	0.90319327766751	-9.59127885000238	2.34507407909730
H	1.98588197501039	-8.52026974767081	1.40440577687471
H	0.26301037052823	-8.68834845352771	0.93842817545600
H	1.60115365572510	-8.34714820342226	4.46799077611586
H	1.40676152754932	-6.57321449178185	4.59854508198495
H	2.64859786695688	-7.25595668811236	3.50517229122012
H	3.45813620657488	-5.39346646453833	0.02502385033568
H	4.05612745826049	-3.30519765324131	-2.02558528214065
H	4.86379266101284	-4.89023621923364	-1.86429000365741
H	6.40387269113629	-3.07812374172238	-1.23787732758023
H	6.09931567833054	-4.15709181900375	0.13377489162877
H	6.10823998410249	-1.97362263153282	2.25412942040484
H	5.32028679397371	-3.53357500326971	1.96025363353782
H	4.01102966165720	-2.37952360659124	3.54719603965327
H	3.05541686188655	-2.44290611985476	2.04107937067604
H	4.44523109975471	0.02645906078157	3.04415064929151
H	-0.93006647141783	3.55239825920609	2.88392415840839
H	-1.15505951364990	5.32866199283797	2.95632149819609
H	-0.06472747144091	4.51636327912555	4.11818231620466
H	-0.36636180810864	3.76960136325628	0.42400079507746
H	0.99600526908335	4.79826364255077	-0.12006777755754
H	-0.50941861842876	5.55241894650523	0.48643343605173
H	0.96573966450886	6.77581916745240	2.89320052251771
H	1.88490694154263	6.46875688165644	1.40361113129958
H	4.27647251963187	6.23124301206865	1.60772560391763
H	5.01268473324919	6.05428358679952	3.23121491619212
H	3.99089422349244	7.47729355896964	2.86108297043915
H	1.79287448872713	5.34115433190239	5.10030250299810
H	2.56730072049483	6.94278094075176	4.91087260033488
H	3.56763009565931	5.49481258772520	5.25545580634713
H	6.89615396844117	-0.98431856784393	-0.23852013437812
H	5.59231109820477	-0.20444596681270	0.69816963189693
H	5.47534651032825	-1.03324554763949	-2.26802414353631
H	5.56383351277616	0.61001717045639	-1.60217027720252
H	3.31436241747204	-1.24118048318507	-0.88039413532578
H	4.49012515211340	1.18845481373069	-6.37903448027439
H	5.26284253773112	2.42594883049346	-7.42066364466062
H	3.63652028326549	2.71968206278838	-6.73544248369626
H	6.58169202398062	1.16620222360459	-4.93282211612743
H	7.17451181672703	2.66993943414918	-4.15856809277668
H	7.34022468891205	2.44725664279165	-5.92699836740844
H	5.40532963815226	4.81316763845476	-6.45595511680041
H	6.36163393851396	4.80661977950271	-4.95792792119582

H	5.51642438028979	6.09227223392300	-3.09321402638307
H	3.85462462165235	6.75633139522144	-3.16434762429441
H	5.03568613819900	7.15786774673256	-4.44889158971210
H	2.67406896017172	4.68272738656066	-6.10738770104422
H	3.34853521251913	6.33754359983272	-6.19437257503666
H	2.19069017580874	5.90606878388549	-4.89558773808471
N	-1.85446696573923	1.18955711308164	-0.87197983821803
O	-2.61024681421090	2.12255759864370	-1.12345858117310
O	-1.18415746411689	1.13736669756218	0.22176985531788
O	-1.68786177002518	0.21240395803021	-1.68612681527213
N	-0.65425653947325	-2.69728880623192	-2.79635739103750
O	-0.93518727406555	-3.34302542392078	-3.80294834404128
O	0.18708276295530	-1.73742063799982	-2.81440424563179
O	-1.19855598207945	-2.95714067851232	-1.65787494132532
N	-2.14898978756619	-1.42514815173848	1.95541209825245
O	-3.01377332257688	-1.54946684043982	2.81873712816662
O	-2.40395833523989	-1.61618020653080	0.71181506148448
O	-0.94515700367591	-1.09837364243250	2.24388579646465

5.5: Coordinates of Optimized Geometry of La(III) Complex of Ligand 23b

La	0.55501883811839	-0.40957188419579	-0.18413548546064
C	-4.18370441488736	-5.34914070429854	0.15665845744936
C	-3.23172821529935	-4.13926038565841	0.03812076497080
C	-3.40021604992452	-3.14503426287165	1.18022043568912
C	-2.76121504805042	-3.36142264143246	2.42854940104686
C	-1.74811533543064	-4.49673992123822	2.57961403700208
N	-0.34481013247183	-4.04906873259642	2.67016920462203
C	0.24770592077776	-3.14428928057312	1.88215121660391
O	-0.29714298548214	-2.55705752911391	0.91914151585572
C	1.68520391867196	-2.82467329474130	2.23312308324171
N	2.22524598874441	-1.81923652503994	1.52456284552527
N	3.50361715322179	-1.46742115087962	1.73607513283001
C	4.18168573233038	-2.15732978854167	2.65088740817371
C	3.57551612935449	-3.20867416382498	3.39751413402772
N	2.30606372933569	-3.54549404826094	3.18955727834999
C	4.53269252841849	-3.82038854696298	4.39608825659066
C	4.52252778505131	-5.36194710976886	4.32337810778403
C	4.12709959851056	-3.36161675730827	5.82358303816591
C	5.90154443444821	-3.20533541416422	3.93274643243658
C	5.62920031958391	-1.93800376157003	3.04252419615274
C	6.55213626012644	-1.88502551420685	1.80634689621602
C	5.75145793704349	-0.61349423393849	3.84035724993605
C	-3.03555106674906	-2.52067339615582	3.54367361351628
C	-2.34717290833987	-2.80779974747747	4.87751975609326
C	-3.11820799261430	-3.78752947756102	5.78715648254250
C	-3.95161256307448	-1.44049580995033	3.39517993846220
C	-4.28446649920139	-0.55403861178372	4.60492542616599
N	-5.70235304360672	-0.39006281561484	4.93393686283120
C	-6.54041668876092	-1.38039344001246	5.32966911766945
O	-6.24639295323443	-2.57695252641154	5.42974475599701
C	-7.94351903764077	-0.86189192080096	5.65342148518059
N	-8.84267559802891	-1.78777655953802	6.01849173213235
N	-10.09344335186860	-1.39494157626255	6.32691636702221
C	-10.37300725474358	-0.09397637616850	6.25293623060877
C	-9.39779940725338	0.85836501918326	5.85576016212764
N	-8.16371473911816	0.47363145677378	5.55021184119728
C	-9.94535493221363	2.27231027689186	5.85396298122157
C	-9.33039923775497	3.05648879369775	7.04316281627347
C	-9.62769879812685	3.00348645014889	4.53275242400321
C	-11.48433152262533	2.01180777688721	6.03804009773959
C	-11.70568314961118	0.55242236861809	6.58063048617073
C	-11.92284913116398	0.51985525247364	8.11583856510812
C	-12.87603213678447	-0.16283927644036	5.87497420750620
C	-4.48632645404283	-1.12940408485166	2.11129009575391
C	-5.28128066994025	0.15722573143393	1.91202641409836
C	-4.41148895237959	1.34160952765179	1.44047463675051
C	-4.21297428537663	-1.99195014934622	1.01357358748769
C	-4.81590896523970	-1.70211057581862	-0.35591157437299
N	-3.87754603675381	-1.36839028386574	-1.44493881362452
C	-2.61510308243096	-0.93353589418172	-1.38667282726728
O	-1.96474746753495	-0.66301600807361	-0.34946407973638
C	-1.93434700080723	-0.80234023555503	-2.73587787891223

N	-0.62909335104622	-0.49435182028075	-2.67438047580967
N	0.07926602235940	-0.36048862508332	-3.80729337751263
C	-0.57265786452355	-0.52904951749971	-4.95542166659779
C	-1.95909087647203	-0.85832254481656	-4.98804828759566
N	-2.64959352088602	-1.00417825752530	-3.86142005442559
C	-2.46814814573650	-1.03193588691148	-6.40147703392926
C	-3.77386951866135	-0.24307944216413	-6.63654319918545
C	-2.71743661042959	-2.54316494126892	-6.65852165341211
C	-1.26588739646312	-0.46905532614299	-7.24079019537982
C	0.02556930482083	-0.43229865697875	-6.34392146511689
C	0.94912527223498	-1.65489209349088	-6.58897977790301
C	0.82742001246447	0.87273751953679	-6.53266930102587
H	-4.04123307765180	-6.04302772505538	-0.69163888951420
H	-5.24052075181357	-5.02472903757223	0.15728434852323
H	-4.01170148242365	-5.91437595759558	1.09099305734908
H	-3.40450861353143	-3.64879350362283	-0.93285068114023
H	-2.18601253703323	-4.48976295311693	-0.00970621643649
H	-1.82826879101559	-5.21411769264445	1.74698330723335
H	-1.93376449764538	-5.07542355986620	3.49854644865463
H	0.25724806415946	-4.46755348453816	3.38738575162159
H	3.53266670842755	-5.76308644176356	4.60487065007153
H	5.27312459628297	-5.77213126980499	5.02267142991175
H	4.76748496444155	-5.71661755127160	3.30625178801048
H	3.12214808554077	-3.74111725146411	6.08127647490224
H	4.11593193924184	-2.26188678495495	5.91786604342202
H	4.85176074742367	-3.76356345863276	6.55416371725091
H	6.43474904174649	-3.95612690641020	3.32141737431285
H	6.54960185759578	-2.96585686068040	4.79369382924065
H	6.45935766686857	-2.80174075581949	1.19697555801021
H	7.60444164403241	-1.78986212479260	2.12962275462394
H	6.30627514662522	-1.01637403758769	1.16992490186663
H	5.10535117444576	-0.60505020408458	4.73541540794177
H	5.47146017163415	0.24674868025651	3.20610976940120
H	6.79746800379701	-0.47818097239325	4.16949464312202
H	-2.17016660123261	-1.86657061929413	5.42682361406336
H	-1.33481687320747	-3.20228266220365	4.68945911074108
H	-4.13700967602643	-3.41456454897720	5.98937408516718
H	-2.58737040584742	-3.92594804622168	6.74661211414414
H	-3.22115023705211	-4.78136949166289	5.31308793932545
H	-3.89424547318944	0.46618419033577	4.43940752751240
H	-3.76882380536533	-0.93868321281372	5.49873697482164
H	-6.09736063037610	0.55388234193139	4.96940791937147
H	-8.23370568231782	3.13354786699113	6.93158376035157
H	-9.54457302153618	2.57289578350234	8.01235773313686
H	-9.75085017556329	4.07810834634415	7.06810510633532
H	-8.53714562469029	3.11924300877024	4.39953745666662
H	-10.08298096758112	4.01032825299524	4.54238796372118
H	-10.02820067883169	2.45198696172968	3.66317195520724
H	-11.97772487699900	2.10049205643229	5.05255954526569
H	-11.94689210497457	2.76511365453753	6.69988943568207
H	-11.10325094852380	1.02257917497280	8.65897555559739
H	-11.98162820346684	-0.52395636683589	8.47396060043080
H	-12.86923806961923	1.03041702194485	8.37098564205881
H	-12.73467822561591	-0.17642542347295	4.77917106388277
H	-13.82624065642618	0.35745078493616	6.09388794129695

H	-12.96424546107960	-1.20692934682930	6.22517675496086
H	-5.78525560259700	0.43189917384072	2.84918067348870
H	-6.10626975947705	0.00272488405404	1.19345453837330
H	-3.58923682654412	1.53914393644940	2.15194016760850
H	-5.01956833719819	2.26055484493570	1.35651788851791
H	-3.95217985251535	1.14482860088904	0.45606130087767
H	-5.55269601157791	-0.88464275060626	-0.29851284579820
H	-5.37580565294201	-2.58542115447297	-0.71532793346941
H	-4.22871498272322	-1.50364667991670	-2.40068049121104
H	-4.58545467575121	-0.62452056684008	-5.99168319936094
H	-4.08903121114484	-0.35178749270778	-7.68982506209109
H	-3.63582521731573	0.83253020164595	-6.42610802054879
H	-3.51536254447490	-2.92248361416206	-5.99521363408072
H	-1.81007960374106	-3.14801117820595	-6.48767841729397
H	-3.03592183384792	-2.68605492483328	-7.70668778814240
H	-1.50742835815050	0.56258926231856	-7.55553085499497
H	-1.10161076085797	-1.05945157390379	-8.15875958516192
H	0.41067488941279	-2.61117803102623	-6.46901097054688
H	1.79869742174396	-1.64799328617981	-5.88275258784543
H	1.34905318968994	-1.61237859378143	-7.61804437305137
H	0.20268254331201	1.75988571551437	-6.32509874228196
H	1.18813718809763	0.94036983621848	-7.57489799381183
H	1.70490551319438	0.89897946189720	-5.86219508035625
N	0.82188350826825	1.03208877371114	2.48449397811536
O	0.91281553230729	1.62521283812174	3.55318947212029
O	-0.10434584550143	0.16756729128798	2.26506964681675
O	1.64501163277125	1.23633245321768	1.52170141938265
N	0.35106288175614	2.50979155157343	-1.07042112173921
O	0.28436635507503	3.69184747766609	-1.39234436306748
O	-0.56876548433879	1.94519498266407	-0.37413451903809
O	1.33968964278673	1.76783149347940	-1.41092493800175
N	2.51471004829813	-1.97943506665199	-1.92188450065720
O	3.30835838618536	-2.58837046668963	-2.63090533959088
O	1.41325350688137	-2.50520460261971	-1.52671263414475
O	2.73980616646310	-0.77506131466061	-1.53266738619827
